# Quantitative Phosphoproteomic Analysis Reveals Key Mechanisms of Cellular Proliferation in Liver Cancer Cells

**DOI:** 10.1038/s41598-017-10716-0

**Published:** 2017-09-07

**Authors:** Bo Zhu, Quanze He, Jingjing Xiang, Fang Qi, Hao Cai, Jun Mao, Chunhua Zhang, Qin Zhang, Haibo Li, Lu Lu, Ting Wang, Wenbo Yu

**Affiliations:** 1 State Key Laboratory of Genetic Engineering, Department of Genetics, School of Life Sciences, Fudan University, Shanghai, P.R. China; 2 Center for Reproduction and Genetics, Suzhou Municipal Hospital, Suzhou, Jiangsu China; 3 The Second Department of Surgery, Hospital of China No. 17 Metallurgical Constrution Corp, Maanshan, 243000 Anhui, P.R. China

## Abstract

Understanding the mechanisms of uncontrolled proliferation in cancer cells provides valuable insights into tumor development and is benefit for discovering efficient methods in cancer treatment. In this study, we identified and quantified 2,057 phosphoproteins and 9,824 unique phosphosites in three liver cell lines with high (QGY, Hep3B) and low (L02) proliferative potentials and disclosed the wide variations in phosphorylation sites and levels among them. We found that the number of identified phosphoproteins and phosphosites in these cells were negatively correlated with their proliferative abilities. The function analysis suggested that the aberrant phosphorylation of SR proteins and activation of MAPK pathway might be two critical factors to promote cancer cell proliferation. Meanwhile, the phosphorylation status of mini-chromosome maintenance (MCM) and nuclear pore (NPC) complexes are significantly different between cell lines with high and low proliferative potentials. Furthermore, the phosphosites targeted by kinase families of CDK, STE and HIPK in the proteins coded by cancer driver genes showed distinct profiles between caner and normal cell lines. These results present key phosphorylation networks involving in abnormal proliferation of cancer cells and uncovered potential molecular markers for estimating the proliferation ability of liver cancer cells.

## Introduction

Liver cancer is the sixth common cancer, with nearly 782,500 new cases and 745,500 deaths globally occurred in 2012^1^. Its incidence rate and the mortality rate are the tenth/fifth and third/first in all cancers with men of America in 2017^2^ and China in 2015^3^, respectively. The high mortality rate generally blames on the lack of highly effective methods to diagnosis cancers in early stage and the poor prognosis^4^. As the proliferative capacity of cancer cells is an key indicator of malignant grade of cancers, exploring the essential biological pathways responsible for uncontrolled proliferation of cancer cells is not only important to deepen our understanding of the mechanisms of cancer development but also valuable to discover new diagnosis and prognosis biomarkers to improve cancer treatments.

In the past decade, many genes have been reported to promote or repress cellular proliferation of cancer cells, such as TP53, KRAS and PI3K, by regulating multiple biology processes of gene expression, cellular motility, cell cycle regulation, response stress, DNA repair and metabolism^5–7^. It is well established that these proteins and most of these pathways are tightly controlled by multiple mechanisms including protein phosphorylation^8–10^. Accumulated evidences supported that aberrant protein phosphorylation takes an important role in cancer development and progression^11–13^. For example, dysregulated kinase signaling pathways were commonly observed in various cancers including gastrointestinal stromal tumors^14^, lung cancer^15^, pancreatic cancer^16^ and breast cancer^17^. Recently, cancer genome sequencing showed that codons of phosphosites have significant higher mutation frequencies in cancer samples^18, 19^ and were mutated in a cancer type specific manner^20–22^. It suggests that these mutations in phosphosites may confer selective/growth advantages on cancer cell to achieve clone dominance^12, 23^.

Although, many efforts have been made to explore the relationship between abnormal protein phosphorylation and cancer cell proliferation, the detailed landscape still remains to be elucidated^24, 25^. Fortunately, the recent advance in proteomic technologies presents a powerful solution to profile site-specific phosphorylation events on thousands of proteins in a single experiment, which allows researchers to investigate aberrantly phosphorylation events in a global fashion^8, 24^.

In this study, we used TiO_2_ based phosphopeptide enrichment method combined with high resolution tandem mass spectrometry (MS) to screen and compare phosphoproteome in three liver cell lines (two human liver cancer cell lines (QGY and Hep3B) and one immortalized normal human fetal liver cell line (L02)) with different proliferation potential. Totally 2,057 unique phosphoproteins were quantified and 9,824 unique phosphosites were identified in three cell lines. The enrichment analysis of Gene Ontology (GO) and KEGG pathway suggested the preference of phosphoproteins in the highly proliferative liver cancer cells (QGY) for the biological processes including RNA splicing, DNA, chromatin and histone modification, and signal response. Further analyses indicated that the aberrant phosphorylation profiles of SR protein family resulted in the abnormal splicing of mRNAs of several key cancer related genes. Additionally, the phosphorylation profile analyses uncovered that the MAPK pathway is hyper-activated in liver cancer cell lines suggesting the its potential role for cancer cell proliferation. Furthermore, more than 84 phosphosites in the proteins encoded by cancer driver genes show dramatic difference in phosphorylation patterns between two types of cancer cells (QGY and Hep3B), especially many targeted sites of HIPK, a member of CDK kinase family. Finally, a network of selected differential phosphorylated proteins was constructed to present a potential positive regulatory pathway of cell proliferation in liver cancer cells.

## Results

### Different proliferative potential of three liver cell lines

**Figure 1:**
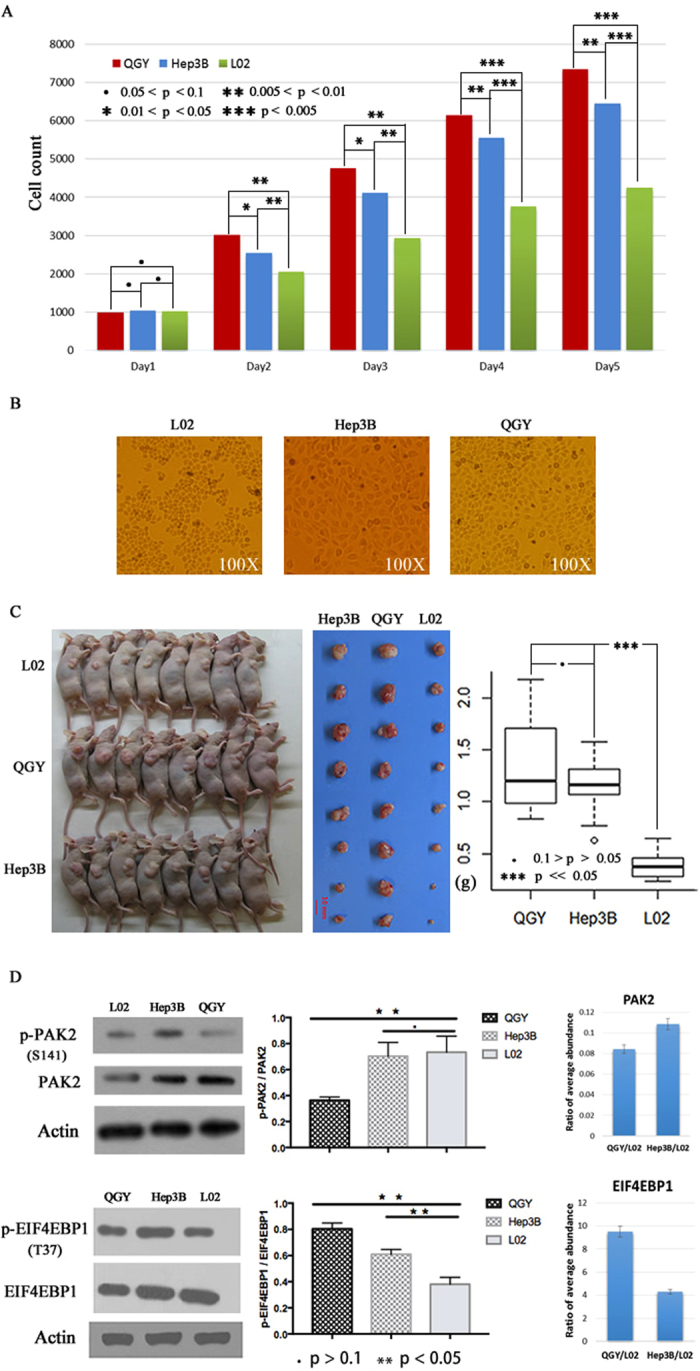
The distinct proliferation potential of three liver cell lines. (**A**) The proliferation of three liver cell lines under the same initial cell number and culture condition from day 1 to day 5. (**B**) The morphology of cultured three cell lines at day 5 under 100X microscope. (**C**) The results of proliferation assay using nude mice model. (**D**) The western blot results of phosphorylated sites T46 of EIF4IBP1 and S141 of PAK2, the middle graph is the statistical results of western blot based on densitometric analysis. The right bar charts compare the relative abundance of phosphorylated PAK2 and EIF4BP1 in QGY and Hep3B to L02 by MS/MS method.

Proliferative ability of cancer cells is one of key features to estimate malignant grades and invasive abilities of cancers and also directly correlates with the lifetime of patients^26, 27^. In this study, we firstly checked the proliferative abilities of three liver cancer cells (two liver cancer cell lines (Hep3B and QGY) and a fetal liver cell line (L02)) by *in vitro* and *in vivo* experiments. The results of cell proliferation assay suggested that QGY and Hep3B cells grown faster than L02 cells in conventional conditions of cell culture (Fig. 1A). Additionally, the morphological differences were observed in three cell lines after cultured for 5 days *in vitro*. Most of L02 cells displayed more irregular polygons shapes, smaller sizes and tighter arrangements than QGY and Hep3B cells (Fig. 1B). To compare the *in vivo* proliferative abilities of the three cell lines, 4 million cells were injected into the flank of each nude mouse for tumorigenesis. All tumors were harvested, weighed and evaluated after 30 days (Fig. 1C). Consistent with the results of previous *in vitro* experiment, two tumor cell lines (QGY and Hep3B) exhibited more rapid tumor growth than L02 cell suggesting higher *in vivo* proliferative potential of tumor cell lines. (Fig. 1C) The results suggested that QGY cells had significantly stronger proliferative ability *in vivo* than both Hep3B and L02. They were coincident with the findings *in vitro*. Then, we took two key proteins (PAK2 and EIF4EB1) responsible for cellular proliferation and cell cycle regulation as examples to investigate the correlation between their phosphorylation status and cellular proliferation potential in three cell lines by western blot using phosphorylation site-specific antibodies (Fig. 1D). Remarkably, the results shown that despite the protein abundance of PAK2 and EIF4BP1 are variant in these cell lines, the phosphorylation level at two phosphosites PAK2 (S141) and EIF4BP1 (T37) have negative and positive correlations with cellular proliferation potential respectively. For PAK2, a serine/threonine-protein kinase involving in motility^28^, cell cycle^29^, apoptosis and proliferation regulations, the enhanced autophosphorylation at the site S141 has been reported repressing cell growth^30^. EIF4EBP1 is a key negative regulator in gene translation by binding translation initiation factor EIF4E. Hyperphosphorylated EIF4EBP1 results in the release of EIF4E^31^ who activates PI3K/AKT, AMPK and mTOR signaling pathways to promote cell proliferation^32^. These data suggesting the possibility using these cell lines as models to investigate key phosphorylation events relevant to the high proliferation rate of liver cancer cells.

### Characterization of the phosphoproteome in three liver cell lines

**Figure 2:**
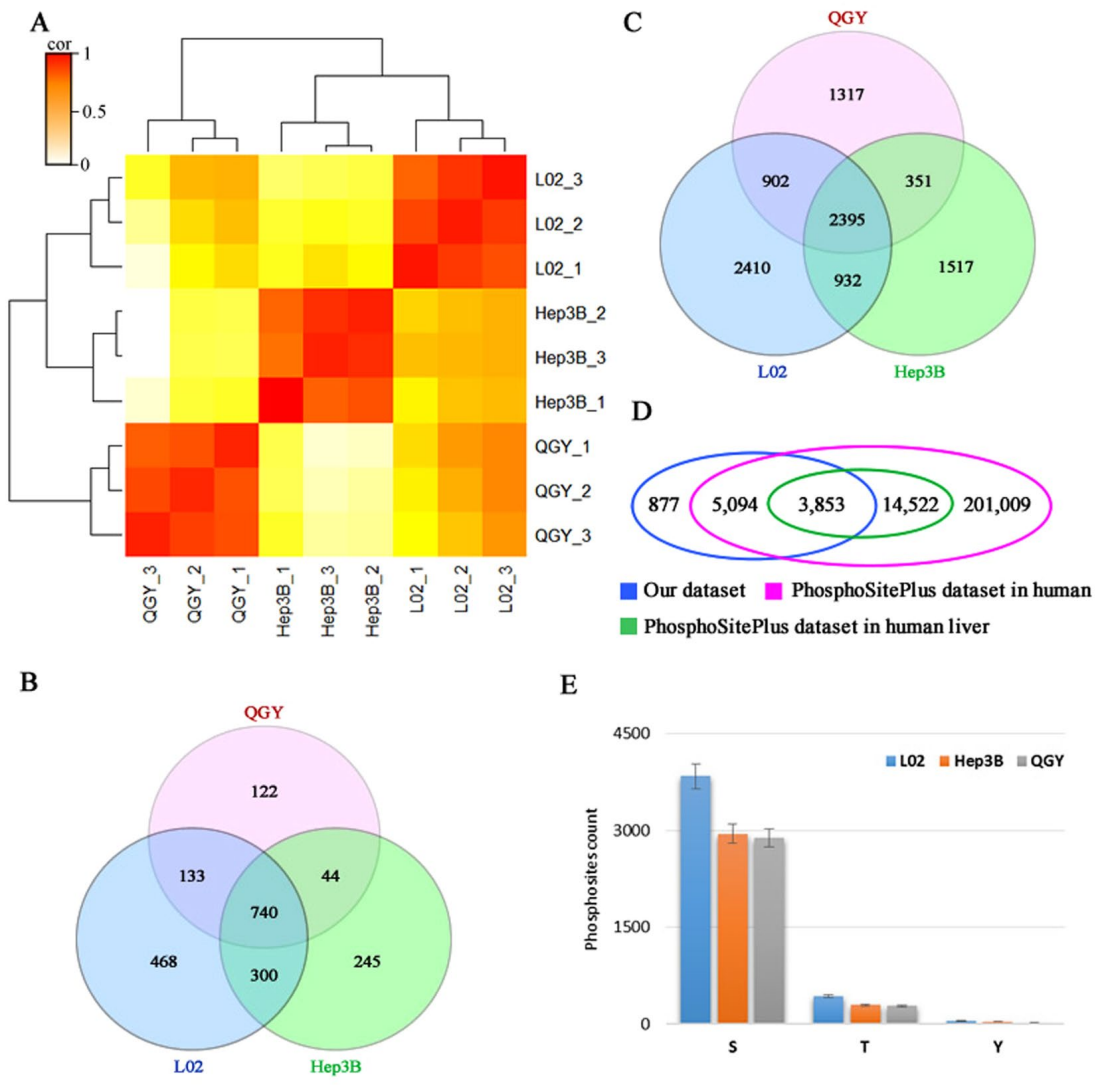
The landscape of phosphoproteomes of QGY, Hep3B and L02 cells. (**A**) The hierarchical cluster analysis of nine phosphorylation profiles showing the repeatability of MS/MS. (**B**) The overlap of identified phosphoproteins in QGY, Hep3B and L02 cell lines. (**C**) The overlap of detected phosphosites in QGY, Hep3B and L02 cell lines. (**D**) The overlap of phosphosites by comparison among our dataset, the datasets of human and human liver in PhosphoSitePlus database. E) The distribution of serine, threonine and tyrosine phosphosites detected in three liver cell lines.

To explore phosphorylation profiles of three liver cell lines, we performed nano-LC-MS/MS (Thermo Orbitrap Elite) analyses on TiO_2_-enriched phosphorylated peptides. The detailed procedures of these experiments can be found in Fig. S1. Phosphopeptides with high confidence (false discovery ratios (FDRs) lower than 0.05 and the PhosphoRS score of phosphosites over 70) were selected for further analyses. (Fig. S2). The relative abundance of phosphoproteins and phosphosites were estimated by normalized ion current of phosphopeptides. To evaluate the reproducibility of the experiments, we did three biological duplicates for each sample and calculated the pairwise correlations (Pearson correlation coefficients) of nine phosphorylation profiles. The results indicated that all of correlation coefficients between the two profiles from the same cell line were over 0.9, which are significantly higher than those from different cell lines (<0.6), and clustered together in hierarchical cluster analysis (Fig. 2A and Table S1). All of these results demonstrated the high reproducibility of our data and suggested the phosphorylation profiles of three cell lines are dramatically different from each other.

Totally, 2,057 phosphorylated proteins were quantified, in which 1,641, 1,329 and 1,039 phosphoproteins were found in L02, Hep3B and QGY respectively (Fig. 2B). And 9,824 unique phosphosites were identified with 6,639 in L02, 5,195 in Hep3B and 4,965 in QGY (Fig. 2C and Table S1), in which, 2,395 phosphosites in 740 proteins were shared by three cell lines (Fig. 2B and C). Intriguingly, more than 877 phosphosites from 482 unique proteins were first reported, which were not included in PhosphoSitePlus (version: 2015/11/12) a comprehensive resource of known phosphorylation sites^33^ (Fig. 2D and Table S2). Interestedly, the number of detected phosphorylated serine, threonine and tyrosine decreased in the order from L02 to Hep3B and to QGY (Fig. 2E) supporting a negative correlation between cell proliferative ability and global protein phosphorylation level in liver cancer cells.

### Differential phosphorylation of SR proteins affected RNA splicing

**Figure 3:**
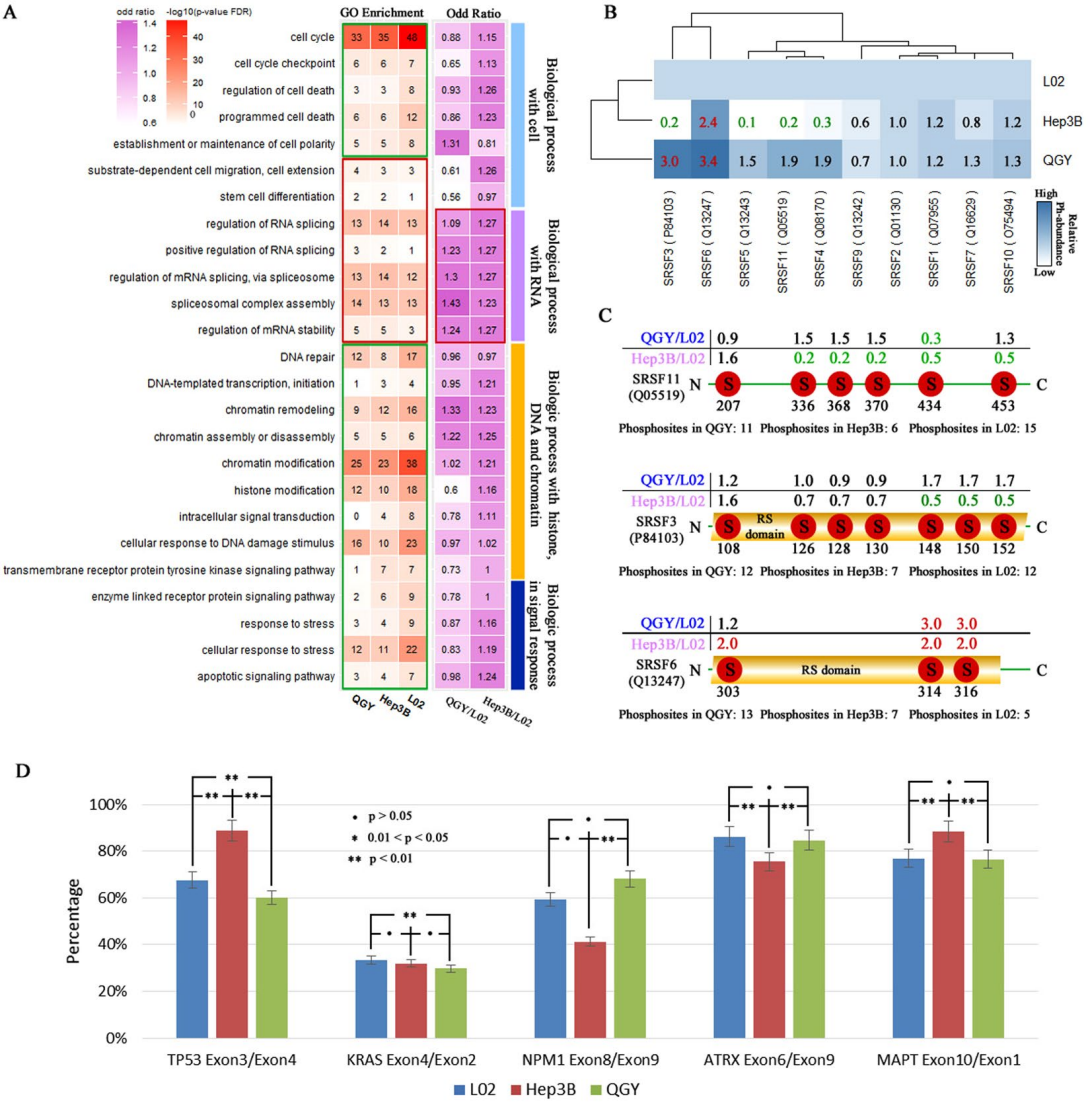
The function analyses of phosphoproteins. (**A**) Enrichment of phosphoproteins and their odd ratio in 25 GO terms from Biology Process category of GO in three liver cell lines. In the column of GO enrichment, the enrichment degrees were estimated by −log (FDR of *p value*); red pane contains GO terms which more enriched in QGY and Hep3B than in L02, but the green pane has the ones with reversed enrichment trend. The odd ratios of GO terms demonstrated the results of comparing phosphoprotein profiles in QGY and Hep3B to L02 cells by fisher’s exact test to detect enriched GO terms using accumulated abundance of phosphoproteins in/out of the GO term. The odd ratio above 1 suggested the GO term is enriched in QGY or Hep3B. The RNA splicing related GO terms were significant enriched in QGY or Hep3B (p-value < 0.05) and marked by red. (**B**) The hierarchical cluster analysis showed that the detected SR proteins and their abundance value were marked by red, green and black, respectively. If the relative abundance was between 2 and 0.5, it was marked by black, otherwise marked by red (>2 times than in L02) or green (<0.5 times than in L02). (**C**) The distribution of phosphorylation sites in SRSF3, SRSF6 and SRSF11. D) The special ratios of the isoforms of TP53, KRAS, NPM1, ATRX and MAPT acquired by QPCR in QGY, Hep3B and L02 cell lines.

To investigate the biological functions of phosphorylated proteins in the three liver cell lines, the enrichment analyses were performed in the biological process category of Gene Ontology (GO) for all 2057 phosphoproteins. As a result, 25 top enriched GO terms were selected and categorized into four classes (“Cell cycle and fate related processes”, “RNA related processes”, “Chromatin/DNA related processes” and “Signaling and response related processes”) based on their functional preferences (Fig. 3A). Comparing QGY, Hep3B with L02, phosphoproteins are more enriched in seven GO terms including the class of “RNA related processes” and two GO terms of “stem cell differentiation” and “cell migration”. Remained 18 GO terms showing higher enrichment in L02 than QGY and Hep3B were involved in certain key biological processes such as cell polarity, cell cycle, cell death, DNA repair, signal response and chromatin modification. We also calculated odd ratio for each GO term using Fisher’s exact test to compare accumulative abundance of annotated phosphoproteins in QGY and Hep3B to in L02. Most of the odd ratios of GO terms in the RNA related class were over 1.2 with significant p value (p < 0.01) (marked by red in the columns of odd rate). The results suggested that the phosphoproteins from liver cancer cell lines were enriched in the RNA splicing related processes and highlighted their potential roles in proliferation regulation of liver cancer cells.

We then further compared the abundance of phosphoproteins within the group of “RNA related processes” across all three cell lines (Table S3) and found that ten out of twelve members of serine/arginine-rich proteins family (SR) were higher phosphorylated in QGY cells comparing with others (Fig. 3B and C). The SR proteins are characterized by an RS domain and at least one RNA recognition motif (RRM) and essential for regulation of splice-site selection^34, 35^ Surprisedly, many phosphosites in SR proteins, such as the sites of S336, S368, S270 and S434 in SRSF11, the sites of S148, S150 and S152 in SRSF3 and the sites of S303, S314 and S316 in SRSF6, are located in the repeats range of arginine and serine or RS domains which were previously thought important for mRNA transportation and alternative splicing (Fig. 3C)^34, 36^. More important, most of these sites are highly phosphorylated in QGY comparing with other cells. And the phosphorylation patterns of these phosphosites in Hsep3B and L02 cell are distinct from each other. These results suggested an aberrant RNA alternative-splicing pathway and abnormal RNA splicing events of downstream in highly proliferative liver cell lines.

It has been reported that the expression of exon 10 of MAPT/Tau is regulated by phosphorylation at the site S303 of SRSF6 through alternative-RNA splicing^37^. Therefore, we used it as reporter to evaluate the consequences of different phosphorylation states of SR proteins in three cell lines by measuring the ratio of mRNAs from exon 10 (variable exon) to exon 1 (constant exons) by QPCR assays. The result suggested that the isoform ratio of MAPT/Tau was significant difference in the three kinds of liver cells (Fig. 3D). The similar method was used to compare the ratio of mRNA isoforms of four known cancer driver genes (TP53, KRAS, ATRX and NPM1). Consistently, significant variations were also observed in the four genes among three liver cell lines (Fig. 3D). Although the biological significance of these splicing changes are not clear, these results combined with data for SRRM2 gene (see below) support the hypothesis that the aberrant RNA splicing pathway promoters cell proliferation^34^ in liver cancer cells.

### Phosphorylaton dynamics of protein complexes

**Figure 4:**
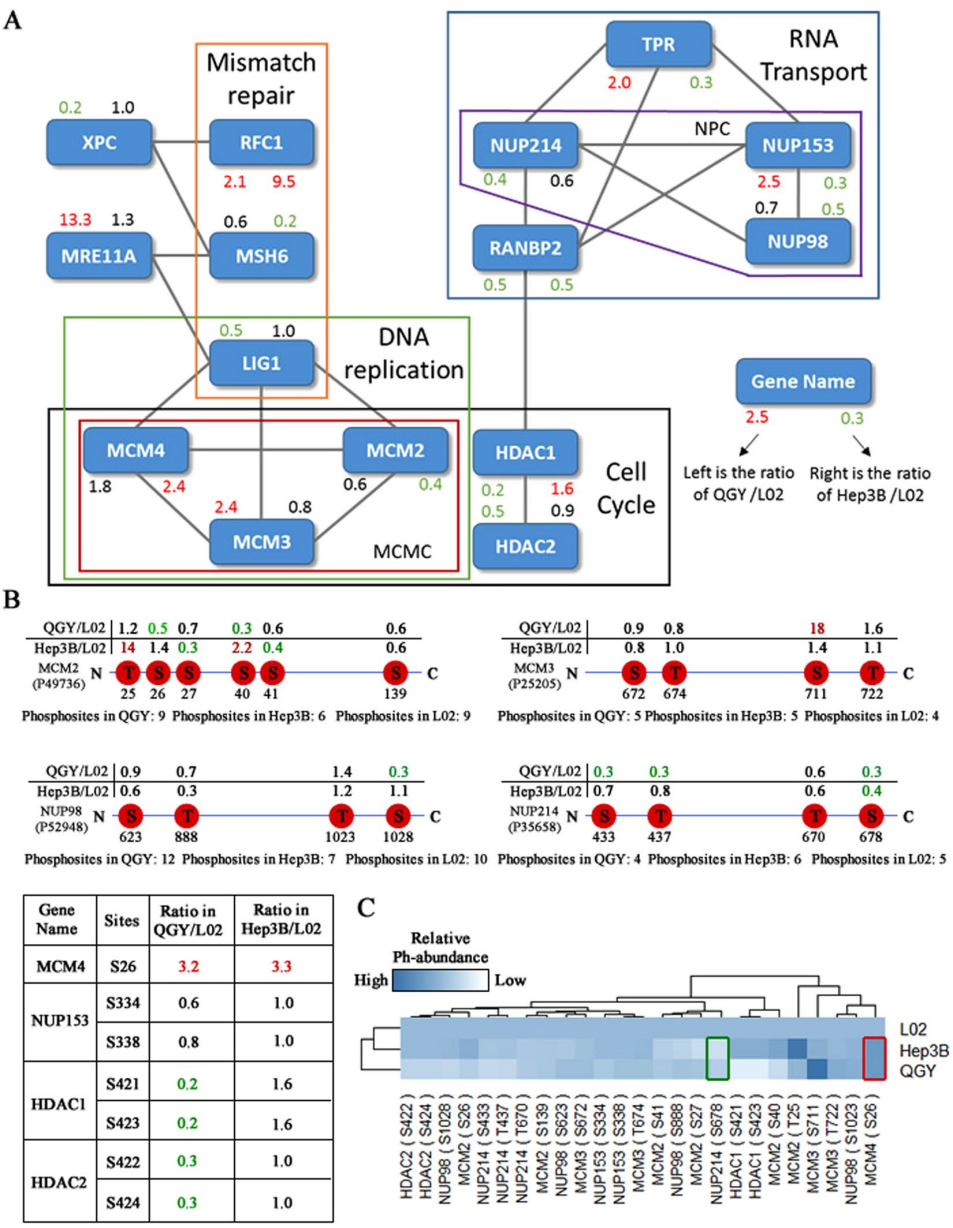
Phosphoproteins in the cell cycle pathway. (**A**) The PPI network of phosphoproteins in the relative processes of cell cycle in KEGG. The red, green and black numbers represent the ratios of phosphoproteins abundance in QGY and Hep3B to L02 respectively. The up-regulated phosphoproteins marked by red; down-regulated marked by green and others marked by black. (**B**) The abundance distribution of phosphorylation sites in MCM2, MCM3, MCM4, NUP98, NUP214, NUP153, HDAC1 and HDAC2 in which MCM4, NUP153, HDAC1 and HDAC2 were listed in the bottom left table of the figure. (**C**) The hierarchical cluster analysis of shared phosphosites from those proteins of relative processes of cell cycle. The red and green pane respectively marked the hyperphosphorylated and hypophosporylated sites both in QGY and Hep3B.

To investigate the functional connections among these phosphorylated proteins of involving in the KEGG signal pathways of cell cycle, regulation of cell cycle, cell proliferation and quantified in the three liver cell lines (Table S4), we searched them against String database (PPI confident >0.7) to construct a protein-protein network. The networks illustrates that some complexes essential for cell cycle, DNA repair and RNA transportation pathways are hotspots of phosphorylation and the phosphorylation profiles of MCM and NPC complex have distinct in three cell lines (Fig. 4A). For example, RFC1, a large subunit of replication factor C, is essential in mismatch repair and replication and interact with DNase gamma at 3′-OH/5′-P ends in telomere^38^. According to the immunohistochemical results in HIS database (http://www.proteinatlas.org), RFC1 is up-regulated in liver cancer. Our data also show that it was hyperphosphorylated in Hep3B and QGY cells. Secondly, MRE11A, the key component of MRN complex, is involved in double-strand break (DSB) repair and plays a central role in the activities of single-strand endonuclease and double-strand-specific 3′-5′ exonuclease^39–41^. It was found that phosphorylated MRE11A has higher abundance in QGY cells. Thirdly, the phosphorylation levels of some phosphosites in mini-chromosome maintenance complex (MCM) proteins are dramatic different in different cell lines (Fig. 4B). For example, the abundance of a novel phosphosite T27 in MCM2 in Hep3B was 14 folds higher than that of L02. And phosphorylated S711 in MCM3 shows 18 folds higher than that of L02. Additionally, the core numbers of nuclear pore complex (NPC) including NUP214, NUP98 and NUP153 were hypo-phosphorylated in QGY and Hep3B comparing with L02 cells (Fig. 4B), Phosphorylation of NUP98 is a crucial step to NPC disassembly and is also a rate-limiting step in cell mitosis^42^.

It is also notable that the phosphosites of S421 and S423 in HDAC1, S422 and S424 in HDAC2 were unambiguously identified and their relative phosphorylation abundance was significantly down-regulated in QGY and L02 (Fig. 4B). Additionally, the hierarchical cluster analysis of the phosphorylation quantification of all shared phosphorylated sites on MCM, NPC and HDACs supported that the phosphorylation profiles in Hep3B were more close to that in L02 than in QGY (Fig. 4C). These results suggested a synergic relationship among the pathways of cell cycle, DNA repair, DNA replication and RNA transport, and they could be regulated by phosphorylation in liver cancer cells.

### Phosphorylation profiles suggested activated MAPK pathway in liver cancer cells

**Figure 5:**
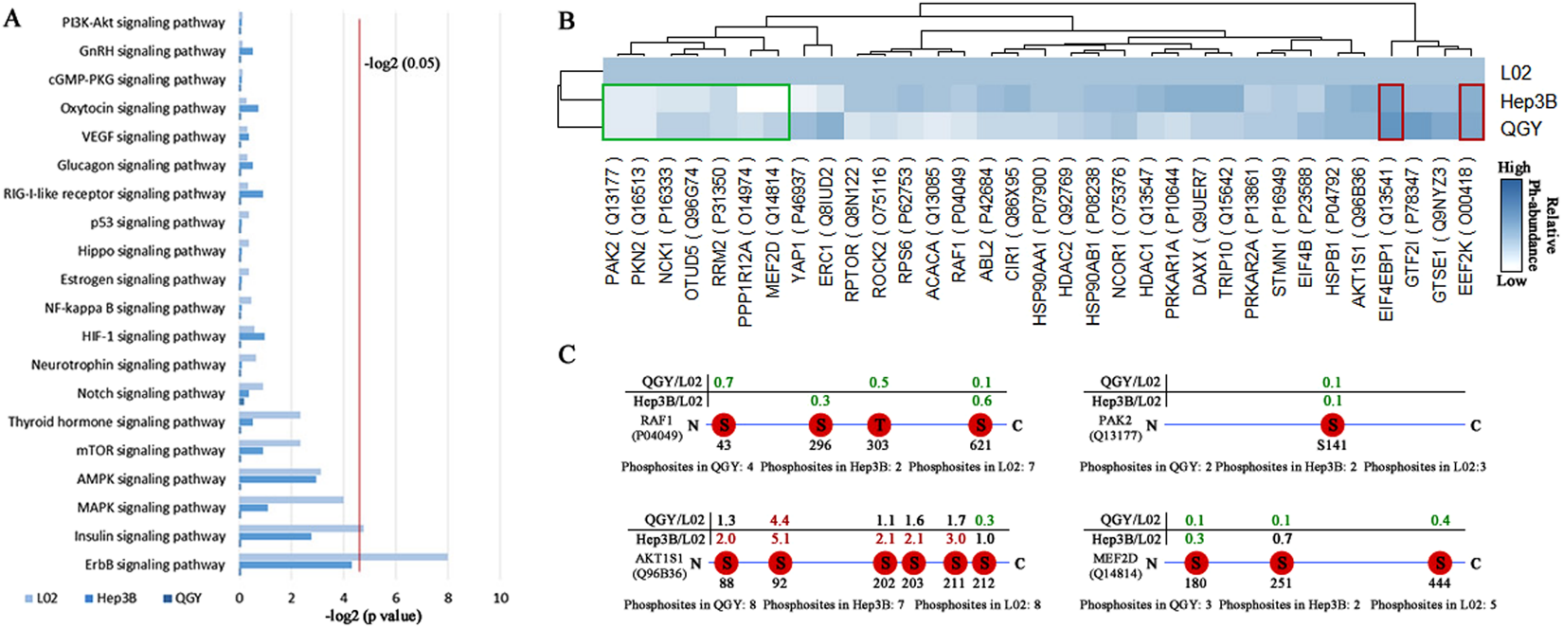
Phosphorylation profiles of signal transduction pathways. (**A**) The KEGG enrichment analysis showed the top 20 signaling pathways in QGY, Hep3B and L02, respectively. (**B**) The hierarchical cluster analysis of phosphoproteins abundance in the top 20 signal pathways crossed the three cell lines. The red and green pane respectively marked the up-regulated and down-regulated phosphoproteins both in QGY and Hep3B. (**C**) The abundance distribution of phosphorylation sties in RAF1, PAK2, AKTIS1 and MEF2D.

It is well established that phosphorylation plays a crucial role in regulating cellular signal transduction. We mapped phosphoproteins shared by three cell lines to KEGG pathways and did enrichment analyses. The top 20 enriched signal pathways include ErbB, AMPK, MAPK, Insulin, Notch, p53, VEGF and PI3K-Akt pathways. In which, the MAPK/ERK pathway has long been known regulated by phosphorylation mechanisms and function as a tumor suppressor as well as more common pro-oncogenic signal, which regulate cell cycle, development, apoptosis and proliferation progresses^43–45^ (Fig. 5A,B and Table S5). The phosphorylation profiles of several key components in MAKP pathway suggested it is activated in high proliferative cancer cell lines. For example, the phosphorylation of S43 and S296 in RAF1, which represses cellular signal transduction between Ras GTPase and the MAPK/ERK cascade, was at lower level in QGY and Hep3B than L02 lines (S43,QGY/L02: 0.7; S296, Hep3B/L02: 0.3). And, the autophosphorylation of S141 in PAK2 (Q13177) has been reported to repress cell growth^30^ and is nearly ten-times less in QGY and Hep3B than in L02 in our data. Furthermore, MEF2D is an essential subtract of MAPK and the phosphorylation at S444 of MEF2D could repress its transcriptional activity^46^. The relative lower phosphorylation level of S444 in QGY and Hep3B than L02 suggesting MEF2D is activated in cancer cell lines. Additionally, AKT1S1 is a key component of mTOR pathway at downstream of MAPK pathway and its phosphorylation plays a critical role in inducing cell growth^47^. We found most of phosphosites in AKT1S1 are hyperphosphoylated in QGY and Hep3B compared to that in L02 (Fig. 5C). Taken together, our data suggested that MAPK pathway is activated by site-specific phosphorylation in high proliferation liver cancer cell.

### Aberrant phosphorylation of proteins encoded by cancer driver genes

**Figure 6:**
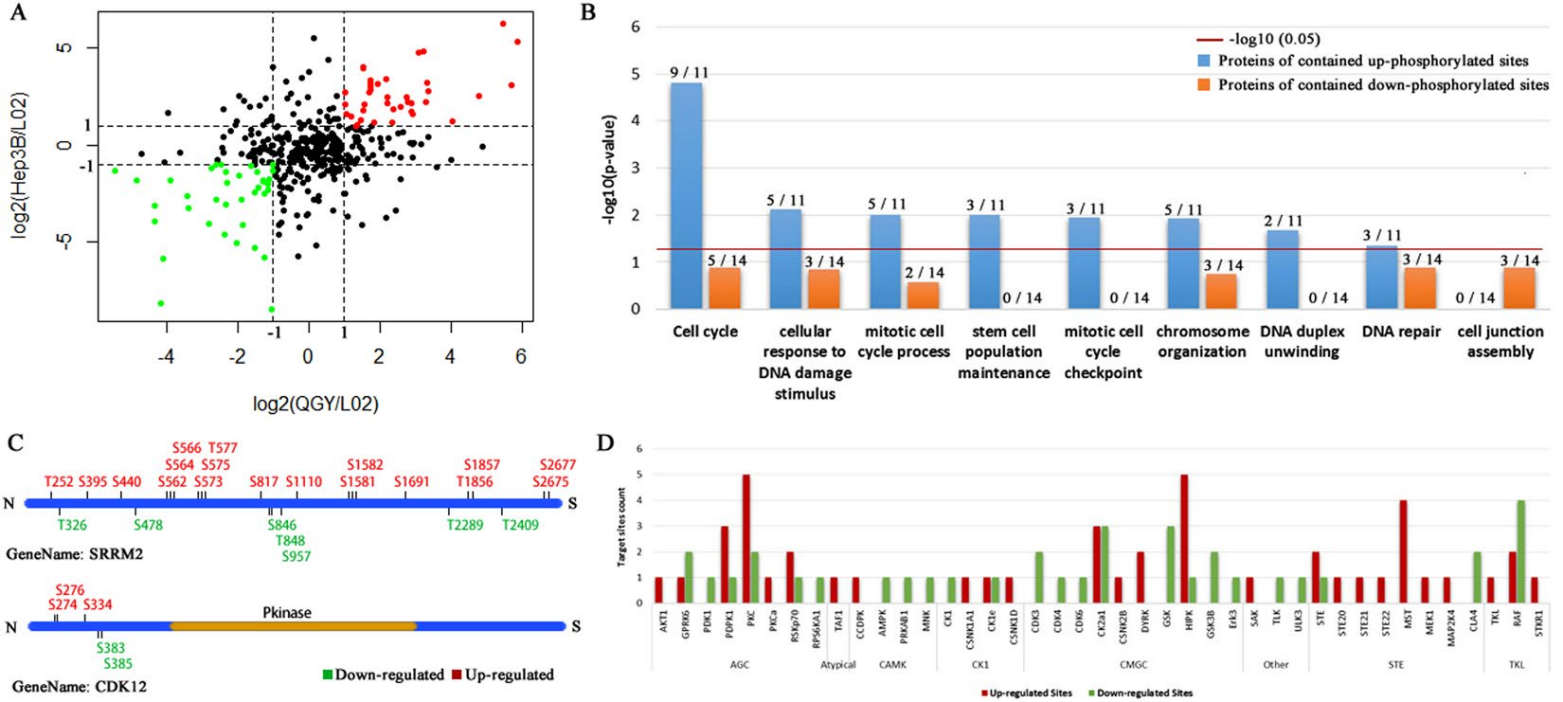
Function analysis of different phosphosites in cancer driver genes. (**A**) Scatter plot of the ratios of phosphorylated sites’ abundance in QGY and Hep3B to L02. The spots marked as green and red have the relative lower (log2 (relative abundance) <-1) and higher (log2(relative abundance) >1) abundances in QGY and Hep3B. (**B**) The comparison of enriched GO biological processes between the phosphoproteins containing hyper-phosphoryrlated and hypo-phosphoryrlated phosphosites. The labels on the columns show the numbers of phosphoproteins in on certain categories (left) vs that in total (right). (**C**) The distribution of down-regulated (green) and up-regulated (red) phosphosites in SRRM2 and CDK12. (**D**) The number of hyper-phosphoryrlated and hypo-phosphoryrlated sites targeted by different kinases/families in Hep3B and QGY.

The aberrant phosphorylation of cancer driver genes/proteins has long been known and it help cancer cells to obtain selective growth advantage *in vivo* ^48^. Although many cancer driver genes have been reported, the phosphorylation profiles of the proteins of encode by them are largely unknown. Here, we mapped identified phosphorylated sites back to 435 cancer driver genes (David Tamborero *et al*.^49^) and found 457 phosphorylated sites in 162 cancer driver proteins. In which, 70 proteins were quantified in all three cell lines (Fig. 6A and Table S6). We selected 31 phosphosites which were hyperphosphorylated and hypophosporylated in both QGY and Hep3B vs L02 cells, named as Hyper-phoSites and Hypo-phoSites (Table S7). Interestedly, that the 12 Hyper-phoSites containing proteins were significantly enriched in the GO terms of cell cycle, cellular response to DNA damage stimulus, chromosome organization and stem cell population maintenance, but no significant enriched GO terms were found for 15 Hypo-phoSites proteins (p-value < 0.05) (Fig. 6B). In the group of Hyper-phoSites, ATRX, MDC1, RIF1, APC and PCM1^50–54^ played a role in cell cycle by phosphorylation. ATRX is a regulator of gene transcription to facilitate DNA replication and the phosphorylation of serine residues is a signal to promote the progression of mitosis^50^. MDC1 is an essential protein in response to DNA damage and mediated both the S phase and G2/M phases of the cell cycle^54^, which relies on the phosphorylation of serine and tyrosine in a specific region (residues 200–420) of MDC1. In our dataset, S299 and T301 of MDC1 were hyperphosphorylated in QGY and Hep3B, supporting that DNA repair pathway was abnormal constitutively activated in QGY and Hep3B. We also found that two active sites (S1859 in CAD and S2125 in FLAN) were hypophosphoylated in Hep3B and QGY cells. It is known that the hyperphospholation of the two sites could promote cancer cellular proliferation. The phospholation at S1859 in CAD persistently stimulated de novo synthesis of pyrimidines in mammalian cells^55^. And highly phosphorylated S2152 improves the development ability of cancer and is considered as molecular target of cancer treatment^56^. In the other four proteins containing up- and down-regulated phosphorylation sites, SRRM2 and CDK12 were related to RNA splicing and their phosphorylation profiles were shown in Fig. 6C. Intriguingly, more than 332 phosphorylated sites from SRRM2, a pre-RNA splicing factor, were detected, 168 sites were observed in all three liver cell lines and over 13% of sites (25 out of 168) in QGY and Hep3B cells were different from that in L02 (Fig. 6C). SRRM2 is a key component of spliceosome and is involved in the first catalytic step of splicing. The distinct phosphoprylation profiles of SRRM2 may indicate its different catalytic preference and the way interacting with other SR proteins in three cell lines^57–59^ To predict upstream kinases targeting 31 differential phosphoproteins form cancer driver genes (Table S7), we used Kinase-specific Phosphorylation Site Prediction (GPS) service and found 41 high-confident kinases candidates. The statistical results demonstrated that STE kinase family and the kinases of MST, HIPK and PKC target most of Hyper-phoSites whereas substrates of CDK subfamily (CDK3/4/6) were enriched in Hypo-phoSites. Their targeting genes such as TP53BP1, NUP214 and CEP170 were highly related to cell cycle process. Previous studies demonstrated that the kinases of HIPK, PKC and CDK subfamily, and their target genes were important in cell growth and proliferation^5, 60–62^ (Fig. 6D).

## Discussion

In this study, we applied an unbiased and global phosphoproteomic approach to disclose the protein phosphorylation profiles in three liver cell lines with different proliferative potential. Thousands of phosphorylated sites and proteins were identified/quantified and many of them were firstly reported in liver even human samples, which generated a deep and expansive phosphoproteomic view in cancer biology. Comparing these phosphorylation profiles, we discovered many phosphorylated protein complexes and pathways that promote aberrant proliferation of cancer cells.

Although constitutive phosphorylation of kinases or their substrates of have been thought as a key feature of various cancer cells^63, 64^, our results suggested that the global phosphorylation level of liver cancer cell lines (Hep3B and QGY) are even lower than that of non-cancer cell line (L02). That’s partially because many phosphosites who have opposite, antagonistic functions to the activate sites were hypophosphorylated in cancer cells, for example S141 in PAK2 and S43 and S296 in RAF1 in the MAPK pathway. These results implied that phosphatases that remove phosphate moieties from these sites may also have significant effects on cancer development.

Our finding, that the aberrant phosphorylation of SR proteins results in dramatically differential isoform expression, highlighted the role of RNA alternative splicing in cell proliferation regulation. SR proteins are key components of spliceosome and their overexpression have been reported conferring cancerous phenotypes, such as cell cycle progression and anti-apoptosis^65–68^, anchorage-independent cell proliferation^65^, tumor formation in nude mice^65^. They usually have a RS domain, which contains a eight arginine and serine repeats known essential for various biological processes such as mRNA export, genome stabilization, nonsense-mediated decay and translation^34, 69^. We found the many arginines and serines in the region are highly phosphorylated in QGY comparing with other cells, which known keeping SR proteins in nucleus but not repressing its translocation to cytoplasm^70^. Although based on our data, it is clear that this transition changes mRNA splicing of many key genes such as ATRX, KRAS, TP53 *et al*., how it affects other cellular progresses is remained for further investigation.

It is well known that the MAPK pathway is deregulated in approximately one-third of all human cancers^71^. Historically, activation of ERK signaling, downstream part of MAPK pathway was synonymous with cell proliferation. Finding therapeutic targets in MAPK pathway is a hot topic in cancer research. Our study revealed the aberrant phosphorylation patterns of two kinases RAF1, PAK2 and one downstream transcription factor MEF2D in the pathway. These results not only consistently suggested that MAPK pathway is activated in liver cancer cells but also provide new potential therapeutic targeting sites for drug development. Moreover, our phosphorylation patterns analyses of several protein complex (MCM and NPC), epigenetic enzymes (HDAC1 and HDAC2.) and cancer driver genes (ATRX, MDC1, RIF1, APC and PCM1) showed a phosphorylation related signal transition network affecting cell proliferation ability (Fig. S3) and revealed the extreme complex nature of phosphoproteome transformation in liver cancer cells. Therefore, combining phosphoproteome data with other genomic and proteomic screening data will be very useful to deep our understanding of mechanisms of underline uncontrolled proliferations in liver cancer cells in future.

In summary, we discussed the potential correlation between proliferation ability and protein phosphorylation by demonstrating the variations of phosphorylation profiles of proteins in the biology process such as cell cycle, RNA splicing, signal transport and known cancer driver genes. Our study also identified many novel phosphorylated proteins and phosphosites in live cells, which not only extended our knowledge of phosphorylation signaling in liver cancer but also provided a useful resource for exploring the potential regulatory mechanisms on proliferation and survival in liver cancer cell.

## Methods

### Cells culture and counting

The liver cancer Hep3B cell line was purchased from American Type Culture Collection (ATCC). Both fetal liver HL-7702 (L02) cell line and liver cancer QGY-7703 (QGY) cell line were preserved in our laboratory. L02 cell is an immortalized non-tumor cell line derived from fetal liver tissue and was widely used as an *in vitro* model of nonmalignant liver^72^. Comparing with other cancer cell lines, it is highly differentiated and growth in a relative lower rate based on our data. They were cultured in high-glucose DMEM medium (Gibco) supplemented with 10% (vol/vol) fetal bovine serum (FBS, Hyclone), and incubated in a humidified incubator (Heraeus) at 37 °C with 5% CO_2_. For each cell line, 10^6^ cells were resuscitated at day 0, the medium was replaced every day and the cells were eventually digested by trypsin and harvested for mass spectrometry (nearly 10^7^ cells).

The cell counting assays were performed in the same culture condition using Celigo Image Cytometer (Nexcelom 200-BFFL-S) and continued culture in 24-well plates from day 0 to day 5. The initial cells used were 1000 cells per well and each type of cells was cultured in three independent wells. Cell counting was auto-completed using Direct Cell Counting. In day 5, cell morphology was imaged by inverted microscope in 100X (Olympus IX51).

### Proliferation assays in nude mice

All animal experiments were carried out in accordance with the approved guidelines and protocols approved by the Animal Experimental Ethics Committee of Fudan University. Briefly, 4–6-week-old BALB/c nude mice were obtained from Institute of Materia Medical (CAS, Shanghai, China) and maintained in our institutional pathogen-free mouse facilities. Cells from each liver cell line were cultured and digested by trypsin. Cells were collected, washed twice with PBS buffer, and suspended in PBS buffer with the cell density of 2 × 10^7^/ml. To grow tumors, 200 μl of cell suspension (4 million cells) was implanted into the flank of each nude mouse. After 30 days, nude mice were executed at the same time, with tumors excised and weighed. The 4 million is the minimum number of cells required to generate tumor stably in nude mice. When the injection number is less than 4 million, the L02 cell vaccination group can not guarantee the formation of tumor.

### Western blot assay

Protein samples were subjected to SDS-PAGE with appropriate concentrations, and then electro-transferred to nitrocellulose membranes (Millipore, MA). The membranes were blocked with 5% skimmed milk, washed and then incubated with primary antibodies overnight at 4 °C, followed by incubation with peroxidase-conjugated secondary antibody. Antibodies used were anti-actin (1:2000, Sigma), anti-EIF4EBP1 (1:500, Abnova), anti-phospho-EIF4EBP1 (T37) (1:500, Abnova), anti-PAK2 (1:500, Abcam) and anti-phospho-PAK2 (S141) (1:500, Abcam). Images were taken and estimated by densitometric analysis with Quantity One (Bio-Rad) and normalization with actin. Statistical analysis was performed with Student’s t-test and a threshold of p < 0.05 was defined as statistical significance cutoff.

### Protein extraction and digestion

The proteins from three liver cells (L02, Hep3B and QGY) were extracted respectively by dissolving in pre-cooled protein lysis solution (8 M Urea). The supernatants were collected after centrifugation at 20,000 g for 15 min at 4 °C. Protein quantification was performed using Bradford method. For each sample, 300 µg proteins was digested via trypsin (Promega, Madison, WI, USA) in 25 mM NH_4_HCO_3_ at 37 °C, and preserved overnight after reductive alkylation. The ratio of enzyme to total protein is 1:70. The digested peptides were equally sub-packaged into three EP tubes (~100 µg peptides) for following phosphopeptide, enrichment experiment.

### Phosphopeptide Enrichment

A self-packed TiO_2_ column assembled in a gel-loading tip (Eppendorf GELoader Tips: 20 ml) was used to capture phosphorylated peptides as described by Larsen MR *et al*. with some modifications^73^. Briefly, a C8 Eempore disk (3 M) was fitted into a gel-loading tip as a plug and then the turbid liquid was injected (TiO2 material (10 mg) and acetonitrile (100 ul ACN)) was injected and pressed into the gel-loading tip. TiO_2_ material was retained on the C8 plug. The mixed liquid (100 µl) was injected and pressed into the self-packed enrichment column containing digested peptides (100 µg) and loading buffer (1 M glycolic acid (Sigma), 5% trifluoroacetic acid (TFA, Sigma) and 80% ACN). To remove non-phosphopeptides, loading buffer, washing buffer (5% TFA and 80% ACN) and UHQ water were pressed through the enrichment column in order. Finally, the enriched phosphopeptides were collected and dried after elution by 2 M ammonium hydroxide and 30% ACN. The dried phosphopeptides were re-dissolved in 0.1% formic acid (FA) for nano-LC-MS/MS analysis (The parameters of MS/MS experiments and database searching can be found in the supplementary file).

### Estimation of relative abundance for phosphorylated sites, peptides and proteins

To obtain reliable results, all samples were treated in the same condition and the data analyses were performed by Proteome Discoverer (PD version 1.4; Thermo Fisher) and Seq-HP (supplementary method) with uniform parameters. And the process was repeated three times for each sample. The phosphopeptides with high confidence were extracted based on the confidence of phosphopeptides and their phosphosites while FDR were lower than 0.05 and PhosphoRS scores of phosphosites were more than 70 (Fig. S2). The abundance information of phosphopeptides were extracted from PD into excel files. To estimate phosphoprotein abundance, firstly, normalizing the ion current intensity of phosphopeptides was performed in each run by dividing the ion current intensity of phosphopeptides into the summation of ion current intensity in the LC-MS/MS. The results were considered as phosphopeptides’ relative abundance. Secondly, relative abundance of phosphoproteins was also calculated by accumulating the relative abundance of phosphopeptides within a protein in each run. For phosphopeptides shared by several phosphoproteins, their ion current was split equally into those proteins. Finally, the relative abundance of phosphoproteins in a live cell line was represented via calculating the relative abundance median in three biological repeats for following analysis. Furthermore, the abundance of phosphosites was also estimated according to the normalized relative abundance of phosphorylated peptides via calculating median in all technological repeats per sample. Additionally, those phosphoproteins undetected in three technological repeats were filtered out. The cut-off values of different phosphoproteins were appointed with that the relative abundance was more than 2 or lower than 0.5 times compared to L02.

### QPCR assays

Total RNAs were extracted from the fresh cells using Trizol (Invitrogen), followed by further purification with RNEasy columns (Qiagen). RNAs were reversely transcribed to cDNAs using the Superscript RT kit (TOYOBO, Japan) according to the manufacturer’s instructions. qRT-PCR analysis was conducted using the SYBR Green Supermix kit (Toyobo, Osaka, Japan) in combination with QuantStudio™ 7 (Life technologies, USA). The cycle parameters were one hot starting cycle at 95 °C for a 1-min, following with 40 cycles of 95 °C for 10 s, 60 °C for 10 s and 72 °C for 30 s. Gene expression levels were normalized by housekeeping gene β2MG, with the paired primers (Forward: 5′-ATGAGTATGCCTGCCGTGTGAAC-3′, Reverse: 5′-TGTGGAGCAACCTGCT CAGATAC-3′). The paired primers of TP53, KRAS, NPM1, ATRX and MAPT were listed in Table S8.

### Computation of odd ratio

To estimate the odd ratio of phosphorylation abundance in GO term, a two-way contingency table was created based on the accumulated abundance of phosphoproteins in/out of a GO term in comparing QGY and Hep3B to L02, and calculated by Fisher’s exact test using R package. The p-value of 0.05 or less was significant.

## Electronic supplementary material

Supplementary Information

## References

[1] Torre LA (2015). Global cancer statistics, 2012. CA: a cancer journal for clinicians.

[2] Siegel RL, Miller KD, Jemal A (2017). Cancer Statistics, 2017. CA Cancer J Clin.

[3] Chen W (2016). Cancer statistics in China, 2015. CA Cancer J Clin.

[4] Davis GL (2008). Hepatocellular carcinoma: management of an increasingly common problem. Proceedings.

[5] Rey C (2013). HIPK1 drives p53 activation to limit colorectal cancer cell growth. Cell cycle.

[6] Hsieh SL (2015). Sedanolide induces autophagy through the PI3K, p53 and NF-kappaB signaling pathways in human liver cancer cells. International journal of oncology.

[7] Kim JY (2016). Phosphoproteomics reveals MAPK inhibitors enhance MET- and EGFR-driven AKT signaling in KRAS-mutant lung cancer. Molecular cancer research: MCR.

[8] Francavilla C (2017). Phosphoproteomics of Primary Cells Reveals Druggable Kinase Signatures in Ovarian Cancer. Cell reports.

[9] Ho TH (2017). Differential gene expression profiling of matched primary renal cell carcinoma and metastases reveals upregulation of extracellular matrix genes. Annals of oncology: official journal of the European Society for Medical Oncology.

[10] Uchida S (2016). Systemic delivery of messenger RNA for the treatment of pancreatic cancer using polyplex nanomicelles with a cholesterol moiety. Biomaterials.

[11] Radivojac P (2008). Gain and loss of phosphorylation sites in human cancer. Bioinformatics.

[12] Reimand J, Wagih O, Bader GD (2013). The mutational landscape of phosphorylation signaling in cancer. Scientific reports.

[13] Hama T (2009). Prognostic significance of epidermal growth factor receptor phosphorylation and mutation in head and neck squamous cell carcinoma. Oncologist.

[14] Corless CL, Fletcher JA, Heinrich MC (2004). Biology of gastrointestinal stromal tumors. Journal of clinical oncology: official journal of the American Society of Clinical Oncology.

[15] Sharma SV, Bell DW, Settleman J, Haber DA (2007). Epidermal growth factor receptor mutations in lung cancer. Nature reviews. Cancer.

[16] Harsha HC (2008). Activated epidermal growth factor receptor as a novel target in pancreatic cancer therapy. Journal of proteome research.

[17] Hynes NE, MacDonald G (2009). ErbB receptors and signaling pathways in cancer. Current opinion in cell biology.

[18] Reimand J, Bader GD (2013). Systematic analysis of somatic mutations in phosphorylation signaling predicts novel cancer drivers. Molecular systems biology.

[19] He Q (2014). Genome-wide prediction of cancer driver genes based on SNP and cancer SNV data. American journal of cancer research.

[20] Forbes, S. A. *et al*. The Catalogue of Somatic Mutations in Cancer (COSMIC). *Current protocols in human genetics* Chapter 10, Unit 10 11, doi:10.1002/0471142905.hg1011s57 (2008). 10.1002/0471142905.hg1011s57 PMC2705836 18428421

[21] Sidoli S, Fujiwara R, Kulej K, Garcia BA (2016). Differential quantification of isobaric phosphopeptides using data-independent acquisition mass spectrometry. Mol Biosyst.

[22] Schmidlin T (2016). Assessment of SRM, MRM(3), and DIA for the targeted analysis of phosphorylation dynamics in non-small cell lung cancer. Proteomics.

[23] Marmiroli S, Fabbro D, Miyata Y, Pierobon M, Ruzzene M (2015). Phosphorylation, Signaling, and Cancer: Targets and Targeting. BioMed research international.

[24] Lopez Villar E, Madero L, J AL-P, W CC (2015). Study of phosphorylation events for cancer diagnoses and treatment. Clinical and translational medicine.

[25] Cohen P (2001). The role of protein phosphorylation in human health and disease. The Sir Hans Krebs Medal Lecture. European journal of biochemistry.

[26] Gleason, D. F., Mellinger, G. T. & Veterans Administration Cooperative Urological Research, G. Prediction of prognosis for prostatic adenocarcinoma by combined histological grading and clinical staging. 1974. *The Journal of urology* **167**, 953-958, discussion 959 (2002). 11905924

[27] Tumor Grade. National Cancer Institute (2014).

[28] Hsu RM, Tsai MH, Hsieh YJ, Lyu PC, Yu JS (2010). Identification of MYO18A as a novel interacting partner of the PAK2/betaPIX/GIT1 complex and its potential function in modulating epithelial cell migration. Molecular biology of the cell.

[29] Orton KC (2004). Phosphorylation of Mnk1 by caspase-activated Pak2/gamma-PAK inhibits phosphorylation and interaction of eIF4G with Mnk. The Journal of biological chemistry.

[30] Jung JH, Traugh JA (2005). Regulation of the interaction of Pak2 with Cdc42 via autophosphorylation of serine 141. The Journal of biological chemistry.

[31] Pause A (1994). Insulin-dependent stimulation of protein synthesis by phosphorylation of a regulator of 5′-cap function. Nature.

[32] Yanagiya A (2012). Translational homeostasis via the mRNA cap-binding protein, eIF4E. Molecular cell.

[33] Hornbeck PV (2015). PhosphoSitePlus, 2014: mutations, PTMs and recalibrations. Nucleic acids research.

[34] Long JC, Caceres JF (2009). The SR protein family of splicing factors: master regulators of gene expression. The Biochemical journal.

[35] Manley JL, Tacke R (1996). SR proteins and splicing control. Genes & development.

[36] Anko ML (2012). The RNA-binding landscapes of two SR proteins reveal unique functions and binding to diverse RNA classes. Genome Biol.

[37] Yin X (2012). Dual-specificity tyrosine phosphorylation-regulated kinase 1A (Dyrk1A) modulates serine/arginine-rich protein 55 (SRp55)-promoted Tau exon 10 inclusion. The Journal of biological chemistry.

[38] Uchiumi F, Ohta T, Tanuma S (1996). Replication factor C recognizes 5′-phosphate ends of telomeres. Biochemical and biophysical research communications.

[39] Kijas AW (2015). ATM-dependent phosphorylation of MRE11 controls extent of resection during homology directed repair by signalling through Exonuclease 1. Nucleic acids research.

[40] Carney JP (1998). The hMre11/hRad50 protein complex and Nijmegen breakage syndrome: linkage of double-strand break repair to the cellular DNA damage response. Cell.

[41] Paull TT, Gellert M (1998). The 3′ to 5′ exonuclease activity of Mre 11 facilitates repair of DNA double-strand breaks. Molecular cell.

[42] Glavy JS (2007). Cell-cycle-dependent phosphorylation of the nuclear pore Nup107-160 subcomplex. Proceedings of the National Academy of Sciences of the United States of America.

[43] Zhang W, Liu HT (2002). MAPK signal pathways in the regulation of cell proliferation in mammalian cells. Cell research.

[44] Mebratu Y, Tesfaigzi Y (2009). How ERK1/2 activation controls cell proliferation and cell death: Is subcellular localization the answer?. Cell cycle.

[45] Chambard JC, Lefloch R, Pouyssegur J, Lenormand P (2007). ERK implication in cell cycle regulation. Biochimica et biophysica acta.

[46] Gregoire S (2006). Control of MEF2 transcriptional activity by coordinated phosphorylation and sumoylation. The Journal of biological chemistry.

[47] He CL (2016). Pyruvate Kinase M2 Activates mTORC1 by Phosphorylating AKT1S1. Scientific reports.

[48] Vogelstein B (2013). Cancer genome landscapes. Science.

[49] Tamborero D (2013). Comprehensive identification of mutational cancer driver genes across 12 tumor types. Scientific reports.

[50] Berube NG, Smeenk CA, Picketts DJ (2000). Cell cycle-dependent phosphorylation of the ATRX protein correlates with changes in nuclear matrix and chromatin association. Human molecular genetics.

[51] Hiraga S (2014). Rif1 controls DNA replication by directing Protein Phosphatase 1 to reverse Cdc7-mediated phosphorylation of the MCM complex. Genes & development.

[52] Trzepacz C, Lowy AM, Kordich JJ, Groden J (1997). Phosphorylation of the tumor suppressor adenomatous polyposis coli (APC) by the cyclin-dependent kinase p34. The Journal of biological chemistry.

[53] Hori A, Barnouin K, Snijders AP, Toda T (2016). A non-canonical function of Plk4 in centriolar satellite integrity and ciliogenesis through PCM1 phosphorylation. EMBO reports.

[54] Wu L, Luo K, Lou Z, Chen J (2008). MDC1 regulates intra-S-phase checkpoint by targeting NBS1 to DNA double-strand breaks. Proceedings of the National Academy of Sciences of the United States of America.

[55] Robitaille AM (2013). Quantitative phosphoproteomics reveal mTORC1 activates de novo pyrimidine synthesis. Science.

[56] Savoy RM, Ghosh PM (2013). The dual role of filamin A in cancer: can’t live with (too much of) it, can’t live without it. Endocrine-related cancer.

[57] Blencowe BJ (2000). The SRm160/300 splicing coactivator subunits. Rna.

[58] Jurica MS, Licklider LJ, Gygi SR, Grigorieff N, Moore MJ (2002). Purification and characterization of native spliceosomes suitable for three-dimensional structural analysis. Rna.

[59] Bennetzen MV (2010). Site-specific phosphorylation dynamics of the nuclear proteome during the DNA damage response. Molecular & cellular proteomics: MCP.

[60] Puca R, Nardinocchi L, Givol D, D’Orazi G (2010). Regulation of p53 activity by HIPK2: molecular mechanisms and therapeutical implications in human cancer cells. Oncogene.

[61] Litchfield DW (2003). Protein kinase CK2: structure, regulation and role in cellular decisions of life and death. The Biochemical journal.

[62] Venerando A (2010). Isoform specific phosphorylation of p53 by protein kinase CK1. Cellular and molecular life sciences: CMLS.

[63] Solit DB, Mellinghoff IK (2010). Tracing cancer networks with phosphoproteomics. Nature biotechnology.

[64] Harsha HC, Pandey A (2010). Phosphoproteomics in cancer. Molecular oncology.

[65] Jia R, Li C, McCoy JP, Deng CX, Zheng ZM (2010). SRp20 is a proto-oncogene critical for cell proliferation and tumor induction and maintenance. International journal of biological sciences.

[66] He X (2011). Knockdown of splicing factor SRp20 causes apoptosis in ovarian cancer cells and its expression is associated with malignancy of epithelial ovarian cancer. Oncogene.

[67] Kurokawa K (2014). Downregulation of serine/arginine-rich splicing factor 3 induces G1 cell cycle arrest and apoptosis in colon cancer cells. Oncogene.

[68] Jang HN (2014). Exon 9 skipping of apoptotic caspase-2 pre-mRNA is promoted by SRSF3 through interaction with exon 8. Biochimica et biophysica acta.

[69] Lane C, Qi J, Fawcett JP (2015). NCK is critical for the development of deleted in colorectal cancer (DCC) sensitive spinal circuits. Journal of neurochemistry.

[70] Saitoh N (2012). The distribution of phosphorylated SR proteins and alternative splicing are regulated by RANBP2. Molecular biology of the cell.

[71] Dhillon AS, Hagan S, Rath O, Kolch W (2007). MAP kinase signalling pathways in cancer. Oncogene.

[72] Shi DY (2009). The role of cellular oxidative stress in regulating glycolysis energy metabolism in hepatoma cells. Mol Cancer.

[73] Thingholm TE, Jorgensen TJ, Jensen ON, Larsen MR (2006). Highly selective enrichment of phosphorylated peptides using titanium dioxide. Nature protocols.

