# Supplementary material for: Quantitative Phosphoproteomic Analysis Reveals Key Mechanisms of Cellular Proliferation in Liver Cancer Cells
Source: Sci Rep. 2017 Sep 7;7:10908. doi: 10.1038/s41598-017-10716-0 (PMC5589854; doi:10.1038/s41598-017-10716-0)
Supplement: Supplementary file 1 — Supplementary Information [file 41598_2017_10716_MOESM1_ESM.pdf]

Supplementary Information

Quantitative Phosphoproteomic Analysis Reveals Key Mechanisms of Cellular Proliferation in Liver Cancer Cells

Bo Zhu[1]†, Quanze He[2]†, Jingjing Xiang[2], Fang Qi[3], Hao Cai[1], Jun Mao[2], Chunhua Zhang[2], Qing Zhang[2], Haibo Li[2], Lu Lu[2], Ting Wang[2]*, Wenbo Yu[1]*

[1]State Key Laboratory of Genetic Engineering, Department of Genetics, School of Life Sciences, Fudan University, Shanghai, P.R. China

[2]Center for Reproduction and Genetics, Suzhou Municipal Hospital, Jiangsu

[3]The Second Department of Surgery, Hospital of China No.17 Metallurgical Constrution Corp, Maanshan 243000, Anhui, P.R. China

Supplementary file contained：

Supplementary methods

Figure S1： The profile of experiment design and data analysis.

Figure S2： Evaluation and assessment of MS data quality.

Figure S3： A potential PPI network to promote cellular proliferation of cancer.

Table S1: A list of quantified phosphoproteins in QGY, Hep3B and L02 cell lines.

Table S2: The list for novel phosphsites.

Table S3: Phosphoproteins shared by three celllines and involved in RNA related biological processes.

Table S4: Phosphoproteins shared by three celllines and involved in cell cycle related bilogical processes.

Table S5: Phosphoproteins shared by three celllines and involved in top 20 signaling pathways.

Table S6: The list of phosphorylated proteins encoded by cancer-driven genes

Table S7: The list for up/down-phosphorylated sites in cancer driver genes.

Table S8: The primers list of five genes.

Note: The term of “NA” means un-detected or un-repeated detection in three experiments with one cell line.

Supplementary methods

The method of nano LC-MS/MS analysis

The dried phosphopeptides were dissolved in 10 µl 0.1% FA and analyzed via nanoLC-MS/MS; (Orbitrap Elite). The elution gradient of nano-HPLC (Easy nano Thermo Fisher) is 4 hours and the flow rate is 300 nl/min. The trap column and analysis column were reversed-phase columns and purchased from Thermo Fisher: trap column (C18, 5 µm, 100 Å and 100 µm×2 cm); analysis column (C18, 2 µm, 100 Å and 75 µm×25 cm). The scan method of mass spectrum is MSA which reported by Ulintz PJ research at J Proteome Res in 2009, and the parameters were listed as follows: source fragment action: 35v; segment neutral loss: 32.66, 48.99, 65.32 and 97.98 m/z; MS/MS activation type: CID; isolation width: 2.0; normalized collision energy: 35; activation Q: 0.25; mass resolution for precursor ion analysis: 240,000; mass resolution for product ion analysis: fast ion-trap; number of selected precursors for fragmentation in each scan segment: 15; threshold for triggering tandem-MS: 5,000; dynamic exclusion parameters: 15s; and activation time: 10 ms.

Phosphopeptide identification and phosphosite validation

All three samples were repeated three times and the raw data of them were processed in Proteome Discoverer (PD version 1.4; Thermo Fisher). The database search engine is Sequest-HT and human proteome data from UniProt database (published on 2015/09/27, 84,158 protein sequences) was used. The parameters of Sequest-HT were followed: instrument: ESI-trap; max miss cleavage sites: 2; precursor mass tolerance: 10 ppm; fragment mass tolerance: 0.6 DA; static modification: carbamidomethyl (C). The variable modifications included: acetyl (protein N-term), oxidation (M), gln->pyro-glu (N-term Q), ammonia-loss (N-term C), phospho (ST) and phospho (Y), and the parameters of the decoy were also selected. Finally, all results from Mascot were integrated into PD, and strict criteria were used to filter out potential false positive result (FDR < 0.05) using the Target Decoy PSM Validator (a plug-in PD, Thermo Fisher). The quantity of phosphopeptides was calculated based on the intensity of precursor ions in MS (mass precision 2 ppm) using a plug-in PD (Precursor Ions Area Detector, Thermo Fisher). The reliability of phosphosite were estimated by PhosphoRS (ver: 3.0) and those phosphositeds were remained if their PhosphoRS scores more than 70.

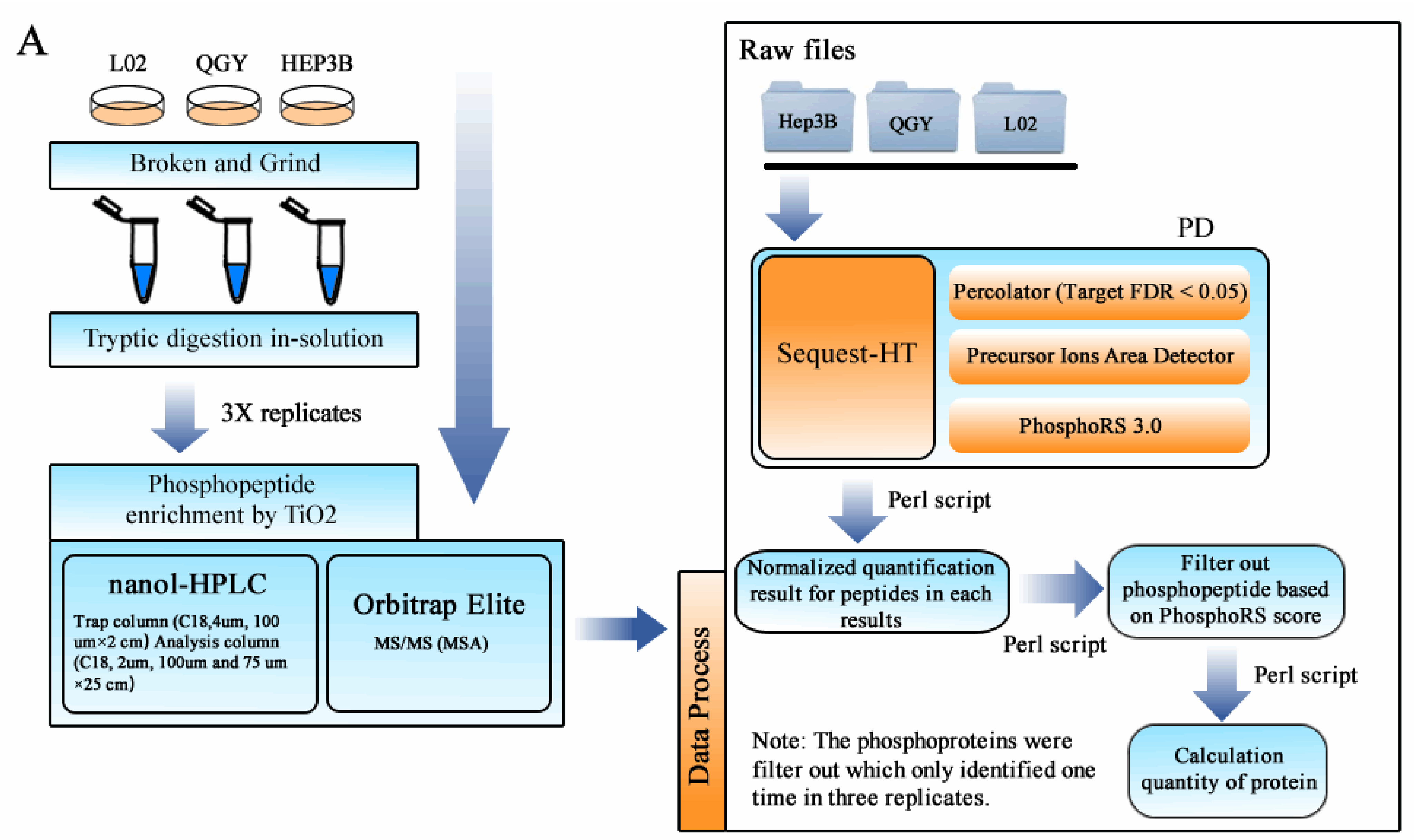

Figure S1: The profile of experiment design and data analysis.

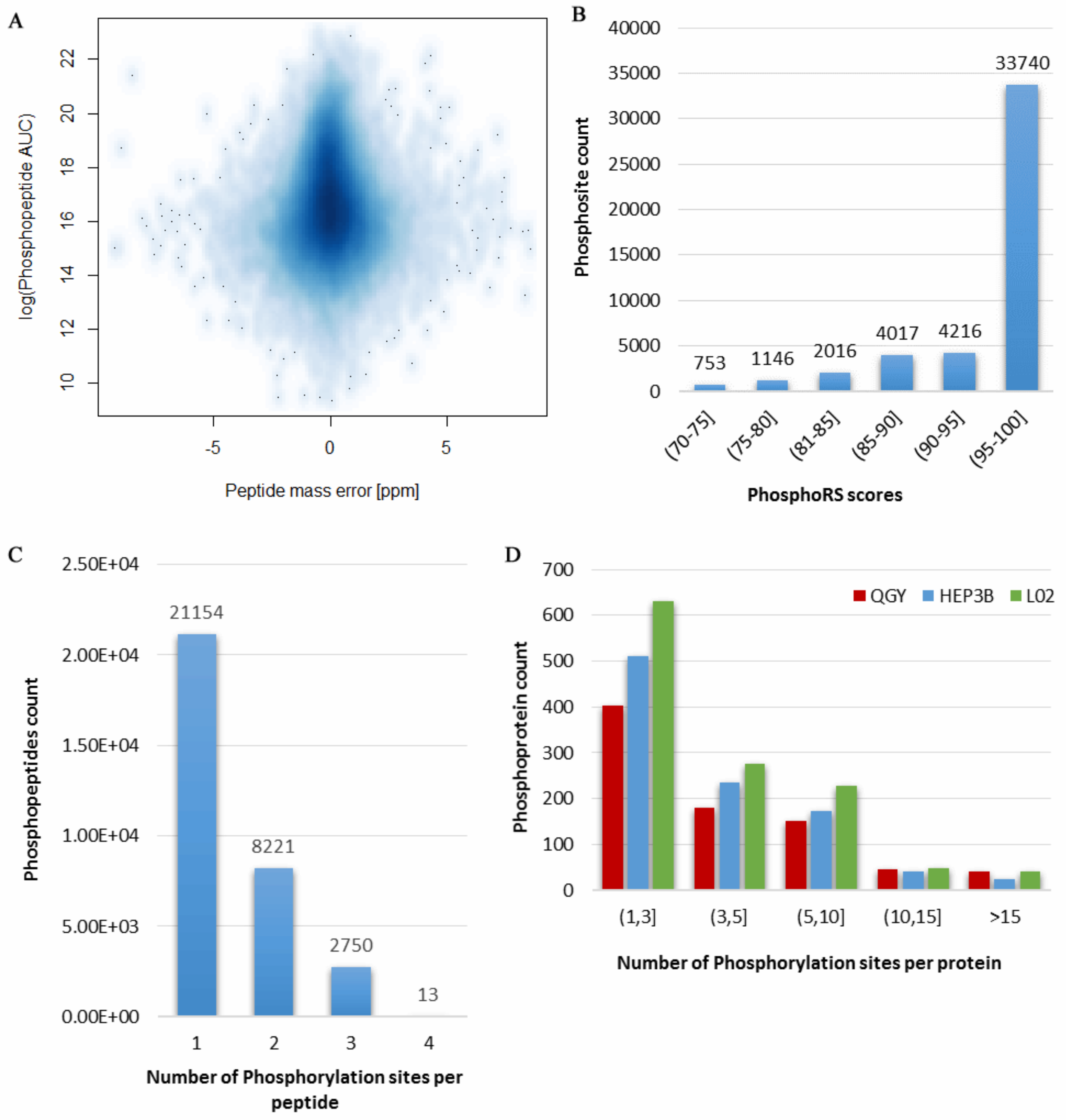

Figure S2: Evaluation and assessment of MS data quality. A) Peptide mass accuracy. Calibrated precursor mass errors measured for all peptides in partsper-million (ppm). B) Histogram illustrating the PhosphoRS score distribution of all phosphosites. C) It demonstrate the number distribution of phosphosites in phosphopeptides. D) Histogram illustration the counting distribution of phosphosite in per phosphoproteins.

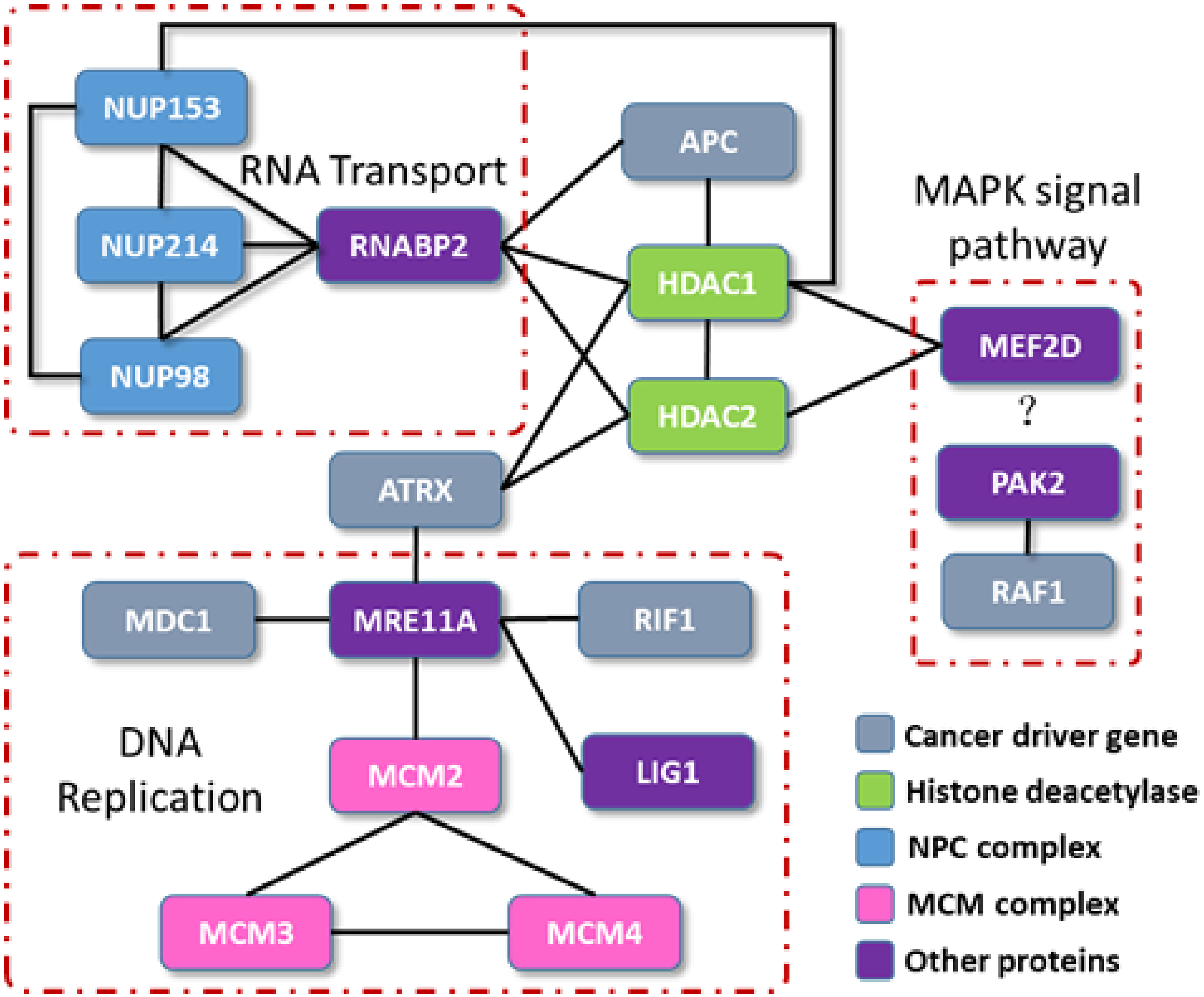

Figure S3: A potential PPI network to promote cellular proliferation of cancer.

Table S1: A list of quantified phosphoproteins in QGY, Hep3B and L02 cell lines.

Accession ID	Gene Name	QGY Sites	QGY Quality	Hep3B Sites	Hep3B Quality	L02 Sites	L02 Quality	QGY/L02	Hep3B/L02
A0FGR8	ESYT2	NA	NA	S761;S755;S758;T753;	5.52E-05	S758;T753;S755;S761;	3.36E-05	NA	1.64E+00
A0MZ66	SHTN1	S494;T537;	9.27E-05	T496;S493;S494;T537;	1.71E-04	T496;S494;	3.97E-05	2.33E+00	4.30E+00
A2RRP1	NBAS	S473;T1804;S1827;	1.97E-05	NA	NA	S473;S475;	6.40E-05	3.08E-01	NA
O00151	PDLIM1	S130;	7.32E-05	NA	NA	NA	NA	NA	NA
O00161	SNAP23	NA	NA	S110;	1.38E-04	S110;	5.21E-05	NA	2.66E+00
O00178	GTPBP1	S25;S44;S47;	6.80E-05	S44;S47;	2.62E-04	S44;S47;	1.03E-04	6.60E-01	2.55E+00
O00193	C11orf58	S15;S17;	3.63E-04	S15;S17;	1.48E-04	S17;S15;	5.65E-04	6.43E-01	2.63E-01
O00203	AP3B1	S750;S752;S276;	1.03E-04	S750;S752;S276;	2.78E-04	S750;S752;S276;	5.02E-04	2.05E-01	5.53E-01
O00257	CBX4	NA	NA	NA	NA	S293;S291;	7.59E-06	NA	NA
O00264	PGRMC1	S57;S181;Y180;	6.28E-03	Y180;S57;S181;	7.63E-03	S57;S181;Y180;	4.05E-03	1.55E+00	1.88E+00
O00267	SUPT5H	NA	NA	S32;S36;	2.38E-04	S32;S36;	2.17E-04	NA	1.09E+00
O00273	DFFA	S315;	1.86E-04	S315;	5.38E-05	S315;	3.76E-05	4.95E+00	1.43E+00
O00303	EIF3F	NA	NA	NA	NA	S258;	5.13E-05	NA	NA
O00418	EEF2K	S470;S474;S477;S27;S31;S18;S445;	2.77E-04	S27;S31;S470;S474;S18;S445;S477;	2.03E-04	S470;S474;S477;S27;S31;S445;S18;	8.51E-05	3.26E+00	2.38E+00
O00499	BIN1	S296;S303;S298;	1.62E-04	S296;S298;S331;	7.77E-05	S296;S298;S303;S331;S333;	5.04E-04	3.22E-01	1.54E-01
O00505	KPNA3	S60;	1.69E-04	S60;S56;	5.29E-04	S60;S56;	9.03E-04	1.87E-01	5.86E-01
O00512	BCL9	NA	NA	NA	NA	T315;S11;	1.49E-05	NA	NA
O00566	MPHOSPH10	S163;S167;S171;	4.40E-05	S167;S171;S163;S242;	5.83E-04	S163;S167;S171;	5.39E-04	8.17E-02	1.08E+00
O00567	NOP56	S569;S570;	5.56E-05	S569;S570;S563;S579;S580;S581;	2.30E-03	NA	NA	NA	NA
O00571	DDX3X	NA	NA	S612;S92;S594;	1.75E-04	NA	NA	NA	NA
O14497	ARID1A	S696;S366;S702;S363;S381;	1.38E-04	S696;S702;S1182;	4.41E-05	S1182;S696;	7.66E-05	1.80E+00	5.75E-01
O14545	TRAFD1	S470;S415;S327;S409;	1.90E-03	S415;S327;	1.75E-04	S327;S415;	6.79E-04	2.80E+00	2.57E-01
O14617	AP3D1	S632;S634;S636;S829;	2.12E-05	S632;S634;S636;	5.03E-05	NA	NA	NA	NA
O14639	ABLIM1	S431;S452;S435;	4.19E-05	S367;S431;S435;S587;S363;S450;S452;	1.79E-04	S431;S435;T451;	1.09E-04	3.86E-01	1.65E+00
O14647	CHD2	S1085;S1087;	1.58E-05	S1085;S1087;S207;S208;	8.79E-06	S207;S208;S1085;S10	1.42E-05	1.11E+00	6.18E-01

						87;T1082;			
O14737	PDCD5	S119;	1.17E-03	S119;	2.57E-05	S119;	3.87E-05	3.02E+01	6.64E-01
O14908	GIPC1	S232;	7.72E-06	NA	NA	NA	NA	NA	NA
O14974	PPP1R12A	S299;S445;T508;S509;S507;S871;S910;S908;	2.09E-04	S910;S871;S862;S507;	3.70E-05	T859;S871;S445;S507;T508;S908;S422;S509;S862;S910;	8.94E-04	2.33E-01	4.13E-02
O15013	ARHGEF10	S59;	1.86E-05	NA	NA	S59;S379;	1.71E-04	1.08E-01	NA
O15014	ZNF609	S605;S609;S467;S1313;S491;S842;S846;S849;S576;S578;T478;	4.89E-05	S842;S849;S576;S578;S846;S1313;	1.10E-04	S842;S846;S849;S576;S578;	7.44E-05	6.57E-01	1.47E+00
O15021	MAST4	S360;S1398;S1406;S1410;T1393;S1394;	5.37E-04	NA	NA	S1406;S1410;T2516;T2519;S2526;S1828;S356;S358;S360;	3.79E-05	1.42E+01	NA
O15027	SEC16A	S391;S1844;S1905;S891;S1178;S1181;S1786;S1044;S1903;S411;S417;S1169;S1172;T415;S1184;S2113;S1175;	4.49E-04	S1844;S1905;S1786;S391;S1169;S1172;S1175;S1149;S1044;S411;S417;S390;S409;S891;	7.39E-04	S1844;T415;S417;S1905;S414;S1786;S391;S891;S1859;S1149;S1903;T1147;S1048;S1178;S411;S1864;S1181;S1044;S1045;S1184;S136;T1876;T397;S1169;S1172;S1050;	3.50E-03	1.28E-01	2.11E-01
O15042	U2SURP	T931;S932;S934;S974;S946;S948;S49;S930;S951;S1012;S1016;S1014;	2.72E-04	S67;S946;S948;S930;S932;S934;S1012;S1014;S1016;S951;	6.28E-04	S946;S948;S974;S67;S930;S932;S934;S951;S788;S800;T919;S1014;	1.67E-04	1.63E+00	3.77E+00
O15061	SYNM	S1044;S1049;S1107;T1109;	9.58E-05	NA	NA	NA	NA	NA	NA
O15119	TBX3	S371;S375;S707;S432;S435;	2.84E-04	NA	NA	NA	NA	NA	NA
O15164	TRIM24	S1042;S1025;S1028;	5.30E-05	S1025;S1028;S811;S1019;S768;S771;S1042;	1.38E-04	S1025;S1028;S811;S1042;S1019;	9.33E-05	5.68E-01	1.48E+00
O15173	PGRMC2	Y210;	1.62E-04	T211;Y210;	8.96E-04	T211;S90;	2.03E-05	8.01E+00	4.42E+01
O15231	ZNF185	T447;S446;S519;T513;S453;S457;	1.34E-04	NA	NA	S465;T513;S519;T447;S453;S455;S446;	1.48E-03	9.05E-02	NA
O15234	CASC3	S148;S265;S363;	2.63E-04	S148;S265;S363;S381;	9.81E-05	S148;S125;S265;S363;S373;T127;S126;Y263;	2.61E-04	1.01E+00	3.76E-01
O15320	CTAGE5	S596;S590;T592;S594;	1.66E-05	S536;S548;Y519;S522;S5	2.18E-05	NA	NA	NA	NA

				90;T592;S596;S517;					
O15446	CD3EAP	S285;S490;S126;S136;S128;	1.82E-04	T287;S136;S128;S124;T282;	4.72E-05	S128;S136;S285;S124;	1.65E-04	1.11E+00	2.87E-01
O15541	RNF113A	S84;S85;S253;	3.86E-04	S84;S85;S253;	6.69E-04	S84;S85;S253;	5.69E-04	6.79E-01	1.18E+00
O43159	RRP8	S62;S64;S104;S106;	6.48E-05	S62;S64;S104;S106;	1.99E-04	S62;S64;S104;S106;S176;S171;S174;	3.84E-04	1.69E-01	5.19E-01
O43166	SIPA1L1	S1645;S1647;S1649;S1585;S1549;	1.00E-04	NA	NA	S1549;S1585;S193;S211;S208;S1181;Y206;	1.76E-04	5.69E-01	NA
O43237	DYNC1LI2	S194;S443;S446;S407;	7.89E-05	S407;S446;S443;	7.86E-05	S446;S407;S381;S391;S443;	5.86E-05	1.35E+00	1.34E+00
O43290	SART1	S448;S474;S486;	2.24E-05	S591;S596;S598;S448;	9.85E-05	S591;S596;S598;S448;	1.71E-04	1.31E-01	5.76E-01
O43395	PRPF3	S619;	1.31E-04	S619;	1.75E-04	S619;	7.21E-04	1.82E-01	2.43E-01
O43399	TPD52L2	S21;S166;	8.48E-04	S166;S96;	1.49E-03	S166;S96;	1.90E-03	4.47E-01	7.86E-01
O43432	EIF4G3	NA	NA	S495;S1156;	1.10E-04	S495;S1409;S1156;	6.89E-05	NA	1.59E+00
O43491	EPB41L2	NA	NA	S598;S715;S39;S58;S798;	3.64E-04	S598;S715;S58;S806;S798;	3.95E-05	NA	9.20E+00
O43493	TGOLN2	S71;	8.09E-05	S71;T296;S70;S68;	1.33E-04	S71;T296;S224;	2.07E-04	3.91E-01	6.44E-01
O43583	DENR	S73;T86;	2.98E-04	S73;	8.53E-05	T69;S73;	1.49E-04	2.00E+00	5.71E-01
O43598	DNPH1	S169;S12;	1.60E-04	S169;S12;	2.81E-04	S169;S12;	2.08E-04	7.68E-01	1.35E+00
O43719	HTATSF1	S642;S616;S624;S702;S713;S714;S498;S721;S579;S597;S600;S387;S676;S607;	1.21E-03	S616;S624;S642;S702;S713;S676;S714;S721;S387;S579;S597;S600;S607;S498;	1.60E-03	S616;S624;S676;S642;S713;S714;S702;S579;S721;S387;S498;S453;	1.46E-03	8.28E-01	1.10E+00
O43765	SGTA	S305;S77;	3.42E-04	S305;	1.38E-04	S305;S77;T81;T303;	3.32E-04	1.03E+00	4.15E-01
O43815	STRN	S245;	3.63E-05	S245;	3.29E-05	S245;	1.34E-04	2.71E-01	2.46E-01
O43818	RRP9	S50;S51;S53;	1.41E-04	NA	NA	S50;S51;S53;	1.23E-04	1.15E+00	NA
O43896	KIF1C	S1033;S674;S676;S1022;	2.56E-05	S1022;S1033;	2.78E-05	S1033;Y671;S676;S674;	9.34E-05	2.74E-01	2.97E-01
O43933	PEX1	NA	NA	NA	NA	S1181;	3.07E-05	NA	NA
O60231	DHX16	S103;S107;S106;S160;	7.07E-04	S106;S107;S103;S160;	3.67E-04	S103;S160;S106;S107;	6.70E-04	1.06E+00	5.48E-01
O60264	SMARCA5	NA	NA	NA	NA	T113;S116;	3.86E-05	NA	NA
O60293	ZFC3H1	S28;S655;S949;S953;	6.07E-05	NA	NA	S28;S1303;S1304;	2.70E-05	2.25E+00	NA
O60341	KDM1A	S131;S137;S166;	1.67E-04	S166;	1.64E-04	S166;S137;S131;	1.58E-04	1.05E+00	1.04E+00
O60502	MGEA5	T370;S364;	1.89E-05	NA	NA	NA	NA	NA	NA

O60504	SORBS3	S530;S373;	1.81E-04	S530;S563;S547;S373;S551;	6.52E-04	S530;S544;S545;	1.75E-04	1.03E+00	3.73E+00
O60566	BUB1B	NA	NA	NA	NA	S543;S670;T1042;	5.19E-05	NA	NA
O60583	CCNT2	S480;	5.71E-05	S480;S536;S537;S538;	5.64E-05	S480;S530;S531;S532;S536;S542;	3.30E-05	1.73E+00	1.71E+00
O60678	PRMT3	S25;S27;	3.07E-04	S25;S27;	4.59E-04	S25;S27;	2.85E-03	1.08E-01	1.61E-01
O60716	CTNND1	NA	NA	S859;Y865;S349;S268;S346;S352;S269;T650;S651;	1.55E-04	NA	NA	NA	NA
O60763	USO1	S942;	1.05E-05	S942;	2.56E-04	S942;	3.18E-04	3.32E-02	8.04E-01
O60784	TOM1	S462;	4.94E-05	S355;S462;S461;	7.07E-04	S462;S464;	6.09E-05	8.11E-01	1.16E+01
O60832	DKC1	S494;S451;S453;S455;S485;S513;T497;	3.27E-04	S451;S455;S453;S513;S21;S494;T497;S485;	3.79E-03	S494;S451;S455;S453;S21;S513;S485;	3.09E-03	1.06E-01	1.23E+00
O60841	EIF5B	S107;S113;S164;S137;S214;S135;S182;S183;S186;	4.69E-03	S135;S137;S107;S113;S214;S182;S183;S186;S164;	6.25E-03	S107;S113;S214;S182;S183;S186;S135;S137;S12;T13;S164;T301;	5.32E-03	8.82E-01	1.17E+00
O60884	DNAJA2	S401;S394;S395;	3.70E-05	S394;S395;Y391;	1.23E-03	S400;S401;S394;S395;	6.58E-04	5.63E-02	1.87E+00
O60885	BRD4	S1126;T1080;S1083;S1117;	4.75E-05	S1126;S1117;	1.14E-05	S1126;S1117;	4.45E-05	1.07E+00	2.56E-01
O75116	ROCK2	S1137;S1134;S25;	3.89E-05	S1134;S1137;	1.97E-04	S1137;S1134;	2.13E-04	1.83E-01	9.25E-01
O75128	COBL	S815;	2.77E-05	NA	NA	NA	NA	NA	NA
O75151	PHF2	S882;S879;	2.04E-05	S879;S882;S840;	7.77E-06	S882;	2.14E-05	9.52E-01	3.63E-01
O75152	ZC3H11A	S495;S108;S758;	2.33E-05	T177;S758;S761;S171;	1.45E-04	T762;S758;S132;S108;S171;S761;	7.27E-05	3.20E-01	1.99E+00
O75153	CLUH	S664;	1.60E-05	NA	NA	S664;	6.68E-05	2.40E-01	NA
O75175	CNOT3	S299;	4.46E-05	NA	NA	S299;	6.18E-05	7.22E-01	NA
O75179	ANKRD17	S2044;S2047;S1696;S1940;S2045;S2059;Y2038;S1319;	9.86E-05	S19;S2401;S2041;S2042;S2047;S1696;	1.75E-04	S2045;S2047;T5;S2401;S1709;S19;S2067;S2059;S2042;	4.21E-04	2.34E-01	4.15E-01
O75351	VPS4B	S102;	3.75E-05	S102;	8.30E-05	S102;	2.62E-04	1.43E-01	3.17E-01
O75376	NCOR1	S2184;S1472;S1206;S2136;S2151;S157;S2120;Y1966;S2436;S2438;S1977;S1981;S158;S172;	2.87E-04	S2184;S2151;S1472;S224;S2436;S2438;S157;S172;S1322;S1977;S1981;S158;	3.86E-04	S2436;S2438;S2184;S2151;S158;S2120;S224;S1977;S1472;S2136;S999;S1196;	3.52E-04	8.16E-01	1.10E+00
O75400	PRPF40A	S933;S935;S938;S883;S888;S885;S903;	1.33E-03	S935;S938;S885;S888;S933;S883;T932;	6.71E-04	S933;S935;S938;S883;S885;S888;T932;	7.88E-04	1.69E+00	8.51E-01
O75475	PSIP1	S273;S275;T267;T272;S17	1.78E-03	T272;S273;S275;S271;T1	4.83E-04	S273;S275;S106;S129;	1.92E-04	9.27E+00	2.52E+00

		7;S129;S106;S271;T122;S102;		69;S106;T115;S116;T167;		T115;			
O75494	SRSF10	S129;S131;S133;S156;S158;S160;S119;S121;S123;S141;S251;T255;S256;	3.01E-03	S129;S131;S133;S123;S119;S121;S156;S158;Y142;	2.69E-03	S129;S131;S133;S156;S158;S160;S256;S119;S251;T255;S121;	2.16E-03	1.39E+00	1.24E+00
O75528	TADA3	S298;S296;	2.32E-05	NA	NA	NA	NA	NA	NA
O75554	WBP4	S262;	3.11E-05	NA	NA	S262;	1.22E-05	2.54E+00	NA
O75569	PRKRA	NA	NA	S18;	1.53E-04	S18;	4.47E-05	NA	3.42E+00
O75607	NPM3	S147;S151;S158;	4.72E-05	NA	NA	NA	NA	NA	NA
O75643	SNRNP200	NA	NA	S225;	4.66E-05	S225;	5.66E-05	NA	8.23E-01
O75674	TOM1L1	S323;	2.07E-05	S323;	1.61E-04	S321;S323;	1.32E-04	1.56E-01	1.22E+00
O75676	RPS6KA4	NA	NA	NA	NA	S745;T687;S343;S347;	2.63E-05	NA	NA
O75691	UTP20	T1741;S2523;	3.68E-05	T1741;	6.77E-05	T1741;S2601;	7.98E-05	4.61E-01	8.48E-01
O75717	WDHD1	S1090;S868;	2.58E-05	S868;	4.30E-05	S868;S1090;	5.25E-04	4.91E-02	8.18E-02
O75815	BCAR3	S83;	2.12E-05	NA	NA	NA	NA	NA	NA
O75909	CCNK	S340;S324;S329;	2.29E-04	S340;S324;	2.03E-04	S329;S324;S340;	2.77E-04	8.28E-01	7.34E-01
O75962	TRIO	S2429;S2426;S2455;S2459;	3.69E-05	NA	NA	S2455;S2459;S2426;	4.55E-05	8.11E-01	NA
O75971	SNAPC5	NA	NA	NA	NA	S96;	1.65E-05	NA	NA
O76021	RSL1D1	S427;S392;S396;S400;T423;S421;S361;T340;T401;	1.09E-03	S427;T358;S392;S396;S361;T401;T423;	3.82E-03	S392;S396;S427;T423;T340;S421;S400;S361;T465;T401;	5.67E-03	1.93E-01	6.73E-01
O76094	SRP72	T618;	4.57E-04	T618;S625;S610;	3.16E-03	T618;S625;	6.72E-04	6.80E-01	4.71E+00
O94762	RECQL5	S815;	3.44E-05	S815;	6.68E-05	S488;S491;S815;Y484;	5.56E-05	6.18E-01	1.20E+00
O94804	STK10	S438;T952;	2.23E-04	S438;	3.94E-05	S438;	2.23E-04	1.00E+00	1.77E-01
O94826	TOMM70A	S91;	1.07E-04	S91;T85;	6.30E-05	NA	NA	NA	NA
O94842	TOX4	S178;S181;S182;	3.73E-05	NA	NA	T176;S178;	5.05E-05	7.39E-01	NA
O94874	UFL1	S458;	5.00E-05	S458;	1.25E-04	S458;	1.89E-04	2.64E-01	6.59E-01
O94880	PHF14	S530;S25;S26;	1.05E-05	NA	NA	S290;T292;T287;S530;S25;S26;S835;	2.84E-05	3.69E-01	NA
O94913	PCF11	S370;S372;S509;S511;S494;	8.99E-05	NA	NA	S182;S502;S370;S372;	1.92E-05	4.67E+00	NA
O94979	SEC31A	S799;S527;	3.90E-05	S799;	8.65E-04	S527;S532;S1163;S79	8.77E-04	4.45E-02	9.87E-01

						9;T1165;			
O95071	UBR5	S286;S287;S289;S2192;T2213;S1549;	1.67E-04	S287;S327;S1549;S289;	5.61E-04	S287;S1549;S286;	1.13E-04	1.48E+00	4.98E+00
O95155	UBE4B	S105;S88;Y862;T866;S871;	9.51E-05	NA	NA	S101;S78;S92;S803;S31;S88;	2.99E-05	3.18E+00	NA
O95218	ZRANB2	S153;S120;S188;S318;	1.85E-03	S153;S188;S120;	8.59E-04	S120;S188;S153;	3.12E-03	5.95E-01	2.75E-01
O95232	LUC7L3	S425;S431;T303;S304;	6.66E-05	S425;S431;	3.10E-04	S431;T419;S425;	1.39E-04	4.79E-01	2.23E+00
O95251	KAT7	S57;S50;S56;T88;	1.88E-05	S57;S99;S100;S102;T88;	8.78E-05	S50;S52;S57;S100;S102;T104;	9.59E-06	1.96E+00	9.16E+00
O95359	TACC2	S2317;S2321;S2359;S2226;S2072;	2.92E-04	S2317;S2321;	2.43E-04	T2082;S2084;S2317;S2321;	4.60E-05	6.35E+00	5.29E+00
O95361	TRIM16	S60;	7.16E-05	NA	NA	S24;S34;S38;S60;T55;S29;	3.45E-04	2.08E-01	NA
O95365	ZBTB7A	S526;S549;S337;	9.80E-05	S525;S549;S526;S337;	7.48E-05	S549;S526;S337;	5.98E-05	1.64E+00	1.25E+00
O95391	SLU7	S235;S513;S515;S215;S466;	1.47E-04	NA	NA	S215;	2.01E-04	7.35E-01	NA
O95400	CD2BP2	S49;S194;	8.88E-04	S49;S194;S195;	4.47E-04	S49;S195;S194;	9.07E-04	9.78E-01	4.92E-01
O95425	SVIL	S1000;	3.02E-04	NA	NA	Y966;S245;S968;	6.54E-05	4.62E+00	NA
O95429	BAG4	NA	NA	NA	NA	S179;	8.96E-05	NA	NA
O95433	AHSA1	S193;	2.62E-05	NA	NA	NA	NA	NA	NA
O95453	PARN	S557;S570;S587;	1.59E-05	S570;S587;S557;S628;S619;S163;	2.06E-04	S570;S587;S167;S557;S628;S163;S619;S623;S625;	2.38E-04	6.69E-02	8.66E-01
O95466	FMNL1	S1031;	5.87E-05	NA	NA	S1031;	7.30E-05	8.03E-01	NA
O95628	CNOT4	S432;	1.87E-05	S432;	1.28E-04	S324;S432;S430;	1.28E-04	1.47E-01	1.01E+00
O95639	CPSF4	NA	NA	S202;	6.30E-05	S200;S202;S212;	6.52E-05	NA	9.66E-01
O95684	FGFR1OP	S156;S160;S321;S326;	1.02E-04	S156;S160;S152;	3.60E-04	S156;S160;	8.76E-05	1.17E+00	4.11E+00
O95747	OXSR1	S425;S324;S339;	1.31E-04	S339;S159;	1.02E-04	S339;S427;T239;S246;T249;	7.85E-04	1.67E-01	1.30E-01
O95785	WIZ	S996;S1012;S1017;S1146;S1006;S1151;S1127;	2.66E-04	S1012;S1017;S1146;T1153;	1.05E-04	S1012;S1017;S1127;S1134;S1006;S1146;S1151;S1155;S1517;S1309;S1314;S996;	1.54E-04	1.73E+00	6.80E-01
O95817	BAG3	S171;S177;S377;S279;T406;S274;S275;T285;S289;S264;S386;S291;	2.98E-03	S275;S377;S386;	8.57E-05	S171;S274;S279;S377;S275;T406;T285;S289;	5.43E-04	5.50E+00	1.58E-01

O95997	PTTG1	S165;	3.91E-05	S165;	8.61E-06	S165;	1.15E-05	3.41E+00	7.49E-01
O96028	WHSC1	S579;S580;S447;	5.22E-06	NA	NA	NA	NA	NA	NA
P02545	LMNA	S403;S404;S407;S615;T19;S22;S636;S414;S390;S392;S277;T416;S612;S12;S458;S613;S406;S652;T424;T394;	2.25E-02	S22;S628;S390;S392;S395;S406;S404;S407;S616;T19;S403;T409;S632;	4.14E-03	S616;S636;T19;S22;S406;S404;S407;S390;S392;T409;T394;S277;S414;S403;S612;S628;T416;	1.45E-02	1.56E+00	2.86E-01
P02765	AHSG	NA	NA	S138;	1.95E-04	S138;	1.16E-03	NA	1.69E-01
P02794	FTH1	S179;	4.69E-04	NA	NA	T175;S179;	2.11E-04	2.22E+00	NA
P04049	RAF1	S43;T260;S621;T303;	9.65E-05	S621;S296;	4.21E-04	S642;T303;S43;S621;S289;S296;S301;	6.75E-04	1.43E-01	6.23E-01
P04083	ANXA1	NA	NA	NA	NA	S37;	4.39E-05	NA	NA
P04150	NR3C1	S134;S45;	2.99E-04	NA	NA	S134;	7.35E-05	4.07E+00	NA
P04792	HSPB1	S199;S65;S82;S15;	1.12E-02	S82;S15;	1.19E-02	S82;S15;	7.65E-03	1.46E+00	1.56E+00
P05114	HMGN1	S86;S89;S99;S7;	1.54E-04	T81;S89;S86;S7;	1.30E-04	S99;S86;S89;T81;S7;	8.33E-05	1.85E+00	1.56E+00
P05387	RPLP2	S17;S102;S105;S86;	1.79E-02	S102;S105;S79;S17;	8.26E-03	S17;S102;S105;S79;S74;S86;	6.88E-03	2.60E+00	1.20E+00
P05388	RPLP0	S304;S307;	2.77E-03	S304;S307;	1.40E-03	S304;S307;	1.29E-03	2.15E+00	1.09E+00
P05455	SSB	S366;S92;	2.83E-03	S366;S92;	4.12E-03	S366;T379;	3.54E-03	8.00E-01	1.16E+00
P05783	KRT18	S399;S401;	5.02E-05	NA	NA	NA	NA	NA	NA
P05787	KRT8	S74;S432;S253;S475;Y25;S43;S34;S330;S37;	1.23E-04	S478;S24;S43;	9.20E-04	S475;S43;S37;S330;S35;S478;	5.43E-04	2.26E-01	1.69E+00
P06493	CDK1	NA	NA	Y15;T14;	8.99E-04	Y15;T14;	8.55E-04	NA	1.05E+00
P06748	NPM1	S125;S70;S254;S260;T234;T237;S243;S227;S222;T199;S139;T219;	6.79E-03	S125;T199;S227;S260;S243;S214;T219;	1.40E-03	S125;S70;T199;S260;T234;S139;S243;T219;S227;	3.88E-03	1.75E+00	3.62E-01
P07814	EPRS	S882;S886;S885;S845;S880;T888;	2.25E-03	S882;S886;S845;	2.46E-03	S882;S886;S999;S883;S1000;S845;	3.09E-03	7.28E-01	7.98E-01
P07900	HSP90AA1	S263;S231;	3.99E-03	S263;S231;S252;	1.10E-02	S231;S263;	1.47E-02	2.71E-01	7.45E-01
P07910	HNRNPC	S260;S253;S299;S233;S162;S239;	3.77E-03	S260;S299;S233;	7.34E-04	S260;S253;S299;S233;S162;S241;	3.20E-03	1.18E+00	2.29E-01
P08047	SP1	S59;S42;	3.65E-05	NA	NA	S56;S59;	9.80E-06	3.73E+00	NA
P08238	HSP90AB1	S255;S261;S226;	2.29E-02	S255;S261;S226;	5.46E-02	S255;S261;S226;	4.18E-02	5.49E-01	1.31E+00
P08240	SRPR	S296;S297;S298;	2.98E-04	S296;S297;S298;S307;T3	1.12E-03	S296;S297;S298;T284;	3.78E-04	7.89E-01	2.98E+00

				08;		S286;			
P08559	PDHA1	NA	NA	S232;	8.47E-06	NA	NA	NA	NA
P08621	SNRNP70	S226;S268;S410;S293;S295;	2.22E-04	S410;S268;	5.75E-05	S410;S226;S268;	1.30E-04	1.71E+00	4.42E-01
P08651	NFIC	S323;S333;S339;S194;	5.97E-05	NA	NA	S194;	4.34E-05	1.38E+00	NA
P08670	VIM	S56;S459;S430;S10;	2.22E-03	S420;S459;S56;S430;	2.05E-04	S56;S430;S325;S419;S459;	7.78E-04	2.86E+00	2.64E-01
P09651	HNRNPA1	S337;S6;S368;S199;	5.88E-03	S363;S6;S368;S95;	6.78E-03	S361;S6;S337;S365;	7.68E-03	7.66E-01	8.83E-01
P0C1Z6	TFPT	S180;S249;S252;	7.42E-05	S249;S252;S180;	1.55E-04	S249;S252;S180;	1.32E-04	5.64E-01	1.18E+00
P0DJ93	SMIM13	S58;T62;S60;	2.28E-05	S60;T62;S58;S50;	6.25E-04	S58;T62;	1.26E-04	1.81E-01	4.96E+00
P10412	HIST1H1E	S187;	6.04E-05	S36;S187;	1.53E-03	S36;S187;T146;	2.19E-04	2.76E-01	6.97E+00
P10636	MAPT	S713;S717;S721;S519;T522;T720;	3.20E-04	NA	NA	S713;S721;S717;T720;S428;S516;S519;T498;S552;T548;S411;	1.33E-03	2.41E-01	NA
P10644	PRKAR1A	S83;	1.20E-04	S83;T75;S77;	1.06E-03	S83;T75;S77;	5.07E-04	2.37E-01	2.09E+00
P10809	HSPD1	NA	NA	NA	NA	S70;	4.57E-05	NA	NA
P11171	EPB41	NA	NA	NA	NA	S709;T490;T492;T494;S188;	8.42E-06	NA	NA
P11388	TOP2A	S1106;S1247;S1469;S1471;S1474;S1393;S1377;S1525;T1343;T1470;T930;T932;T934;Y935;S1374;T1112;S1332;S1337;S1351;S1213;S1392;	1.90E-03	S1247;S1374;S1377;S1393;S1106;S1471;S1474;T1470;S1525;T1397;S1332;S1337;T1343;S1469;S29;	2.71E-03	S1106;S1374;S1377;S1247;S1525;S1471;S1474;S1476;T1397;S29;	1.07E-03	1.78E+00	2.54E+00
P11717	IGF2R	S2484;S2409;	4.59E-05	S2484;S2409;S2479;	3.46E-04	S2484;S2479;S2409;	4.18E-04	1.10E-01	8.26E-01
P11831	SRF	S224;	5.56E-05	NA	NA	NA	NA	NA	NA
P12270	TPR	S379;S2048;T2042;	1.30E-04	S2048;	1.76E-05	S2048;S1185;S2034;S2155;	6.42E-05	2.02E+00	2.73E-01
P12694	BCKDHA	NA	NA	S347;	6.09E-05	S347;	1.98E-05	NA	3.07E+00
P13051	UNG	S23;T60;	1.44E-04	T31;T60;S63;	8.97E-05	S23;T60;S64;	3.81E-04	3.78E-01	2.36E-01
P13807	GYS1	NA	NA	S710;S653;S657;	2.86E-05	S727;S657;S653;S698;S652;	1.57E-05	NA	1.82E+00
P13861	PRKAR2A	S99;S78;S80;	1.87E-03	S78;S80;S99;T104;	1.49E-03	S99;S78;S80;	3.17E-03	5.89E-01	4.70E-01
P13984	GTF2F2	NA	NA	NA	NA	S248;	1.24E-05	NA	NA

P14618	PKM	S37;	3.45E-05	NA	NA	S37;	1.65E-04	2.09E-01	NA
P15056	BRAF	NA	NA	S365;S151;	6.64E-05	S365;S151;S447;S729;S363;	2.95E-05	NA	2.25E+00
P15336	ATF2	T69;T71;S328;T320;	6.03E-05	T69;T71;S328;S62;	3.02E-04	NA	NA	NA	NA
P15408	FOSL2	S230;S200;	1.62E-04	NA	NA	S230;S16;S19;S307;S320;S314;S315;	4.62E-05	3.51E+00	NA
P15923	TCF3	S379;	7.83E-05	NA	NA	S379;	3.53E-05	2.22E+00	NA
P15924	DSP	S2608;S2022;S165;S166;S2825;S2849;	1.03E-04	S165;S166;S2209;Y172;T173;	1.79E-04	S165;S166;S2608;S2606;	1.72E-04	5.98E-01	1.04E+00
P16104	H2AFX	S122;S140;	2.59E-05	NA	NA	S140;S122;	1.46E-05	1.77E+00	NA
P16333	NCK1	S85;	3.54E-05	S85;	1.51E-05	S166;S85;	9.78E-05	3.62E-01	1.54E-01
P16383	GCFC2	S174;S180;S16;S17;S19;	5.77E-05	S16;S17;S19;S174;S180;S96;T97;S40;	1.82E-04	S16;S19;S25;S174;S180;S17;S96;T97;	2.11E-04	2.74E-01	8.65E-01
P16949	STMN1	S16;S25;S38;S63;	5.98E-04	S16;S25;S38;S63;S46;	6.20E-04	S16;S25;S38;S63;S46;	9.79E-04	6.10E-01	6.33E-01
P16989	YBX3	S201;S203;S204;S34;S134;S38;S79;	1.07E-03	S201;S203;S204;S38;S79;S369;	6.61E-05	S201;S203;S204;S34;	1.80E-04	5.97E+00	3.67E-01
P17029	ZKSCAN1	S13;	2.59E-05	NA	NA	NA	NA	NA	NA
P17096	HMGA1	S102;S99;S103;S36;T53;T39;S49;	1.30E-02	S99;S102;S103;S36;T53;T39;S44;	2.03E-02	S99;S102;S103;S36;T39;T53;	9.41E-03	1.38E+00	2.16E+00
P17535	JUND	S90;S255;S259;S100;	4.78E-05	NA	NA	NA	NA	NA	NA
P17544	ATF7	T51;T53;	3.09E-05	NA	NA	T51;T53;	5.79E-05	5.33E-01	NA
P17676	CEBPB	T235;	1.53E-04	NA	NA	S237;T235;	4.68E-04	3.26E-01	NA
P18583	SON	S1780;S1782;S1556;S2009;S2011;S2013;S1697;S1783;S1769;S94;S1026;S2029;S2031;S283;S910;S2020;T2022;S1948;S1950;S1954;S1874;S1876;S1784;S1885;S1887;S1952;S1829;S1831;S1832;S1929;S1931;T1933;	1.86E-03	S2009;S2011;S2013;S1697;S283;S2020;T2022;S1950;S1952;S2001;S2003;S2129;S1940;S1942;T1555;S2029;S2031;S1948;S1829;S1831;	1.29E-03	S2009;S2011;S2013;S1697;S1948;S1950;S1952;S1784;S2029;S2031;S1782;S1783;S283;S2020;T2022;	1.30E-03	1.43E+00	9.92E-01
P18615	NELFE	S49;S115;S353;S181;S251;S185;	9.19E-05	S115;S353;S251;S113;S178;	8.65E-04	S353;S165;S181;S251;S187;S191;S115;	4.21E-04	2.18E-01	2.05E+00
P18669	PGAM1	S118;	1.48E-04	NA	NA	NA	NA	NA	NA
P18754	RCC1	S11;	4.39E-05	NA	NA	S11;	4.19E-05	1.05E+00	NA
P18846	ATF1	S198;S186;S36;	1.53E-04	NA	NA	NA	NA	NA	NA

P18858	LIG1	S911;S913;S76;T195;S66;S141;	3.31E-04	S911;S913;S66;S76;S141;T197;S47;S49;	6.13E-04	S66;S76;S141;S911;S913;T195;S199;S47;T183;S49;S51;	6.80E-04	4.86E-01	9.02E-01
P18887	XRCC1	S241;T453;T440;S226;S447;S266;	2.91E-04	T202;S226;S241;S199;T453;S447;S461;S485;T488;	1.07E-03	S241;S447;T457;S446;S204;S226;S236;T453;S266;S229;	5.12E-04	5.69E-01	2.09E+00
P19105	MYL12A	S19;	3.63E-05	S19;	1.46E-05	NA	NA	NA	NA
P19338	NCL	S28;S34;S41;S145;S153;S206;S184;S67;S563;T84;S42;T121;T69;T106;T99;	1.00E-02	S145;S153;S563;T76;T106;S67;S28;S34;S41;T69;T121;	1.73E-03	S145;S153;S184;S206;T69;S67;S563;T121;S28;S34;S41;T76;T106;T84;T99;	2.78E-03	3.61E+00	6.23E-01
P20700	LMNB1	T575;S391;S393;S23;T20;S395;S396;	3.00E-03	S391;S393;T20;T25;T575;S395;S396;	1.68E-03	S391;S393;T399;S401;T20;S23;T575;	2.05E-03	1.47E+00	8.22E-01
P20749	BCL3	NA	NA	S374;	8.66E-06	S374;T381;T368;T363;	3.60E-05	NA	2.41E-01
P20810	CAST	S243;	6.77E-04	S243;T240;	1.44E-03	S243;S364;S563;	8.14E-04	8.32E-01	1.77E+00
P21291	CSRP1	S192;	3.35E-04	S192;	1.06E-04	S192;	3.30E-04	1.01E+00	3.21E-01
P21333	FLNA	S1084;S1459;S2152;S1906;	3.54E-04	S2152;	2.08E-05	S1338;S2152;S1084;S1459;S2327;S2158;	1.77E-03	2.01E-01	1.18E-02
P21796	VDAC1	S104;	1.06E-05	NA	NA	S104;	3.52E-05	3.00E-01	NA
P22059	OSBP	S190;S193;	1.14E-04	S190;S193;S382;S385;S198;	6.94E-05	S190;S193;S382;S386;S379;S385;	2.07E-04	5.49E-01	3.35E-01
P22234	PAICS	S27;	1.30E-05	S27;	3.85E-05	S27;	1.61E-04	8.05E-02	2.39E-01
P22314	UBA1	NA	NA	NA	NA	S46;	6.43E-05	NA	NA
P22626	HNRNPA2B1	S259;S344;S236;	5.20E-04	S259;S212;	7.64E-04	S259;S212;S236;	6.35E-04	8.20E-01	1.20E+00
P23193	TCEA1	S100;	8.67E-04	S100;	2.70E-03	S100;	7.22E-04	1.20E+00	3.74E+00
P23497	SP100	S407;S409;S410;	2.71E-04	S407;S409;S410;	3.23E-04	S407;S409;S410;S157;S111;	1.06E-04	2.55E+00	3.04E+00
P23588	EIF4B	S497;S498;S504;S422;S597;S424;S406;S409;S207;S93;S219;S459;S192;S495;S425;S462;T420;S442;S489;S445;S434;	7.40E-03	S497;S504;S495;S498;S597;T420;S424;S459;S425;S93;S489;S283;S422;Y96;S406;T427;	3.40E-03	S497;S498;S495;S504;S597;S422;S424;S93;S425;S459;S192;S406;S409;T500;T420;T450;T197;Y285;S418;S462;S489;	6.92E-03	1.07E+00	4.90E-01
P24534	EEF1B2	S106;S95;	1.16E-02	S106;S95;S90;	3.95E-03	S106;S95;	1.64E-02	7.07E-01	2.40E-01
P24928	POLR2A	S1906;S1913;T1915;S1920	3.49E-04	S1913;T1919;Y1853;S187	3.73E-04	Y1874;T1884;S1847;S	9.25E-05	3.78E+00	4.03E+00

		;S1878;S1843;T1854;Y1853;S1882;S1847;		8;T1884;S1843;S1966;Y1909;T511;Y1916;		1878;Y1909;S1910;S1920;S1913;S1843;Y1853;S1906;				
P25054	APC	S2671;S1044;S1360;S559;S2830;S1861;S1863;S1864;S1436;	6.49E-05	S1861;S1863;S1864;S1360;S2088;S2093;S2830;S780;S2772;S2283;	4.61E-05	S2088;S2093;S780;S1861;S1864;S2671;S1863;S2674;S1360;S2830;	4.41E-05	1.47E+00	1.05E+00
P25205	MCM3	S672;T674;S711;T713;T722;	1.07E-03	S672;T674;S711;T722;Y708;	3.68E-04	S672;T674;T722;S711;	4.27E-04	2.50E+00	8.62E-01
P25490	YY1	S247;	6.59E-05	NA	NA	NA	NA	NA	NA
P25788	PSMA3	S250;	2.71E-03	S250;	8.07E-05	S250;	5.25E-04	5.15E+00	1.54E-01
P26358	DNMT1	S714;S127;S143;S1467;S1468;S1469;	1.25E-04	S143;	1.84E-05	NA	NA	NA	NA
P26368	U2AF2	S79;	2.11E-04	S79;	3.22E-04	S79;	8.98E-05	2.35E+00	3.58E+00
P27708	CAD	S1859;	1.83E-05	S1859;	3.27E-04	S1859;S1038;	8.26E-04	2.21E-02	3.96E-01
P27816	MAP4	S507;S510;T521;S280;S789;S636;S624;S1073;S928;S358;T627;S787;T354;S1151;T828;S822;T687;S941;	3.90E-03	S636;S1151;S787;S822;T521;S928;T942;T270;S827;T82;S280;	2.76E-04	S1151;T521;S636;S99;T828;S787;T917;S928;T82;S94;T571;	7.83E-04	4.99E+00	3.53E-01
P27824	CANX	S554;S564;T562;S583;	5.05E-03	S554;S564;T562;S583;	2.33E-02	S554;S564;T562;S583;	8.67E-03	5.83E-01	2.69E+00
P28290	SSFA2	S739;S746;T1168;S1174;S92;S759;	1.43E-04	S737;S739;S318;S320;S759;	1.17E-04	S92;S1174;S737;S739;S668;S1175;	1.45E-04	9.83E-01	8.03E-01
P28715	ERCC5	S562;S563;S355;S356;S357;S384;	1.63E-04	S384;S341;S526;S562;S563;S532;	3.42E-04	S384;S563;S562;S526;	2.24E-04	7.27E-01	1.53E+00
P29590	PML	S36;S518;S527;S530;	3.05E-05	S518;S527;S530;	9.86E-05	S518;S527;S530;S36;S38;S40;S480;S403;S504;S505;	2.85E-04	1.07E-01	3.45E-01
P29692	EEF1D	T147;S162;S133;	1.00E-02	T147;S162;S133;	5.00E-03	T147;S162;S133;	6.61E-03	1.52E+00	7.58E-01
P29966	MARCKS	S118;S83;S101;S46;S27;T150;S145;T143;S26;S170;S159;T120;S135;S81;S77;S29;S128;	1.21E-03	S81;T120;S128;S134;S101;T143;S46;S27;S118;S145;T150;S167;S170;S163;S131;S132;S135;S147;S83;	2.94E-02	S46;S101;T150;S29;S27;S145;S170;S163;T120;S118;S135;S167;S81;	5.93E-03	2.04E-01	4.96E+00
P30414	NKTR	S887;S889;S891;S699;S701;	2.17E-04	S463;S887;S889;S891;Y702;S703;S699;S701;	1.99E-04	S463;S887;S889;S891;S379;	6.96E-05	3.12E+00	2.85E+00
P30622	CLIP1	S204;S147;S43;S48;	2.58E-04	NA	NA	S195;S44;S48;S204;S197;	5.49E-05	4.70E+00	NA
P31350	RRM2	S20;	1.62E-04	S20;	1.50E-04	S20;	6.00E-04	2.70E-01	2.50E-01

P31943	HNRNPH1	S104;	1.00E-03	S104;	7.01E-04	S310;S104;	1.07E-03	9.37E-01	6.55E-01
P31948	STIP1	S481;	1.19E-04	S481;	1.34E-04	S481;	2.45E-04	4.86E-01	5.45E-01
P33240	CSTF2	S524;	2.73E-04	NA	NA	NA	NA	NA	NA
P33991	MCM4	S26;S32;S31;	4.14E-05	S131;S142;S145;S26;S31;S32;S34;	5.67E-05	S131;S772;T774;T778;S26;S105;T110;	2.29E-05	1.81E+00	2.48E+00
P35221	CTNNA1	S641;T634;	3.86E-04	S641;S652;S655;T658;T654;T634;	2.26E-03	T634;S652;S641;T654;S655;	8.56E-04	4.51E-01	2.64E+00
P35251	RFC1	S69;S71;S73;S108;	3.40E-04	S69;S71;S156;T161;S312;S368;S73;S29;	1.50E-03	S69;S71;S368;	1.58E-04	2.15E+00	9.49E+00
P35269	GTF2F1	S385;S377;T389;T445;T446;S433;	1.07E-04	S385;T389;S224;S391;S433;	1.49E-04	S385;S391;S433;S442;T389;T445;	1.85E-04	5.81E-01	8.07E-01
P35579	MYH9	S1943;	1.43E-02	NA	NA	S1943;T1151;	6.00E-03	2.38E+00	NA
P35580	MYH10	S1956;S1975;S1952;	5.41E-05	S1952;S1956;S1975;T1960;	2.22E-04	S1956;S1975;S1952;	1.20E-04	4.53E-01	1.85E+00
P35611	ADD1	S465;S358;S726;T16;S12;	7.34E-05	S465;S358;S12;S427;Y407;S355;	1.63E-03	S465;S358;	8.75E-05	8.39E-01	1.86E+01
P35658	NUP214	T436;T987;S988;S433;T439;T670;S678;T1981;S1985;T2007;T437;S657;	3.72E-04	T434;T437;T436;T670;S678;S433;S457;	5.16E-04	S1023;S433;T437;S985;S986;T670;S678;S430;S648;S646;	9.30E-04	4.00E-01	5.55E-01
P35659	DEK	S301;S306;S307;S32;S243;S244;S251;S303;	6.19E-04	S32;S243;S244;S251;S301;S303;	7.75E-04	S32;S51;S121;S122;S243;S244;S251;S306;S307;	1.60E-03	3.87E-01	4.84E-01
P36507	MAP2K2	NA	NA	S23;T396;	9.00E-04	NA	NA	NA	NA
P36578	RPL4	NA	NA	NA	NA	S295;T339;	1.01E-04	NA	NA
P37802	TAGLN2	S163;	9.81E-05	S163;	8.03E-05	S163;	1.23E-04	7.94E-01	6.50E-01
P38159	RBMX	S208;S88;	4.06E-04	S208;S88;	3.76E-04	NA	NA	NA	NA
P38398	BRCA1	S395;S398;	1.63E-05	NA	NA	S395;S398;	1.92E-05	8.46E-01	NA
P40123	CAP2	S309;	1.14E-04	S301;T311;S312;	9.12E-05	S309;T311;	3.01E-05	3.77E+00	3.03E+00
P40222	TXLNA	S515;	7.04E-04	S35;S515;S18;	7.50E-04	S515;S19;S495;	1.11E-03	6.32E-01	6.74E-01
P41227	NAA10	S186;S205;	1.09E-04	S209;S186;S205;	3.18E-05	S205;S186;S228;S231;	2.37E-04	4.60E-01	1.34E-01
P41236	PPP1R2	S87;S121;S122;S127;	3.23E-03	S122;S121;	1.75E-03	S121;S122;S130;	1.59E-03	2.03E+00	1.10E+00
P42166	TMPO	S66;S67;T74;S168;S184;T160;	3.64E-03	NA	NA	S67;S66;T74;S184;T160;S424;	1.81E-03	2.01E+00	NA
P42167	TMPO	S66;S67;T74;S168;S184;T160;	4.51E-03	S66;S67;T74;Y183;T160;S180;S184;S159;T208;S16	5.39E-03	S66;S67;S306;T160;S180;T74;S184;T211;	1.08E-03	4.16E+00	4.97E+00

				8;S158;					
P42345	MTOR	T1252;S1166;	2.54E-03	NA	NA	T1162;S1166;	3.90E-03	6.52E-01	NA
P42696	RBM34	S14;	3.42E-05	S14;S28;	1.78E-04	S14;	2.61E-04	1.31E-01	6.81E-01
P43243	MATR3	S596;S598;S604;S766;S188;Y597;S195;S620;	1.57E-03	S598;S604;S195;S208;S9;S188;Y597;	1.19E-03	S188;S604;S598;S208;S631;Y597;S195;S9;S620;	1.63E-03	9.66E-01	7.28E-01
P43487	RANBP1	T18;S21;	6.01E-05	T18;T13;S14;S60;	7.50E-05	T15;T18;T7;S21;S60;	5.10E-04	1.18E-01	1.47E-01
P45973	CBX5	S14;S12;S13;	1.16E-04	NA	NA	NA	NA	NA	NA
P46013	MKI67	S1861;S2828;S308;S2344;S357;S1376;S859;S1131;S2223;S2588;S2105;T2502;S2471;S1983;S579;S584;S2827;	1.54E-03	S308;S357;S2223;S2344;S1131;S859;T2231;S2588;S374;S579;S584;S1071;	2.77E-04	S308;S357;S1131;S3197;T1355;T1359;	9.42E-05	1.63E+01	2.94E+00
P46060	RANGAP1	S428;S442;	1.28E-04	S428;S442;	2.14E-04	S428;S442;S427;	2.50E-04	5.13E-01	8.55E-01
P46087	NOP2	S732;S67;S181;T185;S786;	4.92E-04	S732;T736;S67;T605;S786;	6.63E-04	S732;S67;S58;S786;S181;T185;T195;T739;	6.76E-04	7.27E-01	9.80E-01
P46100	ATRX	S677;S1348;S1352;S92;S849;S850;S784;T977;S675;S1073;S1076;S876;Y89;S729;S731;S974;S978;	3.29E-04	S677;S675;S974;T977;S978;T674;S1073;S1076;S1061;S596;S1244;S1245;S1077;S849;S850;S875;S876;S1352;	1.25E-04	S677;S849;S850;S1352;S675;S598;T1230;S1236;S594;S1073;S1076;	1.02E-04	3.23E+00	1.23E+00
P46379	BAG6	Y1116;S113;S964;T978;S973;	1.00E-03	S113;	1.23E-03	T117;S1117;S113;Y1116;	7.64E-04	1.31E+00	1.61E+00
P46821	MAP1B	S1389;S1915;S1396;S1400;S1501;S1298;S1312;S1819;S2209;S2211;S1779;S1154;S614;S1782;S1443;S1797;S1965;S1438;T1633;T704;T1302;S1801;S1254;S1256;S1262;S1324;T1067;S1322;S1339;S1917;T1788;S1792;S831;S832;	1.01E-03	NA	NA	S1400;S1501;T1067;S1917;S937;S1396;S1298;S1312;S1779;S1782;T1949;S1016;S1915;S891;S1819;S1339;S1254;S1265;S1793;S1797;S1965;S1785;T1788;S2271;S1801;S614;S1852;S831;S832;S1322;T1328;S1276;T1282;S1881;S1252;S1154;S1256;S1260;S1792;S1412;T1334;S561;T163	5.33E-03	1.89E-01	NA

						3;S2209;S2211;S1415;S1818;S1258;S1324;S1326;S1280;S1631;T704;			
P46937	YAP1	S367;S289;S61;S138;S109;T337;	4.05E-04	S138;S61;S109;S131;T141;	2.30E-05	S138;S61;S127;S109;T143;S367;	3.49E-04	1.16E+00	6.57E-02
P47914	RPL29	NA	NA	NA	NA	S158;S31;	1.28E-04	NA	NA
P47974	ZFP36L2	S426;	2.90E-05	NA	NA	NA	NA	NA	NA
P48051	KCNJ6	S212;T202;	4.26E-05	NA	NA	T202;S212;S23;	2.19E-05	1.95E+00	NA
P48634	PRRC2A	S380;S383;S761;S342;S350;S1106;T1112;S1306;S1691;	2.03E-04	S1306;S342;S350;S363;S764;T205;S766;S380;S1092;S1085;S1089;S1004;S1147;S761;S1106;T387;S1410;S932;S383;	6.61E-04	S1306;S342;S350;S363;T610;S1147;S1219;S380;S761;	2.65E-04	7.66E-01	2.50E+00
P48681	NES	S1409;S1418;S680;S471;S1615;S1617;S1618;S465;	3.99E-04	S1409;S1418;S471;S1496;S1498;S1492;S680;S1617;S1618;S768;S459;S352;S1615;S1489;S1502;T315;S320;S323;	1.24E-03	S1409;S1418;S471;S680;	4.35E-05	9.17E+00	2.86E+01
P49006	MARCKSL1	S104;S22;S120;	8.69E-05	S120;T178;S135;S22;T148;S48;S104;S93;S119;S71;	3.95E-03	S22;T178;T148;S104;S93;S120;T122;	1.07E-03	8.14E-02	3.70E+00
P49023	PXN	S106;S85;S126;	4.52E-04	S85;S106;S84;	1.29E-04	S106;S85;S332;	3.18E-04	1.42E+00	4.04E-01
P49207	RPL34	NA	NA	NA	NA	S12;	5.97E-04	NA	NA
P49321	NASP	S451;S244;S726;S751;T477;S480;S421;	1.10E-04	T390;S726;	2.09E-05	T390;S726;S451;S244;	4.54E-04	2.42E-01	4.60E-02
P49411	TUFM	T423;	1.33E-04	NA	NA	NA	NA	NA	NA
P49454	CENPF	S821;S3150;S3175;	6.69E-05	NA	NA	S3175;S2996;S3007;S1750;S276;	2.07E-05	3.23E+00	NA
P49585	PCYT1A	S315;S319;S323;S362;S347;T342;S343;S352;	2.72E-04	S343;S347;S362;S339;	8.53E-04	S362;S347;S343;S352;	4.76E-04	5.72E-01	1.79E+00
P49674	CSNK1E	S363;	1.29E-05	NA	NA	NA	NA	NA	NA
P49736	MCM2	S108;S139;S41;S27;Y137;S26;T25;T39;S40;	4.51E-03	S139;S108;S40;S41;T25;S26;S27;T39;Y137;	3.29E-03	S139;T25;S26;S27;S41;S40;	7.34E-03	6.14E-01	4.48E-01
P49756	RBM25	S677;S683;S583;	5.29E-04	S677;	9.15E-04	S677;S683;	6.90E-04	7.67E-01	1.33E+00
P49761	CLK3	S224;S226;S24;	1.12E-05	NA	NA	S224;S226;S197;S199;S283;	1.65E-05	6.76E-01	NA

P49790	NUP153	S334;S338;S192;S687;T691;	1.43E-04	S338;S334;	1.98E-05	S614;S619;S334;S338;S343;S209;S192;S217;	5.78E-05	2.47E+00	3.43E-01
P49792	RANBP2	S2287;S2290;T2293;S2510;S781;S1573;S2900;T1644;S2462;S796;T799;S2276;T1396;S2835;S2741;T2743;S1160;T779;S2280;T2458;S2858;S2831;S2278;	4.19E-04	S2741;T2743;S1509;S1160;T1396;S781;S2454;T779;S2280;S2900;S1456;T783;S1400;	4.29E-04	S2741;T2293;S2668;S2250;S1160;S2510;T19;S21;S955;S2900;S796;T799;S1400;T1396;T2743;T2458;S2462;S2454;T2450;S2278;S2280;S2241;S2251;S18;S2457;S2246;S1573;	9.13E-04	4.59E-01	4.71E-01
P49959	MRE11A	S688;S689;	2.41E-04	S649;	2.42E-05	S688;S689;	1.80E-05	1.34E+01	1.34E+00
P50219	MNX1	S77;S79;	2.98E-05	NA	NA	NA	NA	NA	NA
P50402	EMD	S49;S57;S173;S54;	1.59E-04	NA	NA	S54;S142;S143;S120;T122;S123;S141;	7.30E-05	2.17E+00	NA
P50479	PDLIM4	S112;T124;T115;S116;S120;	1.79E-04	NA	NA	NA	NA	NA	NA
P50502	ST13	S75;S76;S79;	7.02E-05	S75;S76;S79;	2.33E-04	S75;S76;S79;	1.72E-04	4.08E-01	1.36E+00
P50579	METAP2	NA	NA	NA	NA	S74;	1.61E-05	NA	NA
P50747	HLCS	NA	NA	NA	NA	S79;	1.58E-05	NA	NA
P50914	RPL14	S139;	2.28E-04	S139;	1.44E-04	S139;	4.35E-04	5.24E-01	3.31E-01
P51003	PAPOLA	S660;	2.03E-05	T544;S628;S629;S635;T640;S654;S660;	4.38E-05	S660;T652;	6.70E-06	3.04E+00	6.53E+00
P51116	FXR2	T411;S601;S603;	2.19E-04	NA	NA	S453;S601;S603;T411;S533;	3.51E-04	6.25E-01	NA
P51397	DAP	S51;	4.46E-05	T56;S49;S51;	3.28E-04	S49;S51;	1.99E-04	2.24E-01	1.65E+00
P51532	SMARCA4	S1570;S1575;S1586;S1421;S1617;S1620;T859;Y860;Y862;S1452;	1.18E-04	S1452;S1570;S1575;S1586;S1382;T1423;S695;	7.30E-05	T1423;S1570;S1575;S1586;S1452;T1425;S1417;S1421;S695;	7.59E-05	1.56E+00	9.62E-01
P51608	MECP2	S80;S229;	1.32E-04	NA	NA	S80;	6.21E-05	2.13E+00	NA
P51610	HCFC1	S1507;S598;S1205;S666;	2.79E-04	S1507;S666;	8.01E-05	S1507;S666;S1205;S669;T413;	3.75E-04	7.44E-01	2.14E-01
P51858	HDGF	S132;S133;S165;T200;S206;S202;	1.36E-02	S132;S133;S165;S202;S206;	1.83E-02	S132;S133;S165;S107;T200;S206;S202;	1.69E-02	8.09E-01	1.09E+00
P52272	HNRNPM	S29;S575;S701;S513;S446;	4.65E-05	NA	NA	S701;	2.25E-05	2.06E+00	NA
P52292	KPNA2	S62;	4.65E-04	S62;	2.02E-04	NA	NA	NA	NA
P52594	AGFG1	S181;	1.65E-04	S181;S293;	2.08E-04	S181;	2.30E-04	7.19E-01	9.06E-01

P52701	MSH6	S252;S261;S254;S227;S65;	1.27E-04	T139;S227;S41;S252;S256;S261;S65;S63;S43;Y478;	3.66E-05	S252;S254;S261;S14;S65;S830;S256;S227;S137;	2.02E-04	6.29E-01	1.81E-01
P52756	RBM5	S624;S621;S59;	7.03E-05	S621;S624;	5.47E-05	S621;S624;	2.61E-04	2.70E-01	2.10E-01
P52948	NUP98	S623;S888;S1023;S1028;	1.10E-03	S623;S888;S1023;S612;S1028;S608;	6.90E-04	S623;S888;S1028;S1023;S612;	1.48E-03	7.48E-01	4.67E-01
P53396	ACLY	S481;S455;	8.03E-05	S455;	5.12E-04	S455;	2.64E-04	3.04E-01	1.94E+00
P53999	SUB1	S118;S17;	1.78E-05	S118;	1.05E-05	S118;S12;	1.80E-05	9.87E-01	5.82E-01
P54105	CLNS1A	S102;	6.80E-03	S102;	4.04E-03	S102;	1.07E-02	6.38E-01	3.79E-01
P54259	ATN1	S77;S34;S103;S689;T653;S661;T669;S107;S746;S748;Y734;T736;	1.93E-04	NA	NA	S101;S107;S103;S77;S79;S34;S14;	4.85E-05	3.98E+00	NA
P54725	RAD23A	S133;S205;T94;S123;	3.15E-04	S123;	5.40E-04	S123;S133;S136;S140;T131;S205;	1.10E-03	2.87E-01	4.91E-01
P54727	RAD23B	S160;	8.38E-04	S160;T155;T159;	1.75E-03	S160;	1.56E-03	5.37E-01	1.12E+00
P55010	EIF5	S389;S390;	3.19E-04	S389;S390;	1.42E-03	S389;S390;S419;	5.88E-04	5.43E-01	2.41E+00
P55081	MFAP1	S52;S53;S132;S133;S116;T267;S118;	2.02E-03	S116;S118;T267;S132;S133;	1.63E-03	S116;S118;T267;S132;S133;S52;S53;	1.56E-03	1.30E+00	1.05E+00
P55196	MLLT4	S1182;S1779;	9.89E-05	S1182;Y1132;S1173;T1207;S1799;S1275;S1779;S1140;S1143;S1721;S1172;	7.98E-04	NA	NA	NA	NA
P55327	TPD52	S223;S176;S171;	5.17E-04	S223;T173;S176;	5.60E-04	S223;T173;S171;S176;	1.59E-04	3.25E+00	3.52E+00
P55884	EIF3B	S154;S83;S164;S152;	4.83E-04	S152;S154;S164;S78;S125;S83;S81;S117;	1.57E-03	S154;S164;S83;S85;S152;S125;	1.63E-03	2.96E-01	9.63E-01
P56945	BCAR1	S139;S355;S428;T432;S434;	6.40E-05	S355;	2.47E-05	S139;S355;	3.14E-05	2.04E+00	7.86E-01
P57682	KLF3	S250;S80;	1.50E-05	S250;S71;	9.92E-06	NA	NA	NA	NA
P58107	EPPK1	S2716;S2508;	6.88E-04	NA	NA	S2716;S2508;S1529;	2.51E-03	2.75E-01	NA
P61978	HNRNPK	S284;S379;	1.10E-03	S379;T118;S284;	7.26E-05	S379;S216;Y135;S284;	3.11E-04	3.55E+00	2.33E-01
P62070	RRAS2	NA	NA	S186;	2.53E-04	S186;	3.87E-04	NA	6.52E-01
P62263	RPS14	S139;	5.79E-05	T140;S139;	3.94E-05	S139;	7.96E-05	7.27E-01	4.95E-01
P62753	RPS6	S240;S242;S247;S235;S236;S244;	6.98E-04	S235;S236;S240;T241;S244;	6.26E-03	S240;T241;S235;S236;S246;S242;S244;S247;	4.91E-03	1.42E-01	1.28E+00
P62995	TRA2B	S95;S97;S99;S264;S266;T33;S37;T201;T69;S71;S73;S280;S284;S102;S81;S83;S8	5.09E-03	S29;T33;S264;S266;S280;S284;S95;S97;S99;S39;S85;Y86;S87;S81;S83;S37;T	5.29E-03	S264;S266;S280;Y283;S284;S83;Y86;S87;T33;S37;S39;S95;S97;S99;	4.96E-03	1.03E+00	1.07E+00

		5;S39;S282;S29;Y86;S87;S22;		69;S71;S26;		S29;S85;T69;S71;S73;S81;			
P63208	SKP1	T131;	1.07E-05	T131;	1.01E-05	T131;	1.76E-05	6.09E-01	5.72E-01
P67809	YBX1	S174;S176;S314;S165;S167;S102;	1.67E-03	S165;S176;S314;S167;S174;	1.70E-03	S165;S176;S314;S174;	2.90E-03	5.75E-01	5.87E-01
P78310	CXADR	NA	NA	S332;	9.32E-05	S332;	5.64E-05	NA	1.65E+00
P78317	RNF4	S94;S95;	5.50E-05	S94;S95;	6.27E-05	S94;S95;	1.12E-04	4.90E-01	5.58E-01
P78332	RBM6	S891;S1022;S912;S360;S362;Y914;T581;S582;	2.50E-04	S360;S891;Y914;S1025;	8.50E-05	S360;S362;S891;Y914;S1025;T923;	2.57E-04	9.73E-01	3.31E-01
P78347	GTF2I	S210;S823;	6.44E-04	S823;S210;S831;S668;T556;	1.18E-04	S210;S820;	8.43E-05	7.64E+00	1.40E+00
P78362	SRPK2	NA	NA	S497;S494;	6.73E-05	S497;S494;	5.61E-05	NA	1.20E+00
P78368	CSNK1G2	S36;S37;	7.13E-04	NA	NA	NA	NA	NA	NA
P78524	ST5	T364;S368;	5.40E-06	S1011;S1013;S1015;	1.31E-06	T364;S368;	6.17E-06	8.75E-01	2.12E-01
P78559	MAP1A	S2104;S1654;	2.95E-05	NA	NA	NA	NA	NA	NA
P80723	BASP1	S164;S219;T36;T196;T31;T222;S172;S195;	1.33E-03	NA	NA	T36;S219;S172;S164;T196;T162;T31;S176;T222;S182;S40;	1.55E-03	8.60E-01	NA
P83916	CBX1	S89;S91;	4.35E-04	S89;S91;	2.82E-04	S89;S91;	4.46E-04	9.74E-01	6.33E-01
P84098	RPL19	NA	NA	NA	NA	S189;T194;	4.27E-06	NA	NA
P84103	SRSF3	S148;S108;S150;S152;S138;S140;S120;S122;S124;S126;S128;S130;	1.11E-02	S108;S148;S150;S152;S126;S128;S130;	6.54E-04	S108;S148;S150;S152;S126;S128;S130;S138;S140;S120;S122;S124;	3.66E-03	3.02E+00	1.79E-01
P85037	FOXK1	S420;S441;S445;S213;S223;S239;T245;S257;S101;S299;S416;T247;	5.51E-04	S213;S223;T436;S441;S445;	4.55E-04	S441;S445;T436;S213;S223;S705;S711;T731;	7.55E-04	7.29E-01	6.02E-01
P98082	DAB2	S471;S401;S723;S394;	4.51E-05	NA	NA	S401;	4.73E-05	9.54E-01	NA
P98175	RBM10	S733;S736;S738;S723;	7.40E-04	S736;S738;S89;S733;S723;	3.74E-04	S736;S738;S733;S797;S723;S845;Y732;S89;S207;	1.02E-03	7.29E-01	3.68E-01
Q00341	HDLBP	S31;S944;	3.18E-04	S31;T567;S944;	4.81E-04	S31;S944;	4.01E-04	7.94E-01	1.20E+00
Q00613	HSF1	T367;S368;S303;S363;T369;	1.84E-04	S303;S307;S314;S326;T367;T369;	4.74E-05	S363;T369;S303;S307;	1.39E-04	1.32E+00	3.40E-01
Q00839	HNRNPU	S271;S59;	2.74E-04	S271;S764;S766;	2.62E-04	S271;S59;	5.27E-05	5.21E+00	4.98E+00
Q01082	SPTBN1	S2138;S2165;S2169;S2102;S2161;	2.17E-03	S2138;S2341;S2165;S2169;S2102;	4.59E-03	S2138;S2164;S2169;S2165;S2102;	1.47E-03	1.47E+00	3.12E+00

Q01105	SET	S7;	2.15E-04	NA	NA	S7;	2.08E-04	1.03E+00	NA
Q01130	SRSF2	S206;S208;S212;S187;S189;S191;S119;S121;S26;	5.51E-03	S206;S208;S212;S189;S191;S187;S119;S121;	5.69E-03	S187;S189;S191;S206;S208;S212;S26;S119;S121;S140;S142;	5.46E-03	1.01E+00	1.04E+00
Q01167	FOXK2	S168;S398;S373;S428;	1.20E-04	S398;S373;	5.50E-05	S428;S398;S170;S168;	1.25E-04	9.53E-01	4.39E-01
Q01518	CAP1	S310;T307;S308;	1.82E-03	T307;S308;S310;	1.58E-03	T307;S308;S310;	2.39E-03	7.59E-01	6.62E-01
Q01664	TFAP4	S124;S121;	3.52E-04	NA	NA	S123;S124;	1.65E-04	2.14E+00	NA
Q01780	EXOSC10	T675;T747;	5.97E-05	NA	NA	NA	NA	NA	NA
Q01804	OTUD4	NA	NA	S546;S1023;S1024;	5.38E-05	S1006;S1023;S1024;	3.00E-05	NA	1.80E+00
Q01831	XPC	S883;S884;	8.77E-05	S883;S884;	4.06E-04	S883;S884;S397;S398;S399;	4.22E-04	2.08E-01	9.62E-01
Q02040	AKAP17A	NA	NA	S537;S685;S638;S640;	7.83E-05	S537;	2.05E-04	NA	3.82E-01
Q02241	KIF23	S867;S902;	2.76E-05	NA	NA	NA	NA	NA	NA
Q02447	SP3	S73;	3.80E-05	NA	NA	S73;	2.93E-05	1.30E+00	NA
Q02880	TOP2B	S1400;S1413;S1424;S1522;S1524;S1375;S1550;S1552;S1581;T1403;S1336;S1344;S1466;	4.21E-04	S1400;S1413;S1424;S1522;S1524;S1375;S1466;S1581;S1526;T1575;S1550;S1552;T1403;S1461;	1.49E-03	S1400;S1424;S1522;S1524;S1375;S1413;S1581;T1403;S1526;S1466;T1575;S1550;S1552;Y1609;S1336;S1340;S1344;S1457;S1461;	1.75E-03	2.41E-01	8.53E-01
Q03111	MLLT1	S292;S475;S296;S267;	3.06E-05	S267;S359;S361;S475;	1.94E-05	S292;S475;	1.54E-05	1.99E+00	1.26E+00
Q03164	KMT2A	S3036;T3510;T3038;S3527;	5.49E-05	S3036;S2691;S2196;S2201;	1.55E-05	S1837;T1845;S3036;S2391;S2392;S523;	2.09E-05	2.63E+00	7.42E-01
Q03252	LMNB2	S405;S407;T34;S37;S421;S409;S410;	2.72E-03	S405;S407;T34;S37;S424;T39;S426;S421;S420;S409;S410;	1.69E-03	S405;S407;S419;S420;T34;S37;T39;	1.78E-03	1.53E+00	9.53E-01
Q03468	ERCC6	S158;	2.50E-05	NA	NA	S429;S430;S158;	5.83E-05	4.29E-01	NA
Q04323	UBXN1	S200;S199;	6.55E-04	S200;S199;	3.13E-04	S200;S199;	4.30E-04	1.53E+00	7.28E-01
Q04637	EIF4G1	S1187;T1211;S1231;S1209;T207;S1596;	3.32E-04	S1187;S1596;S1209;S204;T205;S1231;S1185;S1194;T1211;	8.53E-04	S1092;S1231;S1194;S1185;T1211;S1209;T207;S1187;	5.43E-04	6.11E-01	1.57E+00
Q04695	KRT17	S32;S12;S13;	1.71E-04	NA	NA	S39;S13;S32;T27;S28;	9.78E-04	1.75E-01	NA
Q04726	TLE3	S245;S203;S286;Y238;	1.91E-04	NA	NA	S286;S203;Y238;S245;T259;S240;S263;S267;	5.86E-04	3.26E-01	NA
Q05193	DNM1	S817;T776;T780;S774;S778;	4.45E-05	NA	NA	NA	NA	NA	NA

Q05209	PTPN12	S603;S606;S673;S571;T569;S588;S608;	1.19E-04	T569;S571;S603;S606;S673;T578;S588;S449;	3.02E-04	S603;S608;S673;S571;S514;S606;T569;S588;T509;T519;S435;S449;	2.15E-04	5.53E-01	1.40E+00
Q05519	SRSF11	S434;S483;S207;S456;S449;S360;T325;S414;S366;S368;S370;	2.77E-03	S434;S207;S456;S366;S368;S370;	2.72E-04	S207;S434;S449;T325;S456;S414;S360;S366;S368;S370;S412;S323;S262;S295;S297;	1.46E-03	1.90E+00	1.87E-01
Q05682	CALD1	S759;S789;	9.49E-05	S765;S789;	2.34E-05	S759;S724;S789;	9.80E-05	9.69E-01	2.38E-01
Q06265	EXOSC9	NA	NA	NA	NA	S306;	1.16E-04	NA	NA
Q07157	TJP1	S125;S968;Y132;S622;S175;S912;S313;S315;S320;S166;S168;S171;S933;T965;S178;S617;S160;S162;	7.39E-04	S125;S131;S175;S178;S1617;S617;S912;S329;S334;S968;S160;S162;	6.29E-04	S125;S968;S617;S1617;S175;S178;S179;S329;S277;Y132;S131;S912;	7.18E-04	1.03E+00	8.77E-01
Q07666	KHDRBS1	S20;S58;	5.11E-04	S58;T61;	7.76E-04	NA	NA	NA	NA
Q07866	KLC1	NA	NA	NA	NA	S460;S524;S521;	1.20E-04	NA	NA
Q07889	SOS1	S1082;S1210;	1.69E-05	NA	NA	NA	NA	NA	NA
Q07955	SRSF1	S234;S238;S199;S201;S205;S223;S227;S231;Y237;S242;Y202;S225;	5.83E-03	S234;S238;S199;S223;S227;S201;Y237;S205;	6.02E-03	S199;Y237;S238;S223;Y226;S227;S201;S205;S234;Y202;	5.01E-03	1.16E+00	1.20E+00
Q08117	AES	NA	NA	NA	NA	S196;	1.65E-06	NA	NA
Q08170	SRSF4	S431;S444;S448;S458;S269;S267;S186;S188;S190;S446;S450;S290;S304;S322;	9.34E-05	S267;S269;S446;S448;S290;S464;S466;S444;	1.60E-05	S431;S444;S446;S448;S269;S456;S450;S458;S460;S267;S184;S186;S188;	4.86E-05	1.92E+00	3.29E-01
Q08945	SSRP1	S444;S667;S671;	1.45E-03	S444;Y452;	1.12E-04	S671;S444;S659;S667;S668;S673;T642;	4.81E-04	3.02E+00	2.33E-01
Q08AD1	CAMSAP2	S599;S1021;	8.32E-05	S599;	1.43E-05	S1148;S599;S1019;S673;	9.39E-05	8.86E-01	1.52E-01
Q08J23	NSUN2	S743;S751;S456;	8.83E-04	S743;S751;S456;	2.26E-03	S743;S751;S456;S473;	7.64E-03	1.16E-01	2.95E-01
Q09161	NCBP1	NA	NA	S22;	1.13E-05	S22;T21;S7;	1.39E-03	NA	8.15E-03
Q09666	AHNAK	S41;S210;S216;S5731;S511;S5763;S5841;S135;S5552;T5798;S5863;S5110;S5752;S5780;S5782;S5793;S93;S4908;S177;S4986;S5077;S115;T5839;S3054;S5784;S5864;S5867;S1042;S34	7.72E-03	S216;S210;S212;S5731;S5752;S5782;T5839;S5077;S1088;S177;	2.13E-04	S115;S5752;S5763;S210;S216;S135;S5731;S5749;T4430;S5552;S41;S1042;T5839;S93;T4100;S5780;S4908;S5841;S3412;S5830;S5782;S5077;S177;S4360;T	1.43E-02	5.41E-01	1.49E-02

		26;S5762;S559;				5794;S5110;S5790;S793;S1170;S3054;S4220;S4993;S5762;S5400;S5784;S5332;S220;T5798;S1298;S212;T4766;S3426;			
Q0JRZ9	FCHO2	S403;S394;S533;T570;S579;	7.31E-05	S488;S496;S403;S533;	1.06E-04	S488;S403;S496;S478;	1.97E-04	3.72E-01	5.39E-01
Q0ZGT2	NEXN	S80;	1.74E-04	S665;T670;S673;T666;	1.66E-05	NA	NA	NA	NA
Q12766	HMGXB3	NA	NA	NA	NA	S226;S227;S1500;	3.34E-04	NA	NA
Q12789	GTF3C1	S1062;S1068;S1856;S1063;	3.42E-05	S1068;S1856;	2.77E-05	S1856;S1062;S1068;S739;	1.95E-04	1.76E-01	1.43E-01
Q12797	ASPH	S29;	2.65E-05	S14;S20;S29;	9.26E-05	S29;	3.53E-05	7.51E-01	2.62E+00
Q12802	AKAP13	S2709;S2728;S983;	4.19E-05	S2709;S983;S1929;T1930;	3.87E-05	S983;S1645;S1647;S345;S2561;S2709;S2728;S403;S395;	9.00E-05	4.66E-01	4.30E-01
Q12834	CDC20	NA	NA	NA	NA	T106;T70;	8.13E-05	NA	NA
Q12872	SFSWAP	S909;S283;S604;S866;S868;S870;S815;	3.49E-05	S866;S868;S815;S283;S870;S604;S872;	6.29E-05	S604;S909;S283;S866;S868;S870;S815;	2.41E-04	1.45E-01	2.62E-01
Q12873	CHD3	S1601;S1549;T715;	9.20E-05	NA	NA	T715;S1219;S1221;S713;S1545;	1.20E-04	7.64E-01	NA
Q12874	SF3A3	S365;S367;S369;	1.96E-05	S365;S367;S369;	3.90E-05	S365;S367;S369;T475;	1.25E-05	1.57E+00	3.13E+00
Q12888	TP53BP1	S500;S1028;S294;S1462;S1114;S727;S482;S552;S525;S1317;S380;S1094;S1101;S1426;S1430;T1056;S831;S398;S862;S265;S1673;S1678;S1481;S316;S1759;S809;T1055;S1362;S523;S222;S1086;	2.99E-03	S1028;S500;S1426;S1430;S380;S1362;S1758;S1759;	1.86E-04	S294;S1028;S500;S1758;S1759;S222;S1618;S1430;S831;S834;S1426;S782;S784;S786;S380;S1481;T1056;S1362;S1068;T1654;S1460;S1701;S1705;S552;S830;T1756;S771;S809;S1462;S1646;S1480;S1673;S1678;S1094;S1101;	2.84E-03	1.05E+00	6.55E-02
Q12906	ILF3	S382;S482;S384;	6.59E-04	S382;S482;	1.77E-04	S482;S382;S384;S812;T486;	1.29E-04	5.10E+00	1.37E+00
Q12996	CSTF3	S691;	1.81E-05	S691;	3.07E-04	S691;S676;	2.13E-04	8.48E-02	1.44E+00
Q13033	STRN3	NA	NA	S229;	1.32E-05	S229;	2.22E-05	NA	5.98E-01

Q13045	FLII	S856;S436;	5.02E-05	NA	NA	S856;S436;	1.18E-04	4.26E-01	NA
Q13085	ACACA	S25;S29;S23;	1.13E-05	S29;S23;S25;	9.50E-05	S29;S23;S25;	1.17E-04	9.66E-02	8.09E-01
Q13111	CHAF1A	S138;S65;S141;T783;S203;S206;	1.11E-04	S775;S206;S65;	6.78E-05	S203;S206;S775;S65;	6.09E-05	1.82E+00	1.11E+00
Q13112	CHAF1B	S429;T432;	1.57E-04	S429;S538;S409;T432;	1.43E-04	S538;S429;T432;S410;	2.73E-04	5.73E-01	5.22E-01
Q13177	PAK2	S141;Y139;	4.53E-05	S141;S58;	4.59E-05	S141;T169;T143;	4.80E-04	9.43E-02	9.56E-02
Q13185	CBX3	S95;S102;S97;S99;S93;S176;	3.59E-03	S97;S99;S95;S176;S93;S102;	1.46E-03	S93;S95;S97;S99;S176;S102;	2.82E-03	1.27E+00	5.18E-01
Q13200	PSMD2	S16;	7.22E-04	S16;S361;	1.21E-03	S16;	2.09E-03	3.45E-01	5.79E-01
Q13206	DDX10	S829;S7;	5.91E-05	S831;S829;	8.87E-05	S831;	2.53E-05	2.34E+00	3.51E+00
Q13233	MAP3K1	S250;S21;	7.18E-06	S21;	1.79E-05	NA	NA	NA	NA
Q13242	SRSF9	S197;S199;S204;S211;S189;S193;S216;S195;	1.26E-03	S211;S216;S204;S195;	1.02E-03	S211;S216;S204;S197;S199;S189;S193;S195;	1.77E-03	7.11E-01	5.78E-01
Q13243	SRSF5	S246;S248;S250;S229;S231;S233;	5.28E-04	S208;S211;Y212;S248;S250;S253;S213;	2.52E-06	S250;S253;S229;S231;S233;	3.51E-04	1.50E+00	7.19E-03
Q13247	SRSF6	S314;S316;S297;S299;S303;S265;S272;S257;S212;S214;S259;S261;S263;	5.92E-03	S314;S316;S303;S295;S297;S301;S299;	4.26E-03	S314;S316;S265;S272;S303;	1.75E-03	3.38E+00	2.43E+00
Q13263	TRIM28	S594;S473;S50;S601;	4.01E-04	S50;S594;S596;S600;S601;S598;S473;	4.90E-04	S752;S50;S600;S757;S594;S697;S473;	1.83E-04	2.19E+00	2.67E+00
Q13283	G3BP1	S232;S149;	1.06E-02	S149;S232;S231;	9.06E-03	S232;S149;S231;	9.88E-03	1.08E+00	9.17E-01
Q13286	CLN3	NA	NA	S12;S14;	7.62E-05	S12;	2.76E-05	NA	2.76E+00
Q13371	PDCL	NA	NA	NA	NA	S296;	5.45E-05	NA	NA
Q13425	SNTB2	S110;S95;	1.81E-04	NA	NA	S231;S393;S110;S95;	1.79E-04	1.01E+00	NA
Q13427	PPIG	S744;S745;T748;S356;T358;S290;S397;S687;S413;S415;S628;S630;S631;T611;S616;S254;S256;S257;S259;	3.08E-04	S256;S257;S259;S413;S415;S356;T358;S716;S717;S397;S290;S744;T748;S687;S745;	1.76E-03	S744;S745;T748;S356;T358;S413;S415;S397;S716;S717;S687;S585;S587;S259;S290;S254;	6.01E-04	5.12E-01	2.92E+00
Q13428	TCOF1	S1378;S381;S1111;S1228;S1190;S1191;S583;S233;S156;S1257;S1350;T914;S503;S906;S375;S1473;	9.26E-03	S1378;S1228;T983;T230;S1350;S381;S156;S1376;S906;T102;S111;S1111;S233;T533;	1.94E-03	S1378;S1111;S583;T533;S233;S156;S381;S1350;S906;S1230;S1190;S1191;T249;S1228;T581;T983;T914;S153;	4.89E-03	1.89E+00	3.97E-01
Q13435	SF3B2	S307;S309;S431;S435;S436;S346;S303;S343;S302;	2.86E-03	S307;S309;S431;S435;S436;S302;T311;	3.91E-03	S307;S309;S431;S435;S436;S346;S302;S303;	1.42E-03	2.02E+00	2.76E+00
Q13439	GOLGA4	S266;S41;S71;	1.87E-04	NA	NA	S266;S41;S71;S89;S92	2.11E-04	8.83E-01	NA

						;			
Q13442	PDAP1	S60;S63;S57;S19;	3.86E-03	S60;S63;S57;	2.04E-03	S60;S63;S57;	1.64E-03	2.35E+00	1.24E+00
Q13459	MYO9B	S1290;S1267;T1271;T460;T1346;	8.62E-05	S1290;S1354;S1267;T1271;S1281;S1992;T2001;S2002;S1115;S1114;S1122;	2.98E-04	S1992;S1290;S1405;	5.10E-05	1.69E+00	5.84E+00
Q13470	TNK1	T91;S502;	1.31E-05	S502;S518;	6.07E-05	NA	NA	NA	NA
Q13501	SQSTM1	S328;S332;S287;S366;T269;S272;T278;S275;	8.59E-04	S266;S272;S275;T269;S366;S24;S276;	6.36E-03	S328;S332;T269;S272;S366;S24;S365;S276;S277;T278;S284;S355;S275;S361;	7.31E-03	1.18E-01	8.70E-01
Q13523	PRPF4B	S20;S23;S32;S142;S144;S427;S431;S437;S354;S356;S277;S328;S366;S368;S578;S580;S410;S411;S292;S294;S394;S396;S349;S239;S241;S518;S519;S520;Y849;S232;T243;S257;S458;S460;S379;S381;T615;	4.89E-03	S518;S519;S520;S578;S580;S354;S356;S277;S427;S431;S437;S366;S368;S232;S383;S410;S411;S239;S241;S451;S453;S458;S460;S292;S294;S394;S396;T615;S622;Y849;T576;S443;S445;S381;S257;T243;	6.40E-03	S518;S519;S520;S32;Y849;S277;S578;S580;S354;S356;S366;S368;S257;T576;S427;S431;S437;S292;S294;S349;S328;S458;S460;S410;S411;S394;S396;S376;S379;S451;S453;S239;	3.50E-03	1.40E+00	1.83E+00
Q13541	EIF4EBP1	T37;S101;S65;T46;T77;S83;T70;T68;	1.47E-03	T37;T41;T46;	6.64E-04	T46;T41;S65;T68;T37;	1.55E-04	9.50E+00	4.29E+00
Q13542	EIF4EBP2	T37;T45;T46;	7.58E-05	T37;T46;	1.17E-04	NA	NA	NA	NA
Q13547	HDAC1	S421;S423;S393;	2.07E-04	S421;S423;	1.35E-03	S421;S423;	8.34E-04	2.48E-01	1.62E+00
Q13563	PKD2	NA	NA	NA	NA	S812;	7.73E-06	NA	NA
Q13573	SNW1	S224;S232;	8.68E-04	NA	NA	NA	NA	NA	NA
Q13586	STIM1	S618;S620;	3.43E-05	S616;S621;S618;S668;	5.73E-05	S618;S553;S556;	1.35E-05	2.54E+00	4.24E+00
Q13595	TRA2A	S84;S86;T88;S96;S98;S71;S73;S75;S260;S262;T202;S100;S16;S20;T22;S274;S278;S276;S18;	4.20E-03	S260;S262;S84;S86;T88;S16;S18;S20;S274;S278;S96;S98;S100;	4.88E-03	S260;S262;S274;Y277;S278;S16;S18;S20;S77;S84;Y87;T88;S86;S96;S98;S100;	4.07E-03	1.03E+00	1.20E+00
Q13596	SNX1	S32;S39;T41;	5.66E-04	S32;S39;T41;	6.85E-04	S32;S39;T41;	4.70E-04	1.20E+00	1.46E+00
Q13610	PWP1	S50;	1.11E-04	S485;	1.01E-05	S50;S485;	1.81E-05	6.11E+00	5.57E-01
Q13620	CUL4B	T52;S146;	3.17E-05	NA	NA	T49;	2.64E-05	1.20E+00	NA
Q13769	THOC5	S312;S314;	2.40E-05	S312;S314;S307;	4.07E-04	S312;S314;S307;	7.09E-05	3.38E-01	5.74E+00
Q13895	BYSL	S98;	1.85E-04	S98;	6.48E-05	S98;	1.48E-04	1.25E+00	4.38E-01
Q14004	CDK13	S337;S340;S352;S437;S439;S383;S315;S317;S328;T4	1.00E-04	S383;S437;S439;S400;T1147;S315;S317;S325;S39	2.12E-04	S315;S317;S325;T1147;S383;S395;S397;S43	2.29E-04	4.38E-01	9.26E-01

		42;S325;S395;S397;		7;Y399;		7;S439;S411;S413;T496;S400;T500;S340;S342;S348;S441;S204;S205;S206;			
Q14103	HNRNPD	S80;S83;S82;	3.31E-03	S83;S80;S82;T193;	3.93E-03	S80;S83;	1.20E-03	2.75E+00	3.26E+00
Q14135	VGLL4	S262;S283;S285;S149;	6.06E-05	S262;	1.22E-05	T151;S262;S52;	5.72E-05	1.06E+00	2.13E-01
Q14137	BOP1	S126;S127;	6.03E-04	S126;S127;	1.13E-03	S126;S127;	5.83E-04	1.03E+00	1.94E+00
Q14151	SAFB2	S343;S330;S331;S832;T244;S513;	1.06E-03	NA	NA	NA	NA	NA	NA
Q14157	UBAP2L	S608;S609;S467;S470;S471;S454;S116;T419;S493;S477;S604;S605;S607;S416;	3.42E-03	S454;S609;S608;S604;S605;S493;T495;Y602;S496;	1.63E-03	S467;S116;S454;S609;S605;S470;S608;S458;S416;S604;S476;	1.52E-03	2.25E+00	1.07E+00
Q14160	SCRIB	S1348;S504;S1566;S1448;S1306;S1309;S1475;S1561;S1300;S1439;	2.17E-04	S504;S1348;S1547;S1566;S1475;S1448;S1220;S1442;	2.35E-04	S1348;S1566;S1475;S504;S1439;S1220;S1448;	2.71E-04	7.98E-01	8.66E-01
Q14161	GIT2	S394;S397;	3.80E-05	NA	NA	S394;T401;S397;Y392;	3.08E-05	1.23E+00	NA
Q14244	MAP7	S365;S209;	1.15E-04	S202;S365;S200;	8.72E-05	S209;S365;	2.27E-04	5.04E-01	3.84E-01
Q14247	CTTN	T401;S405;S418;T399;T411;S417;Y421;	3.07E-03	S418;S426;S11;T399;T411;Y421;T401;S405;S417;S113;T24;S33;S150;	2.89E-02	S405;T411;T399;S417;T401;S418;	6.28E-03	4.89E-01	4.60E+00
Q14258	TRIM25	NA	NA	S100;	5.56E-05	S100;	9.41E-05	NA	5.90E-01
Q14432	PDE3A	S312;S520;	4.59E-05	NA	NA	S523;S524;S520;S528;S1033;T1036;S312;S1030;S408;S527;	3.70E-04	1.24E-01	NA
Q14444	CAPRIN1	S24;S335;	1.36E-04	NA	NA	NA	NA	NA	NA
Q14498	RBM39	S136;S125;S127;S129;S40;S42;S44;	1.86E-03	S136;S125;S127;S129;Y95;S40;S42;S44;	2.13E-03	S136;S97;S125;S127;S129;Y95;	2.68E-03	6.95E-01	7.95E-01
Q14527	HLTF	S397;S398;S400;	2.61E-05	S397;S398;S400;Y394;	3.65E-05	S397;S398;S400;	7.60E-05	3.44E-01	4.80E-01
Q14669	TRIP12	S1317;S1322;S77;S1427;	2.45E-05	S1317;S1322;S159;S1427;S312;S77;S991;S1329;	3.79E-04	S1317;S1322;S1427;S312;S77;S991;Y1015;S1329;	9.78E-05	2.51E-01	3.88E+00
Q14671	PUM1	S229;S709;S247;	2.69E-05	S709;S209;S247;	1.58E-04	S709;	7.52E-05	3.58E-01	2.10E+00
Q14676	MDC1	S168;T449;S453;T1425;S402;T404;S780;S988;S1711;S793;S1820;S1775;S299;T301;T455;T966;S998;S495	1.54E-03	S372;T449;S453;S780;S299;T301;S1775;T455;S168;S1786;	5.94E-04	S780;S299;T301;S1775;T449;S1786;	2.11E-04	7.32E+00	2.82E+00

		;S498;S307;							
Q14677	CLINT1	S227;S232;S234;S299;	2.67E-05	NA	NA	S227;S232;S234;S299;	7.67E-05	3.48E-01	NA
Q14678	KANK1	NA	NA	S325;S879;S881;	1.66E-04	S325;	7.20E-05	NA	2.31E+00
Q14684	RRP1B	S245;T451;S452;S706;S732;S736;S711;S350;S513;T454;S458;	1.41E-04	S732;S735;S513;S245;S392;S736;S706;T728;	1.84E-04	T728;S735;S245;T747;S513;T730;S706;S731;S732;	3.91E-04	3.59E-01	4.70E-01
Q14692	BMS1	S552;	1.97E-05	S552;	2.27E-05	NA	NA	NA	NA
Q14694	USP10	T208;Y791;Y792;S576;S218;	6.51E-05	S576;T82;T208;S218;S226;Y364;S211;	5.64E-04	S576;S218;S215;T208;T216;S547;Y364;S220;S549;	9.20E-04	7.08E-02	6.14E-01
Q14696	MESDC2	S221;S220;	1.35E-05	NA	NA	NA	NA	NA	NA
Q14739	LBR	S82;S84;S86;S97;S99;S71;S67;S73;	2.28E-04	S67;S71;S99;T68;S73;S82;S84;S86;	1.52E-03	S82;S84;S86;	1.26E-04	1.81E+00	1.21E+01
Q14814	MEF2D	S180;S251;S444;	1.21E-04	S251;S180;	9.29E-06	S180;S251;S471;S98;S444;	3.08E-04	3.92E-01	3.02E-02
Q14839	CHD4	T1549;S1535;S103;S515;S531;S105;S108;	5.01E-04	S515;S531;S1535;S103;S105;S108;T529;S1537;	1.67E-04	T1549;S103;S105;S108;S515;S531;T529;S1535;	7.42E-05	6.75E+00	2.25E+00
Q14847	LASP1	S146;	5.41E-04	S146;	1.63E-04	S146;T104;	3.76E-05	1.44E+01	4.33E+00
Q14865	ARID5B	S1032;	2.03E-05	NA	NA	NA	NA	NA	NA
Q14966	ZNF638	S1401;S128;	1.67E-05	S128;	2.20E-05	S488;S490;S492;S605;S128;T125;Y514;Y516;	3.96E-05	4.22E-01	5.57E-01
Q14978	NOLC1	S563;S698;S643;S508;S397;S538;T607;T610;S622;S623;	2.77E-03	S563;S698;S538;S397;T607;T610;S508;S643;	6.71E-04	S563;S538;S397;T607;T610;S698;S643;S508;	4.09E-04	6.78E+00	1.64E+00
Q14980	NUMA1	S169;S1757;T2000;S200;S203;S271;S1862;	2.01E-03	S169;S1853;S1757;	2.01E-04	S169;S1757;S1862;S271;T2000;	8.48E-04	2.37E+00	2.37E-01
Q14C86	GAPVD1	S746;S966;S902;	5.08E-05	S902;S766;S966;	2.04E-04	S746;S1096;S966;S902;S766;T938;S1019;S772;T774;T747;	4.11E-04	1.24E-01	4.96E-01
Q15018	FAM175B	S368;S372;S375;	1.07E-04	S368;S372;S375;	1.95E-04	NA	NA	NA	NA
Q15019	SEPT2	S218;	1.48E-03	S218;	1.90E-03	S218;	4.02E-03	3.69E-01	4.72E-01
Q15036	SNX17	NA	NA	S409;S336;	3.11E-05	S409;S336;	1.10E-04	NA	2.82E-01
Q15054	POLD3	S307;	1.31E-04	NA	NA	NA	NA	NA	NA
Q15061	WDR43	S77;	7.13E-05	S77;	3.40E-04	S431;S77;	6.46E-04	1.10E-01	5.27E-01

Q15084	PDIA6	S428;	3.27E-05	S428;	1.65E-04	S428;	3.74E-05	8.76E-01	4.41E+00
Q15121	PEA15	S116;	1.51E-04	S116;	1.27E-04	S116;	6.82E-05	2.21E+00	1.86E+00
Q15149	PLEC	S4406;S4382;S4385;S4386;S4396;S4618;S1732;S4626;S1435;T723;S4389;S4384;	2.21E-03	S4626;S4386;S4391;S1435;S4389;S4390;T4623;S4622;S4385;	2.03E-04	S4626;S4406;S4618;T4628;S4386;S4390;S4382;S4385;S1435;S1732;S4389;S4622;S4630;S149;S125;S720;T4623;S4384;Y4611;	2.62E-03	8.45E-01	7.76E-02
Q15154	PCM1	S65;S1257;S1260;S1263;S68;S69;S1765;S1768;S1776;S159;T530;S533;S537;S430;S93;Y535;	2.80E-04	S1765;S1768;S1776;S65;S68;S69;S116;S119;T530;S537;Y535;	9.11E-04	S1768;S65;S1765;S1776;S1730;S430;S159;S68;S69;T530;S533;S537;S428;S116;S119;	4.14E-04	6.76E-01	2.20E+00
Q15170	TCEAL1	S36;S37;S41;S42;	2.14E-05	NA	NA	NA	NA	NA	NA
Q15185	PTGES3	S113;S82;S85;S148;S151;	6.30E-03	S113;	2.06E-04	S113;	1.06E-03	5.95E+00	1.94E-01
Q15233	NONO	T450;T428;	1.91E-04	NA	NA	T450;	1.11E-05	1.73E+01	NA
Q15287	RNPS1	S155;S157;S251;S137;S139;S141;S27;S274;S276;	6.66E-04	S155;S157;T161;S137;S139;S141;S274;S276;S251;	1.29E-03	S155;S157;S251;S274;S276;S27;	3.97E-04	1.68E+00	3.24E+00
Q15311	RALBP1	NA	NA	S29;S92;S93;S34;T36;S647;S48;S62;	4.17E-05	S92;S93;S29;S30;S34;	3.65E-05	NA	1.14E+00
Q15365	PCBP1	S190;	2.53E-04	NA	NA	NA	NA	NA	NA
Q15366	PCBP2	S189;S187;S364;	2.48E-04	S189;S187;	2.10E-04	S189;	5.14E-04	4.83E-01	4.09E-01
Q15390	MTFR1	NA	NA	NA	NA	S119;	2.12E-05	NA	NA
Q15417	CNN3	NA	NA	S323;	1.33E-04	NA	NA	NA	NA
Q15424	SAFB	S344;S383;S384;S331;S332;S604;S580;S582;T245;	1.19E-03	S344;S197;S331;S332;S604;S582;T200;S325;T194;S32;	1.66E-03	S325;S344;S331;S332;T194;S235;S604;S197;S383;S384;S234;	9.27E-04	1.28E+00	1.80E+00
Q15435	PPP1R7	S24;S27;	8.43E-05	S24;S27;	6.58E-04	S24;S27;	2.67E-04	3.16E-01	2.47E+00
Q15459	SF3A1	S329;S359;	1.98E-04	S329;	3.53E-05	S329;S451;	9.94E-05	2.00E+00	3.55E-01
Q15477	SKIV2L	S245;S256;	4.71E-05	S256;	7.53E-05	S256;S245;	1.44E-04	3.27E-01	5.23E-01
Q15637	SF1	S80;S82;	1.65E-03	S80;S82;	3.09E-03	S80;S82;	1.36E-03	1.21E+00	2.27E+00
Q15642	TRIP10	S296;T302;S299;	1.37E-04	S296;S298;	8.81E-04	S296;S299;	3.25E-04	4.21E-01	2.71E+00
Q15648	MED1	S1479;S1481;S1156;S1223;S1401;S1207;	1.59E-04	S1479;S1482;S1403;S770;S1481;T391;S774;	3.00E-05	S1481;S1156;S1437;S1479;	1.47E-04	1.09E+00	2.05E-01
Q15651	HMGN3	S78;S6;	1.21E-04	S93;S6;	2.94E-05	S6;S78;T10;	9.32E-06	1.29E+01	3.16E+00
Q15654	TRIP6	S142;	7.10E-05	NA	NA	NA	NA	NA	NA

Q15691	MAPRE1	S155;	5.84E-05	NA	NA	S155;	1.35E-04	4.32E-01	NA
Q15773	MLF2	S238;	3.12E-04	S238;	6.23E-04	S238;	1.48E-04	2.11E+00	4.21E+00
Q15811	ITSN1	S904;	1.17E-04	S203;S904;	7.03E-05	S902;S904;S203;	9.67E-05	1.21E+00	7.27E-01
Q15911	ZFHX3	S1590;S3409;S3418;	1.84E-05	NA	NA	NA	NA	NA	NA
Q15942	ZYX	S281;S267;S308;S344;S290;S278;S259;	1.25E-03	S308;S259;S267;S344;S281;S278;	1.40E-03	S308;S281;S259;S288;S344;S278;S267;T270;S258;	1.98E-03	6.30E-01	7.06E-01
Q16204	CCDC6	S244;S240;S419;S323;S367;	4.24E-04	S240;S244;S367;S419;	5.42E-04	S240;S244;S367;S419;	1.41E-03	3.00E-01	3.84E-01
Q16513	PKN2	S583;	3.87E-05	S583;	4.23E-05	S583;S535;S360;S21;T958;	3.94E-04	9.83E-02	1.07E-01
Q16514	TAF12	NA	NA	S51;	2.13E-05	S51;	1.31E-04	NA	1.62E-01
Q16555	DPYSL2	S518;S522;T512;T509;	6.86E-05	S518;T514;S522;	3.15E-05	T514;S518;S522;T509;T512;	8.55E-05	8.02E-01	3.69E-01
Q16629	SRSF7	S231;S233;S192;S194;S171;S173;S175;S196;S163;S165;S200;S202;S204;S179;S181;S183;S130;S132;S134;S223;S225;S227;S167;	6.05E-03	S179;S181;S183;S192;S194;S173;S175;S196;S231;S233;S171;S223;S225;S227;S155;S157;S159;S165;S167;S163;	3.78E-03	S179;S181;S183;S192;S194;S196;S171;S173;S175;S163;S165;S167;	4.49E-03	1.35E+00	8.42E-01
Q16637	SMN1	S28;S31;	6.33E-04	S28;S31;T25;	1.05E-03	S28;S31;T25;	9.87E-04	6.42E-01	1.06E+00
Q16643	DBN1	T335;S345;S601;S142;S339;S337;	6.44E-04	S601;T346;S142;S342;T343;	1.06E-03	S337;S342;S339;S345;S142;T343;	1.10E-03	5.87E-01	9.64E-01
Q16666	IFI16	S780;S153;S106;	1.76E-04	NA	NA	S106;	1.95E-04	9.06E-01	NA
Q1KMD3	HNRNPUL2	S185;S161;S193;	1.03E-03	S161;T165;S185;S193;	2.30E-03	S185;S161;S193;S188;T165;S168;	1.86E-03	5.53E-01	1.24E+00
Q29RF7	PDS5A	S1305;	1.72E-04	S1195;S1305;	4.05E-04	S1305;	8.40E-05	2.05E+00	4.81E+00
Q2KHR3	QSER1	T1341;T1346;S1348;S1227;S1228;	3.19E-05	T1341;S1348;	2.43E-05	S1230;S1231;T1341;S1348;S1228;	6.02E-05	5.30E-01	4.03E-01
Q2M2I8	AAK1	S670;T674;S678;T620;S623;S668;T606;	1.14E-04	T606;T620;S670;T674;S678;S623;	3.79E-04	S670;T674;S678;S637;T606;T620;S623;	3.06E-04	3.74E-01	1.24E+00
Q2M3G4	SHROOM1	S133;S188;S186;	2.63E-05	S133;S49;	1.85E-05	NA	NA	NA	NA
Q2TBE0	CWF19L2	NA	NA	NA	NA	S479;	1.02E-05	NA	NA
Q32MZ4	LRRFIP1	S735;S16;S714;S66;S68;S733;	2.57E-04	S120;S714;S618;S733;S639;	1.88E-04	S714;T65;S68;S735;S768;S66;S766;	3.35E-04	7.68E-01	5.63E-01
Q3KQU3	MAP7D1	S113;S116;S544;S548;S55	1.45E-04	S113;S116;S123;S544;S5	1.64E-04	S113;S116;T118;S125;	3.90E-04	3.72E-01	4.19E-01

		2;S742;T554;S125;S70;S86;T118;		48;S552;T118;S742;T554;		S544;S548;S552;S460;S70;S86;S742;S399;T554;S446;S123;			
Q3YEC7	RABL6	S596;T599;S425;S427;	8.42E-05	S596;T599;S454;S470;S471;S464;	8.33E-04	S596;T599;S470;T468;S471;	1.40E-03	6.00E-02	5.93E-01
Q4G0J3	LARP7	T257;S258;S261;S298;S299;S337;T338;S300;	2.34E-04	T257;S258;S300;S261;S337;T338;S299;	6.53E-04	T257;S258;S261;S337;T338;S298;S300;S299;	6.96E-04	3.36E-01	9.38E-01
Q4KMP7	TBC1D10B	S678;S316;S322;S707;	7.73E-05	S687;S656;S661;T148;S678;S132;S658;	2.65E-04	S132;S678;S707;	4.77E-05	1.62E+00	5.56E+00
Q4LE39	ARID4B	S790;S838;T793;	1.88E-05	S790;T793;	4.48E-05	S790;T793;	9.33E-05	2.01E-01	4.80E-01
Q4V328	GRIPAP1	NA	NA	NA	NA	S690;S688;S692;	1.14E-04	NA	NA
Q53EL6	PDCD4	S94;S78;T93;	2.29E-04	S94;	2.18E-04	S76;	9.05E-06	2.54E+01	2.41E+01
Q53F19	NCBP3	S30;S500;S25;S209;S210;	4.25E-05	S209;S210;S225;	2.72E-05	S25;S209;S210;S226;S500;	3.85E-05	1.10E+00	7.05E-01
Q53GA4	PHLDA2	NA	NA	NA	NA	S141;S144;	2.00E-04	NA	NA
Q53GS9	USP39	S82;	3.15E-05	S82;	1.36E-04	S82;S46;	1.49E-04	2.11E-01	9.12E-01
Q53H80	AKIRIN2	S57;S31;S39;	2.62E-05	NA	NA	NA	NA	NA	NA
Q53HL2	CDCA8	NA	NA	S219;S215;	1.01E-04	NA	NA	NA	NA
Q53LP3	SOWAHC	S226;S229;S231;	3.56E-05	NA	NA	S226;S513;S229;	1.26E-05	2.83E+00	NA
Q53SF7	COBLL1	S362;S1146;S1023;	4.23E-05	S294;S518;S515;S1023;T298;	1.82E-04	NA	NA	NA	NA
Q56P03	EAPP	S109;S111;	2.63E-05	S109;S111;T121;	2.89E-05	S109;T121;S111;	6.60E-05	3.99E-01	4.38E-01
Q58WW2	DCAF6	S847;S850;T654;S657;S336;	4.18E-05	T654;S657;	5.97E-05	S847;S850;S657;T654;	9.18E-05	4.55E-01	6.50E-01
Q5BKZ1	ZNF326	S270;T272;	1.66E-04	S270;	1.66E-04	S270;	1.11E-04	1.50E+00	1.50E+00
Q5C9Z4	NOM1	S317;S320;S321;S139;	3.50E-05	S317;S320;S321;S280;T287;	1.13E-04	S317;S320;S321;T327;	3.86E-05	9.06E-01	2.92E+00
Q5F1R6	DNAJC21	S370;	1.87E-04	NA	NA	NA	NA	NA	NA
Q5H9R7	PPP6R3	S617;S853;	3.65E-04	T517;S523;S524;S617;S525;S853;	1.90E-03	S617;	7.65E-04	4.78E-01	2.49E+00
Q5HYJ3	FAM76B	NA	NA	NA	NA	S193;	4.15E-05	NA	NA
Q5JSH3	WDR44	S403;S50;	3.52E-04	S403;S561;S50;T163;	1.40E-04	S403;S96;	3.91E-04	9.01E-01	3.58E-01
Q5JSZ5	PRRC2B	S613;S480;	2.82E-05	S613;S1185;	2.62E-05	S388;S1185;S556;	2.39E-05	1.18E+00	1.10E+00
Q5JTD0	TJAP1	S300;S545;	1.76E-05	S300;S545;	9.34E-05	S545;S300;	9.91E-05	1.78E-01	9.43E-01
Q5JTH9	RRP12	S1080;	7.03E-05	S1080;	2.18E-04	S1080;	6.82E-04	1.03E-01	3.19E-01

Q5JTV8	TOR1AIP1	S156;S157;S154;S242;S143;S315;T220;	1.88E-04	S157;S154;S156;S315;T220;S242;S215;	9.42E-04	S154;S156;S157;T220;	2.55E-04	7.38E-01	3.70E+00
Q5M775	SPECC1	S134;S810;S131;T136;	8.47E-05	NA	NA	S131;S356;T357;S360;T140;T142;	2.01E-05	4.22E+00	NA
Q5QJE6	DNTTIP2	S117;S175;S82;T83;S184;S189;	2.37E-05	S117;S88;S82;T83;Y94;	4.59E-05	S117;T83;T131;T129;S184;S175;T79;S82;	5.50E-05	4.30E-01	8.36E-01
Q5SSJ5	HP1BP3	T48;T51;	5.69E-05	S441;S442;S446;T51;T48;	7.15E-05	S441;S442;S446;T51;T48;	8.82E-05	6.45E-01	8.10E-01
Q5SW79	CEP170	S1079;S1529;S838;S971;S928;S930;S933;S630;	8.04E-05	S928;S933;T937;S930;S446;S838;	5.92E-05	S928;S930;S933;S379;T1078;S446;S838;S1529;T632;T945;T948;S1112;S630;S1160;T1533;T937;S939;S1521;S1522;	2.86E-04	2.82E-01	2.07E-01
Q5SXM2	SNAPC4	T625;S1224;S1398;S1400;	2.19E-05	S1398;S1400;S1407;	1.54E-05	S1398;S1400;S1407;	2.73E-05	8.01E-01	5.63E-01
Q5SXM8	DNLZ	NA	NA	NA	NA	S171;	2.01E-05	NA	NA
Q5SYE7	NHSL1	T1235;T1236;S720;S727;S730;S1233;	1.15E-05	S1089;T862;S851;S857;S727;S730;S853;Y856;S723;S1233;	2.04E-04	S1386;S1388;S723;S727;S730;	4.04E-06	2.84E+00	5.05E+01
Q5T1M5	FKBP15	S956;S1114;S1164;S960;S1097;S1100;	3.64E-04	S956;S1164;S311;S1114;S962;	3.75E-04	S1097;S1114;S1100;S311;S1164;S962;S956;S326;S960;	7.60E-04	4.79E-01	4.93E-01
Q5T200	ZC3H13	S1017;S831;S833;S837;S993;S207;S209;T263;S265;S316;T317;S325;S380;S242;S318;S877;S198;S110;S370;S372;S381;S77;S1279;S642;S1208;S845;Y851;S853;S387;S875;S1010;S1014;S848;S1278;	1.18E-03	S1010;S1014;S1017;S993;T263;S265;S198;S370;S372;S381;S325;S875;S877;S209;S211;S387;S207;S831;S833;S837;S77;S1208;S110;S316;T317;	2.01E-03	T263;S265;S207;S209;S877;S1208;S110;S316;T317;S325;S370;S372;S381;S831;S833;S837;S318;T354;S356;S845;S848;S853;S1010;S1014;S1017;S993;S242;S198;Y851;S358;S1210;S387;	5.74E-04	2.05E+00	3.50E+00
Q5T3I0	NA	NA	NA	NA	NA	NA	NA	NA	NA
Q5T4S7	UBR4	NA	NA	S1634;S1647;S1652;T2884;S620;	5.24E-05	S1763;S1760;S364;S457;S362;S620;S178;T360;T1761;	3.68E-04	NA	1.43E-01
Q5T6F2	UBAP2	NA	NA	NA	NA	T476;	7.98E-06	NA	NA
Q5T8D3	ACBD5	S196;S200;S193;	4.76E-05	S196;S200;	5.06E-05	S196;S200;	4.31E-05	1.10E+00	1.18E+00

Q5T8P6	RBM26	S616;S127;S518;S176;S178;S184;S186;S188;	2.81E-04	S616;Y179;S180;S127;S176;S178;	2.85E-04	S616;S127;S184;S186;S188;	1.29E-04	2.18E+00	2.21E+00
Q5TAQ9	DCAF8	NA	NA	NA	NA	S99;S129;S130;	7.23E-05	NA	NA
Q5TCZ1	SH3PXD2A	S547;S318;Y319;	1.74E-05	S547;	2.74E-05	S547;	1.16E-05	1.50E+00	2.36E+00
Q5TGP6	MROH9	NA	NA	NA	NA	S135;S136;Y137;S357;	8.34E-05	NA	NA
Q5UIP0	RIF1	S2144;S1688;S1579;S2161;S2243;S2393;S2172;S2176;S1162;S2196;	2.59E-04	S2144;S1688;S1579;S2196;S1542;S1613;S2231;S2393;	6.07E-04	S2144;S1579;S1688;S1613;S782;S2393;	1.11E-04	2.33E+00	5.46E+00
Q5VT52	RPRD2	S593;S374;S928;S932;S614;S665;T732;S476;S479;	5.33E-04	S593;S614;S599;S596;T482;	9.93E-05	S614;S593;S925;S932;T598;T484;S976;T723;S928;S930;S491;S758;	2.32E-04	2.30E+00	4.29E-01
Q5VTL8	PRPF38B	S527;S529;S471;S473;S475;S268;S260;S262;S266;	1.70E-03	S527;S529;S266;S268;S473;T479;S481;S475;S260;S262;S471;	6.32E-04	S527;S529;S471;S473;S475;S481;S268;T479;S260;S262;	2.84E-04	5.98E+00	2.23E+00
Q5VTR2	RNF20	S524;S138;T31;	2.39E-04	S524;T526;S522;S138;	2.02E-04	S138;S524;S136;S652;	2.63E-04	9.10E-01	7.69E-01
Q5VUA4	ZNF318	S40;S305;S307;S79;S81;S69;S71;	1.16E-03	S214;S79;S81;S1717;S40;S2189;S2192;S305;S307;S207;S89;S91;S2030;S2035;	5.41E-04	NA	NA	NA	NA
Q5VZ89	DENND4C	S737;S810;S863;S1042;	3.36E-05	S1042;S732;S737;S810;	1.00E-04	S737;S753;S863;S1042;S732;S1089;S810;	9.27E-05	3.62E-01	1.08E+00
Q5VZK9	LRRC16A	S1288;S1331;S1291;S1151;S1148;S1150;S1315;S1290;S1319;S1094;	4.98E-04	S1288;S1291;	2.21E-05	S1290;S1315;S1319;S1288;S1357;S1291;S1151;	2.35E-04	2.12E+00	9.39E-02
Q5VZL5	ZMYM4	T118;S122;	5.61E-05	S122;T118;S1542;S1547;	4.05E-05	S122;S1241;S1542;S1547;T118;S1539;	1.85E-04	3.03E-01	2.19E-01
Q5ZPR3	CD276	S525;	4.96E-05	S525;	4.83E-05	NA	NA	NA	NA
Q63ZY3	KANK2	S323;	1.94E-05	NA	NA	NA	NA	NA	NA
Q63ZY6	NA	NA	NA	NA	NA	NA	NA	NA	NA
Q641Q2	FAM21A	S498;S333;S619;S620;T331;	2.39E-04	S158;S160;S498;S539;S619;S620;S333;S56;S356;S352;	3.37E-04	S619;S620;S498;S352;T331;S333;S158;S160;S1087;S1091;S441;S539;S284;	5.10E-04	4.69E-01	6.62E-01
Q659C4	LARP1B	S60;	1.12E-05	S432;S436;S60;S340;S343;	6.81E-05	S60;S906;S432;S436;S869;	3.67E-05	3.06E-01	1.86E+00
Q66PJ3	ARL6IP4	S332;	5.80E-04	S332;	7.58E-04	S332;	8.96E-04	6.47E-01	8.45E-01

Q68CZ2	TNS3	S776;S660;S850;	2.60E-05	S690;S660;S1154;S1115;S1123;Y780;S332;T692;S1149;	3.06E-04	S690;Y780;S660;S332;	1.28E-04	2.03E-01	2.39E+00
Q68EM7	ARHGAP17	S625;S575;S667;	7.43E-04	S575;S674;S676;	1.27E-04	S575;S674;S676;S667;	5.03E-04	1.48E+00	2.53E-01
Q69YN4	KIAA1429	S133;S138;S173;	4.02E-05	S133;S1579;S173;S1578;S138;	2.53E-04	S138;S173;S133;S1579;T184;	1.97E-04	2.04E-01	1.29E+00
Q6FI81	CIAPIN1	S183;	1.41E-04	S183;	8.01E-05	S183;S305;	6.04E-05	2.33E+00	1.33E+00
Q6FIF0	ZFAND6	S138;	5.88E-05	NA	NA	NA	NA	NA	NA
Q6IQ23	PLEKHA7	NA	NA	S903;S907;S463;T472;S476;	2.49E-04	NA	NA	NA	NA
Q6IQ49	SDE2	S278;	5.82E-05	T274;T314;T316;	2.20E-05	NA	NA	NA	NA
Q6KC79	NIPBL	S256;S280;S284;S306;S850;S2658;	9.82E-05	S2658;S274;S280;S284;S306;S2493;S2498;	1.70E-04	S1089;S1090;S1096;S2658;S280;S306;S2672;S284;S318;	3.01E-04	3.26E-01	5.66E-01
Q6MZP7	LIN54	S310;S314;	2.13E-05	NA	NA	NA	NA	NA	NA
Q6NT89	TRNP1	S78;S90;	7.11E-05	NA	NA	NA	NA	NA	NA
Q6NYC8	PPP1R18	S490;S224;S530;	7.16E-05	NA	NA	S224;S139;	6.02E-05	1.19E+00	NA
Q6NZI2	PTRF	S202;S203;S387;S389;S366;	2.44E-04	NA	NA	NA	NA	NA	NA
Q6NZY4	ZCCHC8	S427;S598;T479;	3.05E-05	S598;S424;S601;	1.26E-05	S598;S427;T479;T485;S424;	3.23E-05	9.43E-01	3.91E-01
Q6P158	DHX57	S132;S127;S36;	1.86E-04	S132;	7.23E-05	S132;S127;	1.66E-04	1.12E+00	4.35E-01
Q6P1L5	FAM117B	T111;S116;S106;S273;	2.99E-05	S449;S106;S273;S136;T162;	4.10E-05	S152;S417;S136;	3.88E-05	7.70E-01	1.06E+00
Q6P2E9	EDC4	S708;S725;S729;S875;S879;S844;	1.04E-04	S708;S725;S729;S844;S875;S871;S741;S887;S890;	4.55E-04	S708;S725;S875;S879;S705;	4.44E-04	2.34E-01	1.02E+00
Q6P582	MZT2A	S152;	2.94E-05	NA	NA	NA	NA	NA	NA
Q6P5R6	RPL22L1	S118;S120;	2.00E-04	S118;	3.69E-05	S118;	1.17E-04	1.71E+00	3.16E-01
Q6P6C2	ALKBH5	S64;S69;	2.89E-05	S371;S64;Y71;S69;S361;	1.17E-04	S64;S69;S384;S361;Y367;	1.46E-04	1.98E-01	8.05E-01
Q6PD62	CTR9	S941;S943;S970;S1081;S1085;S1087;T925;S1041;S1037;S1043;S1039;	3.54E-04	T925;S970;S941;S943;S1081;S1085;S1087;S1041;S1043;S1037;	4.58E-04	S941;S943;T925;S970;S1039;S1041;S1043;S1081;S1085;S1087;S1091;	1.66E-03	2.13E-01	2.76E-01
Q6PJG2	ELMSAN	S700;T704;S923;T655;S66	5.61E-05	T655;S661;S648;S923;	2.00E-05	T655;S661;S461;S996;	9.06E-06	6.19E+00	2.21E+00

	1	1;S461;				S991;			
Q6PJT7	ZC3H14	S515;S409;S620;S132;S135;	4.03E-04	S515;S409;	3.56E-04	S515;S409;S132;S135;S620;	4.17E-04	9.68E-01	8.54E-01
Q6PKG0	LARP1	S75;S90;S627;S631;S225;S228;S143;T526;S220;S774;S521;S517;T223;S548;S217;	2.15E-03	S90;S75;S548;Y633;S627;S631;S849;T856;T526;S766;S774;S517;S521;T68;S143;S228;T788;	3.09E-03	S627;S631;S548;S853;S225;S228;S143;T526;S774;S766;S517;S521;S217;S847;S849;T622;S220;	1.83E-03	1.18E+00	1.69E+00
Q6QNY0	BLOC1S3	T63;S65;	3.33E-04	T63;S65;	4.94E-04	T63;S65;	2.73E-04	1.22E+00	1.81E+00
Q6SPF0	SAMD1	S161;T107;	8.78E-05	S161;	3.23E-05	S161;	5.36E-05	1.64E+00	6.02E-01
Q6T4R5	NHS	S1329;S415;	3.14E-05	S504;S506;S855;	6.37E-05	S1329;	5.31E-05	5.92E-01	1.20E+00
Q6UN15	FIP1L1	S492;S500;S554;T494;S496;S304;S85;S87;S89;	2.74E-04	S85;S87;S89;S492;S554;T494;S500;S259;S304;S62;T68;S496;	1.23E-03	S85;S87;S89;S554;S492;S500;T68;S496;S259;	9.32E-04	2.94E-01	1.32E+00
Q6UUV7	CRTC3	NA	NA	NA	NA	S329;S443;	1.26E-05	NA	NA
Q6UX04	CWC27	S299;	1.57E-05	NA	NA	NA	NA	NA	NA
Q6VMQ6	ATF7IP	S474;S559;S113;T860;S862;S852;	2.70E-04	S113;	3.86E-05	S113;T118;S673;	9.56E-05	2.83E+00	4.03E-01
Q6VN20	RANBP10	NA	NA	NA	NA	S365;S369;	3.40E-05	NA	NA
Q6WCQ1	MPRIP	S217;S224;S289;S294;	3.04E-05	S220;S224;	3.04E-05	S220;S224;S365;S993;S362;S292;S372;	1.77E-04	1.71E-01	1.71E-01
Q6WKZ4	RAB11FIP1	S202;S341;S345;S343;S342;S199;S545;	3.46E-04	S202;S529;S199;S339;S343;S345;S206;S477;	2.14E-04	S206;S343;S345;S202;S341;S357;S545;	1.99E-04	1.74E+00	1.08E+00
Q6Y7W6	GIGYF2	S26;S30;S201;S160;T382;	2.57E-04	S26;S201;S30;S160;S19;S23;T382;	3.20E-04	T382;T25;S26;S160;S392;S30;S201;S236;	4.67E-04	5.51E-01	6.85E-01
Q6ZN18	AEBP2	S206;S139;S141;	1.22E-04	NA	NA	NA	NA	NA	NA
Q6ZNJ1	NBEAL2	NA	NA	S757;S2739;T651;T658;	7.29E-05	S757;T1879;S2739;S2742;	5.86E-05	NA	1.24E+00
Q6ZRS2	SRCAP	S2790;S3177;T2425;S2430;S3148;S274;S1859;	6.26E-05	S3148;S2790;	2.03E-05	S3148;S808;T812;Y821;	1.09E-05	5.73E+00	1.86E+00
Q6ZRV2	FAM83H	S870;S914;S892;S881;	1.02E-04	S870;S523;S881;S924;S892;	7.79E-05	S892;S870;S881;S914;	6.89E-05	1.48E+00	1.13E+00
Q6ZS17	FAM65A	S351;	2.57E-05	S351;	3.38E-05	S351;T728;S732;	1.21E-04	2.13E-01	2.80E-01
Q6ZW31	SYDE1	NA	NA	NA	NA	S681;S683;S231;S235;S244;	7.85E-05	NA	NA
Q70E73	RAPH1	S1154;T1153;	2.16E-05	NA	NA	NA	NA	NA	NA

Q71RC2	LARP4	S583;S722;T595;S647;	4.42E-04	S722;S583;T649;S597;	2.94E-04	S583;S722;S594;T649;	8.26E-04	5.36E-01	3.56E-01
Q75WM6	H1FNT	S236;S237;	4.09E-05	NA	NA	NA	NA	NA	NA
Q76FK4	NOL8	S1082;S1083;S1084;T888;S890;S365;S660;S662;S1099;S298;T302;S304;	5.31E-04	S1082;S1083;S1084;T888;S890;S298;T302;S304;	2.67E-04	S1082;S1083;S1084;T888;S890;S660;S268;S1099;	2.81E-04	1.89E+00	9.50E-01
Q7KZ85	SUPT6H	S73;S75;S78;S125;	5.71E-05	S73;S75;S78;	3.64E-04	S73;S75;S78;S125;	4.76E-04	1.20E-01	7.63E-01
Q7L014	DDX46	S804;	2.46E-05	S24;S28;S804;	1.29E-05	S804;S24;	1.30E-05	1.89E+00	9.91E-01
Q7L0X2	ERICH6	S189;	1.37E-06	NA	NA	NA	NA	NA	NA
Q7L1Q6	BZW1	S411;S413;	9.03E-05	S411;S413;	5.08E-04	S411;S413;	4.78E-04	1.89E-01	1.06E+00
Q7L2J0	MEPCE	S60;S69;S216;S217;S57;S101;	4.00E-04	S152;S330;S57;S60;S216;T213;S217;T245;S334;	2.72E-04	S330;S69;S216;S57;S152;T213;S60;S217;S334;S254;	2.57E-04	1.56E+00	1.06E+00
Q7L4I2	RSRC2	S17;S104;S32;S30;	3.01E-04	S30;S32;S27;	3.90E-05	S17;S32;S27;	5.33E-04	5.64E-01	7.31E-02
Q7L7X3	TAOK1	NA	NA	NA	NA	S421;S965;	1.04E-04	NA	NA
Q7LC44	ARC	NA	NA	NA	NA	Y137;S143;	1.56E-05	NA	NA
Q7Z2W4	ZC3HAV1	T273;S275;	3.92E-05	T273;S275;S378;S636;Y637;	3.20E-05	S284;T273;S275;S378;	1.92E-04	2.05E-01	1.67E-01
Q7Z309	FAM122B	S115;S119;S50;S151;S149;	5.06E-04	NA	NA	S115;S119;	7.80E-05	6.49E+00	NA
Q7Z3B3	KANSL1	NA	NA	S249;	1.30E-05	NA	NA	NA	NA
Q7Z3C6	ATG9A	S828;Y762;S735;S738;S741;	3.72E-05	S828;S735;S738;S741;	8.55E-05	S735;S828;S761;S759;S738;S741;	4.42E-05	8.41E-01	1.93E+00
Q7Z3K3	POGZ	T463;S445;S425;T851;S856;	1.51E-04	S445;S425;S1364;	2.29E-04	S445;S425;S1367;T1368;T463;S1364;S1338;	1.45E-04	1.04E+00	1.58E+00
Q7Z417	NUFIP2	S379;S212;S629;S572;S652;S112;S214;	1.01E-03	S212;S214;S629;S112;S572;S652;Y218;	8.69E-04	S629;S379;S212;S214;S112;S572;S652;S304;S306;	1.30E-03	7.76E-01	6.66E-01
Q7Z422	SZRD1	S37;S107;S39;	2.95E-04	S107;S39;	3.11E-04	S37;S107;S39;S51;	1.95E-04	1.52E+00	1.59E+00
Q7Z434	MAVS	S222;	2.73E-05	S222;S258;	6.15E-04	S222;	6.15E-05	4.43E-01	1.00E+01
Q7Z460	CLASP1	S646;S647;S572;S727;T730;S731;	6.55E-05	S646;S649;S1091;S572;S731;S797;S600;S555;S1196;S647;S727;T730;	3.76E-04	S646;S1091;S723;S727;T798;S600;S649;S1196;S647;S797;S572;	3.38E-04	1.94E-01	1.11E+00
Q7Z4S6	KIF21A	S853;S1212;	1.15E-04	S853;S1662;S1673;S1212;S1239;T1664;	2.49E-04	S853;S1662;S1673;S1304;S1307;S1309;T1664;S1239;S1212;S1274;S1275;	5.33E-04	2.16E-01	4.68E-01

Q7Z4V5	HDGFRP2	S236;S240;S366;S369;S370;S142;S144;S232;S664;S625;S418;S633;S634;S652;	8.96E-04	S366;S369;S370;S664;S625;S652;	1.27E-03	S366;S369;S370;S625;S664;	6.08E-04	1.47E+00	2.09E+00
Q7Z569	BRAP	S97;S117;	4.11E-05	NA	NA	S117;S107;S119;	1.19E-04	3.45E-01	NA
Q7Z5J4	RAI1	S683;S691;T696;S1374;	3.49E-05	NA	NA	S1374;	1.04E-05	3.37E+00	NA
Q7Z5K2	WAPL	S459;S461;S77;S221;S226;	4.99E-04	S459;S461;S77;S221;S223;S226;	4.73E-04	S459;S461;S221;S223;S77;S226;	6.97E-04	7.16E-01	6.79E-01
Q7Z5L9	IRF2BP2	S71;S455;S460;S244;S360;S406;S457;S175;	1.09E-03	S360;S175;	7.82E-04	S360;S244;S175;	9.22E-04	1.18E+00	8.48E-01
Q7Z6E9	RBBP6	S1277;S1328;S1694;S1699;S1179;S768;S770;S772;S861;S1644;S1646;S1648;S712;S714;	3.11E-04	Y715;S716;S770;S772;S1273;S1277;S712;S714;S1179;	2.12E-04	S1328;S1277;S770;S772;S768;S1715;S1694;S1695;S1705;S1535;	1.04E-04	2.98E+00	2.03E+00
Q7Z6Z7	HUWE1	S2362;S2365;T2366;S1907;	4.95E-05	S1907;S2362;S2365;S3373;S2887;T2889;S3919;T3924;T3927;S3662;	2.91E-04	S1907;S2595;S3373;S2362;S2365;S3919;T3924;T3927;S2593;S3936;S2918;S3662;S2887;S2888;	1.21E-03	4.11E-02	2.42E-01
Q86SQ0	PHLDB2	S384;S387;S489;S415;S204;S501;S71;S73;	9.23E-05	S334;S384;S387;S415;	2.64E-05	S384;S387;S513;S334;S415;S468;T916;S255;	1.79E-04	5.15E-01	1.48E-01
Q86TB9	PATL1	S179;T178;S184;	5.15E-05	S179;S177;S184;	1.00E-04	S179;T178;S177;	9.59E-05	5.37E-01	1.05E+00
Q86TC9	MYPN	S418;S131;S112;S928;S759;T251;S258;S813;T820;S127;S561;S255;S818;S124;	4.97E-04	NA	NA	S928;S131;T251;S418;S759;T250;S255;S127;	2.44E-04	2.03E+00	NA
Q86TG7	PEG10	S252;	2.63E-05	NA	NA	S252;	1.79E-06	1.47E+01	NA
Q86U42	PABPN1	S150;	1.67E-05	NA	NA	NA	NA	NA	NA
Q86U86	PBRM1	S1453;S648;Y462;Y470;	1.57E-05	S1453;S10;	1.69E-05	S1453;S648;	2.44E-05	6.44E-01	6.96E-01
Q86U90	YRDC	NA	NA	NA	NA	S60;S37;	8.18E-05	NA	NA
Q86UE4	MTDH	S298;S426;S84;	1.40E-04	S568;S426;S298;	1.18E-04	S298;S84;	1.77E-04	7.89E-01	6.68E-01
Q86UU0	BCL9L	S116;S118;S21;S25;S813;S750;	1.72E-04	S116;S118;S21;S25;	3.33E-05	S88;S21;S25;S118;	1.08E-04	1.59E+00	3.08E-01
Q86UU1	PHLDB1	S518;S520;S443;S430;S692;S693;S539;S578;S583;S404;	9.57E-05	T580;S583;T522;	7.81E-06	S578;S520;T522;S443;S430;S518;T580;T516;	6.78E-05	1.41E+00	1.15E-01
Q86VM9	ZC3H18	S67;S74;S78;S95;S46;S842;T109;S857;S868;T851;S492;T93;S83;S536;S110;S34;	1.37E-03	S532;S534;S67;S74;S94;S95;S868;S46;S110;S83;	4.10E-04	S95;S46;S67;S534;S74;S78;S842;S868;T93;S83;S59;T796;S532;S53	6.86E-04	2.00E+00	5.97E-01

		S59;S893;				6;T162;S893;			
Q86VQ1	GLCCI1	S105;S108;S223;S138;S139;	3.91E-05	S105;S108;S223;S345;S394;S397;S107;S30;S26;S172;S138;S139;S398;	2.29E-04	S30;S108;S145;S148;S223;T81;S303;T110;S138;	7.38E-05	5.30E-01	3.10E+00
Q86WB0	ZC3HC1	S335;S62;S344;S338;S350;S407;	8.33E-05	S58;S62;S350;S338;S344;S407;S370;S335;S358;S24;T28;	3.70E-04	S62;S335;S338;S344;S354;S407;S370;S358;S321;S409;S410;	6.90E-04	1.21E-01	5.37E-01
Q86WR0	CCDC25	S204;	1.87E-04	NA	NA	NA	NA	NA	NA
Q86X53	ERICH1	S254;S238;	3.84E-05	NA	NA	S238;S254;	9.10E-05	4.22E-01	NA
Q86X95	CIR1	S305;S202;S394;	2.18E-05	S202;	1.04E-04	S202;	6.72E-05	3.24E-01	1.55E+00
Q86XP3	DDX42	Y183;S751;S754;S185;	9.25E-05	S754;S758;S96;S751;	2.16E-04	S185;S96;S751;S754;	1.13E-04	8.15E-01	1.90E+00
Q86YP4	GATAD2A	S100;S107;S114;S340;T189;	3.78E-04	S100;S107;S114;S343;S340;	5.37E-04	S100;S107;S114;T189;S340;	3.78E-04	1.00E+00	1.42E+00
Q86YV5	SGK223	NA	NA	NA	NA	S745;S148;	1.95E-05	NA	NA
Q8IU81	IRF2BP1	S453;S384;S66;S436;	1.07E-04	S384;S453;	1.98E-04	S453;S66;S384;S421;S436;	3.64E-04	2.94E-01	5.45E-01
Q8IUD2	ERC1	S37;S17;S21;	1.89E-04	S37;T35;	1.36E-05	S37;S17;S21;T38;T35;	7.84E-05	2.41E+00	1.74E-01
Q8IV50	LYSMD2	NA	NA	NA	NA	S24;S29;	7.37E-06	NA	NA
Q8IVF2	AHNAK2	S294;S280;T5709;	9.13E-05	NA	NA	S294;S593;S280;T5709;S765;S3408;S1112;S509;	3.72E-04	2.46E-01	NA
Q8IVT2	MISP	S575;S543;S284;T287;S394;S395;	4.76E-05	S284;T287;S575;S394;	1.80E-04	S284;T287;S575;S394;S395;S397;S471;S214;S400;S586;S541;S675;S543;T377;S213;	9.22E-04	5.16E-02	1.96E-01
Q8IWA0	WDR75	S779;S782;	7.77E-05	NA	NA	T798;S796;	8.82E-05	8.81E-01	NA
Q8IWS0	PHF6	S199;S204;S155;S138;S203;S206;	2.03E-04	S155;S203;S204;S199;	3.00E-05	S154;S138;S4;T12;S155;S199;S203;	1.02E-04	1.99E+00	2.94E-01
Q8IWX8	CHERP	S815;S817;T819;S802;S804;S806;	4.30E-04	S815;S817;T819;	7.56E-04	S815;S817;T819;S806;S802;S804;	6.07E-04	7.08E-01	1.24E+00
Q8IXM2	C17orf49	S96;S110;	8.10E-05	S96;S146;S150;S110;	2.96E-04	S96;S110;	1.77E-04	4.56E-01	1.67E+00
Q8IXQ3	C9orf40	S76;	1.10E-05	NA	NA	S76;T94;	1.65E-05	6.64E-01	NA
Q8IXT5	RBM12B	S638;S375;S377;S250;S254;	1.23E-04	S250;S254;S710;S718;	1.82E-05	S250;S254;S377;S638;S839;	2.47E-05	5.00E+00	7.39E-01
Q8IY57	YAF2	S167;	3.99E-05	NA	NA	T158;T134;S167;S136;	1.43E-05	2.79E+00	NA
Q8IY81	FTSJ3	S335;S336;S347;S333;S59	2.13E-04	S335;S336;S644;S333;S5	5.45E-04	S333;S335;S336;S644;	4.75E-04	4.49E-01	1.15E+00

		9;S458;S468;S471;		99;		S599;			
Q8IYB3	SRRM1	S463;S465;S260;S769;S773;S775;S738;S740;S874;T445;S207;S211;S429;S431;S402;T406;S713;S717;S605;S607;S560;S562;S754;S756;T220;T872;S752;S694;T614;S616;S549;S551;S781;S597;S683;S685;S450;S452;S626;S628;S636;S638;T581;S583;S791;S802;T572;T574;T416;S653;S389;S393;S316;S209;S306;S310;S725;T727;S658;S162;S186;S187;S308;S590;S592;S705;S707;S391;S695;S696;T693;S667;S414;S743;S797;S715;S380;S901;S436;S196;S198;S200;	4.03E-02	T872;S874;S738;S740;S769;S775;S777;S773;S781;S754;S756;S260;S463;S465;T614;S616;S705;S389;S391;S393;S743;S605;S607;S429;S431;S626;S628;S683;S685;T581;S583;S402;T406;S560;S562;S450;S549;S551;T572;T574;T220;S414;S207;S209;S211;S452;S306;S310;Y596;S636;S638;S667;S713;S717;S590;S592;S725;T727;S380;S179;S181;S752;S597;S707;S694;S316;S186;T378;S379;S196;S198;S200;T416;T718;S426;S653;	3.53E-02	T872;S874;S769;S775;S738;S740;S260;S463;S465;S773;S402;T406;S754;S756;S605;S607;S389;S391;S393;S560;S562;S549;S551;S752;S636;S638;S450;S452;S626;S628;S683;S685;S429;S431;T614;S616;S713;S717;T581;S583;S694;S414;T572;T574;T220;T416;Y596;S306;Y309;S310;S725;T727;S207;S211;S653;S187;S209;S590;S592;S696;S186;S781;S695;S597;T445;S316;S265;S715;S426;S705;S707;S380;	2.20E-02	1.83E+00	1.60E+00
Q8IYL3	C1orf174	S148;	9.88E-06	S145;S148;	1.10E-05	S148;	5.69E-06	1.74E+00	1.94E+00
Q8IZ21	PHACTR4	T358;S118;	2.10E-04	S118;T358;S514;S516;S518;S590;	3.86E-04	S118;S344;T358;	2.26E-04	9.29E-01	1.71E+00
Q8IZH2	XRN1	S1645;S1299;	4.93E-05	NA	NA	S1645;	3.57E-05	1.38E+00	NA
Q8IZL8	PELP1	S477;S481;S743;T745;S1043;	2.56E-04	S477;S481;S485;	2.43E-04	S477;S481;T745;	3.89E-04	6.57E-01	6.23E-01
Q8IZP0	ABI1	S183;S323;S225;	2.86E-04	NA	NA	S225;S323;	8.67E-05	3.30E+00	NA
Q8N122	RPTOR	S863;T865;	3.59E-05	S877;S859;S863;S722;	1.86E-04	S859;S863;S877;S722;T865;	2.27E-04	1.59E-01	8.19E-01
Q8N163	CCAR2	S675;S678;S681;	1.60E-05	NA	NA	NA	NA	NA	NA
Q8N1F8	STK11IP	S398;S772;	1.96E-05	NA	NA	S398;S772;	3.03E-05	6.48E-01	NA
Q8N1G0	ZNF687	S1191;S1057;S242;S253;S266;S271;S227;S239;S140;S225;S275;	2.43E-04	S253;S242;S183;S1191;	6.97E-05	S1191;S253;S1057;	5.42E-05	4.49E+00	1.29E+00
Q8N292	GAPT	NA	NA	NA	NA	S82;T89;Y93;	3.35E-05	NA	NA
Q8N2M8	CLASRP	S335;S285;Y453;S547;S315;S317;T319;	5.76E-05	S285;S547;	3.85E-05	S335;S315;S317;T319;S101;S285;S294;	3.55E-05	1.62E+00	1.08E+00
Q8N3V7	SYNPO	S833;S758;S777;	5.33E-05	NA	NA	NA	NA	NA	NA

Q8N3X1	FNBP4	T479;S116;S499;S508;S432;S18;S435;S464;	2.41E-04	S116;S432;S464;	8.22E-05	S116;S18;S432;T479;	4.01E-04	5.99E-01	2.05E-01
Q8N4C8	MINK1	S763;S777;S601;	6.55E-05	NA	NA	S601;S641;	1.17E-05	5.60E+00	NA
Q8N556	AFAP1	S668;	6.77E-05	NA	NA	T341;S277;S342;S343;S265;S679;T689;S687;S668;S710;S664;S336;T337;S283;S683;S548;S665;	1.37E-03	4.94E-02	NA
Q8N5F7	NKAP	S157;T161;S149;S9;S36;	9.96E-05	S149;S46;S48;S25;S27;S85;S157;T161;	1.08E-04	S157;T161;S149;S9;	5.36E-05	1.86E+00	2.01E+00
Q8N5I9	C12orf45	S15;S14;	1.01E-04	NA	NA	NA	NA	NA	NA
Q8N684	CPSF7	S195;S197;S48;S60;T203;S423;S429;	1.01E-04	T203;S205;	8.49E-05	T203;S195;S197;S48;	1.92E-04	5.24E-01	4.43E-01
Q8N6N3	C1orf52	S158;	2.97E-04	NA	NA	S158;	6.74E-04	4.40E-01	NA
Q8N6T3	ARFGAP1	NA	NA	S361;	5.76E-06	T135;S343;S129;	4.64E-05	NA	1.24E-01
Q8N8A6	DDX51	S83;	1.91E-04	NA	NA	S83;S103;	3.00E-04	6.38E-01	NA
Q8N8S7	ENAH	T489;	1.02E-04	S125;T489;S541;	7.63E-04	S125;T489;T481;T487;	8.83E-04	1.15E-01	8.64E-01
Q8N9T8	KRI1	S171;S136;S628;S639;	7.71E-04	S136;S171;	3.45E-04	S136;S171;Y130;S141;	7.59E-04	1.02E+00	4.55E-01
Q8NAV1	PRPF38A	S193;S194;S292;S294;S296;S226;S277;S279;S281;S250;S252;	4.48E-04	S250;S252;S226;S292;S294;S296;S193;S194;	5.18E-04	S193;S194;S260;S262;S292;S294;S296;S279;S281;S277;S250;S252;S226;	1.48E-04	3.02E+00	3.50E+00
Q8NBN3	TMEM87A	S540;	3.22E-05	S540;T530;	1.65E-04	S540;	2.14E-04	1.51E-01	7.73E-01
Q8NC44	FAM134A	S385;S344;S347;T279;S281;	1.02E-04	S385;T279;S281;T334;S337;S283;S344;S347;	1.22E-04	T279;S281;S344;S347;S283;	2.63E-05	3.87E+00	4.64E+00
Q8NC51	SERBP1	S234;T226;S197;S74;S25;T388;	1.48E-04	S234;S25;S394;	8.22E-05	S25;S234;S394;S74;T226;S392;	1.68E-04	8.82E-01	4.88E-01
Q8NCD3	HJURP	NA	NA	S473;S486;S123;S140;	4.40E-05	S473;S210;	2.56E-05	NA	1.72E+00
Q8NCF5	NFATC2IP	S204;S90;S92;S88;S37;S168;S170;S173;	1.67E-04	S88;S90;S92;	5.09E-05	S201;S204;S88;S90;S92;S198;	2.90E-04	5.77E-01	1.75E-01
Q8NCN4	RNF169	NA	NA	NA	NA	S368;S371;S374;S423;	9.08E-06	NA	NA
Q8ND56	LSM14A	S216;S192;S182;	9.43E-04	S216;S178;S182;S183;	2.03E-03	S216;S192;S178;S182;	1.15E-03	8.23E-01	1.77E+00
Q8NDI1	EHBP1	S1058;S426;S436;S751;	1.28E-05	S426;S436;S1058;S432;S428;	2.34E-04	S295;S428;S432;S436;S1058;S426;T663;S854;S751;S1035;S649;	1.32E-04	9.73E-02	1.77E+00

Q8NDT2	RBM15B	S552;S109;S267;S562;S609;	1.45E-04	S552;S598;S600;S601;S562;	2.92E-05	S552;S265;S267;S113;	5.42E-05	2.68E+00	5.39E-01
Q8NDX5	PHC3	S315;S263;T609;S616;	2.82E-05	NA	NA	T609;S616;S315;S842;S229;	3.37E-05	8.38E-01	NA
Q8NE71	ABCF1	S228;S105;T108;S109;S140;S22;	2.20E-03	S228;T108;S109;S22;S105;S140;	6.40E-03	S228;S105;T108;S109;S140;S22;	2.21E-03	9.93E-01	2.89E+00
Q8NEF9	SRFBP1	S203;	1.25E-04	S205;S203;	2.32E-04	S203;	9.97E-05	1.25E+00	2.33E+00
Q8NEJ9	NGDN	S142;S143;	9.20E-05	S142;S143;	1.23E-04	S142;S143;	1.70E-04	5.40E-01	7.22E-01
Q8NEY8	PPHLN1	S205;S201;T204;S133;S210;	6.93E-04	S205;S155;S133;T204;S201;S197;	3.83E-04	T200;S201;S205;S133;S143;S155;S207;	9.11E-04	7.61E-01	4.21E-01
Q8NF91	SYNE1	T3803;S1993;T2001;	2.80E-05	NA	NA	NA	NA	NA	NA
Q8NFC6	BOD1L1	S2986;S2907;S482;S484;Y2777;S2779;S1531;S3019;	3.28E-04	S2986;S482;S484;S2779;S3019;T1529;S2907;	1.65E-04	S2986;S2779;S2780;S482;S484;	5.62E-05	5.84E+00	2.93E+00
Q8NFH5	NUP35	T308;S73;	2.44E-05	NA	NA	S66;S73;T308;S259;T265;	3.51E-05	6.95E-01	NA
Q8NHQ9	DDX55	S594;	1.43E-05	S594;	1.18E-04	S594;	1.98E-04	7.23E-02	5.95E-01
Q8NI08	NCOA7	S211;S208;Y187;	6.19E-05	S208;S211;	1.92E-05	S208;S211;S209;T360;Y187;S179;	1.61E-04	3.84E-01	1.19E-01
Q8NI27	THOC2	S1417;S1422;	3.79E-05	S1417;	7.87E-06	S1419;T1285;T1289;T1288;S1417;	1.67E-05	2.27E+00	4.71E-01
Q8NI35	INADL	S1212;	2.30E-05	T1209;S1212;	5.18E-05	S1212;T1209;	2.91E-05	7.91E-01	1.78E+00
Q8TAD8	SNIP1	S52;S54;S35;S153;S74;S76;	4.50E-04	S52;S54;S35;S74;S76;S153;	2.17E-04	S49;S394;S52;S54;S35;S153;S74;S76;S159;	2.85E-04	1.58E+00	7.60E-01
Q8TAP9	MPLKIP	S115;S47;T51;	4.49E-05	S115;	3.05E-05	S47;T51;S115;	7.10E-05	6.33E-01	4.29E-01
Q8TAQ2	SMARCC2	S302;S306;S286;S304;S347;S283;	6.77E-04	S302;S306;S283;S286;T308;	4.44E-04	S302;S306;S283;S286;T308;S304;	5.01E-04	1.35E+00	8.85E-01
Q8TB72	PUM2	S136;S182;	1.50E-04	S136;S587;S182;	6.94E-05	S136;S182;	1.39E-04	1.08E+00	5.01E-01
Q8TBB5	KLHDC4	S413;S418;	2.93E-05	S413;S418;	4.41E-05	S413;S418;S407;	1.15E-04	2.55E-01	3.85E-01
Q8TBF4	ZCRB1	S210;S216;S155;	9.54E-05	S210;S216;S155;T68;	7.15E-05	S210;S216;S155;	7.12E-05	1.34E+00	1.00E+00
Q8TBM8	DNAJB14	S67;	1.40E-05	NA	NA	NA	NA	NA	NA
Q8TBZ3	WDR20	NA	NA	NA	NA	S432;	7.85E-05	NA	NA
Q8TCJ2	STT3B	S498;S499;	3.05E-05	S498;S499;	9.35E-04	S498;S499;	4.81E-04	6.33E-02	1.94E+00
Q8TD16	BICD2	T573;T567;S568;	1.32E-04	NA	NA	NA	NA	NA	NA
Q8TDC3	BRSK1	NA	NA	NA	NA	S322;S325;	1.26E-03	NA	NA
Q8TDD1	DDX54	S75;S39;S41;S782;	9.98E-05	S39;S41;S75;	2.27E-04	S75;S782;S34;S41;	1.25E-03	7.97E-02	1.81E-01

Q8TE77	SSH3	S87;S9;S653;	1.87E-05	S87;S9;S649;S653;S37;S484;	2.31E-04	S9;S87;S649;S653;	1.28E-04	1.46E-01	1.80E+00
Q8TEA8	DTD1	S197;S194;	3.95E-04	S197;S205;S194;	5.05E-04	S194;S196;S204;S197;S205;	5.93E-04	6.67E-01	8.51E-01
Q8TF01	PNISR	S290;S304;S305;S311;S313;S321;S571;S573;S575;S211;S611;S613;S597;	2.94E-04	S286;S571;S573;S575;Y576;S577;S611;S613;S290;S211;S601;	3.93E-04	S290;S286;S211;S611;S613;S571;S573;S575;	1.45E-04	2.03E+00	2.71E+00
Q8TF74	WIPF2	T269;S235;S267;	8.94E-05	S235;S267;	2.11E-04	T269;S235;S267;	8.87E-05	1.01E+00	2.38E+00
Q8WU90	ZC3H15	S381;	5.79E-05	S381;	7.26E-05	S381;	2.49E-05	2.32E+00	2.91E+00
Q8WUA4	GTF3C2	NA	NA	S165;S167;	6.35E-05	S167;S901;	7.30E-05	NA	8.70E-01
Q8WUB8	PHF10	S297;S301;S327;	1.07E-04	S297;S301;	1.16E-04	S297;S301;S327;	1.47E-04	7.25E-01	7.86E-01
Q8WUZ0	BCL7C	T111;T118;S126;	5.98E-04	S126;T118;S122;	5.38E-05	T111;S126;T118;S97;	1.95E-04	3.06E+00	2.75E-01
Q8WVB6	CHTF18	S871;S64;	3.42E-05	S871;S64;	4.06E-05	S64;S871;	8.95E-05	3.83E-01	4.54E-01
Q8WVC0	LEO1	S294;S296;S300;S151;S154;S162;S608;S610;T188;S197;S277;S279;S658;S630;S66;T629;S171;S179;S607;	9.38E-04	S171;S179;S658;S608;S610;S630;T629;S151;S154;S162;S294;S296;S300;Y606;S607;S637;S277;S279;	5.03E-04	S294;S296;S300;S630;S658;S277;S279;T302;S151;S154;S162;S171;S179;T629;Y606;S607;S608;	1.23E-03	7.62E-01	4.08E-01
Q8WVK2	SNRNP27	S61;S6;S8;S59;S63;	2.01E-04	NA	NA	NA	NA	NA	NA
Q8WW12	PCNP	S147;S119;	2.17E-04	NA	NA	NA	NA	NA	NA
Q8WWI1	LMO7	S960;S1423;S988;S991;S1593;S1026;S867;S873;S1510;S1586;S865;S964;S1493;	6.34E-04	S1510;S1593;S865;S867;S988;S991;S1423;	4.07E-04	S1586;S1510;S1516;S867;T990;S991;S1423;S988;S1593;S873;S276;T956;S960;S805;S751;	1.56E-03	4.06E-01	2.61E-01
Q8WWM7	ATXN2L	S594;S111;S32;S426;S634;S684;S339;S680;T683;S449;S335;S630;S423;S391;S56;S306;T681;	2.63E-03	S391;S634;S684;T681;S424;S111;S630;T632;S496;S680;S56;	7.27E-04	S111;S32;S391;S634;S493;S496;S423;S684;S594;S56;S559;S680;S339;S630;T683;S335;T31;	1.77E-03	1.49E+00	4.11E-01
Q8WWQ0	PHIP	S1783;S911;S1281;S1283;S879;S880;S881;	6.07E-05	S1281;S1283;S1783;S911;S879;S880;S881;	2.78E-04	S1281;S1283;S879;S880;S881;S1783;S911;S1315;S674;	2.45E-04	2.48E-01	1.14E+00
Q8WX93	PALLD	S766;S763;S1116;S1118;S1121;S684;T704;S708;S893;S1101;S1104;S641;S688;S115;S755;	3.42E-03	S1116;S1118;S1121;S893;S1104;	1.56E-04	S1118;S1121;S1116;S893;S684;S688;S641;S1104;S1101;T704;	2.70E-03	1.27E+00	5.79E-02

Q8WXI9	GATAD2B	S135;S486;T120;S122;S129;S338;	1.83E-04	NA	NA	S129;S135;S486;T120;S122;	8.17E-05	2.24E+00	NA
Q8WY36	BBX	S844;S159;	5.30E-05	NA	NA	T722;S844;	1.96E-05	2.70E+00	NA
Q8WYP5	AHCTF1	S1160;S1222;S1283;S1541;S1944;S1218;S1214;S1216;	2.90E-04	S1160;S1218;S1944;S1541;S1884;S1222;S2222;S2226;	8.14E-05	S1160;S1232;S1222;S1944;S1541;S1218;	1.01E-04	2.87E+00	8.04E-01
Q8WZ42	TTN	S16647;S16661;	4.31E-04	S16649;S16661;	2.10E-03	S16661;Y6045;T6046;T14042;S14046;S16649;S21720;	5.73E-04	7.52E-01	3.67E+00
Q92504	SLC39A7	S275;S276;S293;T294;	4.48E-05	S275;S276;	4.99E-04	S275;S276;	9.69E-05	4.62E-01	5.15E+00
Q92530	PSMF1	S153;	2.44E-05	S153;	3.67E-04	S153;	2.92E-05	8.36E-01	1.25E+01
Q92538	GBF1	T1317;S128;S1298;S1318;	1.26E-04	Y1316;S1298;S1318;	1.01E-03	S1298;S1773;S1784;Y1316;S1318;S1300;	5.07E-04	2.48E-01	2.00E+00
Q92576	PHF3	S1614;S125;S1133;	3.85E-05	S1642;S122;	1.26E-05	S1614;S1642;	2.17E-05	1.77E+00	5.82E-01
Q92597	NDRG1	S330;S333;S336;	8.46E-05	S330;	3.97E-05	S330;T328;S333;T335;S336;	1.21E-04	7.00E-01	3.28E-01
Q92598	HSPH1	S809;	5.18E-04	S809;	3.35E-04	S809;	6.72E-04	7.71E-01	4.98E-01
Q92609	TBC1D5	NA	NA	NA	NA	T42;S554;S544;	1.22E-05	NA	NA
Q92613	JADE3	NA	NA	NA	NA	S566;S793;S794;	1.72E-05	NA	NA
Q92614	MYO18A	S2041;S2043;S2020;S1998;S2002;S2007;	4.06E-04	S2041;S2043;S2020;S1998;S2002;S2010;T2045;S102;S103;S140;T2009;	8.43E-04	S2041;S2043;S1998;S2002;S2020;	3.14E-04	1.29E+00	2.68E+00
Q92615	LARP4B	T518;S718;S736;T732;	5.08E-05	S516;T518;S524;S736;S601;S731;T732;S526;	2.32E-04	S524;T518;S736;S601;S526;T732;Y501;	1.54E-04	3.31E-01	1.51E+00
Q92667	AKAP1	NA	NA	S429;S151;S445;	1.30E-04	S151;S429;S445;	1.54E-05	NA	8.49E+00
Q92688	ANP32B	T244;	4.56E-03	T244;	1.12E-03	T244;	7.73E-04	5.90E+00	1.44E+00
Q92733	PRCC	S157;S159;S267;S212;T239;S241;S209;	1.11E-03	S267;S157;S159;T261;	2.65E-03	S157;S159;S267;S212;S209;T215;S241;	2.27E-03	4.91E-01	1.17E+00
Q92734	TFG	S197;S193;	4.54E-04	NA	NA	NA	NA	NA	NA
Q92766	RREB1	S1653;S1219;S1225;S1320;	1.97E-05	S1219;S1225;S1167;S1174;S1175;S42;S36;S161;T31;T1121;	1.36E-04	S1167;S1174;S1175;	1.88E-05	1.05E+00	7.20E+00
Q92769	HDAC2	S422;S424;S394;	7.94E-04	S422;S424;	1.52E-03	S422;S424;S394;	1.62E-03	4.90E-01	9.35E-01
Q92797	SYMPK	S1259;S1243;T1257;	2.96E-05	S1259;	2.65E-05	NA	NA	NA	NA
Q92841	DDX17	S599;	4.57E-05	S64;S599;	6.01E-04	S64;S599;S125;S671;	1.53E-04	2.99E-01	3.94E+00
Q92882	OSTF1	S213;T200;	8.20E-04	T200;S213;	8.81E-04	S213;	7.66E-04	1.07E+00	1.15E+00

Q92890	UFD1L	NA	NA	S299;S247;	2.45E-04	S247;S299;	7.32E-05	NA	3.35E+00
Q92922	SMARCC1	S328;S330;S310;	5.18E-04	S328;S330;	5.83E-04	S328;S330;S310;	6.47E-04	8.01E-01	9.01E-01
Q92945	KHSRP	S54;S193;S480;	1.93E-05	S181;S193;S480;	1.09E-04	S184;S480;	5.01E-05	3.86E-01	2.18E+00
Q92974	ARHGEF2	S956;S941;S960;S932;S782;S645;	2.72E-04	S886;S932;S953;S960;S952;S645;S782;	9.33E-05	S953;S956;S932;S960;S645;S696;S782;	4.81E-04	5.65E-01	1.94E-01
Q92994	BRF1	NA	NA	S357;T365;S358;	1.79E-05	T365;S358;	1.90E-05	NA	9.43E-01
Q93009	USP7	S18;	5.63E-05	NA	NA	NA	NA	NA	NA
Q969G5	PRKCDBP	S165;S166;	3.79E-04	NA	NA	S165;S166;	4.08E-04	9.29E-01	NA
Q969T4	UBE2E3	S8;	2.07E-05	NA	NA	S8;	6.88E-05	3.00E-01	NA
Q969V6	MKL1	S454;S511;T450;	4.40E-05	NA	NA	NA	NA	NA	NA
Q96A49	SYAP1	S269;T248;	3.95E-05	S269;T248;	9.27E-05	S269;T248;	1.48E-04	2.66E-01	6.24E-01
Q96AE4	FUBP1	S630;T153;	7.80E-04	NA	NA	S630;Y626;	1.09E-04	7.13E+00	NA
Q96AT1	KIAA1143	S50;S146;	8.98E-04	S146;S50;	1.78E-04	S146;S50;	1.33E-03	6.75E-01	1.34E-01
Q96B01	RAD51AP1	NA	NA	NA	NA	S19;S21;S296;	7.48E-06	NA	NA
Q96B23	C18orf25	S326;S327;S329;S145;S66;	2.82E-04	S66;S69;S76;	1.23E-04	S66;S67;S145;	1.38E-04	2.04E+00	8.89E-01
Q96B36	AKT1S1	S202;S203;S212;S88;T97;S211;S92;T198;	1.10E-03	S202;S203;S211;S88;S92;S183;S212;	1.66E-03	S202;S203;S92;S212;S211;S183;S88;T97;	7.63E-04	1.44E+00	2.18E+00
Q96B86	RGMA	T128;	5.13E-05	NA	NA	NA	NA	NA	NA
Q96C19	EFHD2	S76;S74;	2.82E-04	NA	NA	S76;S74;	9.48E-05	2.98E+00	NA
Q96C57	C12orf43	S175;S138;	2.84E-04	NA	NA	S138;T182;	1.33E-04	2.14E+00	NA
Q96CB8	INTS12	S127;S378;	7.56E-05	NA	NA	S128;S378;	3.12E-05	2.43E+00	NA
Q96CP2	FLYWCH2	S21;	3.33E-04	NA	NA	S21;	6.16E-05	5.41E+00	NA
Q96CW6	SLC7A6OS	NA	NA	S308;S302;	9.33E-05	S302;S308;	8.66E-05	NA	1.08E+00
Q96D46	NMD3	S468;T470;	3.64E-05	T470;	2.46E-05	S468;T470;	9.69E-05	3.76E-01	2.54E-01
Q96E09	FAM122A	S37;S270;S267;S62;S143;S147;	3.33E-04	S143;S147;S270;S276;S267;S45;S62;	2.18E-04	S267;S270;S37;S35;S48;S76;S276;S143;S147;S286;	2.62E-04	1.27E+00	8.33E-01
Q96EB6	SIRT1	NA	NA	NA	NA	S47;T719;S26;	3.99E-04	NA	NA
Q96EV2	RBM33	S205;S41;S765;S973;S739;	2.76E-04	S205;S765;S973;S41;	1.95E-04	S205;S849;S765;S973;T853;	1.79E-04	1.54E+00	1.09E+00

Q96F86	EDC3	S131;	1.36E-04	S131;	2.67E-04	S131;S163;S161;	3.27E-04	4.17E-01	8.17E-01
Q96FF9	CDCA5	S21;	3.44E-05	NA	NA	NA	NA	NA	NA
Q96FS4	SIPA1	S55;	3.62E-05	S67;S55;	5.37E-05	S834;S839;S55;S67;	7.90E-05	4.59E-01	6.80E-01
Q96FW1	OTUB1	NA	NA	NA	NA	S16;	4.01E-05	NA	NA
Q96G46	DUS3L	T273;S276;	1.31E-04	NA	NA	T273;S276;	2.01E-04	6.52E-01	NA
Q96G74	OTUD5	S64;S165;Y175;S508;S177;	1.99E-04	S165;Y175;	6.71E-05	S165;S177;S508;S64;Y175;	4.75E-04	4.18E-01	1.41E-01
Q96GA3	LTV1	S331;Y204;S206;S211;	2.50E-05	NA	NA	NA	NA	NA	NA
Q96GN5	CDCA7L	S79;T77;	2.28E-05	T77;S21;S79;S139;	7.22E-05	NA	NA	NA	NA
Q96HR8	NAF1	S315;	1.56E-04	S315;	3.38E-05	S315;	1.44E-04	1.08E+00	2.35E-01
Q96I25	RBM17	S155;S229;S222;Y214;	1.96E-04	S155;	1.69E-04	S155;	1.17E-04	1.68E+00	1.44E+00
Q96IZ0	PAWR	S233;S108;	2.27E-05	S231;	8.25E-05	S231;S108;	4.98E-05	4.56E-01	1.66E+00
Q96IZ7	RSRC1	S6;S116;S61;	1.66E-05	NA	NA	S6;	1.89E-05	8.82E-01	NA
Q96JM3	CHAMP1	S382;S386;S651;S653;S204;S214;S308;S319;S459;S282;S286;S297;S652;S627;S452;S507;S427;S432;S436;S445;S476;S632;S633;S626;S405;S311;S108;S443;	1.30E-03	S627;S632;S651;S652;S653;S308;S319;S282;S286;S297;S626;S427;S432;S436;S382;S386;S204;S214;S452;S405;S476;S459;S379;S389;S260;S264;	2.58E-03	S653;S651;S652;S627;S632;S308;S319;S282;S286;S452;S459;S204;S507;S108;S382;S389;S476;T403;S427;S297;S432;S436;S386;S214;S443;S603;S405;S87;S416;S284;S445;S502;	1.80E-03	7.22E-01	1.44E+00
Q96JP5	ZFP91	S85;S101;S103;S83;	1.43E-04	S83;S101;S103;S82;	8.52E-05	S83;S101;S103;S82;	1.66E-04	8.59E-01	5.14E-01
Q96K21	ZFYVE19	S354;S144;S463;	9.74E-04	S354;	2.91E-04	S354;S144;S463;	3.87E-04	2.52E+00	7.52E-01
Q96KR1	ZFR	S1054;	2.39E-05	S1054;	5.97E-05	S1054;	1.04E-04	2.29E-01	5.71E-01
Q96MH2	HEXIM2	S76;S29;T32;	3.88E-05	S29;S76;	2.41E-05	S51;S53;S29;T32;S76;	3.04E-05	1.28E+00	7.93E-01
Q96MU7	YTHDC1	S146;T148;S308;	1.01E-04	S308;S146;T148;	3.16E-04	S308;S146;T148;	2.45E-04	4.10E-01	1.29E+00
Q96NB3	ZNF830	S351;	2.28E-05	S351;	3.31E-05	S351;	6.05E-05	3.76E-01	5.47E-01
Q96P16	RPRD1A	S156;	8.57E-06	NA	NA	S156;	3.84E-05	2.23E-01	NA
Q96PE1	ADGRA2	Y1311;T1329;	3.17E-05	NA	NA	NA	NA	NA	NA
Q96PK6	RBM14-RBM4	T572;S582;S215;T206;	7.26E-04	S618;T206;S582;	2.61E-04	T206;S582;S215;Y648;S618;	2.87E-04	2.53E+00	9.10E-01
Q96QC0	PPP1R10	S545;S313;	1.14E-04	S313;	1.39E-04	S313;T256;	1.50E-04	7.62E-01	9.29E-01
Q96QR8	PURB	S298;S304;S101;	7.84E-05	S298;S304;	2.35E-04	S298;S304;S101;	3.00E-04	2.61E-01	7.84E-01
Q96RK0	CIC	S1382;S1397;S1409;S431;T435;	1.97E-05	S1373;S1382;S1397;S1402;	2.85E-05	S1373;S1382;S700;S173;	3.61E-05	5.45E-01	7.89E-01

Q96RL1	UIMC1	S653;S677;	3.54E-05	NA	NA	NA	NA	NA	NA
Q96RT1	ERBB2IP	S602;S603;S857;S1015;	6.32E-05	T917;S857;	4.64E-05	S1015;S857;S913;	3.59E-05	1.76E+00	1.29E+00
Q96RU3	FNBP1	S296;	3.60E-05	S296;	5.23E-05	S296;	9.72E-05	3.71E-01	5.39E-01
Q96S55	WRNIP1	S153;S65;S77;S91;S92;	4.30E-04	S65;S153;	1.27E-04	S153;S151;S65;	6.18E-04	6.96E-01	2.06E-01
Q96S82	UBL7	S230;	6.47E-05	NA	NA	NA	NA	NA	NA
Q96ST2	IWS1	S438;S440;S415;S420;S362;S363;S365;S287;S289;S398;S400;S196;S198;S69;S80;S377;S300;S302;S274;S235;S237;S304;S248;S261;S263;S250;S313;S511;S513;S422;T317;S82;S315;S93;S95;	4.11E-03	S398;S400;S438;S440;S287;S289;S235;S237;S313;S315;S415;S420;S196;S198;S248;S250;S261;S263;S69;S80;S84;S300;T317;S511;S513;S93;S422;S274;S362;S363;S365;S95;S302;	4.78E-03	S415;S420;S422;S313;S315;S398;S400;S235;S237;S377;S438;S440;S287;S289;S196;S198;S300;S248;S250;S363;S365;S511;S513;S261;S263;S362;S69;S80;S252;S274;S304;S302;	3.72E-03	1.11E+00	1.29E+00
Q96T23	RSF1	S622;S1345;T1305;S1282;S1221;S1223;Y1281;S604;S1359;S1375;S1277;T628;S1226;T1278;	3.96E-04	S1345;S622;T628;S1359;S1375;S604;S629;T1305;	2.42E-04	S1345;T1305;Y1281;S1282;S397;S1359;S604;S1375;S748;S1221;S1223;S622;S1310;S629;S1226;T1371;	6.96E-04	5.68E-01	3.47E-01
Q96T37	RBM15	S670;S674;S257;S259;S656;S109;S294;T568;S686;S128;	5.70E-04	T265;Y266;S670;S674;S294;S656;S257;S259;	1.03E-03	S670;S674;S294;T568;S109;S257;S259;S656;S765;Y266;T32;S722;	4.96E-04	1.15E+00	2.07E+00
Q96T58	SPEN	S736;S740;S1222;S2493;S1006;S725;S727;S1918;S1390;S749;S2120;S2124;S1268;S1278;S1261;S1380;S1382;S2366;T2393;S2101;S1857;	2.18E-04	S2120;S2124;S1380;S1382;S1390;T1643;S1261;	4.80E-05	S1268;S1278;S1380;S1382;S1390;S1225;S2120;S2124;S736;S740;S725;S727;S1392;T2374;S1222;S250;	1.83E-04	1.19E+00	2.63E-01
Q96T60	PNKP	T118;	4.43E-05	T118;	1.86E-04	T118;T122;	6.96E-05	6.37E-01	2.68E+00
Q96T88	UHRF1	S95;S91;	4.75E-05	NA	NA	S91;T93;	1.02E-04	4.65E-01	NA
Q96TA1	FAM129B	S665;S646;S641;S692;S696;S681;S638;	9.03E-04	S641;S646;S665;S681;S692;S696;	5.78E-04	S665;S641;S646;S681;S638;S692;S696;	2.64E-03	3.42E-01	2.19E-01
Q96TC7	RMDN3	S46;	5.17E-05	S46;	5.30E-04	S46;	2.66E-04	1.94E-01	1.99E+00
Q99442	SEC62	NA	NA	T375;T158;S117;	4.79E-04	T375;	1.03E-04	NA	4.64E+00
Q99549	MPHOSPH8	S51;S136;S138;S149;	1.17E-04	S51;S85;	1.49E-04	S51;S85;S136;S138;S149;S403;T440;	2.20E-04	5.29E-01	6.77E-01
Q99575	POP1	S730;	3.17E-05	NA	NA	S730;	1.22E-04	2.61E-01	NA

Q99590	SCAF11	S405;S413;S338;S776;S830;S832;S834;S608;S796;S802;S816;S818;S848;S850;S473;S878;S880;S882;S400;S401;S402;S937;S939;S941;S902;S798;S963;T410;S771;S943;	1.78E-03	S338;S830;S832;S834;S878;S880;S882;S816;S818;S848;S850;S796;S802;S839;	3.87E-04	S405;S413;S608;S472;S907;S796;S802;S816;S818;S830;S832;S834;S878;S880;S882;S338;S848;S850;S839;S475;S902;S937;S939;S943;	7.67E-04	2.32E+00	5.04E-01
Q99613	EIF3C	S166;S182;S39;S178;S181;	4.85E-04	S39;	4.60E-04	S178;S181;S182;S39;S166;	1.11E-03	4.35E-01	4.13E-01
Q99618	CDCA3	S29;S31;S87;	9.86E-06	S31;T37;S29;	2.87E-05	S44;S29;S31;T37;S151;T161;S87;	3.11E-05	3.16E-01	9.21E-01
Q99700	ATXN2	S865;S889;S758;S772;T741;S744;S861;	2.32E-04	NA	NA	T771;S758;S772;S865;S530;S889;T666;S466;S784;	9.45E-05	2.45E+00	NA
Q99729	HNRNPAB	S242;	2.97E-05	NA	NA	NA	NA	NA	NA
Q99733	NAP1L4	S125;	7.65E-04	S125;	9.88E-04	S125;S304;S121;	2.00E-03	3.83E-01	4.94E-01
Q99933	BAG1	NA	NA	NA	NA	T123;S119;	1.01E-05	NA	NA
Q99959	PKP2	S151;S155;S154;	3.47E-05	S151;S154;S155;S82;	1.81E-04	NA	NA	NA	NA
Q9BPX3	NCAPG	S1015;S973;S975;S674;	3.41E-05	S674;S1015;	7.13E-05	S674;S1015;	6.14E-04	5.55E-02	1.16E-01
Q9BQ61	C19orf43	S49;S39;	9.16E-06	NA	NA	NA	NA	NA	NA
Q9BQE9	BCL7B	S114;S122;S127;	6.91E-05	S114;S122;S127;	4.54E-05	S122;S114;S127;	9.62E-05	7.19E-01	4.72E-01
Q9BQG0	MYBBP1A	S1267;T1161;S1163;T1239;S11;S1248;	1.37E-04	S1267;T1161;S1163;S1166;T1227;	1.46E-03	S1267;T1161;S1163;	9.67E-04	1.42E-01	1.51E+00
Q9BRD0	BUD13	S184;S188;S197;S201;S222;S226;S172;S248;S358;S325;T159;S163;S127;T147;S151;S271;S210;S211;S214;S235;S240;T135;S139;T174;S239;T187;	4.41E-04	S197;S201;T135;S139;S325;T159;S163;S354;T123;S127;S240;S184;S185;T187;	2.01E-04	S358;S325;S197;S201;S210;T147;S151;T135;S139;T174;S175;S248;S222;S226;T159;S163;S214;S127;S184;S185;T187;S172;T123;S240;	2.82E-04	1.56E+00	7.13E-01
Q9BRJ6	C7orf50	S175;	4.77E-05	S175;	7.77E-05	S59;S175;	2.14E-05	2.24E+00	3.64E+00
Q9BRQ0	PYGO2	T302;	9.93E-05	T302;	1.10E-04	T302;	1.05E-04	9.46E-01	1.05E+00
Q9BTA9	WAC	S525;S511;S53;	7.31E-05	S525;T293;S64;	3.52E-05	S511;S523;S535;S525;S64;	1.77E-05	4.14E+00	1.99E+00
Q9BTC0	DIDO1	T151;S152;S154;S1456;T1256;S1260;S805;S809;S898;T1255;S1040;T1719;	7.06E-04	T151;S152;S154;S805;S809;S834;S835;T1256;S1260;S1040;S1456;	2.55E-04	S1456;T151;S152;S154;S1260;S1040;T1255;S805;S809;T1733;T12	7.38E-04	9.56E-01	3.46E-01

						56;			
Q9BTE3	MCMBP	NA	NA	NA	NA	S154;	1.10E-04	NA	NA
Q9BTK6	PAGR1	S237;	1.15E-04	S237;	9.62E-05	S237;	8.11E-05	1.41E+00	1.19E+00
Q9BTU6	PI4K2A	S47;S51;S462;	3.64E-05	S47;S51;	3.94E-05	S47;S51;S44;	1.78E-04	2.04E-01	2.21E-01
Q9BU76	C1orf35	S217;S220;T219;S177;S178;S216;	4.19E-04	S217;S220;T219;T175;S177;S178;	3.85E-04	S217;T219;S220;S178;T215;S216;S231;S235;S177;S233;	5.40E-04	7.75E-01	7.13E-01
Q9BUA3	C11orf84	S308;S248;S251;S154;	1.10E-04	S308;S148;S154;	4.02E-05	S248;S251;S308;S148;T253;	3.34E-04	3.31E-01	1.21E-01
Q9BUJ2	HNRNPUL1	S718;S194;	1.45E-04	S194;S718;Y717;	4.99E-04	S718;S194;	2.25E-04	6.46E-01	2.22E+00
Q9BUQ8	DDX23	S107;S109;S14;S39;S23;T25;S106;	8.87E-04	S106;S107;S14;S109;	6.38E-04	S16;S39;S106;S107;S14;S23;T25;S109;	4.43E-04	2.00E+00	1.44E+00
Q9BUR4	WRAP53	S26;S30;S85;S90;S491;	9.63E-05	S85;S90;S114;S112;S491;	2.31E-04	S85;S90;S114;S54;S491;S112;	4.37E-04	2.20E-01	5.28E-01
Q9BUT9	FAM195A	S82;S78;T89;	1.15E-04	NA	NA	S82;	1.12E-04	1.03E+00	NA
Q9BUW7	C9orf16	S82;	2.48E-05	NA	NA	S82;S79;	4.57E-05	5.42E-01	NA
Q9BVC5	C2orf49	S182;S189;S193;T197;S184;	5.24E-04	S189;S193;T198;S182;T197;	5.73E-05	S189;S193;T198;S182;T197;S146;S184;	4.62E-04	1.13E+00	1.24E-01
Q9BVG4	PBDC1	S181;S197;	7.70E-05	S197;	8.99E-05	NA	NA	NA	NA
Q9BVJ6	UTP14A	S29;S31;S437;S405;S445;Y26;	3.35E-04	S29;S31;S445;S405;S407;	3.00E-04	S29;S31;S405;S407;S437;	2.30E-04	1.45E+00	1.30E+00
Q9BVS4	RIOK2	S380;S382;S385;S332;S335;S337;	5.97E-05	S332;S335;S337;S380;S382;S385;	1.11E-04	S380;S382;S337;S335;S332;S442;T295;T328;	1.39E-04	4.30E-01	7.99E-01
Q9BVV8	C19orf24	S117;S120;	1.60E-05	NA	NA	NA	NA	NA	NA
Q9BW61	DDA1	S95;	1.31E-04	NA	NA	NA	NA	NA	NA
Q9BW71	HIRIP3	S159;S160;S330;S332;S333;S223;S227;S359;S363;S370;S196;S199;S125;S142;S143;T84;S87;S551;S555;S530;S98;S100;	2.52E-03	S223;S227;S330;S332;S333;S196;S199;T84;S87;S159;S160;S142;S143;S125;	7.90E-04	S330;S332;S333;S370;S142;S143;S159;S160;S196;S199;S125;S227;S223;S219;T84;S87;S100;S102;S104;	1.05E-03	2.40E+00	7.55E-01
Q9BW85	CCDC94	S211;S213;S220;	2.40E-04	S316;S319;S322;S211;S213;S220;Y313;	1.65E-03	S211;S213;S220;S316;S319;S322;	5.95E-04	4.03E-01	2.77E+00
Q9BWF3	RBM4	NA	NA	NA	NA	S86;S309;	3.06E-04	NA	NA
Q9BWG6	SCNM1	NA	NA	NA	NA	S219;	4.02E-05	NA	NA

Q9BWU0	SLC4A1AP	S466;S312;S82;	1.88E-04	S466;	1.55E-04	S466;S82;	2.69E-04	6.97E-01	5.74E-01
Q9BXB4	OSBPL11	NA	NA	S189;	1.12E-05	S189;S181;T24;	4.56E-05	NA	2.46E-01
Q9BXF6	RAB11FIP5	S393;S307;	2.27E-05	NA	NA	NA	NA	NA	NA
Q9BXK1	KLF16	T55;S109;	1.02E-05	NA	NA	NA	NA	NA	NA
Q9BXP5	SRRT	S67;S74;S540;T544;	2.64E-04	S540;S74;S67;T544;	2.26E-04	T544;S550;S74;S540;S493;S67;	5.61E-04	4.71E-01	4.02E-01
Q9BXY0	MAK16	NA	NA	NA	NA	S197;S199;S200;	1.28E-04	NA	NA
Q9BY44	EIF2A	S506;	3.59E-04	S506;	4.10E-04	S506;	3.95E-04	9.08E-01	1.04E+00
Q9BY89	KIAA1671	S1701;S1695;	2.29E-05	S1701;S1757;S1760;S1488;S1224;	1.37E-04	S128;S1366;S1701;T600;S1695;T1231;	3.38E-05	6.79E-01	4.06E+00
Q9BYE7	PCGF6	NA	NA	NA	NA	S115;	2.06E-05	NA	NA
Q9BYW2	SETD2	S458;S459;S461;S744;S754;S2080;S2082;S321;S323;S1413;S1415;S1417;S624;	9.11E-05	S321;S323;S1413;S1415;S1417;	2.43E-05	S458;S459;S461;S624;S1413;S1415;S1417;S321;S323;	6.67E-05	1.37E+00	3.64E-01
Q9C0B5	ZDHHC5	T696;S380;S554;	7.42E-05	S621;S380;S694;S432;T436;	1.70E-04	S380;T696;S621;S415;S593;S694;S554;	3.38E-04	2.20E-01	5.02E-01
Q9C0C2	TNKS1BP1	S762;S1620;S1621;S494;S1666;S836;S672;S691;S601;S1138;S221;S1103;S1297;S498;S1029;S712;S1715;S228;S872;	2.54E-03	S1620;S1621;S601;S1473;S1476;S429;S836;S1029;S1385;T501;S672;S691;S1666;S178;S1024;S504;S987;S872;	8.78E-04	S984;S836;S601;S691;S695;S712;S1029;S494;S498;S1666;S672;S429;S936;S1138;S1024;S1046;S1620;S1621;S987;T501;S893;S1473;S1476;S1715;S221;S228;S1652;S920;S178;S1031;S872;S435;S1385;S1103;	3.30E-03	7.69E-01	2.66E-01
Q9C0C9	UBE2O	S87;S89;S896;S401;T834;T838;	8.83E-05	NA	NA	S87;S89;S475;S896;	1.79E-04	4.93E-01	NA
Q9C0J8	WDR33	NA	NA	NA	NA	S1213;S1210;	1.62E-05	NA	NA
Q9GZP8	C19orf33	S62;S29;	5.61E-05	NA	NA	S29;	1.81E-05	3.09E+00	NA
Q9GZR2	REXO4	S14;S15;	1.98E-05	S14;S15;	2.80E-05	NA	NA	NA	NA
Q9GZR7	DDX24	S82;S94;	7.04E-05	S94;S82;S93;	2.80E-04	S82;S93;T302;S94;	2.99E-04	2.35E-01	9.36E-01
Q9H0D6	XRN2	S499;S501;S448;	3.53E-04	S499;S501;S448;	2.04E-03	S499;S501;S448;	1.90E-03	1.86E-01	1.08E+00
Q9H0E3	SAP130	S855;T856;S442;	2.09E-05	NA	NA	NA	NA	NA	NA

Q9H0G5	NSRP1	S33;S254;S255;S248;S289;S291;S293;	1.94E-04	S248;S254;S255;S33;S27;	3.43E-04	S33;S291;S293;S289;S248;S254;S255;	1.27E-04	1.53E+00	2.71E+00
Q9H0L4	CSTF2T	S563;	3.65E-05	S563;	6.91E-05	S563;S560;	8.52E-05	4.29E-01	8.11E-01
Q9H1B7	IRF2BPL	S547;S334;S337;S659;S662;S215;S657;S658;S639;	3.42E-04	S657;S662;S547;S659;	2.66E-05	S657;S659;S662;S547;S334;S337;S215;S69;S336;	6.52E-04	5.24E-01	4.08E-02
Q9H1E3	NUCKS1	S54;S58;S61;S19;S75;S79;S214;T202;S204;S73;S229;S234;T179;S181;S130;S132;S223;S240;S50;S14;S30;S113;S144;	3.05E-02	S19;S54;S58;S61;S73;S75;S79;S181;S214;S229;S234;S240;T202;S204;T179;S50;Y26;S30;	3.58E-02	S19;S54;S58;S61;S75;S79;S73;S181;S214;T202;S204;T179;S240;S30;S234;Y26;S130;S132;S229;S223;	3.76E-02	8.11E-01	9.51E-01
Q9H1H9	KIF13A	S1698;	7.81E-05	S1698;T1696;	6.05E-05	S1698;	3.84E-05	2.04E+00	1.58E+00
Q9H2G2	SLK	S571;S372;	1.64E-05	T569;S571;	3.16E-05	S347;S348;S571;	1.64E-05	9.95E-01	1.92E+00
Q9H2H9	SLC38A1	S52;S25;S28;	1.04E-04	T54;S52;	1.27E-04	S52;T54;S25;S28;S56;	1.38E-03	7.52E-02	9.23E-02
Q9H2P0	ADNP	S953;S955;S709;S736;S372;	9.14E-05	NA	NA	S921;S709;S98;	4.87E-05	1.88E+00	NA
Q9H307	PNN	S66;S100;S690;S692;	9.50E-04	S96;S66;S100;S690;S692;	1.39E-03	S66;S100;S690;S692;	1.18E-03	8.08E-01	1.19E+00
Q9H3N1	TMX1	S247;S270;	2.92E-04	S247;S253;S270;	1.22E-03	S247;S270;	9.99E-04	2.92E-01	1.22E+00
Q9H3Q1	CDC42EP4	S64;S292;S295;S174;S142;S138;	4.25E-04	S292;S295;S64;S174;	3.01E-04	S64;S292;S295;S174;S142;S74;	3.29E-04	1.29E+00	9.14E-01
Q9H3S7	PTPN23	NA	NA	S1126;S1123;T1131;S1133;	6.50E-05	T1131;S1133;S1126;S1123;T587;	1.97E-04	NA	3.30E-01
Q9H4A3	WNK1	S2029;S2032;S2027;S2012;S2372;S185;S183;	2.76E-04	S2005;S2029;S2032;S2027;S378;S2012;S2011;	2.52E-04	S2011;S2027;S2029;S2032;S2012;S185;	6.85E-05	4.03E+00	3.67E+00
Q9H4G0	EPB41L1	S578;T475;S678;S648;	4.54E-05	NA	NA	S510;S461;S466;	5.98E-06	7.59E+00	NA
Q9H4H8	FAM83D	S458;S462;	2.64E-05	NA	NA	S458;T459;S462;	1.64E-05	1.61E+00	NA
Q9H4L7	SMARCAD1	S211;S213;S34;S39;T54;S57;Y217;S95;S96;S37;S50;S214;S124;S127;	2.34E-04	S34;S39;S211;S214;S95;S96;S98;Y217;T54;S57;	2.21E-04	S96;S98;S103;S34;S39;S37;S50;S212;S213;S214;S95;T54;S57;S211;Y217;	3.70E-04	6.31E-01	5.98E-01
Q9H4Z3	PCIF1	S30;S144;S17;	3.06E-05	S19;S30;S140;S144;T150;S14;S146;S143;	1.56E-04	T135;S30;S146;T150;S144;	5.31E-05	5.76E-01	2.94E+00
Q9H501	ESF1	S657;S663;S153;S198;T311;S312;S313;S75;S77;S79;	2.71E-04	S657;S663;T311;S312;S313;S153;S198;S694;S75;S77;S82;	2.57E-03	S657;S663;S75;S77;S79;S694;S153;T311;S312;S313;S198;	5.59E-04	4.85E-01	4.60E+00
Q9H5H4	ZNF768	S139;S90;S97;S160;	2.10E-05	S90;S97;S83;	3.83E-05	NA	NA	NA	NA

Q9H6F5	CCDC86	S91;S58;T65;S69;S47;S18;S102;S110;S113;S80;S217;	4.97E-03	S91;S80;T65;S69;S217;S47;S58;S18;S102;Y109;S113;S110;	4.79E-03	S18;S80;S91;S47;S102;S110;S58;T65;S69;S113;S217;	4.63E-03	1.07E+00	1.03E+00
Q9H6T3	RPAP3	S116;S119;S121;	9.00E-05	S116;S119;S121;	6.31E-05	S110;S114;S121;S119;S116;	1.71E-04	5.26E-01	3.69E-01
Q9H6Y2	WDR55	S14;S382;	1.65E-04	NA	NA	NA	NA	NA	NA
Q9H6Z4	RANBP3	S96;S101;S108;S333;S100;	4.07E-04	S353;S108;S333;S96;S101;S100;	9.69E-04	S96;S101;S108;S100;S333;S353;T214;	6.24E-04	6.52E-01	1.55E+00
Q9H788	SH2D4A	S315;	9.72E-05	S315;	9.86E-05	S315;	9.98E-05	9.74E-01	9.89E-01
Q9H799	C5orf42	T2771;S2779;	2.83E-03	NA	NA	T2771;S2779;	4.12E-04	6.87E+00	NA
Q9H7L9	SUDS3	S45;T49;S53;S234;S236;S237;	4.78E-04	S234;S236;S237;	4.65E-04	S45;T49;S53;S234;S236;	3.47E-04	1.38E+00	1.34E+00
Q9H7N4	SCAF1	S612;S614;S498;S500;S965;S734;S738;T989;S724;	2.34E-04	S734;S738;S498;S500;S612;S614;S239;S874;S1005;T1012;S719;S965;	6.43E-04	S734;S738;S239;S612;S614;S500;S498;S965;S724;S548;T994;T989;S1005;S874;	8.13E-04	2.88E-01	7.90E-01
Q9H8G2	CAAP1	S203;S312;S89;	3.30E-05	NA	NA	NA	NA	NA	NA
Q9H8M2	BRD9	S568;S588;	1.56E-05	S588;	1.84E-05	S588;T448;	4.24E-05	3.67E-01	4.35E-01
Q9H8Y8	GORASP2	S451;	3.89E-05	S451;T415;	3.81E-04	S451;S432;	2.68E-04	1.45E-01	1.42E+00
Q9H910	HN1L	S97;S30;	8.57E-05	S97;S144;	6.26E-05	S97;	4.76E-05	1.80E+00	1.32E+00
Q9H9J4	USP42	S856;	3.90E-05	S75;S611;S612;S615;S1219;S1222;S1226;S856;	9.84E-05	S1219;S1222;S1226;S856;S75;S1166;Y607;S611;S612;S615;	1.11E-04	3.52E-01	8.87E-01
Q9HAF1	MEAF6	S122;T124;S125;S118;T120;	9.76E-05	S136;S118;T120;S122;T124;S125;	2.07E-04	S136;S118;T120;S122;	2.90E-05	3.37E+00	7.15E+00
Q9HAU0	PLEKHA5	S933;S937;S855;	3.39E-05	S933;S937;	8.99E-05	S933;S937;S855;S930;S161;	2.92E-05	1.16E+00	3.08E+00
Q9HAW4	CLSPN	S65;S67;S1156;S1005;S1018;S1020;	1.48E-05	NA	NA	NA	NA	NA	NA
Q9HBL0	TNS1	S1269;S899;S1177;	1.83E-05	NA	NA	NA	NA	NA	NA
Q9HC35	EML4	S146;S176;S144;	5.09E-05	T899;S144;S146;S903;T96;T897;T160;	4.97E-04	S176;S144;S146;T899;	5.29E-05	9.62E-01	9.39E+00
Q9HC52	CBX8	S311;S191;S265;	2.40E-04	S191;	4.00E-05	S191;S265;S110;	7.31E-05	3.28E+00	5.48E-01
Q9HCD5	NCOA5	S126;S29;S34;	2.39E-04	S29;S34;S381;	4.16E-04	S29;S34;S381;	2.71E-04	8.80E-01	1.54E+00
Q9HCE3	ZNF532	S130;S133;S134;S314;	3.93E-05	NA	NA	NA	NA	NA	NA

Q9HCH0	NCKAP5L	S567;S573;	2.12E-05	S763;S567;S573;	1.64E-05	NA	NA	NA	NA
Q9HCK8	CHD8	NA	NA	NA	NA	S2519;S2008;T1993;S1995;	5.67E-05	NA	NA
Q9HCN4	GPN1	S338;	2.84E-04	S338;	5.39E-04	S338;S314;	1.14E-03	2.49E-01	4.72E-01
Q9HDC5	JPH1	NA	NA	NA	NA	S475;S479;T461;S465;	5.80E-06	NA	NA
Q9NPI6	DCP1A	S525;S315;S319;S523;T528;	6.78E-05	S315;S319;S525;S522;	2.28E-04	S315;S319;S525;	2.29E-04	2.96E-01	9.92E-01
Q9NPQ8	RIC8A	Y435;T441;S436;	3.97E-05	NA	NA	NA	NA	NA	NA
Q9NQ29	LUC7L	S363;S304;S306;	2.33E-04	NA	NA	NA	NA	NA	NA
Q9NQ55	PPAN-P2RY11	S359;	3.07E-04	S238;S240;S359;	1.66E-03	S238;S240;S359;T280;	1.15E-03	2.68E-01	1.45E+00
Q9NQC3	RTN4	S184;S181;S182;S107;	3.58E-04	S107;S111;S181;S182;S184;T188;	4.04E-04	S181;S182;S184;T188;S107;S361;S362;S664;S666;	7.26E-04	4.94E-01	5.56E-01
Q9NQG5	RPRD1B	S166;S134;	1.89E-04	S166;	2.26E-04	S166;	1.63E-04	1.16E+00	1.38E+00
Q9NQS1	AVEN	S94;	4.40E-05	S94;	6.24E-05	NA	NA	NA	NA
Q9NQS7	INCENP	S828;S831;T832;S148;S899;S218;T219;S481;S197;S263;S306;T199;	2.74E-04	T213;S214;S306;S314;T199;T298;S312;S311;	1.33E-04	S197;T213;S481;S218;T219;T298;S314;	5.64E-05	4.86E+00	2.36E+00
Q9NQW6	ANLN	S99;S102;S54;S182;S323;S642;S95;S97;	3.09E-04	S54;S182;S225;S485;Y671;S672;	4.59E-04	S54;S225;S661;T320;S323;T364;S485;S642;	2.09E-04	1.48E+00	2.19E+00
Q9NQZ2	UTP3	S365;S368;S37;	8.96E-05	S365;S368;S37;	3.67E-04	S365;S368;S37;	2.24E-04	3.99E-01	1.63E+00
Q9NR12	PDLIM7	S217;	8.08E-05	S217;	5.43E-05	S217;	3.01E-04	2.68E-01	1.80E-01
Q9NR19	ACSS2	S30;	1.58E-05	S30;	4.24E-04	NA	NA	NA	NA
Q9NR30	DDX21	S121;S89;S71;S168;S171;S173;	1.78E-03	S121;S171;S173;S168;S89;	4.33E-03	S168;S171;S173;S121;S89;S71;	4.27E-03	4.16E-01	1.02E+00
Q9NR48	ASH1L	NA	NA	NA	NA	T561;S1681;S557;	1.39E-05	NA	NA
Q9NRA8	EIF4ENIF1	S564;	1.73E-05	NA	NA	NA	NA	NA	NA
Q9NRF8	CTPS2	NA	NA	S568;S571;S574;	2.17E-04	S568;S571;S574;	9.27E-05	NA	2.34E+00
Q9NRG0	CHRAC1	S124;	9.89E-05	S124;	7.86E-06	S124;	1.51E-04	6.55E-01	5.20E-02
Q9NRL2	BAZ1A	S1413;S1279;S1283;	8.29E-05	S1413;	2.90E-05	S1363;T1367;S1371;S1413;S1283;S1417;T1547;S1280;S1279;S1320;	3.21E-04	2.58E-01	9.04E-02
Q9NS91	RAD18	S158;S164;S471;	2.00E-04	S471;	5.27E-05	S99;S158;S164;S471;	2.25E-04	8.86E-01	2.34E-01

Q9NSC5	HOMER3	S159;	9.87E-05	NA	NA	S159;	1.17E-04	8.40E-01	NA
Q9NSK0	KLC4	S590;	5.93E-05	S590;S460;	1.22E-04	S590;S460;	7.36E-05	8.06E-01	1.65E+00
Q9NTI5	PDS5B	S1358;S1257;S1283;T1381;S1385;T1370;S1383;S1166;	3.21E-04	S1358;S1283;T1370;T1381;S1383;	2.27E-04	S1358;T1370;S1257;S1283;S1166;	1.71E-04	1.88E+00	1.33E+00
Q9NTJ3	SMC4	S22;S27;S28;	5.54E-04	S22;S27;S28;	5.11E-04	S22;S27;S28;S41;	7.31E-04	7.58E-01	6.99E-01
Q9NTZ6	RBM12	S413;	1.67E-05	NA	NA	NA	NA	NA	NA
Q9NU22	MDN1	T4898;S5015;	1.94E-05	S4538;S5015;T4898;	1.04E-04	T4898;S5015;S4538;	2.86E-05	6.78E-01	3.65E+00
Q9NUL3	STAU2	T488;S492;S486;	2.70E-05	T488;S492;	5.68E-05	T488;S492;	2.03E-05	1.33E+00	2.80E+00
Q9NUQ3	TXLNG	S105;	1.04E-04	NA	NA	S97;Y100;T102;S105;S510;	8.95E-04	1.16E-01	NA
Q9NV56	MRGBP	S194;S195;S191;	1.66E-04	S191;S195;S194;	8.78E-05	S191;S194;S195;	7.47E-05	2.23E+00	1.17E+00
Q9NVD7	PARVA	T16;S19;S14;	6.71E-05	S14;S19;S22;	2.13E-05	S14;S19;T16;S22;	4.67E-04	1.44E-01	4.55E-02
Q9NVM6	DNAJC17	S112;	1.50E-04	S112;	2.89E-05	S112;	1.34E-04	1.12E+00	2.16E-01
Q9NVU0	POLR3E	S161;S162;	3.74E-05	S161;S162;	6.70E-05	S161;S162;	1.15E-04	3.24E-01	5.82E-01
Q9NW82	WDR70	S638;	1.50E-04	NA	NA	NA	NA	NA	NA
Q9NWB6	ARGLU1	S77;S76;	1.70E-04	S77;S56;S58;T61;	1.28E-04	S77;S56;S58;S60;	1.05E-04	1.63E+00	1.22E+00
Q9NWH9	SLTM	S289;S1002;S553;	8.05E-04	S289;	5.37E-04	S289;	6.53E-04	1.23E+00	8.22E-01
Q9NWV8	BABAM1	S49;S29;	2.09E-05	S49;	2.12E-05	S66;S29;S49;	1.69E-04	1.24E-01	1.26E-01
Q9NX40	OCIAD1	S123;	6.48E-06	S108;S123;	6.36E-05	S123;S108;	5.60E-05	1.16E-01	1.14E+00
Q9NX58	LYAR	S276;	1.94E-05	S276;	3.00E-05	S276;	1.44E-05	1.35E+00	2.08E+00
Q9NXE8	CWC25	S218;	1.90E-05	NA	NA	NA	NA	NA	NA
Q9NXG2	THUMPD1	S86;S88;	4.91E-04	S86;S88;	4.45E-03	S86;S88;	3.17E-03	1.55E-01	1.41E+00
Q9NY61	AATF	S316;S320;S321;S203;	5.83E-04	S316;S320;S321;	8.15E-04	S316;S320;S321;	9.37E-04	6.22E-01	8.70E-01
Q9NYB0	TERF2IP	S203;	1.42E-04	S203;	1.11E-04	S203;	1.64E-04	8.67E-01	6.77E-01
Q9NYB9	ABI2	S183;S227;	9.88E-05	NA	NA	NA	NA	NA	NA
Q9NYF8	BCLAF1	S397;S264;S268;S222;S385;S177;S297;S496;S760;S183;S102;S104;S290;Y284;S512;S285;S658;S119;S122;S450;S531;S648;S17;S19;S20;T726;S763;T257;S759;S262;S23;S25;S27;T661;T402;S757;S161;S717;S718;	9.28E-03	S397;T402;S385;S177;S512;S281;S290;S496;S222;S183;S757;S759;T257;S268;S658;S272;S102;S104;S161;S531;S297;S264;Y284;S259;S760;S274;S660;S717;S718;	1.00E-02	S397;T402;S222;S177;S385;S290;S512;S183;Y511;T257;S264;S102;S104;S658;S285;S660;S119;S122;S496;S531;T726;Y383;S268;S181;Y284;S648;S760;S389;	1.18E-02	7.89E-01	8.52E-01

Q9NYV4	CDK12	S681;S685;S274;S276;S383;S385;S423;S323;S325;T692;S334;S1083;S357;S359;S338;S341;S343;S1082;S345;Y319;Y327;	4.92E-04	S681;S685;S274;S276;S236;S251;S383;S385;S343;S345;S334;S357;S359;S382;S332;S333;S1083;S320;S323;S325;	8.66E-04	S274;S276;S383;S385;S681;S685;S14;S18;T20;S1083;S355;S357;S359;S334;S301;S303;S1082;S423;	4.08E-04	1.21E+00	2.12E+00
Q9NYZ3	GTSE1	S592;S575;S547;	6.70E-05	S592;S575;S583;S536;	2.27E-05	S477;S592;S575;S580;S138;S141;	1.91E-05	3.50E+00	1.18E+00
Q9NZ56	FMN2	S450;S452;S1361;	4.75E-05	NA	NA	NA	NA	NA	NA
Q9NZ63	C9orf78	S15;S17;S261;	2.02E-03	S15;S17;S261;	5.95E-04	S15;S17;S261;	2.38E-03	8.48E-01	2.50E-01
Q9NZI8	IGF2BP1	S181;	7.82E-04	S181;	2.27E-03	S181;	3.48E-04	2.25E+00	6.53E+00
Q9NZN8	CNOT2	S165;	4.60E-05	NA	NA	NA	NA	NA	NA
Q9NZT2	OGFR	S378;S315;	8.13E-05	S378;S315;S349;	3.69E-04	S378;S315;S484;S349;S468;S473;	6.59E-04	1.23E-01	5.60E-01
Q9P0P8	C6orf203	S106;S116;	4.33E-05	NA	NA	NA	NA	NA	NA
Q9P1Y6	PHRF1	S1371;T917;S973;S1124;S1202;S1127;S1360;S1359;S1163;S1165;S1167;S1116;S1114;	4.35E-04	S915;S1163;S1165;S1114;S1116;T917;S1124;S589;T591;	2.43E-04	T981;S1360;S1359;S1124;S1032;S1034;S1202;	7.71E-05	5.64E+00	3.16E+00
Q9P206	KIAA1522	S979;S342;S545;S669;S673;	1.69E-04	S669;S673;S929;S545;S342;	1.52E-04	S858;S862;S620;S979;S669;S673;S929;S545;S562;S342;	2.39E-04	7.05E-01	6.33E-01
Q9P260	KIAA1468	NA	NA	S180;T183;S186;	1.38E-05	S180;T183;S186;S453;	1.75E-05	NA	7.88E-01
Q9P275	USP36	S610;S613;S618;S742;S614;S612;	9.15E-05	S742;	4.05E-05	S610;S613;S618;S614;S742;S611;S612;S582;	1.37E-04	6.69E-01	2.96E-01
Q9P2D1	CHD7	NA	NA	S2956;S2983;S2559;	3.07E-05	NA	NA	NA	NA
Q9P2E9	RRBP1	S615;T225;S1277;S583;S533;	6.75E-05	S615;S1277;S583;S575;S533;T225;S1403;S1276;	8.21E-04	S615;S1277;	7.21E-05	9.36E-01	1.14E+01
Q9P2G1	ANKIB1	NA	NA	S737;	1.07E-05	S737;	1.50E-05	NA	7.11E-01
Q9P2I0	CPSF2	S419;S420;S423;	8.91E-05	S419;S420;S423;	7.00E-04	S419;S420;S423;	5.74E-04	1.55E-01	1.22E+00
Q9P2P5	HECW2	NA	NA	NA	NA	S48;	2.20E-05	NA	NA
Q9P2R6	RERE	S613;S679;S656;S615;S1106;S1113;S1115;	4.20E-05	S53;S56;S1106;S1113;S1115;S656;S594;T599;	5.66E-05	S613;S348;S53;S56;S1106;S1113;S1115;S656;T658;	5.79E-05	7.25E-01	9.77E-01
Q9UBB5	MBD2	S407;	4.09E-05	NA	NA	NA	NA	NA	NA
Q9UBB9	TFIP11	S98;S210;S59;	2.18E-04	S98;S59;	9.99E-05	S98;S59;	4.06E-04	5.37E-01	2.46E-01

Q9UBC2	EPS15L1	S255;S793;	4.83E-05	S255;S229;S253;	5.88E-05	S255;S229;S793;	5.69E-05	8.48E-01	1.03E+00
Q9UBF8	PI4KB	S428;	4.04E-05	S428;	2.28E-04	S428;S511;	2.33E-04	1.74E-01	9.81E-01
Q9UDY2	TJP2	S1159;S170;S174;S244;S986;S966;S266;S441;S398;S400;S292;S294;S296;S961;	1.91E-04	S398;S400;S130;T925;S1067;S1068;S170;S174;S244;S978;S986;T933;S292;S294;S296;S394;S1159;S961;S266;Y423;Y428;S163;S966;T1027;S979;S702;	6.63E-03	S130;S1159;S1068;S986;S398;S400;S244;T925;T933;S170;S174;S266;S292;S294;S296;S966;S163;	9.32E-04	2.05E-01	7.11E+00
Q9UER7	DAXX	S495;S737;S739;	4.26E-05	S737;S739;S671;S668;S702;S688;S178;	2.14E-04	S671;S178;S737;S739;S668;S675;S702;S688;	1.03E-04	4.15E-01	2.08E+00
Q9UEY8	ADD3	S681;S673;S679;S693;S677;	1.21E-04	S683;S681;S673;S677;S679;S461;	1.15E-03	S673;S677;S681;	2.60E-05	4.64E+00	4.44E+01
Q9UFC0	LRWD1	S212;	6.31E-05	S253;S259;S212;S251;	2.41E-04	S253;S259;S212;S251;S243;	2.10E-04	3.01E-01	1.15E+00
Q9UGN5	PARP2	S33;Y260;T262;	6.74E-05	NA	NA	NA	NA	NA	NA
Q9UGU0	TCF20	S574;S583;S1335;S559;	7.00E-05	S574;S583;S1335;	2.31E-05	S574;S583;Y1533;S1322;T1671;	3.25E-05	2.15E+00	7.11E-01
Q9UGV2	NDRG3	NA	NA	S361;S331;	1.27E-04	NA	NA	NA	NA
Q9UH62	ARMCX3	NA	NA	S61;S70;	1.06E-04	S61;	6.89E-05	NA	1.54E+00
Q9UHB6	LIMA1	S225;S686;S604;S698;S374;S132;S582;S490;S609;S369;S371;S583;S362;S365;S692;	1.18E-02	S362;S365;S374;S490;S132;S698;S582;S583;S373;S609;	2.01E-03	S686;S225;S362;S726;S374;S490;S698;S132;S609;S582;S583;S228;Y229;S365;S369;S371;	4.30E-03	2.73E+00	4.68E-01
Q9UHB7	AFF4	S549;S487;S490;S491;S1043;S180;	8.03E-05	S703;S706;S1062;S1043;	5.09E-05	S1043;S487;S703;S706;S549;	4.70E-05	1.71E+00	1.08E+00
Q9UHD8	42987	S30;S82;	1.01E-04	S30;	9.01E-04	S30;T42;T38;S80;S327;S85;	5.43E-04	1.85E-01	1.66E+00
Q9UHF7	TRPS1	NA	NA	NA	NA	S216;S1041;	7.92E-06	NA	NA
Q9UHI6	DDX20	S677;S678;	3.88E-05	NA	NA	S677;S678;S703;	6.46E-05	6.00E-01	NA
Q9UHR5	SAP30BP	S18;S22;S69;S72;	3.94E-05	S18;S22;S33;S72;S77;	5.65E-05	S72;S77;S18;S22;S43;S69;Y14;S33;	6.00E-05	6.56E-01	9.41E-01
Q9UI10	EIF2B4	NA	NA	NA	NA	S130;	3.70E-05	NA	NA
Q9UID6	ZNF639	NA	NA	NA	NA	S60;	1.70E-05	NA	NA
Q9UIF9	BAZ2A	T1399;S1770;S1397;	7.26E-05	S1783;	1.12E-05	NA	NA	NA	NA
Q9UIG0	BAZ1B	S158;S1468;	5.51E-05	S1468;S705;S708;S1342;S158;	6.58E-04	S708;T710;S1468;S947;S705;S158;S161;S16	4.98E-04	1.11E-01	1.32E+00

						0;			
Q9UJF2	RASAL2	S864;	2.94E-05	NA	NA	S89;S864;	2.30E-05	1.28E+00	NA
Q9UJK0	TSR3	NA	NA	NA	NA	S282;	4.50E-06	NA	NA
Q9UJU6	DBNL	S232;S275;	8.37E-05	NA	NA	S232;	6.61E-05	1.27E+00	NA
Q9UK58	CCNL1	S352;	3.47E-05	S352;S329;T330;	8.70E-05	S335;S338;S342;S352;	4.23E-04	8.20E-02	2.06E-01
Q9UK59	DBR1	NA	NA	S514;	2.83E-05	S514;	4.08E-05	NA	6.94E-01
Q9UK76	HN1	S131;S87;T54;S88;S70;S92;	2.14E-03	S131;S87;S88;S70;	1.83E-03	S131;S87;S88;S70;S31;T54;S91;S92;	1.27E-03	1.69E+00	1.45E+00
Q9UKJ3	GPATCH8	S1076;S1081;S814;S815;S820;S740;S1035;S1107;S653;S985;S987;	2.23E-04	S1076;S1081;S740;S1107;S814;S815;S820;Y988;S989;S985;S987;S1087;S1035;	2.17E-04	S1076;S1081;S1107;S1035;S1009;S1014;S650;S758;S1087;S738;S740;	1.81E-04	1.23E+00	1.19E+00
Q9UKL0	RCOR1	NA	NA	S257;	1.43E-04	S257;	3.06E-04	NA	4.66E-01
Q9UKM9	RALY	T286;S288;S135;T298;	1.12E-03	T298;S288;S135;T286;S295;	4.40E-03	T286;T298;S288;S135;	4.53E-04	2.48E+00	9.71E+00
Q9UKS6	PACSIN3	S354;S341;S358;	6.07E-04	S344;S354;S276;T347;	4.38E-04	S354;S319;S344;	3.70E-04	1.64E+00	1.18E+00
Q9UKV3	ACIN1	S216;T682;S240;S243;S490;S838;S410;T414;S1004;S386;S388;S478;S210;S1329;S1331;T1332;S710;S714;S384;S655;S657;T393;S729;T408;S208;S365;S605;	5.99E-03	S240;S243;S838;S216;S490;S410;S1004;S714;S1329;S1331;T1332;S655;S657;S729;S384;S386;S388;S825;T414;S208;S365;	3.11E-03	S240;S243;S208;S216;S490;T254;S410;T414;S838;S714;S655;S657;S1004;S386;S388;S898;S561;T393;S729;S1329;S1331;T1332;S384;S365;S710;T563;S206;T408;	4.86E-03	1.23E+00	6.40E-01
Q9ULF5	SLC39A10	NA	NA	NA	NA	S591;S546;	3.21E-05	NA	NA
Q9ULH7	MKL2	T227;T217;T215;S225;S543;S834;	1.08E-04	NA	NA	NA	NA	NA	NA
Q9ULL5	PRR12	NA	NA	S558;S560;S561;T740;S747;	5.06E-05	S560;S561;	3.89E-05	NA	1.30E+00
Q9ULM3	YEATS2	S519;	2.10E-05	NA	NA	NA	NA	NA	NA
Q9ULT8	HECTD1	NA	NA	S357;S632;	5.35E-05	S632;S1380;S357;	1.79E-04	NA	2.99E-01
Q9ULU4	ZMYND8	S490;S668;S655;S486;S488;	2.73E-04	S490;S486;S668;S655;	1.88E-04	S486;S488;S490;S655;S652;S547;S668;	2.18E-04	1.26E+00	8.63E-01
Q9ULW0	TPX2	S738;S121;S125;S186;	4.29E-04	S738;S292;S186;S121;S125;S293;	8.66E-04	S738;	1.75E-04	2.45E+00	4.94E+00

Q9ULX3	NOB1	S201;S184;	4.62E-04	S201;S184;	5.77E-04	S184;S201;	8.89E-04	5.20E-01	6.49E-01
Q9ULX6	AKAP8L	S283;T294;S300;S302;	1.95E-04	S300;S302;S283;	2.12E-05	S302;T308;S283;	7.50E-05	2.60E+00	2.82E-01
Q9UMN6	KMT2B	NA	NA	S1032;S1035;S1039;S861;	1.83E-05	S1032;S1035;S1039;T1026;	1.17E-05	NA	1.56E+00
Q9UMZ2	SYNRG	S473;	4.76E-05	S473;S1075;	8.09E-05	S1006;S473;S752;S1075;	1.28E-04	3.72E-01	6.32E-01
Q9UN37	VPS4A	S95;S97;S99;	1.93E-05	S95;S97;S99;	5.68E-05	S95;S97;S99;	1.99E-05	9.67E-01	2.85E+00
Q9UN86	G3BP2	S141;S149;T227;	1.70E-04	S141;S149;	5.03E-04	S141;S149;	6.42E-04	2.64E-01	7.83E-01
Q9UNE7	STUB1	S19;S23;	8.08E-04	S19;	4.01E-04	S19;S23;	9.49E-04	8.51E-01	4.22E-01
Q9UNF0	PACSIN2	S399;	1.01E-04	S399;S403;T424;	1.22E-04	S403;S399;	1.00E-04	1.00E+00	1.21E+00
Q9UNF1	MAGED2	NA	NA	S190;S191;S264;	5.07E-06	S190;S191;	2.07E-05	NA	2.45E-01
Q9UNZ2	NSFL1C	S272;S140;	4.93E-05	S272;S114;	9.29E-05	S272;S114;	6.28E-05	7.85E-01	1.48E+00
Q9UPN3	MACF1	S4521;	3.10E-05	S4521;S7330;	1.60E-05	S4521;S2451;S7292;T5594;	8.82E-05	3.52E-01	1.82E-01
Q9UPN4	CEP131	S78;S381;	7.32E-05	S47;S78;	3.03E-05	NA	NA	NA	NA
Q9UPN6	SCAF8	S617;	9.68E-05	T619;S617;	6.69E-05	S617;	4.41E-05	2.20E+00	1.52E+00
Q9UPN7	PPP6R1	NA	NA	S635;S638;	2.84E-05	S635;S638;S664;S667;T668;S670;S759;S726;	9.53E-05	NA	2.98E-01
Q9UPN9	TRIM33	NA	NA	T1102;S1105;	3.29E-04	S1119;T1102;S1105;S862;	3.31E-04	NA	9.93E-01
Q9UPP1	PHF8	S857;	3.57E-05	S854;S857;S1021;	3.55E-05	S857;S854;S1021;S880;	9.34E-06	3.82E+00	3.80E+00
Q9UPQ0	LIMCH1	S523;S718;S231;S233;S226;S469;S973;S217;T215;S670;S493;	7.12E-04	NA	NA	S201;S204;S225;S233;S231;S718;S226;S207;T672;S377;S973;T215;S217;S680;S875;S670;S192;	6.60E-04	1.08E+00	NA
Q9UPQ9	TNRC6B	S561;S567;S1832;	5.61E-06	NA	NA	NA	NA	NA	NA
Q9UPT8	ZC3H4	S1114;S1275;S807;S808;S1108;T1265;S908;T1106;S1269;	5.43E-04	S1114;S1275;S1269;T1106;S1108;S807;S808;S1104;S908;T1118;Y1121;	4.94E-04	S807;S808;S1114;S1275;S1269;T1106;S1108;Y806;S92;S94;S908;	2.58E-04	2.11E+00	1.92E+00
Q9UPW0	FOXJ3	S223;	2.45E-05	NA	NA	NA	NA	NA	NA
Q9UQ35	SRRM2	S377;S398;S1387;T2316;S1648;S295;S297;S857;S950;S952;S954;T983;S994;S1497;S1499;S1502;T384;T8	1.03E-01	S377;S398;S1383;S1387;T1003;S1014;S295;S297;S351;S353;S358;S1064;S1069;S1497;S1499;S1502	6.52E-02	S377;S398;S1387;S1320;S1326;S1329;T384;T1003;S1014;S322;S323;S950;S952;S954;S3	6.71E-02	1.54E+00	9.71E-01

		56;S1818;S1822;S1824;T866;S1404;S2272;S1542;S1552;S1579;S1581;S1582;T1003;S1014;S1384;S435;S436;S437;S1598;S1600;S1601;S1691;S1693;S1694;T1413;T848;S972;S973;S974;S875;S876;S1420;S1421;S1415;S1401;S741;S743;S746;T2738;S2740;S346;S351;S353;S562;S564;S566;S2702;S2706;S1864;S1866;S1869;S440;T1231;S1441;S1443;S1444;S2100;S1482;S1483;S1762;S1764;S778;S780;S783;T1453;S1923;S1925;T1927;S1975;T2409;S2102;T2104;S2407;S2412;S2042;S2044;S2046;T1071;S1072;S1073;S2382;S854;S506;S508;S510;S2121;S2123;S1103;S322;S323;S901;T903;S908;S1458;S1460;S988;S990;S1517;S1519;S1320;S2581;S1616;S1620;S1621;S2415;S1179;S2018;S2020;T2022;S2132;S1462;S1463;S2067;T2069;S2071;S1318;S1329;S2690;S2692;S2694;S1972;S1857;S1854;S895;S1132;S2030;S2032;T2034;S1124;S2449;S892;S894;S2398;S838;S1888;S1890;S1101;T1106;S1982;S1984;S1987;S1478;T1531;S472;S478;S1893;S474;S864;S2684;S1112;T1434;T476;S534;S536;S191		;T384;S854;S857;S1099;S1101;S950;S952;S954;S2100;T2104;S322;T326;S300;S957;S1318;S1320;S1329;S846;S323;S2690;S2692;S1539;S1552;S974;S1478;S1482;S1483;S876;S778;S780;T866;S435;S436;S562;S564;S566;S2702;T983;T2289;S1188;S2067;T2069;S2071;S2706;S1600;S1601;S2449;S437;S1579;S1581;S1582;S1441;S1913;S1916;S204;S1864;S1866;S1869;S1598;S972;S973;S2398;S2030;S2032;T2034;S2018;S2020;T2022;S506;S508;S510;S1923;S1925;T1927;S1103;S1110;S1672;S1674;S1675;S2581;S1384;S2042;S2044;S2046;S1693;S1694;S1935;S1937;T1939;S1975;S1124;S1444;S1762;S1764;S1083;T1531;S2694;S472;S474;S478;S2688;S575;T577;S1691;S346;S2102;S625;T627;S629;S1984;T1986;S2412;S2675;S2677;S2407;S819;S817;S818;S783;S395;S713;S715;T717;S2090;T2092;S1888;S1890;S484;S486;T1856;S1857;T848;S1747;S1749;S1750;S1682;T1684;S1911;T903;S908;S1902;S1905;S702;S704;S706;S1893;T1904;S901;S573;S		51;S353;S1099;S1101;S1102;S1579;S1581;S1582;S1497;S1499;S1502;S875;S876;S1818;S1824;S973;S974;S2449;S2272;S295;S297;S1691;S1693;S1694;S1864;S1866;S1869;S435;S436;S437;S1152;S1064;S1069;T1071;S2690;S2692;S2694;S1482;S1483;S846;T848;S1103;S1542;S1550;S2453;S1539;S1552;S2702;S2706;S1403;S2100;S2102;T2104;S857;S506;S508;S510;S778;S780;S2067;T2069;S2071;S1762;S1764;S901;S908;S910;S1916;S1404;T983;S988;S1441;S1443;S1444;S2030;S2032;T2034;S1923;S1925;T1927;S1987;S440;S1318;S2044;S2046;T2316;T2329;S1188;S1478;S1179;S2407;S2412;S892;T2289;S2398;S2727;S566;T569;T2409;S2581;S472;S474;T476;S2020;T2022;T903;S1112;S2042;S713;S715;T717;S1598;S1600;S248;S250;S2018;S534;S536;S1888;S1890;S783;T866;S1682;S1672;S2688;S1124;S1911;S1913;T1856;S1857;S			

		1;S1913;S1916;S992;S454;S455;S456;S713;S715;T717;T1698;T810;T1205;T1472;S2727;S2731;S1423;S1424;T1856;S883;S887;S808;S1900;S1902;S1905;S543;S761;S763;S764;S1970;S1876;S1878;T1880;S702;S704;S706;T1974;S1682;T1684;S1672;S1674;S1675;S625;S629;S573;S575;T577;S834;S839;S231;T233;S1727;S1729;S1731;T569;S357;S1382;S1061;S1069;S387;S1099;S1326;S1102;T428;S1064;S1008;S818;S248;S250;S1088;S1732;S819;S820;S486;S1110;S957;S2426;S2675;S2677;T1986;T489;S594;S596;T598;S890;T627;S759;S846;S300;S1383;S358;S395;T326;Y1820;S2115;S2456;S1152;S1398;S910;S1233;T2289;S484;S1188;S1083;S1852;S817;S2090;T2092;S1935;S1937;T1939;T1208;S200;S204;S1842;T1844;T1847;S2688;T252;S1791;S1793;S1796;S1794;S1081;T1904;S902;S745;S220;S222;S525;S527;		543;S248;S250;T252;S594;S596;T598;S455;S456;S1382;S1326;S387;S875;S1542;S1102;S2453;T856;S1219;Y996;T2316;S892;S1690;S1443;S1987;S534;S536;T1698;S200;S202;S1896;S2415;T2409;S910;T251;S759;S761;S763;S764;S1982;S839;S1112;S895;T489;S808;T810;S1822;S1824;T1071;S440;S988;S2417;T476;S1179;S820;S1970;S1972;S1517;S1854;		1601;S864;S782;S1517;S1519;S1521;S1522;S1893;S562;S564;S1970;S1972;S455;S456;T1531;S808;T810;S1984;S1982;S1012;S817;S231;T233;T885;S895;S1822;T1434;S573;T1990;S1415;S1541;T326;S957;S1501;S854;S2382;S1073;S2456;S1975;T1974;S1083;S2729;S2132;S887;S890;T251;T252;S1110;S702;S704;S706;S972;S454;S834;S759;S761;S1382;Y1820;T359;S395;S1690;T856;S346;S1085;S1383;Y996;S902;S2415;S311;S2675;S2677;S478;S1937;T1939;S2426;T1986;S1674;S1675;S1935;S575;T577;S1577;S1010;T1844;S1848;T1684;S484;S486;			
Q9UQ88	CDK11A	S271;	2.92E-05	S265;S271;S217;S222;S577;T583;T739;	1.67E-04	S271;S265;T739;	7.72E-05	3.78E-01	2.16E+00
Q9UQB8	BAIAP2	S325;	1.01E-05	NA	NA	NA	NA	NA	NA
Q9UQE7	SMC3	S1067;	3.37E-05	S1067;	3.68E-05	S1067;	2.63E-05	1.28E+00	1.40E+00
Q9UQN3	CHMP2B	S199;	4.91E-05	S199;	1.10E-04	S199;	1.28E-04	3.84E-01	8.56E-01
Q9UQR1	ZNF148	T789;S665;S784;S412;S306;	9.77E-05	NA	NA	NA	NA	NA	NA

Q9Y232	CDYL	S129;S201;	4.11E-05	NA	NA	S216;S129;S201;T128;	5.96E-05	6.90E-01	NA
Q9Y266	NUDC	T108;S139;T145;	2.76E-05	S139;T145;T108;	6.17E-05	S139;T108;S326;T145;	1.15E-04	2.39E-01	5.34E-01
Q9Y2D5	AKAP2	S393;S778;S152;S748;S121;	5.55E-04	S393;S778;S159;S748;S152;	5.93E-04	S393;S152;T131;S748;S778;S135;	8.20E-04	6.76E-01	7.23E-01
Q9Y2F5	ICE1	NA	NA	NA	NA	S1891;S1903;S1854;S925;S255;S1692;S1699;	6.64E-05	NA	NA
Q9Y2K7	KDM2A	T713;S718;S731;T720;S721;S692;	4.58E-05	S869;	1.16E-04	S869;Y22;S28;	4.48E-05	1.02E+00	2.58E+00
Q9Y2V2	CARHSP1	S30;S32;T45;S41;	3.35E-03	S30;S32;S41;S52;	1.04E-02	S30;S32;S41;T45;	2.05E-03	1.63E+00	5.09E+00
Q9Y2W1	THRAP3	S740;S743;S746;S939;S51;S53;S55;S928;S320;S698;S672;S379;S315;S243;S248;S253;S682;S406;S408;Y54;S533;S119;S134;S136;T941;S34;S36;S38;S917;S753;	5.01E-03	S928;S939;S248;S257;T941;S243;S315;S379;S672;S320;S51;S55;S682;S134;S136;S753;S34;S36;S40;S253;S406;S408;S53;	4.92E-03	S928;S939;T941;S51;S53;S55;S406;S408;S248;S253;S320;S243;S672;S379;S315;S682;S740;S743;S746;S399;S134;S136;S34;S36;S38;Y54;S575;S176;S753;S326;	5.08E-03	9.86E-01	9.68E-01
Q9Y2W2	WBP11	S353;S361;S364;S600;S237;	7.83E-04	S353;S361;S364;	3.81E-05	S353;S361;S364;	5.16E-05	1.52E+01	7.39E-01
Q9Y2X3	NOP58	S502;S514;	4.15E-04	S502;S514;T508;	2.27E-03	S502;S514;	1.53E-03	2.72E-01	1.48E+00
Q9Y2X7	GIT1	S388;S592;S596;S385;T392;	3.54E-05	S385;S388;S592;T601;Y383;T392;	1.34E-04	Y383;S388;S592;S596;Y598;S362;S385;	3.08E-04	1.15E-01	4.34E-01
Q9Y383	LUC7L2	S323;S327;S336;S281;S283;S285;S358;S354;S383;S384;	2.09E-04	S336;S358;	7.87E-06	S336;S323;S327;S383;S384;S354;S358;	3.19E-05	6.56E+00	2.46E-01
Q9Y388	RBMX2	S188;	1.50E-04	S188;	1.14E-04	S188;	1.79E-04	8.42E-01	6.41E-01
Q9Y3B9	RRP15	S276;S278;T282;	4.44E-05	NA	NA	S276;S278;T282;S280;	3.71E-05	1.20E+00	NA
Q9Y3E7	RNF103-CHMP3	S200;	2.88E-05	NA	NA	NA	NA	NA	NA
Q9Y3T9	NOC2L	S49;S60;S672;S673;S56;S26;S28;S30;S58;	5.19E-04	S672;S673;S60;S49;S30;S32;S26;S28;S56;	2.54E-03	S672;S22;S26;S32;S28;S673;S56;S49;S60;S58;S30;	2.54E-03	2.04E-01	1.00E+00
Q9Y3X0	CCDC9	S386;S390;T381;T385;T95;	7.75E-05	S390;S80;S386;S521;	1.69E-04	S386;S521;T385;S390;S202;T95;	8.03E-05	9.66E-01	2.10E+00
Q9Y446	PKP3	S238;S314;	9.83E-05	NA	NA	S313;S314;S240;	2.21E-04	4.45E-01	NA
Q9Y463	DYRK1B	Y273;	1.29E-05	Y273;	8.91E-05	Y273;	9.55E-05	1.35E-01	9.33E-01

Q9Y478	PRKAB1	NA	NA	NA	NA	S108;S181;	4.27E-05	NA	NA
Q9Y4A5	TRRAP	NA	NA	NA	NA	S2077;	4.65E-05	NA	NA
Q9Y4B4	RAD54L2	S1169;S1172;T411;	5.16E-05	S1169;S1172;S744;	2.25E-05	S1169;S1172;	3.31E-05	1.56E+00	6.79E-01
Q9Y4E1	FAM21C	S498;S333;S539;T331;	1.17E-04	S158;S160;S498;S544;S333;S56;S539;S356;S352;	9.04E-05	S539;S498;S352;T331;S333;S158;S160;S441;S284;	2.02E-04	5.77E-01	4.47E-01
Q9Y4F5	CEP170B	S829;S772;S421;S1545;S1548;	2.63E-05	S1545;S1548;S1551;T358;S785;S796;S809;	4.78E-05	S1135;S1548;S853;S772;S619;S1545;	3.71E-05	7.08E-01	1.29E+00
Q9Y4K1	AIM1	S75;S81;S102;S78;	2.95E-05	NA	NA	S102;S16;S19;T20;S75;	1.22E-05	2.42E+00	NA
Q9Y4K4	MAP4K5	NA	NA	S433;S362;T379;	7.83E-05	NA	NA	NA	NA
Q9Y520	PRRC2C	T1498;S1503;S878;S2143;S2013;S2105;T2682;S187;S2694;T887;S920;S2685;	3.49E-04	S878;S2013;S926;S2105;S1249;S2694;T2682;S2685;S920;T1498;S1503;S187;S929;T1267;S1269;S1274;	4.70E-04	S187;S191;S2013;S2105;T2673;T2682;S2685;S878;S924;S1489;S926;S2143;S1249;T1498;S1500;S1503;T2146;	3.31E-04	1.06E+00	1.42E+00
Q9Y580	RBM7	S107;S204;S136;	3.52E-04	S136;	1.11E-04	S136;	2.04E-04	1.73E+00	5.47E-01
Q9Y5B6	PAXBP1	S154;S155;S295;	8.86E-06	NA	NA	S262;	1.25E-05	7.09E-01	NA
Q9Y5J1	UTP18	S121;S124;S206;S210;	5.82E-04	S206;S210;S121;S124;S205;	1.28E-03	S121;S124;S206;S210;S205;	1.44E-03	4.05E-01	8.89E-01
Q9Y5K6	CD2AP	S510;S514;	9.64E-06	S542;S510;T539;	3.74E-05	S514;S510;	3.50E-05	2.75E-01	1.07E+00
Q9Y5Q9	GTF3C3	S43;	3.43E-05	S43;	1.12E-04	S43;	1.28E-04	2.69E-01	8.79E-01
Q9Y5S2	CDC42BPB	S1690;	6.90E-05	NA	NA	S1690;S1693;	8.95E-05	7.71E-01	NA
Q9Y5S9	RBM8A	S56;S42;S166;S168;	1.82E-04	S42;S166;S168;	2.74E-04	S42;S166;S168;	8.71E-05	2.09E+00	3.14E+00
Q9Y5T5	USP16	NA	NA	S415;	2.65E-05	S415;	4.26E-05	NA	6.22E-01
Q9Y613	FHOD1	S367;S523;	2.28E-05	NA	NA	S367;	1.69E-05	1.35E+00	NA
Q9Y618	NCOR2	S746;S750;S753;T553;S554;S922;S2269;S2234;S2065;S2205;S2208;S2214;S1018;S1025;S956;S149;S152;S1786;S1181;T756;S2057;S2068;S2069;T550;S1783;S1982;S215;S2016;T1391;S1259;	7.98E-04	NA	NA	S2065;S2069;S1025;S922;S2269;S2016;S1786;T553;S554;S149;S152;S215;S956;T1391;S1181;S1487;S1432;S2055;S1018;S2214;S2205;S2208;S746;S750;S753;S1783;S939;S943;T1569;	7.38E-04	1.08E+00	NA

Q9Y6A5	TACC3	S434;S71;S317;	5.32E-05	S434;S250;S25;	9.30E-05	S434;S25;S250;	7.04E-05	7.56E-01	1.32E+00
Q9Y6D5	ARFGEF2	S218;S227;	5.51E-05	S218;S227;	5.94E-04	S218;S227;T1588;T1597;S1528;	2.74E-04	2.01E-01	2.17E+00
Q9Y6D9	MAD1L1	S428;	3.57E-05	NA	NA	S428;	2.81E-05	1.27E+00	NA
Q9Y6G9	DYNC1LI1	S510;T513;S516;T389;S207;S419;	2.62E-04	S207;T513;S516;S510;T512;T515;	8.33E-04	S516;S421;T513;S207;S398;S414;	1.69E-03	1.55E-01	4.93E-01
Q9Y6I3	EPN1	S454;S435;	3.95E-05	NA	NA	S454;T470;	3.45E-05	1.14E+00	NA
Q9Y6K1	DNMT3A	NA	NA	NA	NA	S105;	2.84E-05	NA	NA
Q9Y6M1	IGF2BP2	T243;T247;T251;S164;	2.27E-05	S162;S164;	3.58E-04	S162;S164;	2.85E-05	7.96E-01	1.26E+01
Q9Y6X9	MORC2	NA	NA	S777;S779;S743;S615;S725;T733;S773;	3.43E-04	S777;S779;S615;S711;S743;	7.57E-05	NA	4.53E+00
O00401	WASL	S484;S485;	5.03E-05	S484;S485;	1.53E-04	S484;S485;	1.16E-04	4.32E-01	1.31E+00
O14686	KMT2D	S3202;T3197;S3199;T2639;	2.19E-05	S1294;S1293;S1298;	4.87E-05	T3197;S3199;S4738;S1671;S2274;S2296;	6.16E-05	3.55E-01	7.91E-01
O15226	NKRF	S429;	3.79E-05	NA	NA	NA	NA	NA	NA
O15357	INPPL1	NA	NA	NA	NA	S158;S132;	1.45E-05	NA	NA
O43164	PJA2	NA	NA	NA	NA	S253;S308;	2.11E-05	NA	NA
O43318	MAP3K7	NA	NA	S439;Y33;	2.56E-04	S439;	1.16E-04	NA	2.20E+00
O43683	BUB1	NA	NA	S655;S661;S370;T392;S396;	2.77E-05	S596;T589;S593;S655;S661;S381;	1.19E-04	NA	2.33E-01
O43823	AKAP8	NA	NA	S323;S328;S339;	2.51E-04	S323;S328;S339;	1.97E-04	NA	1.27E+00
O60271	SPAG9	S730;S733;S329;S332;S251;	4.86E-05	S183;S730;S732;S251;S728;S329;S332;	2.53E-04	S183;S185;S730;S732;S728;S329;S332;S733;	2.49E-04	1.95E-01	1.02E+00
O60927	PPP1R11	S73;T109;	4.27E-05	T75;S77;	2.72E-05	S73;S74;S77;	3.63E-05	1.18E+00	7.49E-01
O75446	SAP30	S131;S138;	1.91E-04	S131;S138;	1.92E-05	NA	NA	NA	NA
O75821	EIF3G	T41;T38;	6.38E-04	T41;S42;T38;	3.77E-04	T41;T38;S42;	1.51E-03	4.22E-01	2.50E-01
O95239	KIF4A	NA	NA	S1225;S801;	8.73E-06	NA	NA	NA	NA
O95677	EYA4	NA	NA	NA	NA	S361;	2.74E-05	NA	NA
O95999	BCL10	S138;	9.17E-05	NA	NA	S138;	1.12E-04	8.20E-01	NA
P00533	EGFR	NA	NA	S1039;S1042;T1041;S1166;Y1069;T693;S1037;	1.57E-04	S1039;S1042;S1166;T693;Y1069;S1045;S1037;T1041;	2.17E-04	NA	7.24E-01
P02795	MT2A	S32;S58;	2.65E-05	NA	NA	NA	NA	NA	NA
P05386	RPLP1	NA	NA	S101;S104;	8.26E-03	S101;S104;	6.68E-03	NA	1.24E+00
P0DMV8	HSPA1A	NA	NA	NA	NA	S631;	1.32E-04	NA	NA

P13645	KRT10	S16;S573;	1.91E-04	NA	NA	NA	NA	NA	NA
P14859	POU2F1	S448;	1.50E-04	NA	NA	NA	NA	NA	NA
P17480	UBTF	NA	NA	NA	NA	S484;	1.73E-05	NA	NA
P17812	CTPS1	S574;S575;	1.71E-04	S575;S574;S573;S578;S568;	1.67E-04	S574;S575;S571;S573;S578;S562;	1.20E-03	1.42E-01	1.40E-01
P19634	SLC9A1	S785;	1.28E-05	NA	NA	NA	NA	NA	NA
P22466	GAL	S117;S116;	4.18E-05	NA	NA	S117;	5.21E-05	8.02E-01	NA
P25440	BRD2	S298;S301;S633;	5.63E-05	S298;S301;	4.34E-05	NA	NA	NA	NA
P25685	DNAJB1	S151;	1.06E-04	S151;	8.91E-05	NA	NA	NA	NA
P26599	PTBP1	T138;S141;	1.85E-04	S141;	1.15E-04	S141;	9.18E-05	2.02E+00	1.26E+00
P28066	PSMA5	S16;	1.11E-05	NA	NA	NA	NA	NA	NA
P35606	COPB2	S859;	3.60E-04	S859;	2.00E-03	S859;	2.12E-03	1.70E-01	9.43E-01
P36915	GNL1	S51;S32;S33;S34;	7.39E-05	T48;T50;S51;S55;	5.50E-04	S51;T50;T48;T57;	3.41E-05	2.17E+00	1.62E+01
P42684	ABL2	S817;S820;	1.69E-05	S817;S820;S620;S631;	5.64E-05	S936;S820;S631;S620;S819;	5.33E-05	3.17E-01	1.06E+00
P42694	NA	NA	NA	NA	NA	NA	NA	NA	NA
P49450	CENPA	NA	NA	S17;S19;T21;	2.74E-04	S17;T21;	5.58E-05	NA	4.92E+00
P49750	YLPM1	S924;S634;	9.76E-06	S924;	1.18E-06	S634;S561;S924;	2.13E-05	4.59E-01	5.56E-02
P51114	FXR1	S406;S409;	8.03E-05	S409;S587;S448;S450;	9.94E-05	S406;S409;S420;S423;T483;S587;	1.34E-04	5.98E-01	7.41E-01
P51531	SMARCA2	S1512;S1516;S1528;S1377;	2.11E-05	NA	NA	NA	NA	NA	NA
P51825	AFF1	NA	NA	S199;S206;S212;	3.46E-05	S206;S212;S634;S199;T220;	1.64E-05	NA	2.11E+00
P53621	COPA	NA	NA	S173;	4.73E-05	S173;S402;	9.62E-05	NA	4.92E-01
P55199	ELL	NA	NA	NA	NA	S442;S437;	3.46E-05	NA	NA
P58012	FOXL2	S33;	3.54E-05	NA	NA	NA	NA	NA	NA
P61006	RAB8A	NA	NA	NA	NA	S181;S185;	1.54E-05	NA	NA
P78344	EIF4G2	T508;	5.82E-05	T508;S902;	9.61E-05	T508;S17;	4.56E-04	1.28E-01	2.11E-01
P82094	TMF1	S344;S72;	5.63E-05	S344;	9.39E-05	S344;S72;	1.65E-04	3.42E-01	5.71E-01
Q01814	ATP2B2	S1242;S1205;	1.77E-05	NA	NA	NA	NA	NA	NA
Q04721	NOTCH2	NA	NA	NA	NA	S1778;S2070;S2081;	2.46E-05	NA	NA
Q07960	ARHGAP1	NA	NA	S51;	2.32E-04	S51;	4.31E-04	NA	5.39E-01

Q12894	IFRD2	S93;S95;S96;	2.28E-05	S93;S95;S96;S87;S88;	1.41E-05	S93;S95;S96;	3.00E-05	7.58E-01	4.71E-01
Q13136	PPFIA1	S244;S238;S239;	6.85E-06	S239;S242;S708;S238;	2.48E-04	NA	NA	NA	NA
Q13765	NACA	S166;	4.44E-03	S166;	2.04E-03	S166;	2.43E-03	1.82E+00	8.37E-01
Q14687	GSE1	S826;S828;	3.44E-05	NA	NA	S1101;S826;T831;	5.47E-05	6.29E-01	NA
Q15056	EIF4H	S66;S21;	9.28E-06	NA	NA	NA	NA	NA	NA
Q15059	BRD3	S263;S563;	1.47E-04	S263;	1.11E-04	NA	NA	NA	NA
Q15276	RABEP1	S407;S410;S419;	2.62E-05	S407;S410;S374;S377;	2.06E-04	S407;S374;S377;S419;S410;	1.41E-04	1.86E-01	1.46E+00
Q2NKX8	ERCC6L	S946;S1069;	3.39E-05	NA	NA	NA	NA	NA	NA
Q53EP0	FNDC3B	NA	NA	S208;	4.55E-05	S208;	5.45E-05	NA	8.36E-01
Q53EZ4	CEP55	NA	NA	S425;S428;	4.99E-05	S428;S425;T430;	1.94E-05	NA	2.58E+00
Q5JRA6	MIA3	S1906;	4.48E-05	S1906;S1670;S1673;S1678;	1.27E-04	S1906;S1553;S1561;	7.61E-05	5.89E-01	1.67E+00
Q5T5U3	ARHGAP21	NA	NA	S1430;S1431;S1432;	1.85E-05	S1430;S1431;S1432;T1634;S923;S1636;S1638;	2.79E-05	NA	6.62E-01
Q5VSL9	STRIP1	S335;	2.76E-05	S335;	6.98E-05	S335;	1.14E-04	2.42E-01	6.12E-01
Q5VWQ8	DAB2IP	S35;T37;S33;	5.58E-04	NA	NA	NA	NA	NA	NA
Q5VYS8	ZCCHC6	NA	NA	T796;	2.64E-03	S777;T778;T796;	2.55E-03	NA	1.03E+00
Q66K74	MAP1S	T813;S759;	4.97E-05	S729;S731;S741;	7.70E-05	S582;S729;S731;T813;	4.76E-05	1.04E+00	1.62E+00
Q68DQ2	CRYBG3	S457;S1043;	3.85E-05	NA	NA	NA	NA	NA	NA
Q6NZ67	MZT2B	S152;	3.42E-05	S152;	3.99E-05	NA	NA	NA	NA
Q6VY07	PACS1	S531;T504;S493;	3.22E-05	S495;T526;T46;S528;S531;S529;	1.17E-04	S529;S534;T46;S497;S531;	1.18E-04	2.73E-01	9.90E-01
Q70J99	UNC13D	NA	NA	S150;	2.49E-05	NA	NA	NA	NA
Q86U44	METTL3	S43;	1.42E-05	S43;	3.04E-06	S43;S48;	3.33E-05	4.27E-01	9.12E-02
Q86V48	LUZP1	S659;	1.45E-05	S659;S946;T947;S949;	8.09E-06	S659;	1.39E-04	1.04E-01	5.83E-02
Q86W56	BPHL	S137;	2.73E-05	S268;S137;S261;S264;	1.56E-05	S316;S137;S264;S22;S197;S323;S261;	1.04E-04	2.63E-01	1.51E-01
Q86YS7	C2CD5	NA	NA	S260;S661;S662;S659;S295;	1.54E-04	S661;S662;S657;S659;T666;S260;S295;	9.02E-05	NA	1.71E+00
Q8IVP5	FUNDC1	NA	NA	Y11;	6.75E-05	Y11;S13;	4.17E-05	NA	1.62E+00
Q8IWZ8	SUGP1	S485;S411;	2.10E-05	S485;	1.67E-05	NA	NA	NA	NA
Q8IX90	SKA3	NA	NA	S155;	1.61E-05	NA	NA	NA	NA

Q8IYB7	DIS3L2	NA	NA	S875;	1.22E-04	S875;	5.09E-05	NA	2.39E+00
Q8IYW2	CFAP46	NA	NA	NA	NA	T2217;T2292;S2299;	7.70E-05	NA	NA
Q8NDV7	TNRC6A	NA	NA	NA	NA	S943;S739;	2.06E-05	NA	NA
Q8NEG2	C7orf57	NA	NA	S242;S245;S248;	8.94E-05	NA	NA	NA	NA
Q8NHV4	NEDD1	S516;	3.28E-05	S516;	3.85E-05	NA	NA	NA	NA
Q8WUI4	HDAC7	S486;S181;S405;	9.99E-05	S486;	2.83E-05	S486;S155;S181;	1.32E-04	7.58E-01	2.15E-01
Q8WUM4	PDCD6IP	NA	NA	NA	NA	S730;Y727;T738;	1.10E-04	NA	NA
Q8WXA9	SREK1	S251;S253;S255;S406;S408;S404;S361;T363;	5.38E-04	S404;S406;S408;S361;T363;S386;S390;S392;	1.22E-04	NA	NA	NA	NA
Q8WXG6	MADD	S818;S820;S1157;S1160;	9.84E-06	NA	NA	T1237;S1059;S818;S820;	6.76E-05	1.45E-01	NA
Q92541	RTF1	T55;S58;S60;	5.23E-05	T55;S58;S60;	8.38E-06	T55;S58;S60;	2.63E-05	1.99E+00	3.18E-01
Q96AY2	EME1	S84;S85;S87;S12;S15;	2.63E-05	S12;S15;S84;S85;S87;S17;S9;	6.82E-05	S84;S85;S87;S6;S7;S9;S12;S15;S17;	6.31E-05	4.17E-01	1.08E+00
Q96AY4	TTC28	NA	NA	S2104;S2224;	9.94E-05	NA	NA	NA	NA
Q96JC9	EAF1	S165;	1.41E-04	S165;T157;	5.14E-05	S165;	1.73E-04	8.15E-01	2.96E-01
Q96N21	ENTHD2	NA	NA	NA	NA	S356;	1.03E-05	NA	NA
Q96QU8	XPO6	NA	NA	S208;T204;T201;	3.07E-04	T204;S208;S224;T201;	5.81E-04	NA	5.28E-01
Q99081	TCF12	S558;S559;S67;	9.77E-05	NA	NA	S67;S559;S332;	4.93E-05	1.98E+00	NA
Q99638	RAD9A	NA	NA	S272;S277;S375;S380;S387;S341;T355;	7.29E-04	S277;S375;S387;S380;S270;	6.68E-05	NA	1.09E+01
Q9BQ52	ELAC2	NA	NA	NA	NA	S217;S212;S213;S215;S199;S208;	1.81E-04	NA	NA
Q9BQE3	TUBA1C	S439;T51;	1.34E-04	S439;S48;	1.70E-04	S48;S439;	5.20E-04	2.58E-01	3.26E-01
Q9BQI0	AIF1L	S134;	1.82E-05	NA	NA	NA	NA	NA	NA
Q9BVG9	PTDSS2	NA	NA	S16;	5.03E-05	S16;	2.05E-05	NA	2.45E+00
Q9BXJ9	NAA15	NA	NA	S856;S855;	1.50E-04	S856;S855;T850;	1.70E-04	NA	8.77E-01
Q9H1C4	UNC93B1	NA	NA	S547;S550;	4.12E-04	S547;S550;	2.92E-05	NA	1.41E+01
Q9H2G4	TSPYL2	NA	NA	S16;S20;	5.35E-05	S16;S17;	4.34E-05	NA	1.23E+00
Q9H2Y7	ZNF106	S1025;S1026;S861;S639;S641;	7.13E-05	S1370;S1025;S1026;S1279;	2.52E-05	S1025;S1026;S1279;S1328;S1370;	9.72E-05	7.34E-01	2.59E-01
Q9H8Y5	ANKZF1	S51;S56;	2.66E-05	NA	NA	S361;S56;T674;S51;S675;	2.66E-05	9.99E-01	NA
Q9H9L4	KANSL2	NA	NA	S147;S149;S168;S172;S1	4.12E-05	S168;S172;S175;S147;	8.95E-05	NA	4.60E-01

				75;		S149;			
Q9HAY2	MAGEF1	S95;	1.69E-05	S95;	8.44E-05	NA	NA	NA	NA
Q9NRL3	STRN4	NA	NA	S276;	2.52E-05	S276;	3.76E-05	NA	6.69E-01
Q9NRY4	ARHGAP35	NA	NA	S975;S985;S980;S1179;	3.48E-04	S975;S1179;S985;	6.80E-05	NA	5.12E+00
Q9NZJ0	DTL	S512;T516;	3.15E-05	S679;S512;T516;S485;	1.09E-05	S679;S511;T516;	1.73E-05	1.82E+00	6.28E-01
Q9P013	CWC15	NA	NA	NA	NA	T110;S121;	5.40E-05	NA	NA
Q9P265	DIP2B	S146;S148;S53;T144;	3.84E-05	NA	NA	NA	NA	NA	NA
Q9P2B4	CTTNBP2NL	NA	NA	S560;S563;S568;	9.76E-06	S560;S563;S568;S488;T570;S484;	4.75E-05	NA	2.06E-01
Q9P2W9	STX18	NA	NA	S189;S194;	4.15E-05	NA	NA	NA	NA
Q9UGH3	SLC23A2	NA	NA	NA	NA	S78;S81;	2.35E-05	NA	NA
Q9UJV9	DDX41	NA	NA	S21;S23;	3.93E-04	S23;Y33;S21;S66;S68;S83;	2.91E-04	NA	1.35E+00
Q9UKX7	NUP50	NA	NA	NA	NA	S223;	1.88E-04	NA	NA
Q9UKZ4	TENM1	Y1865;S1868;S1873;	9.33E-06	NA	NA	NA	NA	NA	NA
Q9ULG1	INO80	NA	NA	S47;S48;S51;S58;	7.70E-06	S47;S48;S51;	1.95E-05	NA	3.95E-01
Q9UNS1	TIMELESS	NA	NA	NA	NA	S1173;S1149;S1121;	9.63E-05	NA	NA
Q9UPU5	USP24	S1141;S2047;	4.22E-05	T2565;S2047;S1943;S2561;	3.07E-04	T2565;S2047;S1612;S2077;S2081;S1141;	3.79E-04	1.12E-01	8.10E-01
Q9Y2U8	LEMD3	S140;S141;S144;S261;	3.82E-05	S140;S141;S144;S259;S261;	1.54E-04	S259;S261;S141;S140;S144;	6.73E-05	5.67E-01	2.29E+00
Q9Y3F4	STRAP	NA	NA	NA	NA	S335;S338;	1.98E-05	NA	NA
Q9Y4C8	RBM19	S174;S176;S180;	1.11E-04	S174;S176;S180;	8.58E-05	S174;S176;S180;	5.51E-05	2.01E+00	1.56E+00
Q9Y4E8	USP15	S229;	2.67E-05	S229;	8.10E-05	S225;T226;S229;	3.42E-05	7.80E-01	2.37E+00
Q9Y5G4	PCDHGA9	S224;S223;	8.17E-05	NA	NA	NA	NA	NA	NA
Q9Y5U2	TSSC4	S319;S320;S321;	8.48E-06	NA	NA	NA	NA	NA	NA
Q9Y606	PUS1	T426;S420;	5.49E-05	T426;	3.31E-05	S415;S420;T426;	6.78E-05	8.10E-01	4.88E-01
Q9Y6E2	BZW2	S412;S414;	2.54E-04	S412;S414;	2.72E-03	S412;S414;	1.12E-03	2.27E-01	2.43E+00
A1L390	PLEKHG3	NA	NA	S1154;S1037;S1040;S1169;S433;S640;S643;	1.76E-04	S1154;S1037;S1040;S640;S643;S76;	7.41E-05	NA	2.38E+00
A1X283	SH3PXD2B	NA	NA	NA	NA	S291;	1.21E-05	NA	NA

A6NFI3	ZNF316	NA	NA	S112;	3.56E-05	NA	NA	NA	NA
O14640	DVL1	NA	NA	S581;S582;	3.05E-06	NA	NA	NA	NA
O14745	SLC9A3R1	NA	NA	S288;S280;S290;	1.06E-03	S280;S290;	3.75E-05	NA	2.82E+01
O43847	NRD1	NA	NA	S94;S96;S86;	5.01E-04	S94;S86;	1.93E-04	NA	2.60E+00
O60934	NBN	NA	NA	NA	NA	S432;	6.49E-05	NA	NA
O75410	TACC1	NA	NA	NA	NA	S55;S276;	2.19E-05	NA	NA
O76070	SNCG	NA	NA	NA	NA	S124;	8.53E-05	NA	NA
O94992	HEXIM1	NA	NA	NA	NA	S97;S98;	2.00E-05	NA	NA
O95671	ASMTL	NA	NA	NA	NA	S239;	7.24E-04	NA	NA
P07199	CENPB	NA	NA	NA	NA	S156;	1.70E-05	NA	NA
P08069	IGF1R	NA	NA	NA	NA	S1364;T354;	4.82E-06	NA	NA
P11274	BCR	NA	NA	S462;S122;S459;Y316;	1.37E-04	S459;S122;	6.64E-05	NA	2.06E+00
P15151	PVR	NA	NA	S407;S406;	7.85E-05	NA	NA	NA	NA
P15291	B4GALT1	NA	NA	NA	NA	S74;	2.82E-04	NA	NA
P16144	ITGB4	NA	NA	NA	NA	S1494;S1483;S1696;	8.90E-06	NA	NA
P23396	RPS3	NA	NA	T221;	1.06E-03	NA	NA	NA	NA
P31947	SFN	NA	NA	NA	NA	S248;	3.09E-05	NA	NA
P33527	ABCC1	NA	NA	NA	NA	S919;S916;S915;S918;S930;	9.25E-05	NA	NA
P40818	USP8	NA	NA	NA	NA	S389;S718;	1.90E-04	NA	NA
P41229	KDM5C	NA	NA	NA	NA	S1359;S301;	7.52E-05	NA	NA
P49354	FNTA	NA	NA	NA	NA	S373;	4.89E-05	NA	NA
P49848	TAF6	NA	NA	S653;T660;S634;	1.22E-05	S653;T622;	2.71E-05	NA	4.51E-01
P49916	LIG3	NA	NA	NA	NA	S242;T244;	2.91E-05	NA	NA
P51991	HNRNPA3	NA	NA	S366;S358;	3.87E-04	S366;Y364;S358;	1.81E-04	NA	2.14E+00
P58397	ADAMTS12	NA	NA	T428;T431;S433;	1.78E-04	T428;T431;S433;	7.50E-05	NA	2.37E+00
P61247	RPS3A	NA	NA	NA	NA	S263;	1.03E-04	NA	NA
P62750	RPL23A	NA	NA	T42;	6.62E-05	T42;S43;	1.86E-04	NA	3.56E-01
P78316	NOP14	NA	NA	NA	NA	T161;S146;S148;	4.16E-05	NA	NA
Q06413	MEF2C	NA	NA	NA	NA	S461;	2.42E-03	NA	NA
Q0VF96	CGNL1	NA	NA	S196;S204;S283;	2.35E-05	NA	NA	NA	NA

Q12846	STX4	NA	NA	S14;S15;S117;	1.73E-05	S14;S15;	7.98E-05	NA	2.17E-01
Q13017	ARHGAP5	NA	NA	NA	NA	S1173;S1176;	4.02E-05	NA	NA
Q13131	PRKAA1	NA	NA	S508;S496;	3.53E-05	S496;	5.79E-05	NA	6.10E-01
Q13409	DYNC1I2	NA	NA	S92;S97;S101;	1.16E-05	T95;S92;S94;	3.23E-05	NA	3.58E-01
Q13426	XRCC4	NA	NA	NA	NA	S327;S328;	4.64E-06	NA	NA
Q14524	SCN5A	NA	NA	NA	NA	S524;S525;	2.39E-03	NA	NA
Q14562	DHX8	NA	NA	NA	NA	S226;S228;S230;	4.89E-05	NA	NA
Q15418	RPS6KA1	NA	NA	NA	NA	S363;T359;	1.55E-05	NA	NA
Q15742	NAB2	NA	NA	NA	NA	S6;S162;S171;S159;	1.88E-04	NA	NA
Q3B726	TWISTNB	NA	NA	S328;	3.03E-05	S316;S328;S304;	1.60E-04	NA	1.89E-01
Q5PRF9	SAMD4B	NA	NA	NA	NA	S271;	5.83E-05	NA	NA
Q5TDH0	DDI2	NA	NA	S106;	3.29E-05	S121;S106;S194;	3.14E-05	NA	1.05E+00
Q5TGY3	AHDC1	NA	NA	NA	NA	S1187;S1399;T1401;	1.18E-05	NA	NA
Q684P5	RAP1GAP2	NA	NA	NA	NA	S609;S612;S613;	2.65E-05	NA	NA
Q69YQ0	SPECC1L	NA	NA	S384;S887;S832;S385;S220;	5.28E-04	S384;S832;	3.34E-05	NA	1.58E+01
Q6IBW4	NCAPH2	NA	NA	S284;	2.76E-05	S284;	4.47E-05	NA	6.19E-01
Q6P1N0	CC2D1A	NA	NA	NA	NA	S455;	7.42E-06	NA	NA
Q6ZUT1	C11orf57	NA	NA	S285;S288;S289;	1.64E-05	NA	NA	NA	NA
Q7Z4H7	HAUS6	NA	NA	T584;	1.07E-05	T584;	1.57E-05	NA	6.81E-01
Q86UP2	KTN1	NA	NA	S75;S77;	1.32E-05	S75;	7.33E-05	NA	1.80E-01
Q8IWZ3	ANKHD1-EIF4EBP3	NA	NA	S95;S93;T86;S1679;	1.09E-05	NA	NA	NA	NA
Q8IXQ4	GPALPP1	NA	NA	NA	NA	S105;	5.56E-05	NA	NA
Q8N1G4	LRRC47	NA	NA	S520;S518;	2.18E-04	S520;S518;	1.70E-04	NA	1.29E+00
Q8N3D4	EHBP1L1	NA	NA	NA	NA	S310;S1257;T883;S884;T891;S1016;S1017;S1264;	1.69E-04	NA	NA
Q8NAF0	ZNF579	NA	NA	NA	NA	S483;T482;	7.48E-06	NA	NA
Q8NHM5	KDM2B	NA	NA	NA	NA	S445;T447;S1031;	5.12E-05	NA	NA
Q8TC07	TBC1D15	NA	NA	S70;S675;	6.30E-05	S675;	5.72E-05	NA	1.10E+00
Q8TEK3	DOT1L	NA	NA	NA	NA	S1001;S1009;S826;S8	5.51E-06	NA	NA

						34;			
Q8WUF5	PPP1R13L	NA	NA	NA	NA	Y109;S567;	1.66E-05	NA	NA
Q8WUM0	NUP133	NA	NA	NA	NA	S45;S50;S57;	5.86E-05	NA	NA
Q92466	DDB2	NA	NA	NA	NA	S26;S24;	7.49E-05	NA	NA
Q92522	H1FX	NA	NA	S31;	1.96E-04	S31;	6.76E-04	NA	2.89E-01
Q92685	ALG3	NA	NA	S11;S13;	2.63E-04	S13;	8.66E-05	NA	3.04E+00
Q96B97	SH3KBP1	NA	NA	S230;S183;	1.12E-05	S230;S183;	1.20E-05	NA	9.34E-01
Q96C90	PPP1R14B	NA	NA	NA	NA	S32;	8.45E-05	NA	NA
Q96C92	SDCCAG3	NA	NA	NA	NA	S243;S247;	1.34E-04	NA	NA
Q96D71	REPS1	NA	NA	S162;S170;T173;S709;	7.68E-05	S120;S390;S428;S429;T539;S540;S518;T520;S272;S274;	7.97E-05	NA	9.63E-01
Q96E39	RBMXL1	NA	NA	NA	NA	S58;S208;Y246;	3.18E-04	NA	NA
Q96EU6	RRP36	NA	NA	S73;	2.83E-05	NA	NA	NA	NA
Q96HC4	PDLIM5	NA	NA	S111;S228;	1.44E-04	S313;S228;	3.12E-05	NA	4.63E+00
Q96JY6	PDLIM2	NA	NA	S134;S137;T126;S129;Y305;T312;	3.06E-05	NA	NA	NA	NA
Q96L73	NSD1	NA	NA	NA	NA	S2471;	9.47E-06	NA	NA
Q96MY1	NOL4L	NA	NA	S295;S130;	1.42E-04	NA	NA	NA	NA
Q96N64	PWWP2A	NA	NA	NA	NA	S521;S81;	5.02E-05	NA	NA
Q99569	PKP4	NA	NA	S314;Y275;S281;S227;S233;	5.45E-05	S337;S336;S293;	1.17E-05	NA	4.66E+00
Q99624	SLC38A3	NA	NA	S54;T466;	2.25E-05	NA	NA	NA	NA
Q9BQA1	WDR77	NA	NA	NA	NA	T5;	5.71E-05	NA	NA
Q9BRS2	RIOK1	NA	NA	S22;S21;S24;	4.75E-05	S21;S22;S24;	1.23E-05	NA	3.86E+00
Q9BZZ5	API5	NA	NA	S462;S464;S470;	8.36E-04	S462;S464;	1.25E-03	NA	6.70E-01
Q9H2K8	TAOK3	NA	NA	S324;	3.85E-05	NA	NA	NA	NA
Q9H3P2	NELFA	NA	NA	S363;	1.04E-04	S363;T227;S233;	9.31E-05	NA	1.12E+00
Q9H4L4	SENP3	NA	NA	S232;S169;T229;	6.89E-05	S169;S232;	1.70E-04	NA	4.05E-01
Q9H7D0	DOCK5	NA	NA	NA	NA	S1775;S1789;S1834;S1805;	3.45E-05	NA	NA
Q9NP66	HMG20A	NA	NA	NA	NA	S105;	3.70E-05	NA	NA

Q9NWA0	MED9	NA	NA	NA	NA	S53;	2.30E-05	NA	NA
Q9NWM3	CUEDC1	NA	NA	S8;S10;S9;	7.23E-07	NA	NA	NA	NA
Q9NXH9	TRMT1	NA	NA	NA	NA	T646;S625;	5.56E-05	NA	NA
Q9NY27	PPP4R2	NA	NA	S226;	1.67E-04	S226;S364;	2.28E-04	NA	7.33E-01
Q9NYW8	RBAK	NA	NA	S386;	1.99E-05	NA	NA	NA	NA
Q9NZM3	ITSN2	NA	NA	S889;	1.20E-04	S884;S889;	6.25E-05	NA	1.92E+00
Q9P035	HACD3	NA	NA	S114;	1.11E-05	S114;	9.97E-06	NA	1.12E+00
Q9ULD2	MTUS1	NA	NA	S541;S1268;S399;S1245;S1264;S1255;	1.31E-04	NA	NA	NA	NA
Q9ULH0	KIDINS220	NA	NA	T1679;S1526;	2.14E-05	S1526;S1555;T1679;S1521;	5.80E-05	NA	3.69E-01
Q9ULJ3	ZBTB21	NA	NA	S972;T979;S1003;S981;S983;	1.70E-05	S1003;S983;T979;S415;T431;S438;	3.80E-05	NA	4.48E-01
Q9Y237	PIN4	NA	NA	S19;	1.44E-06	NA	NA	NA	NA
Q9Y253	POLH	NA	NA	NA	NA	S379;S380;	6.38E-03	NA	NA
Q9Y2K6	USP20	NA	NA	S132;S134;	6.10E-05	S132;S134;S373;T377;	5.88E-05	NA	1.04E+00
Q9Y314	NOSIP	NA	NA	S107;	5.18E-05	S135;S107;	2.46E-05	NA	2.11E+00
Q9Y519	TMEM184B	NA	NA	NA	NA	S402;S403;	2.01E-05	NA	NA
Q9Y608	LRRFIP2	NA	NA	S324;S333;	1.05E-04	S328;T331;S324;	1.29E-04	NA	8.09E-01
Q9Y6J9	TAF6L	NA	NA	T604;S605;	2.59E-05	S481;T604;S605;	2.76E-05	NA	9.39E-01
A1L170	C1orf226	NA	NA	S222;S223;S244;S249;S225;	1.28E-04	NA	NA	NA	NA
A2AJT9	CXorf23	NA	NA	S312;	2.41E-04	NA	NA	NA	NA
A5YM69	ARHGEF35	NA	NA	S445;S450;	7.92E-05	NA	NA	NA	NA
A6ND36	FAM83G	NA	NA	S634;S356;S650;S760;	1.03E-04	S634;T658;S760;	5.03E-06	NA	2.04E+01
B1AK53	ESPN	NA	NA	S696;S700;S612;	5.95E-06	NA	NA	NA	NA
B2RUZ4	SMIM1	NA	NA	S22;S27;	1.19E-04	NA	NA	NA	NA
B3KS81	SRRM5	NA	NA	T90;	1.11E-06	NA	NA	NA	NA
C9JLW8	FAM195B	NA	NA	S17;S18;S21;S25;S26;	1.14E-04	NA	NA	NA	NA
O00425	IGF2BP3	NA	NA	T528;	5.61E-06	NA	NA	NA	NA
O00443	PIK3C2A	NA	NA	S60;S259;S1645;S1648;T1662;	4.66E-05	S338;S259;	3.41E-04	NA	1.37E-01

O00515	LAD1	NA	NA	S121;S123;S64;S485;S272;T41;T305;S420;S355;S177;S301;T304;S394;T40;	3.79E-03	NA	NA	NA	NA
O00559	EBAG9	NA	NA	S36;	9.31E-05	S36;	6.56E-05	NA	1.42E+00
O00562	PITPNM1	NA	NA	S373;	2.18E-05	S373;	1.65E-05	NA	1.33E+00
O00629	KPNA4	NA	NA	S60;	1.54E-04	S60;	1.84E-04	NA	8.39E-01
O14715	RGPD8	NA	NA	NA	NA	S1274;S21;T1482;S1486;S1478;T1474;S1265;S1275;S1481;	2.83E-04	NA	NA
O14828	SCAMP3	NA	NA	S32;S76;	2.45E-04	S32;S76;	1.10E-04	NA	2.24E+00
O15127	SCAMP2	NA	NA	S319;	2.13E-04	S320;	1.23E-04	NA	1.74E+00
O15371	EIF3D	NA	NA	S528;S529;	3.29E-04	S528;S529;	1.69E-04	NA	1.95E+00
O43379	WDR62	NA	NA	S1226;S1249;T1268;S33;	4.45E-05	S1226;T1053;	2.40E-05	NA	1.85E+00
O43524	FOXO3	NA	NA	S425;S438;	1.35E-05	NA	NA	NA	NA
O43639	NCK2	NA	NA	S90;	2.47E-05	NA	NA	NA	NA
O43768	ENSA	NA	NA	S109;	1.22E-03	S109;	1.05E-04	NA	1.16E+01
O60307	MAST3	NA	NA	S1223;S709;S710;Y708;	1.64E-05	Y708;S1223;S153;S155;S157;	1.29E-05	NA	1.27E+00
O60318	MCM3AP	NA	NA	S527;S538;	4.71E-05	T530;S538;	1.17E-05	NA	4.04E+00
O60343	TBC1D4	NA	NA	S754;S588;S591;S341;	9.86E-05	S341;S754;S588;S591;	1.48E-04	NA	6.67E-01
O60469	DSCAM	NA	NA	Y482;T485;Y495;	4.32E-05	NA	NA	NA	NA
O60524	NEMF	NA	NA	S747;S748;	3.50E-05	S747;S748;S417;	8.56E-05	NA	4.09E-01
O60825	PFKFB2	NA	NA	S466;S483;S493;	1.68E-04	S466;	1.06E-04	NA	1.58E+00
O75122	CLASP2	NA	NA	S596;	2.20E-05	S596;S370;S499;S503;S507;S533;S536;S541;S529;S327;S523;	3.49E-04	NA	6.32E-02
O75190	DNAJB6	NA	NA	S277;	1.36E-05	NA	NA	NA	NA
O75319	DUSP11	NA	NA	S18;	1.00E-05	NA	NA	NA	NA
O75379	VAMP4	NA	NA	S30;	4.65E-05	S30;	6.15E-05	NA	7.56E-01
O75385	ULK1	NA	NA	NA	NA	S638;S623;T625;	2.24E-05	NA	NA
O75396	SEC22B	NA	NA	S137;S174;	1.93E-04	S137;	1.34E-05	NA	1.44E+01
O75448	MED24	NA	NA	S862;S873;	1.76E-05	S862;S873;T916;	8.72E-05	NA	2.02E-01
O75694	NUP155	NA	NA	S992;	1.41E-04	S992;	2.89E-04	NA	4.89E-01
O75925	PIAS1	NA	NA	S503;S466;S467;S468;	8.12E-06	NA	NA	NA	NA

O75976	CPD	NA	NA	T1368;T1370;T1374;	1.68E-05	NA	NA	NA	NA
O94885	SASH1	NA	NA	S699;S701;S704;S706;S407;	3.27E-05	NA	NA	NA	NA
O94887	FARP2	NA	NA	S399;S410;S402;S344;	1.72E-05	NA	NA	NA	NA
O94888	UBXN7	NA	NA	NA	NA	S285;S288;S350;S278;S280;	6.96E-06	NA	NA
O95049	TJP3	NA	NA	S905;S906;S112;S346;	1.24E-04	NA	NA	NA	NA
O95297	MPZL1	NA	NA	S210;	1.47E-04	S210;S219;	1.70E-04	NA	8.63E-01
O95490	ADGRL2	NA	NA	S1116;S1112;S1430;	7.09E-05	NA	NA	NA	NA
O95674	CDS2	NA	NA	S21;S23;S35;S33;S37;	1.43E-04	NA	NA	NA	NA
O95714	HERC2	NA	NA	NA	NA	S1938;S1948;T1944;	1.95E-05	NA	NA
O95772	STARD3NL	NA	NA	S214;S218;S210;	5.79E-05	S214;S218;S210;	2.32E-05	NA	2.49E+00
O95819	MAP4K4	NA	NA	S900;S629;S631;S710;S715;	1.26E-04	S710;S631;S639;	5.23E-05	NA	2.40E+00
O95831	AIFM1	NA	NA	S268;	1.03E-05	NA	NA	NA	NA
O95835	LATS1	NA	NA	NA	NA	S464;S613;	2.80E-05	NA	NA
O96013	PAK4	NA	NA	S181;S291;	2.29E-04	S181;	7.15E-05	NA	3.20E+00
P00558	PGK1	NA	NA	S203;	2.82E-05	S203;	3.04E-05	NA	9.25E-01
P01106	MYC	NA	NA	NA	NA	T58;S62;T343;S344;	1.82E-05	NA	NA
P01891	NA	NA	NA	NA	NA	NA	NA	NA	NA
P04406	GAPDH	NA	NA	S83;	2.61E-05	NA	NA	NA	NA
P04920	SLC4A2	NA	NA	S144;S173;	6.53E-05	S144;	1.18E-05	NA	5.54E+00
P05412	JUN	NA	NA	NA	NA	S63;S73;	1.04E-04	NA	NA
P09327	VIL1	NA	NA	S747;	3.71E-05	NA	NA	NA	NA
P10696	ALPPL2	NA	NA	NA	NA	T429;	1.14E-05	NA	NA
P11137	MAP2	NA	NA	T1780;S1790;S1782;T1649;S1653;S1787;	6.68E-05	NA	NA	NA	NA
P11474	ESRRA	NA	NA	S19;S22;	4.55E-05	NA	NA	NA	NA
P13639	EEF2	NA	NA	T57;T59;	5.49E-04	T57;	2.28E-04	NA	2.41E+00
P13798	APEH	NA	NA	S187;	9.76E-06	S187;	2.73E-05	NA	3.58E-01
P16401	HIST1H1B	NA	NA	T138;S173;S189;	3.05E-04	NA	NA	NA	NA
P17252	PRKCA	NA	NA	T497;S319;	3.36E-05	T497;S319;S226;	1.78E-05	NA	1.88E+00

P17612	PRKACA	NA	NA	T198;T196;	2.21E-05	T196;T198;	4.86E-06	NA	4.56E+00
P17987	TCP1	NA	NA	S544;	1.35E-05	NA	NA	NA	NA
P18206	VCL	NA	NA	NA	NA	S721;S290;	4.23E-05	NA	NA
P19838	NFKB1	NA	NA	S907;T897;T898;	2.54E-05	S907;S223;S226;T228;S903;	5.64E-05	NA	4.50E-01
P20823	HNF1A	NA	NA	S247;T74;T67;T82;S70;	3.70E-04	NA	NA	NA	NA
P21359	NF1	NA	NA	NA	NA	S2515;S2817;	5.42E-05	NA	NA
P22607	FGFR3	NA	NA	S444;T450;S445;	5.86E-06	NA	NA	NA	NA
P23443	RPS6KB1	NA	NA	S441;T444;S447;T399;	2.41E-05	NA	NA	NA	NA
P23508	MCC	NA	NA	S294;	4.94E-05	NA	NA	NA	NA
P25098	ADRBK1	NA	NA	S670;	1.47E-04	S670;	1.13E-05	NA	1.30E+01
P27348	YWHAQ	NA	NA	T229;S232;	1.08E-04	S232;	2.39E-04	NA	4.52E-01
P29375	KDM5A	NA	NA	S1331;	7.12E-06	NA	NA	NA	NA
P30260	CDC27	NA	NA	S364;T368;	4.46E-05	T366;	4.92E-05	NA	9.06E-01
P30419	NMT1	NA	NA	S47;	1.00E-03	S47;S69;	8.66E-04	NA	1.16E+00
P31321	PRKAR1B	NA	NA	S83;S71;S77;	4.55E-04	S83;S71;	3.67E-05	NA	1.24E+01
P31629	HIVEP2	NA	NA	S2130;S37;	5.75E-05	S2130;	3.32E-05	NA	1.73E+00
P35568	IRS1	NA	NA	S1101;	3.09E-05	NA	NA	NA	NA
P36871	PGM1	NA	NA	S117;	1.60E-05	S117;	3.98E-04	NA	4.03E-02
P38432	COIL	NA	NA	S566;T122;	1.04E-04	NA	NA	NA	NA
P40425	PBX2	NA	NA	T325;S330;	3.92E-05	S151;S146;	1.91E-05	NA	2.05E+00
P41235	HNF4A	NA	NA	S436;S167;	1.82E-04	NA	NA	NA	NA
P41440	SLC19A1	NA	NA	S499;S503;	6.19E-05	S499;S507;S515;S503;	3.44E-05	NA	1.80E+00
P42566	EPS15	NA	NA	NA	NA	S562;S796;S814;S323;S681;T683;S684;	6.94E-05	NA	NA
P42685	FRK	NA	NA	S37;	5.54E-05	NA	NA	NA	NA
P42858	HTT	NA	NA	S419;S432;S1195;S620;S621;S634;	2.70E-04	S419;S1199;S432;	6.63E-05	NA	4.07E+00
P46783	RPS10	NA	NA	S146;	3.37E-05	S146;	2.62E-05	NA	1.29E+00
P46934	NEDD4	NA	NA	S670;S675;	1.80E-04	S670;	6.93E-05	NA	2.59E+00
P47736	RAP1GAP	NA	NA	S499;	4.21E-06	NA	NA	NA	NA
P49327	FASN	NA	NA	T2204;	1.13E-04	T2204;	3.06E-05	NA	3.69E+00
P49768	PSEN1	NA	NA	NA	NA	S366;S367;	4.64E-05	NA	NA

P49815	TSC2	NA	NA	NA	NA	S1371;S1385;S1388;S1411;T659;S664;T667;S1364;	7.25E-05	NA	NA
P50548	ERF	NA	NA	S185;S187;S190;S21;	3.06E-05	NA	NA	NA	NA
P50613	CDK7	NA	NA	S164;	5.23E-05	S164;T170;	1.07E-04	NA	4.88E-01
P51956	NEK3	NA	NA	T479;Y273;S355;	3.53E-05	T479;	2.00E-05	NA	1.77E+00
P52655	GTF2A1	NA	NA	S316;S321;	2.90E-05	S316;S321;	5.49E-05	NA	5.28E-01
P52926	HMGA2	NA	NA	S101;S102;S105;S44;	1.35E-03	S102;S105;T100;S44;	1.19E-04	NA	1.14E+01
P53602	MVD	NA	NA	S96;	8.33E-05	NA	NA	NA	NA
P53671	LIMK2	NA	NA	S297;S287;S293;S298;	2.26E-05	NA	NA	NA	NA
P53814	SMTN	NA	NA	T517;S514;S792;	3.54E-05	NA	NA	NA	NA
P53985	SLC16A1	NA	NA	T466;S467;S461;	2.57E-04	S461;S467;T466;	5.02E-05	NA	5.12E+00
P55317	FOXA1	NA	NA	S331;S307;	1.98E-04	S331;	3.01E-05	NA	6.58E+00
P56211	ARPP19	NA	NA	S104;S108;	2.16E-05	S23;	2.72E-05	NA	7.94E-01
P57740	NUP107	NA	NA	NA	NA	S11;S86;	2.96E-05	NA	NA
P60174	TPI1	NA	NA	S58;S36;S41;	4.35E-05	S58;	5.05E-05	NA	8.60E-01
P62888	RPL30	NA	NA	S10;	8.53E-06	NA	NA	NA	NA
P78312	FAM193A	NA	NA	S293;	1.73E-05	S293;T290;	1.89E-05	NA	9.14E-01
P78356	PIP4K2B	NA	NA	T322;S326;	4.04E-05	T322;S326;	3.44E-05	NA	1.17E+00
P78371	CCT2	NA	NA	S470;S60;T64;T69;T27;S41;T42;	1.29E-05	NA	NA	NA	NA
P78536	ADAM17	NA	NA	S791;	3.40E-05	NA	NA	NA	NA
P98171	ARHGAP4	NA	NA	S930;	1.86E-04	NA	NA	NA	NA
Q00587	CDC42EP1	NA	NA	S19;S192;S350;S353;S65;S195;S121;	1.08E-04	S350;S353;S192;S142;S19;T145;	1.54E-04	NA	7.04E-01
Q01581	HMGCS1	NA	NA	S495;S486;	1.03E-04	S495;	9.44E-05	NA	1.09E+00
Q01850	CDR2	NA	NA	NA	NA	S309;	1.84E-05	NA	NA
Q02487	DSC2	NA	NA	S864;S868;S873;T512;T515;T519;	3.85E-05	NA	NA	NA	NA
Q02952	AKAP12	NA	NA	S627;S629;S1331;S598;S696;S697;S698;	2.24E-05	S598;S627;S286;T597;S629;S696;S697;S698;	1.18E-04	NA	1.91E-01
Q03154	ACY1	NA	NA	T201;S208;	2.94E-05	NA	NA	NA	NA
Q03188	CENPC	NA	NA	S709;S710;	1.73E-05	S709;S710;S73;	9.66E-06	NA	1.79E+00

Q04724	TLE1	NA	NA	S286;	8.15E-05	NA	NA	NA	NA
Q06210	GFPT1	NA	NA	S261;	1.30E-03	S261;	9.36E-04	NA	1.39E+00
Q07617	SPAG1	NA	NA	S423;	8.07E-05	NA	NA	NA	NA
Q08495	DMTN	NA	NA	S96;S105;S333;S289;	7.28E-05	NA	NA	NA	NA
Q08752	PPID	NA	NA	NA	NA	S201;S198;	9.73E-06	NA	NA
Q12769	NUP160	NA	NA	S1157;	2.28E-05	S1157;	8.85E-05	NA	2.57E-01
Q12774	ARHGEF5	NA	NA	S445;S450;S1126;S184;	1.30E-04	S445;S450;S1126;	3.01E-05	NA	4.31E+00
Q12792	TWF1	NA	NA	S143;	3.81E-05	S143;	5.20E-05	NA	7.31E-01
Q12830	BPTF	NA	NA	S763;	1.30E-05	NA	NA	NA	NA
Q12929	EPS8	NA	NA	S660;S659;S811;S815;S661;	1.24E-04	S659;S662;S664;	1.04E-05	NA	1.19E+01
Q12955	ANK3	NA	NA	S4298;S1569;S4342;	6.47E-05	S1569;	9.95E-05	NA	6.51E-01
Q13153	PAK1	NA	NA	S174;S223;T229;T225;	1.22E-04	S174;T230;	9.32E-05	NA	1.31E+00
Q13330	MTA1	NA	NA	T578;	7.39E-05	NA	NA	NA	NA
Q13424	SNTA1	NA	NA	S189;S193;	1.02E-05	S189;S193;	6.55E-06	NA	1.56E+00
Q13464	ROCK1	NA	NA	T1101;S1102;S1105;	1.05E-04	S1105;S1108;S1102;	4.89E-05	NA	2.16E+00
Q13469	NFATC2	NA	NA	S856;S236;S243;S330;T325;S326;	1.12E-04	NA	NA	NA	NA
Q13557	CAMK2D	NA	NA	T337;	5.51E-05	T337;	1.41E-05	NA	3.91E+00
Q13619	CUL4A	NA	NA	S10;	1.44E-04	NA	NA	NA	NA
Q13625	TP53BP2	NA	NA	S737;S556;	2.67E-05	S556;S480;S698;	1.19E-05	NA	2.25E+00
Q13796	SHROOM2	NA	NA	S1036;S1039;S313;	9.22E-05	NA	NA	NA	NA
Q13813	SPTAN1	NA	NA	S1217;	4.69E-05	S1217;	1.33E-05	NA	3.52E+00
Q13884	SNTB1	NA	NA	S219;T214;S87;S389;	9.03E-05	NA	NA	NA	NA
Q14126	DSG2	NA	NA	S680;S703;	1.93E-05	S680;S703;S782;	1.13E-04	NA	1.71E-01
Q14147	DHX34	NA	NA	S749;S750;	8.97E-05	S749;S750;	3.69E-05	NA	2.43E+00
Q14181	POLA2	NA	NA	S141;S147;S154;S150;	1.27E-05	S141;S152;S147;	7.37E-05	NA	1.72E-01
Q14315	FLNC	NA	NA	S2233;S2602;	3.24E-04	S2233;S2624;Y2625;S2626;T2238;T2606;	3.34E-04	NA	9.69E-01
Q14542	SLC29A2	NA	NA	S252;	2.99E-04	NA	NA	NA	NA
Q14573	ITPR3	NA	NA	S934;S2670;S937;S1832;	6.05E-05	NA	NA	NA	NA
Q14653	IRF3	NA	NA	S175;	1.05E-05	S175;	2.76E-05	NA	3.80E-01
Q14738	PPP2R5D	NA	NA	S573;S88;S89;	1.88E-04	NA	NA	NA	NA

Q15014	MORF4L2	NA	NA	S71;S8;	1.61E-05	NA	NA	NA	NA
Q15021	NCAPD2	NA	NA	S1333;	1.35E-05	S1333;	1.11E-04	NA	1.22E-01
Q15025	TNIP1	NA	NA	S434;S435;T438;	1.71E-05	S435;S442;S434;T431;	1.98E-05	NA	8.60E-01
Q15361	TTF1	NA	NA	Y476;S481;	2.11E-05	S481;S487;	2.54E-05	NA	8.32E-01
Q15413	RYR3	NA	NA	S3391;S3395;	4.22E-06	NA	NA	NA	NA
Q15527	SURF2	NA	NA	NA	NA	T190;T195;	4.10E-05	NA	NA
Q15629	TRAM1	NA	NA	S365;	3.95E-05	NA	NA	NA	NA
Q15751	HERC1	NA	NA	NA	NA	T1429;S1514;S1521;T227;S237;S4857;	1.78E-05	NA	NA
Q15910	EZH2	NA	NA	T487;T367;S375;	1.90E-05	T487;T367;S84;	5.11E-05	NA	3.72E-01
Q16512	PKN1	NA	NA	S916;	2.44E-05	S916;S562;	3.31E-05	NA	7.38E-01
Q16625	OCLN	NA	NA	S358;T404;S408;	5.46E-05	NA	NA	NA	NA
Q2KHT3	CLEC16A	NA	NA	S863;S864;	3.14E-05	NA	NA	NA	NA
Q2TAC6	KIF19	NA	NA	T549;	2.71E-05	NA	NA	NA	NA
Q2YD98	UVSSA	NA	NA	S281;S287;	3.82E-05	NA	NA	NA	NA
Q3ZCW2	LGALSL	NA	NA	S25;	1.06E-05	NA	NA	NA	NA
Q4KMQ1	TPRN	NA	NA	S362;S418;S241;	1.59E-04	S418;S362;	3.81E-05	NA	4.17E+00
Q4L180	FILIP1L	NA	NA	S791;	1.91E-05	NA	NA	NA	NA
Q5HYI7	MTX3	NA	NA	S311;	3.36E-05	NA	NA	NA	NA
Q5JVS0	HABP4	NA	NA	S108;	9.12E-05	NA	NA	NA	NA
Q5MIZ7	PPP4R3B	NA	NA	S840;	7.90E-06	S840;	1.74E-05	NA	4.55E-01
Q5NUL3	FFAR4	NA	NA	NA	NA	T363;T365;S366;	8.23E-06	NA	NA
Q5SRE5	NUP188	NA	NA	S1709;S1717;	3.68E-05	NA	NA	NA	NA
Q5T0N5	FNBP1L	NA	NA	S489;S501;S295;T496;	5.75E-05	S501;S295;S505;T496;	1.46E-04	NA	3.93E-01
Q5T3F8	TMEM63B	NA	NA	S114;S115;S113;	8.49E-06	NA	NA	NA	NA
Q5T5C0	STXBP5	NA	NA	NA	NA	T762;S759;	4.19E-06	NA	NA
Q5T5P2	KIAA1217	NA	NA	S1044;	1.50E-05	NA	NA	NA	NA
Q5TCQ9	MAGI3	NA	NA	S1280;S1284;	2.11E-05	NA	NA	NA	NA
Q5VV41	ARHGEF16	NA	NA	T226;S227;S230;S174;S208;S576;	1.30E-04	NA	NA	NA	NA
Q5VVP1	SPATA31	NA	NA	NA	NA	S556;	7.50E-05	NA	NA

	A6								
Q658Y4	FAM91A1	NA	NA	NA	NA	S355;S671;	1.58E-05	NA	NA
Q66K14	TBC1D9B	NA	NA	NA	NA	S435;S411;S1241;	1.12E-04	NA	NA
Q676U5	ATG16L1	NA	NA	S287;	6.87E-05	NA	NA	NA	NA
Q6A1A2	NA	NA	NA	NA	NA	NA	NA	NA	NA
Q6BDS2	UHRF1BP1	NA	NA	T1101;S1103;S1106;	7.91E-05	S1106;T1101;	1.52E-05	NA	5.22E+00
Q6EEV4	POLR2M	NA	NA	S10;	5.96E-06	NA	NA	NA	NA
Q6GQQ9	OTUD7B	NA	NA	S464;S467;S100;S449;	4.42E-05	S464;S467;S100;S449;	4.72E-05	NA	9.36E-01
Q6GYQ0	RALGAPA1	NA	NA	NA	NA	S797;S864;S860;	6.80E-05	NA	NA
Q6IN85	PPP4R3A	NA	NA	S774;S777;S780;	1.55E-05	S774;S777;S771;S780;S741;	2.93E-05	NA	5.30E-01
Q6NXT4	SLC30A6	NA	NA	NA	NA	S382;	1.37E-04	NA	NA
Q6P4R8	NFRKB	NA	NA	S351;S347;	2.29E-05	S1291;S351;	4.23E-05	NA	5.42E-01
Q6PJF5	RHBDF2	NA	NA	S323;S328;S385;S90;	4.79E-05	S90;T175;S385;S323;S328;	6.43E-05	NA	7.46E-01
Q6ULP2	AFTPH	NA	NA	T617;S382;S518;	2.56E-05	NA	NA	NA	NA
Q6XQN6	NAPRT	NA	NA	S537;	3.29E-05	NA	NA	NA	NA
Q6ZRP7	QSOX2	NA	NA	S25;S578;	1.72E-05	S578;S25;	3.61E-04	NA	4.75E-02
Q6ZU35	KIAA1211	NA	NA	S874;	2.13E-05	S874;	2.36E-05	NA	9.02E-01
Q6ZUM4	ARHGAP27	NA	NA	S633;S466;	1.01E-05	NA	NA	NA	NA
Q70Z53	FRA10AC1	NA	NA	S278;S283;S285;	2.11E-05	NA	NA	NA	NA
Q7RTP6	MICAL3	NA	NA	S685;S977;S1173;S1143;	4.21E-05	NA	NA	NA	NA
Q7Z333	SETX	NA	NA	S1017;S1019;S687;S692;S693;	2.06E-05	NA	NA	NA	NA
Q7Z3T8	ZFYVE16	NA	NA	NA	NA	S939;S946;	1.98E-05	NA	NA
Q7Z406	MYH14	NA	NA	S1969;S1983;S1989;	2.72E-04	NA	NA	NA	NA
Q7Z478	DHX29	NA	NA	S71;S200;	1.54E-05	NA	NA	NA	NA
Q86VR2	FAM134C	NA	NA	T440;T310;S313;S320;S436;	7.36E-05	S436;S320;S360;S433;S435;T440;	3.14E-05	NA	2.35E+00

Q86WC4	OSTM1	NA	NA	S325;S322;S323;	6.78E-05	S325;S322;	6.77E-05	NA	1.00E+00
Q86X29	LSR	NA	NA	S493;S530;T501;	2.13E-04	NA	NA	NA	NA
Q86Y91	KIF18B	NA	NA	S674;	4.14E-05	S413;S674;	1.17E-05	NA	3.53E+00
Q86YR5	GPSM1	NA	NA	S492;S493;	8.96E-06	S492;S493;	6.08E-05	NA	1.48E-01
Q8IU60	DCP2	NA	NA	S247;S249;S254;S246;	9.99E-05	NA	NA	NA	NA
Q8IV36	HID1	NA	NA	S653;	5.07E-05	NA	NA	NA	NA
Q8IV63	VRK3	NA	NA	S78;S83;S85;S75;S82;S59;	8.74E-05	S82;S83;S85;	5.60E-06	NA	1.56E+01
Q8IVT5	KSR1	NA	NA	S569;S406;S314;S311;	7.71E-05	NA	NA	NA	NA
Q8IWB9	TEX2	NA	NA	S222;S266;	3.15E-05	NA	NA	NA	NA
Q8IWU2	LMTK2	NA	NA	S1107;S1310;	2.96E-05	NA	NA	NA	NA
Q8IWW6	ARHGAP12	NA	NA	S215;S213;S240;S201;	6.24E-04	S201;T231;S240;S215;	2.08E-04	NA	2.99E+00
Q8IX07	ZFPM1	NA	NA	S668;S901;S909;S786;	2.53E-05	NA	NA	NA	NA
Q8IY67	RAVER1	NA	NA	T463;	2.57E-05	T463;S17;	5.96E-05	NA	4.32E-01
Q8N108	MIER1	NA	NA	S160;S166;S483;S488;	3.34E-05	S160;S166;Y155;S483;S488;T158;S492;	1.10E-04	NA	3.03E-01
Q8N1G2	CMTR1	NA	NA	S51;S53;S55;T57;S31;S63;S64;	7.96E-05	S51;S53;S55;	5.00E-06	NA	1.59E+01
Q8N2F6	ARMC10	NA	NA	NA	NA	S45;	1.38E-05	NA	NA
Q8N4S9	MARVELD2	NA	NA	S116;S120;	5.60E-05	NA	NA	NA	NA
Q8N5A5	ZGPAT	NA	NA	T271;S280;S373;S276;S278;	3.59E-05	NA	NA	NA	NA
Q8N5V2	NGEF	NA	NA	S63;S16;	1.78E-05	NA	NA	NA	NA
Q8N9M1	C19orf47	NA	NA	S280;	2.88E-05	S280;	1.39E-05	NA	2.07E+00
Q8NA72	POC5	NA	NA	S105;S109;	1.21E-05	NA	NA	NA	NA
Q8NC56	LEMD2	NA	NA	S134;S138;S139;S499;S82;	1.57E-04	S499;S174;S175;S134;S138;S139;	1.50E-05	NA	1.04E+01
Q8ND76	CCNY	NA	NA	S326;	1.61E-04	S25;S326;	4.82E-05	NA	3.35E+00
Q8NE01	CNNM3	NA	NA	S700;	5.12E-05	NA	NA	NA	NA
Q8NEN9	PDZD8	NA	NA	S975;S980;S989;T982;S521;	7.50E-05	NA	NA	NA	NA
Q8NFG4	FLCN	NA	NA	S62;S161;S302;	2.38E-05	S302;S62;S73;	2.35E-05	NA	1.01E+00
Q8TAP8	PPP1R35	NA	NA	S52;	1.52E-05	S52;	3.07E-05	NA	4.94E-01

Q8TAX0	OSR1	NA	NA	T154;S157;	3.43E-06	NA	NA	NA	NA
Q8TB61	SLC35B2	NA	NA	S427;	8.61E-05	S427;	8.47E-05	NA	1.02E+00
Q8TBA6	GOLGA5	NA	NA	S116;	6.77E-06	NA	NA	NA	NA
Q8TC20	CAGE1	NA	NA	S190;S197;T204;	3.56E-04	NA	NA	NA	NA
Q8TCU6	PREX1	NA	NA	S1559;T1585;Y496;T500;S512;	5.71E-05	NA	NA	NA	NA
Q8TD19	NEK9	NA	NA	S868;S29;S331;	3.12E-04	S868;S29;S869;T333;	1.82E-04	NA	1.71E+00
Q8TDZ2	MICAL1	NA	NA	S872;S875;S876;	2.20E-05	NA	NA	NA	NA
Q8TE67	EPS8L3	NA	NA	S444;S445;S231;S520;S420;T511;S518;S441;	2.16E-04	NA	NA	NA	NA
Q8TEH3	DENND1A	NA	NA	S592;	8.19E-06	S592;S523;	1.13E-05	NA	7.24E-01
Q8TEQ6	GEMIN5	NA	NA	NA	NA	S778;	4.58E-05	NA	NA
Q8TEU7	RAPGEF6	NA	NA	NA	NA	S1590;S230;	1.16E-04	NA	NA
Q8WTW3	COG1	NA	NA	S459;	1.95E-05	S459;	6.07E-06	NA	3.21E+00
Q8WUA2	PPIL4	NA	NA	S178;	7.85E-05	T182;S178;	8.52E-05	NA	9.22E-01
Q8WUU4	ZNF296	NA	NA	S8;	1.81E-06	NA	NA	NA	NA
Q8WV19	SFT2D1	NA	NA	S9;	4.46E-05	NA	NA	NA	NA
Q8WX92	NELFB	NA	NA	S557;	2.66E-04	S557;	5.22E-04	NA	5.10E-01
Q8WYQ5	DGCR8	NA	NA	S377;	2.05E-05	S373;S377;	2.72E-05	NA	7.53E-01
Q8WZ73	RFFL	NA	NA	S242;S226;S229;	1.86E-04	T224;S232;S226;S229;	3.00E-05	NA	6.20E+00
Q92539	LPIN2	NA	NA	S186;S187;S243;	3.36E-05	NA	NA	NA	NA
Q92540	SMG7	NA	NA	S781;	2.33E-05	S781;	7.80E-06	NA	2.99E+00
Q92610	ZNF592	NA	NA	NA	NA	S573;S689;S142;S145;S146;S460;	2.80E-05	NA	NA
Q92618	ZNF516	NA	NA	S116;	2.82E-05	NA	NA	NA	NA
Q92625	ANKS1A	NA	NA	S663;	7.57E-05	S663;	4.17E-05	NA	1.82E+00
Q92786	PROX1	NA	NA	S511;S514;	6.59E-04	NA	NA	NA	NA
Q92804	TAF15	NA	NA	S228;S231;T235;S226;	5.40E-05	NA	NA	NA	NA
Q92925	SMARCD2	NA	NA	T217;	5.16E-06	NA	NA	NA	NA
Q969X1	TMBIM1	NA	NA	S81;	1.31E-05	NA	NA	NA	NA
Q96A57	TMEM230	NA	NA	S24;	2.00E-05	NA	NA	NA	NA

Q96AC1	FERMT2	NA	NA	S159;	4.10E-05	S159;S181;	1.29E-04	NA	3.19E-01
Q96AQ6	PBXIP1	NA	NA	S43;	9.72E-05	NA	NA	NA	NA
Q96B21	TMEM45B	NA	NA	S270;S272;	6.65E-04	NA	NA	NA	NA
Q96BY6	DOCK10	NA	NA	S1602;	2.77E-04	NA	NA	NA	NA
Q96BZ8	LENG1	NA	NA	S59;	4.73E-05	S59;	4.24E-05	NA	1.12E+00
Q96CV9	OPTN	NA	NA	S342;	2.47E-05	NA	NA	NA	NA
Q96DR7	ARHGEF26	NA	NA	S222;S392;	3.26E-05	NA	NA	NA	NA
Q96EZ8	MCRS1	NA	NA	S282;	2.56E-05	S282;	1.88E-05	NA	1.36E+00
Q96FC7	PHYHIPL	NA	NA	S12;S15;	1.01E-04	NA	NA	NA	NA
Q96FF7	LOC113230	NA	NA	S91;	9.33E-06	NA	NA	NA	NA
Q96FV9	THOC1	NA	NA	S560;	1.96E-05	S560;	7.58E-05	NA	2.58E-01
Q96GE4	CEP95	NA	NA	S449;S451;S453;	8.05E-06	NA	NA	NA	NA
Q96GY3	LIN37	NA	NA	S137;S138;	4.75E-05	S135;S138;	4.30E-05	NA	1.10E+00
Q96HA1	POM121	NA	NA	S697;S184;S188;	8.26E-05	NA	NA	NA	NA
Q96HE9	PRR11	NA	NA	S355;	1.31E-05	NA	NA	NA	NA
Q96I15	SCLY	NA	NA	S129;	1.13E-05	NA	NA	NA	NA
Q96JG6	VPS50	NA	NA	S559;S561;Y557;S494;S498;	1.16E-04	S561;S494;S498;Y557;S559;	1.17E-04	NA	9.90E-01
Q96KC8	DNAJC1	NA	NA	S430;S479;S480;S484;	1.70E-04	NA	NA	NA	NA
Q96N67	DOCK7	NA	NA	S1432;S1438;S894;T907;S910;S898;S900;S1430;S896;S439;S1434;S440;	6.32E-05	S1432;S900;S440;S910;	9.00E-05	NA	7.02E-01
Q96NT5	SLC46A1	NA	NA	S458;	5.83E-05	S458;	6.69E-05	NA	8.72E-01
Q96NU1	SAMD11	NA	NA	S469;S640;	6.38E-05	NA	NA	NA	NA
Q96Q15	SMG1	NA	NA	S34;T3577;	3.21E-05	T3573;S3576;T3577;	2.74E-05	NA	1.17E+00
Q96Q42	ALS2	NA	NA	S483;S492;	3.22E-05	NA	NA	NA	NA
Q96SB4	SRPK1	NA	NA	S51;S33;S309;S311;	7.90E-04	S51;S33;S39;S309;S311;S37;	1.45E-03	NA	5.45E-01
Q96T76	MMS19	NA	NA	NA	NA	S1027;	4.03E-06	NA	NA
Q99460	PSMD1	NA	NA	T311;S315;	6.16E-05	T311;S315;T270;T273;	4.91E-05	NA	1.25E+00
Q99567	NUP88	NA	NA	S517;S35;	2.82E-05	S35;S517;	1.12E-04	NA	2.51E-01

Q99661	KIF2C	NA	NA	S187;	1.76E-05	NA	NA	NA	NA
Q99856	ARID3A	NA	NA	S77;S81;S88;S101;T98;	3.83E-03	NA	NA	NA	NA
Q99956	DUSP9	NA	NA	S356;S361;S364;S16;S375;S351;	4.92E-04	S351;S375;S16;S368;	1.55E-04	NA	3.17E+00
Q9BPX7	C7orf25	NA	NA	S208;S210;S212;	2.06E-05	NA	NA	NA	NA
Q9BST9	RTKN	NA	NA	S520;S529;S220;S543;	6.76E-05	NA	NA	NA	NA
Q9BTD8	RBM42	NA	NA	S135;	2.33E-05	NA	NA	NA	NA
Q9BUH6	C9orf142	NA	NA	NA	NA	S148;S152;	2.51E-04	NA	NA
Q9BUZ4	TRAF4	NA	NA	S426;	8.98E-06	NA	NA	NA	NA
Q9BWH2	FUNDC2	NA	NA	S151;S10;	1.94E-05	S151;	2.11E-05	NA	9.17E-01
Q9BX63	BRIP1	NA	NA	S1032;	1.87E-05	NA	NA	NA	NA
Q9BXI6	TBC1D10A	NA	NA	S43;S45;S39;S40;	2.39E-05	NA	NA	NA	NA
Q9BYG3	NIFK	NA	NA	S247;	1.31E-05	S247;	2.81E-05	NA	4.67E-01
Q9BZ23	PANK2	NA	NA	S168;S189;	9.24E-05	S189;	3.62E-05	NA	2.55E+00
Q9BZ29	DOCK9	NA	NA	S21;	1.90E-05	NA	NA	NA	NA
Q9BZF1	OSBPL8	NA	NA	S808;S810;S814;S807;	1.54E-04	S63;S807;S808;S810;	4.90E-05	NA	3.14E+00
Q9BZL6	PRKD2	NA	NA	S710;S197;T199;S200;	1.75E-05	NA	NA	NA	NA
Q9C0E2	XPO4	NA	NA	NA	NA	S521;	9.03E-05	NA	NA
Q9C0H6	KLHL4	NA	NA	S414;S426;T427;	4.61E-05	T153;S414;	2.15E-06	NA	2.15E+01
Q9GZT9	EGLN1	NA	NA	NA	NA	S125;	1.51E-05	NA	NA
Q9H019	MTFR1L	NA	NA	S103;	6.23E-05	S103;	3.61E-05	NA	1.73E+00
Q9H0B6	KLC2	NA	NA	S589;S582;	2.98E-05	S589;S582;S581;S445;	3.39E-05	NA	8.80E-01
Q9H1A4	ANAPC1	NA	NA	S686;S547;S555;S563;	8.63E-06	S688;S345;	2.29E-05	NA	3.77E-01
Q9H330	TMEM245	NA	NA	S332;S16;	6.67E-05	NA	NA	NA	NA
Q9H3P7	ACBD3	NA	NA	S20;S47;S43;	8.44E-05	NA	NA	NA	NA
Q9H4A6	GOLPH3	NA	NA	S35;S36;	4.64E-05	S35;S36;	2.35E-05	NA	1.97E+00
Q9H4L5	OSBPL3	NA	NA	S304;S437;	6.03E-05	NA	NA	NA	NA
Q9H6H4	REEP4	NA	NA	S194;T196;S202;	1.02E-04	S194;T196;S202;	3.02E-05	NA	3.36E+00
Q9H6S0	YTHDC2	NA	NA	S1202;	3.28E-05	S1202;	4.87E-05	NA	6.74E-01
Q9H6S3	EPS8L2	NA	NA	S240;S479;	3.65E-05	T469;S459;S479;S240;T572;	1.84E-04	NA	1.98E-01
Q9H792	PEAK1	NA	NA	S568;S572;T574;T582;	8.89E-06	S568;	1.97E-05	NA	4.51E-01

Q9H8U3	ZFAND3	NA	NA	S129;S122;S124;	1.35E-05	NA	NA	NA	NA
Q9H9C1	VIPAS39	NA	NA	S121;	1.41E-05	NA	NA	NA	NA
Q9HA82	CERS4	NA	NA	S342;S347;S350;	4.76E-04	NA	NA	NA	NA
Q9HB58	SP110	NA	NA	S380;S256;	5.76E-05	S256;S380;	5.26E-05	NA	1.09E+00
Q9HCE1	MOV10	NA	NA	S969;	9.19E-05	NA	NA	NA	NA
Q9HCE5	METTL14	NA	NA	S399;	4.06E-06	NA	NA	NA	NA
Q9HCG8	CWC22	NA	NA	S829;S27;S28;Y33;S102;T104;S106;S93;	1.92E-04	S829;S91;S93;S862;S27;S28;	1.18E-04	NA	1.63E+00
Q9HCM4	EPB41L5	NA	NA	S422;S436;T439;S418;	1.76E-04	NA	NA	NA	NA
Q9NP71	MLXIPL	NA	NA	S23;S25;T27;S361;S631;	4.79E-05	NA	NA	NA	NA
Q9NQG6	MIEF1	NA	NA	T58;T61;S59;	3.86E-05	NA	NA	NA	NA
Q9NQP4	PFDN4	NA	NA	S125;	3.48E-05	S125;	2.53E-05	NA	1.38E+00
Q9NQT8	KIF13B	NA	NA	S1410;S1381;S1797;	8.07E-05	NA	NA	NA	NA
Q9NR09	BIRC6	NA	NA	NA	NA	S480;S490;	4.69E-05	NA	NA
Q9NRF2	SH2B1	NA	NA	S88;S96;	9.99E-05	S88;S96;	1.63E-05	NA	6.14E+00
Q9NRY5	FAM114A2	NA	NA	S145;	2.52E-05	NA	NA	NA	NA
Q9NRZ9	HELLS	NA	NA	NA	NA	S832;S833;	1.29E-05	NA	NA
Q9NUN5	LMBRD1	NA	NA	S528;S531;	1.43E-04	S528;S531;	3.71E-05	NA	3.85E+00
Q9NUQ6	SPATS2L	NA	NA	S120;S455;S531;S159;	2.65E-04	NA	NA	NA	NA
Q9NV70	EXOC1	NA	NA	S470;	4.61E-05	NA	NA	NA	NA
Q9NVU7	SDAD1	NA	NA	S585;	2.10E-05	S585;	4.58E-05	NA	4.58E-01
Q9NW97	TMEM51	NA	NA	S192;S155;S160;	1.47E-05	S192;T104;S115;	2.78E-05	NA	5.29E-01
Q9NWW5	CLN6	NA	NA	S31;	2.83E-05	NA	NA	NA	NA
Q9NWZ5	UCKL1	NA	NA	S539;	5.10E-05	S16;S56;S539;	7.92E-05	NA	6.44E-01
Q9NXC5	MIOS	NA	NA	S766;	6.95E-05	S766;	4.59E-05	NA	1.51E+00
Q9NXD2	MTMR10	NA	NA	Y224;S607;	2.02E-05	NA	NA	NA	NA
Q9NYF3	FAM53C	NA	NA	S273;S232;S234;	3.12E-05	S232;S234;S273;	3.61E-05	NA	8.62E-01
Q9NYJ8	TAB2	NA	NA	S450;	3.70E-05	NA	NA	NA	NA
Q9NYL9	TMOD3	NA	NA	S25;	1.49E-05	S25;	6.72E-05	NA	2.22E-01
Q9NYV6	RRN3	NA	NA	S170;S172;	1.10E-04	S170;S172;	3.64E-04	NA	3.03E-01
Q9NZN5	ARHGEF12	NA	NA	S637;	1.08E-05	S1068;S1077;S637;	7.17E-06	NA	1.51E+00

Q9P1Y5	CAMSAP3	NA	NA	S341;S351;S554;S814;S334;S349;S350;S347;S769;S431;S193;	3.39E-04	NA	NA	NA	NA
Q9P219	CCDC88C	NA	NA	S2007;S2008;	3.17E-06	NA	NA	NA	NA
Q9P270	SLAIN2	NA	NA	S48;S63;S391;S302;	3.51E-04	S87;S48;S302;S63;	2.15E-05	NA	1.64E+01
Q9P2K5	MYEF2	NA	NA	S17;	2.08E-04	NA	NA	NA	NA
Q9P2M7	CGN	NA	NA	S159;S162;S131;S208;S134;S1169;S1170;	1.69E-04	NA	NA	NA	NA
Q9UBM7	DHCR7	NA	NA	S14;	2.06E-05	NA	NA	NA	NA
Q9UBT2	UBA2	NA	NA	S592;	4.12E-05	NA	NA	NA	NA
Q9UGP4	LIMD1	NA	NA	S421;S424;S272;S277;S233;	4.66E-05	S424;S421;S233;S277;S423;S145;	1.95E-05	NA	2.39E+00
Q9UHR4	BAIAP2L1	NA	NA	S414;T412;	6.12E-05	T416;	1.36E-05	NA	4.51E+00
Q9UIK4	DAPK2	NA	NA	S349;	2.79E-05	NA	NA	NA	NA
Q9UIS9	MBD1	NA	NA	S297;	1.60E-05	S297;	2.23E-05	NA	7.17E-01
Q9UJX2	CDC23	NA	NA	T562;T582;S576;S578;	8.00E-05	S576;S578;T596;S588;	2.52E-04	NA	3.18E-01
Q9UJX5	ANAPC4	NA	NA	S777;S779;	1.85E-05	S777;S779;	5.34E-05	NA	3.45E-01
Q9UJX6	ANAPC2	NA	NA	S470;	8.97E-05	NA	NA	NA	NA
Q9UKA8	RCAN3	NA	NA	S148;S152;	5.26E-05	S148;S152;	3.70E-05	NA	1.42E+00
Q9UKV5	AMFR	NA	NA	S523;	3.73E-05	NA	NA	NA	NA
Q9ULD4	BRPF3	NA	NA	S17;S19;S959;S962;S965;	6.30E-05	NA	NA	NA	NA
Q9ULJ8	PPP1R9A	NA	NA	S338;S840;S184;T185;	3.04E-05	NA	NA	NA	NA
Q9ULR3	PPM1H	NA	NA	S124;	4.86E-05	NA	NA	NA	NA
Q9UN36	NDRG2	NA	NA	T330;S332;S338;	3.00E-04	S332;S338;S335;T330;	8.71E-06	NA	3.45E+01
Q9UN76	SLC6A14	NA	NA	S21;	1.87E-04	NA	NA	NA	NA
Q9UN79	SOX13	NA	NA	S335;	4.44E-05	S335;	1.97E-05	NA	2.25E+00
Q9UNL2	SSR3	NA	NA	S105;	1.98E-05	NA	NA	NA	NA
Q9UPY3	DICER1	NA	NA	S413;S415;	2.75E-05	NA	NA	NA	NA
Q9UQ80	PA2G4	NA	NA	NA	NA	S361;S363;	7.41E-06	NA	NA
Q9UQB3	CTNND2	NA	NA	S474;S461;S267;S276;	5.06E-05	NA	NA	NA	NA
Q9Y261	FOXA2	NA	NA	S303;S306;Y405;Y411;T297;	2.91E-05	NA	NA	NA	NA
Q9Y2G1	MYRF	NA	NA	S294;S304;	1.42E-05	NA	NA	NA	NA

Q9Y2H0	DLGAP4	NA	NA	NA	NA	S716;S763;T975;S844;S854;	2.56E-05	NA	NA
Q9Y2H5	PLEKHA6	NA	NA	S777;S867;S455;S247;S251;	1.53E-04	S867;T779;S453;	1.50E-04	NA	1.02E+00
Q9Y2X9	ZNF281	NA	NA	S651;	3.90E-05	NA	NA	NA	NA
Q9Y343	SNX24	NA	NA	S113;S116;	2.23E-05	NA	NA	NA	NA
Q9Y385	UBE2J1	NA	NA	S266;	3.01E-04	NA	NA	NA	NA
Q9Y3E1	HDGFRP3	NA	NA	S121;S122;	4.07E-05	S121;S122;T117;	3.58E-05	NA	1.14E+00
Q9Y3L3	SH3BP1	NA	NA	S544;S596;S598;	4.33E-04	NA	NA	NA	NA
Q9Y467	SALL2	NA	NA	S797;S802;S806;	3.51E-05	NA	NA	NA	NA
Q9Y4F1	FARP1	NA	NA	S889;T24;S899;S872;S427;S336;S878;	1.72E-04	NA	NA	NA	NA
Q9Y4J8	DTNA	NA	NA	S662;	5.77E-05	NA	NA	NA	NA
Q9Y4P1	ATG4B	NA	NA	S383;	1.46E-05	NA	NA	NA	NA
Q9Y4U1	MMACHC	NA	NA	NA	NA	S245;	4.03E-05	NA	NA
Q9Y4W2	LAS1L	NA	NA	S560;	3.45E-05	NA	NA	NA	NA
Q9Y597	KCTD3	NA	NA	S711;	3.20E-05	NA	NA	NA	NA
Q9Y5B9	SUPT16H	NA	NA	NA	NA	S1023;	5.15E-05	NA	NA
Q9Y624	F11R	NA	NA	S284;S287;	1.06E-03	S284;	2.47E-05	NA	4.29E+01
Q9Y6I9	TEX264	NA	NA	S244;S248;	1.49E-05	NA	NA	NA	NA
Q9Y6R1	SLC4A4	NA	NA	T254;S262;S65;S68;	1.41E-05	NA	NA	NA	NA
Q9Y6R4	MAP3K4	NA	NA	S499;S72;S84;S1252;	2.07E-05	NA	NA	NA	NA
Q9Y6X4	FAM169A	NA	NA	S449;	1.71E-05	S376;S378;S379;S447;S449;	4.82E-05	NA	3.56E-01
Q9Y6X6	MYO16	NA	NA	S210;S211;S200;	3.16E-05	NA	NA	NA	NA
Q9Y6Y0	IVNS1ABP	NA	NA	S322;T328;	5.54E-05	S277;S322;	5.33E-05	NA	1.04E+00
C9JI98	TMEM238	NA	NA	S175;	1.10E-05	NA	NA	NA	NA
O00139	KIF2A	NA	NA	S140;	1.37E-05	NA	NA	NA	NA
O00472	ELL2	NA	NA	S305;	7.38E-06	NA	NA	NA	NA
O15085	ARHGEF11	NA	NA	NA	NA	T1292;S1413;S1155;S1458;T1461;S663;T66	4.96E-05	NA	NA

						8;T672;			
O43149	ZZEF1	NA	NA	S1518;T1521;S2443;	1.03E-04	NA	NA	NA	NA
O60292	SIPA1L3	NA	NA	S172;S1501;	4.68E-05	NA	NA	NA	NA
O60496	DOK2	NA	NA	S122;S129;	4.30E-05	NA	NA	NA	NA
O75530	EED	NA	NA	NA	NA	S34;T57;S29;T55;	1.01E-04	NA	NA
O94763	URI1	NA	NA	NA	NA	S440;S372;S442;	1.17E-04	NA	NA
O94806	PRKD3	NA	NA	NA	NA	S27;S31;S30;S41;	1.56E-05	NA	NA
O95292	VAPB	NA	NA	NA	NA	S206;S204;	1.55E-05	NA	NA
O95696	BRD1	NA	NA	T1051;S1055;	1.05E-04	T1051;S1055;	2.36E-05	NA	4.43E+00
P02686	MBP	NA	NA	S40;T38;	1.59E-04	NA	NA	NA	NA
P04183	TK1	NA	NA	NA	NA	S231;	7.17E-05	NA	NA
P07948	LYN	NA	NA	Y508;S13;	5.25E-05	S13;	8.48E-06	NA	6.19E+00
P17706	PTPN2	NA	NA	S304;	1.61E-05	S304;	5.22E-05	NA	3.09E-01
P26045	PTPN3	NA	NA	S425;S427;	1.78E-05	NA	NA	NA	NA
P32004	L1CAM	NA	NA	NA	NA	S1243;S1244;S1248;S793;T803;T538;	5.26E-05	NA	NA
P42677	RPS27	NA	NA	NA	NA	S78;	4.98E-05	NA	NA
P50851	LRBA	NA	NA	S1118;S1919;S1669;	8.25E-05	NA	NA	NA	NA
P55198	MLLT6	NA	NA	S218;S224;T225;S220;	5.40E-06	NA	NA	NA	NA
P56524	HDAC4	NA	NA	NA	NA	S632;S565;	7.08E-05	NA	NA
P68363	TUBA1B	NA	NA	S48;S340;	7.08E-05	S48;S340;S439;	3.08E-04	NA	2.30E-01
Q01201	RELB	NA	NA	NA	NA	S573;	1.28E-05	NA	NA
Q03001	DST	NA	NA	S7510;S7513;S7502;	4.62E-06	S7510;S7513;T5847;	5.15E-05	NA	8.97E-02
Q05655	PRKCD	NA	NA	NA	NA	S304;	2.59E-05	NA	NA
Q07065	CKAP4	NA	NA	S17;S19;T298;S299;S302;	2.90E-05	NA	NA	NA	NA
Q13443	ADAM9	NA	NA	NA	NA	S758;T761;	4.99E-05	NA	NA
Q14139	UBE4A	NA	NA	S57;S50;S53;	4.12E-06	NA	NA	NA	NA
Q14155	ARHGEF7	NA	NA	NA	NA	S761;S257;S249;S694;S676;S760;	1.49E-04	NA	NA
Q15223	PVRL1	NA	NA	S434;S435;	1.10E-05	NA	NA	NA	NA
Q15554	TERF2	NA	NA	S365;	2.23E-05	S365;	1.67E-05	NA	1.33E+00
Q4ADV7	RIC1	NA	NA	T992;S995;S1021;	3.68E-05	T992;S995;T996;	1.17E-05	NA	3.14E+00
Q5XKK7	FAM219	NA	NA	NA	NA	S125;S127;	1.27E-05	NA	NA

	B								
Q6DN90	IQSEC1	NA	NA	NA	NA	S512;	3.51E-05	NA	NA
Q7L8J4	SH3BP5L	NA	NA	S30;S343;	3.56E-05	NA	NA	NA	NA
Q86TN4	TRPT1	NA	NA	S240;	1.29E-04	S240;S239;S244;S245;	2.57E-05	NA	5.03E+00
Q86Y82	STX12	NA	NA	S142;	3.18E-05	NA	NA	NA	NA
Q8IWE2	FAM114 A1	NA	NA	S196;S200;T199;	1.53E-05	NA	NA	NA	NA
Q8N1F7	NUP93	NA	NA	NA	NA	S767;S769;	3.04E-04	NA	NA
Q8N5C8	TAB3	NA	NA	NA	NA	S506;	1.51E-05	NA	NA
Q8N655	C10orf12	NA	NA	S273;	4.14E-05	NA	NA	NA	NA
Q8N7R7	CCNYL1	NA	NA	NA	NA	S344;T125;Y120;	4.74E-05	NA	NA
Q8NEL9	DDHD1	NA	NA	NA	NA	S723;S731;T726;S727;	5.56E-05	NA	NA
Q8TEW0	PARD3	NA	NA	S809;S692;	2.01E-05	S695;S383;	2.25E-05	NA	8.93E-01
Q92785	DPF2	NA	NA	S142;	6.55E-05	NA	NA	NA	NA
Q93100	PHKB	NA	NA	NA	NA	T702;	7.02E-05	NA	NA
Q96GP6	SCARF2	NA	NA	S651;S653;	1.13E-05	NA	NA	NA	NA
Q96GX9	APIP	NA	NA	S89;	7.63E-05	S89;	1.05E-04	NA	7.26E-01
Q96LD4	TRIM47	NA	NA	NA	NA	S588;	3.90E-05	NA	NA
Q96P48	ARAP1	NA	NA	NA	NA	S229;Y231;	1.20E-04	NA	NA
Q96PU5	NEDD4L	NA	NA	S446;S448;S342;	1.91E-04	S479;S483;S487;S446;	1.16E-05	NA	1.65E+01
Q9BQA9	C17orf62	NA	NA	S178;S173;S175;S176;	5.63E-06	NA	NA	NA	NA
Q9BSJ8	ESYT1	NA	NA	S1034;	2.71E-05	NA	NA	NA	NA
Q9C086	INO80B	NA	NA	NA	NA	S130;S351;S132;	1.77E-05	NA	NA
Q9C0D6	FHDC1	NA	NA	S660;S664;	1.19E-05	NA	NA	NA	NA
Q9HB09	BCL2L12	NA	NA	NA	NA	S242;	6.72E-06	NA	NA
Q9NQQ7	SLC35C2	NA	NA	S335;	7.98E-06	S335;	9.93E-06	NA	8.04E-01
Q9NQX3	GPHN	NA	NA	S188;S194;S200;	6.83E-06	NA	NA	NA	NA
Q9NX63	CHCHD3	NA	NA	NA	NA	Y49;	4.70E-05	NA	NA
Q9UGY1	NOL12	NA	NA	NA	NA	S139;S140;T141;S134;	3.30E-05	NA	NA
Q9UHK0	NUFIP1	NA	NA	S338;S340;S342;	4.85E-05	S338;S340;S342;	3.35E-05	NA	1.45E+00
Q9UQR0	SCML2	NA	NA	S499;S511;S590;	2.33E-05	S499;S511;T503;S590;	1.14E-04	NA	2.05E-01
Q9Y320	TMX2	NA	NA	T285;S288;	7.96E-05	NA	NA	NA	NA
O75391	SPAG7	NA	NA	NA	NA	S158;	8.77E-05	NA	NA

O94864	SUPT7L	NA	NA	NA	NA	S108;	9.43E-05	NA	NA
O94929	ABLIM3	NA	NA	NA	NA	S373;S503;S504;	3.48E-05	NA	NA
P02751	FN1	NA	NA	NA	NA	S1527;S1530;T1786;	1.54E-04	NA	NA
P35613	BSG	NA	NA	NA	NA	S362;	7.54E-05	NA	NA
P41252	IARS	NA	NA	NA	NA	S1047;	1.00E-04	NA	NA
P42695	NCAPD3	NA	NA	NA	NA	S1384;S1382;	1.10E-05	NA	NA
Q05D32	CTDSPL2	NA	NA	NA	NA	S28;S134;S9;	2.39E-05	NA	NA
Q13309	SKP2	NA	NA	NA	NA	S64;S57;	4.29E-05	NA	NA
Q147X3	NAA30	NA	NA	NA	NA	S89;S39;S55;	1.10E-04	NA	NA
Q15596	NCOA2	NA	NA	NA	NA	S698;S771;	8.49E-06	NA	NA
Q53EU6	GPAT3	NA	NA	NA	NA	S68;	2.57E-05	NA	NA
Q5T011	SZT2	NA	NA	NA	NA	S1819;S1825;	1.35E-05	NA	NA
Q63HN8	RNF213	NA	NA	NA	NA	S1258;S2273;	1.72E-04	NA	NA
Q6R327	RICTOR	NA	NA	NA	NA	S21;	6.01E-05	NA	NA
Q86XL3	ANKLE2	NA	NA	NA	NA	S662;	1.38E-05	NA	NA
Q86YZ3	HRNR	NA	NA	NA	NA	S1001;S885;S540;	1.65E-05	NA	NA
Q8N2S1	LTBP4	NA	NA	NA	NA	S96;S1169;T1170;	1.16E-04	NA	NA
Q8NHG8	ZNRF2	NA	NA	NA	NA	S82;S135;	8.96E-06	NA	NA
Q8WU79	SMAP2	NA	NA	NA	NA	S219;	2.30E-05	NA	NA
Q96AD5	PNPLA2	NA	NA	NA	NA	S428;S404;	4.11E-05	NA	NA
Q96AP0	ACD	NA	NA	NA	NA	T422;S435;T431;S425;	1.92E-05	NA	NA
Q96QF0	RAB3IP	NA	NA	NA	NA	T268;T277;	1.99E-05	NA	NA
Q9GZR1	SENP6	NA	NA	NA	NA	S352;S919;	2.20E-05	NA	NA
Q9UBN7	HDAC6	NA	NA	NA	NA	S22;	8.07E-06	NA	NA
Q9UKI8	TLK1	NA	NA	NA	NA	S159;	2.44E-05	NA	NA
Q9UKT5	FBXO4	NA	NA	NA	NA	S12;	1.25E-05	NA	NA
Q9UP95	SLC12A4	NA	NA	NA	NA	S967;	6.17E-05	NA	NA
Q9UPS6	SETD1B	NA	NA	NA	NA	Y849;T1679;	1.18E-05	NA	NA
Q9Y5Y0	FLVCR1	NA	NA	NA	NA	S536;	3.61E-05	NA	NA
Q9Y6M7	SLC4A7	NA	NA	NA	NA	S84;S242;S407;T408;S400;S403;S233;	1.50E-04	NA	NA
Q9Y6R0	NUMBL	NA	NA	NA	NA	S263;S324;S411;	4.54E-05	NA	NA
A2RU67	FAM234	NA	NA	NA	NA	S62;S16;S30;S33;T26;	5.98E-05	NA	NA

	B								
A3KN83	SBNO1	NA	NA	NA	NA	S794;	6.87E-06	NA	NA
A7E2V4	ZSWIM8	NA	NA	NA	NA	S53;S1202;S48;	8.12E-06	NA	NA
O00148	DDX39A	NA	NA	NA	NA	T171;	6.35E-06	NA	NA
O00220	TNFRSF10A	NA	NA	NA	NA	T461;S466;	3.60E-06	NA	NA
O00470	MEIS1	NA	NA	NA	NA	S194;S196;	1.88E-05	NA	NA
O14578	CIT	NA	NA	NA	NA	S440;	2.45E-05	NA	NA
O14980	XPO1	NA	NA	NA	NA	S397;S391;S1031;	3.39E-05	NA	NA
O15020	SPTBN2	NA	NA	NA	NA	S2171;	4.61E-05	NA	NA
O15067	PFAS	NA	NA	NA	NA	S569;	1.68E-05	NA	NA
O15427	SLC16A3	NA	NA	NA	NA	S436;S464;	1.49E-05	NA	NA
O15440	ABCC5	NA	NA	NA	NA	S509;S505;	1.47E-05	NA	NA
O43294	TGFB1I1	NA	NA	NA	NA	S68;S140;S141;S143;	1.01E-04	NA	NA
O43310	CTIF	NA	NA	NA	NA	S299;	3.08E-05	NA	NA
O60287	URB1	NA	NA	NA	NA	S9;	2.90E-06	NA	NA
O75369	FLNB	NA	NA	NA	NA	S2107;S2478;S983;T980;	1.48E-04	NA	NA
O75533	SF3B1	NA	NA	NA	NA	T211;T278;	2.33E-05	NA	NA
O75683	SURF6	NA	NA	NA	NA	S74;	1.95E-05	NA	NA
O75822	EIF3J	NA	NA	NA	NA	S127;	1.79E-05	NA	NA
O94782	USP1	NA	NA	NA	NA	S475;S67;	1.79E-05	NA	NA
O95685	PPP1R3D	NA	NA	NA	NA	S28;S46;S77;S78;S74;	3.76E-05	NA	NA
O96019	ACTL6A	NA	NA	NA	NA	S233;	3.94E-05	NA	NA
P00519	ABL1	NA	NA	NA	NA	S569;S1035;T1036;S1044;S718;	2.11E-05	NA	NA
P01583	IL1A	NA	NA	NA	NA	S89;S104;S87;S16;	5.42E-05	NA	NA
P04626	ERBB2	NA	NA	NA	NA	S1054;	9.81E-06	NA	NA
P06400	RB1	NA	NA	NA	NA	S249;S788;S794;S37;	4.28E-05	NA	NA
P07355	ANXA2	NA	NA	NA	NA	S127;T19;S26;	1.89E-04	NA	NA
P08729	KRT7	NA	NA	NA	NA	S254;	2.09E-05	NA	NA
P09234	SNRPC	NA	NA	NA	NA	S17;	3.14E-05	NA	NA
P09874	PARP1	NA	NA	NA	NA	S782;	3.23E-05	NA	NA

P16403	HIST1H1C	NA	NA	NA	NA	S36;S173;T146;T31;	2.84E-05	NA	NA
P20020	ATP2B1	NA	NA	NA	NA	S1257;S1216;S17;T1203;S1249;	1.26E-04	NA	NA
P21580	TNFAIP3	NA	NA	NA	NA	S575;S573;	1.23E-05	NA	NA
P25445	FAS	NA	NA	NA	NA	S212;S209;	2.10E-05	NA	NA
P27540	ARNT	NA	NA	NA	NA	S77;	4.45E-05	NA	NA
P29317	EPHA2	NA	NA	NA	NA	S897;S901;	7.27E-05	NA	NA
P30050	RPL12	NA	NA	NA	NA	S38;	2.70E-05	NA	NA
P31323	PRKAR2B	NA	NA	NA	NA	S114;	3.52E-05	NA	NA
P33981	TTK	NA	NA	NA	NA	S281;S436;	4.82E-05	NA	NA
P45974	USP5	NA	NA	NA	NA	T367;T370;S785;	5.81E-05	NA	NA
P46777	RPL5	NA	NA	NA	NA	S286;	2.54E-06	NA	NA
P47712	PLA2G4A	NA	NA	NA	NA	S435;S437;S434;S729;S431;T447;T659;	1.10E-03	NA	NA
P48960	ADGRE5	NA	NA	NA	NA	S831;	1.75E-05	NA	NA
P51692	STAT5B	NA	NA	NA	NA	S193;	1.19E-05	NA	NA
P51784	USP11	NA	NA	NA	NA	S648;Y642;	4.56E-05	NA	NA
P51812	RPS6KA3	NA	NA	NA	NA	S715;T365;S369;	2.57E-04	NA	NA
P51946	CCNH	NA	NA	NA	NA	T315;S307;S322;	2.74E-05	NA	NA
P54278	PMS2	NA	NA	NA	NA	S522;	4.64E-05	NA	NA
P55211	CASP9	NA	NA	NA	NA	S302;S310;	1.66E-05	NA	NA
P57081	WDR4	NA	NA	NA	NA	S391;	5.00E-06	NA	NA
P61073	CXCR4	NA	NA	NA	NA	S339;S348;	2.21E-05	NA	NA
P62258	YWHAE	NA	NA	NA	NA	S210;	9.27E-06	NA	NA
P62328	TMSB4X	NA	NA	NA	NA	T23;	3.63E-05	NA	NA
P78346	RPP30	NA	NA	NA	NA	S251;	1.84E-04	NA	NA
P78563	ADARB1	NA	NA	NA	NA	S26;S458;S467;	1.37E-05	NA	NA
P86790	CCZ1B	NA	NA	NA	NA	S266;	8.89E-06	NA	NA
Q02790	FKBP4	NA	NA	NA	NA	S453;	7.54E-05	NA	NA
Q07352	ZFP36L1	NA	NA	NA	NA	S54;S92;	2.07E-05	NA	NA
Q08378	GOLGA3	NA	NA	NA	NA	S21;	1.79E-04	NA	NA
Q12948	FOXC1	NA	NA	NA	NA	T228;T232;S241;	5.35E-06	NA	NA

Q12965	MYO1E	NA	NA	NA	NA	S1002;S1005;S736;T1003;	4.19E-05	NA	NA
Q13098	GPS1	NA	NA	NA	NA	T479;S474;S468;	6.92E-05	NA	NA
Q13433	SLC39A6	NA	NA	NA	NA	S478;	5.51E-05	NA	NA
Q14106	TOB2	NA	NA	NA	NA	S75;	1.05E-05	NA	NA
Q14195	DPYSL3	NA	NA	NA	NA	S522;	3.07E-05	NA	NA
Q14699	RFTN1	NA	NA	NA	NA	S220;	5.69E-06	NA	NA
Q14746	COG2	NA	NA	NA	NA	S104;	4.36E-05	NA	NA
Q14764	MVP	NA	NA	NA	NA	S873;	6.36E-05	NA	NA
Q14789	GOLGB1	NA	NA	NA	NA	S3010;	7.22E-05	NA	NA
Q15003	NCAPH	NA	NA	NA	NA	T598;S28;	4.47E-06	NA	NA
Q15024	EXOSC7	NA	NA	NA	NA	S177;	3.20E-05	NA	NA
Q15386	UBE3C	NA	NA	NA	NA	S379;S380;S372;	1.85E-05	NA	NA
Q15388	TOMM20	NA	NA	NA	NA	S135;S138;	6.05E-06	NA	NA
Q15434	RBMS2	NA	NA	NA	NA	S106;	1.66E-05	NA	NA
Q15464	SHB	NA	NA	NA	NA	S312;S317;S307;	3.05E-05	NA	NA
Q15758	SLC1A5	NA	NA	NA	NA	S503;	1.66E-05	NA	NA
Q2TB10	ZNF800	NA	NA	NA	NA	S336;	1.64E-05	NA	NA
Q3MII6	TBC1D25	NA	NA	NA	NA	S506;	1.74E-05	NA	NA
Q3V6T2	CCDC88A	NA	NA	NA	NA	T1673;S1439;	3.78E-05	NA	NA
Q5QP82	DCAF10	NA	NA	NA	NA	S92;S89;	1.19E-05	NA	NA
Q5TG92	C1orf195	NA	NA	NA	NA	S29;S24;T25;	1.32E-05	NA	NA
Q5VT25	CDC42BPA	NA	NA	NA	NA	S1654;S1719;S1651;	1.48E-05	NA	NA
Q5VTB9	RNF220	NA	NA	NA	NA	S390;T401;	5.63E-05	NA	NA
Q5VYK3	KIAA0368	NA	NA	NA	NA	S833;	3.12E-05	NA	NA
Q5W0B1	RNF219	NA	NA	NA	NA	S719;S210;	2.07E-05	NA	NA
Q659A1	ICE2	NA	NA	NA	NA	S570;S571;T573;	8.01E-06	NA	NA
Q68E01	INTS3	NA	NA	NA	NA	S502;	1.37E-05	NA	NA
Q6DD88	ATL3	NA	NA	NA	NA	S149;	1.22E-05	NA	NA
Q6IAA8	LAMTOR1	NA	NA	NA	NA	S27;S26;	1.68E-05	NA	NA

Q6IQ26	DENND5A	NA	NA	NA	NA	S193;T1079;S1087;	4.52E-05	NA	NA
Q6P1M3	LLGL2	NA	NA	NA	NA	Y275;T284;	1.04E-05	NA	NA
Q6P4E1	CASC4	NA	NA	NA	NA	S366;S232;	1.54E-05	NA	NA
Q6PJG9	LRFN4	NA	NA	NA	NA	S626;S585;	1.42E-05	NA	NA
Q6PK04	CCDC137	NA	NA	NA	NA	S19;	7.21E-05	NA	NA
Q6Q0C0	TRAF7	NA	NA	NA	NA	S108;	7.97E-06	NA	NA
Q6ZV73	FGD6	NA	NA	NA	NA	S1197;	2.07E-05	NA	NA
Q70CQ2	USP34	NA	NA	NA	NA	S3410;S3406;T3390;	3.34E-05	NA	NA
Q7Z2T5	TRMT1L	NA	NA	NA	NA	S66;	5.20E-05	NA	NA
Q86UE8	TLK2	NA	NA	NA	NA	S223;S99;	1.26E-05	NA	NA
Q86VP1	TAX1BP1	NA	NA	NA	NA	S666;	1.25E-05	NA	NA
Q86VX9	MON1A	NA	NA	NA	NA	S56;Y57;	2.24E-05	NA	NA
Q86WR7	PROSER2	NA	NA	NA	NA	S43;T146;S166;S179;	1.24E-04	NA	NA
Q8IYH5	ZZZ3	NA	NA	NA	NA	S130;S131;S135;S89;	2.10E-05	NA	NA
Q8N201	INTS1	NA	NA	NA	NA	S1326;S1318;	1.12E-04	NA	NA
Q8N3F8	MICALL1	NA	NA	NA	NA	S621;S640;S644;S486;S619;	9.76E-05	NA	NA
Q8N5P1	ZC3H8	NA	NA	NA	NA	Y76;S22;	9.61E-05	NA	NA
Q8N5Y2	MSL3	NA	NA	NA	NA	S407;S400;	8.86E-06	NA	NA
Q8NBB4	ZSCAN1	NA	NA	NA	NA	S247;	2.49E-06	NA	NA
Q8ND82	ZNF280C	NA	NA	NA	NA	T540;S80;	1.93E-05	NA	NA
Q8NEM2	SHCBP1	NA	NA	NA	NA	S42;S44;	3.68E-05	NA	NA
Q8TAA9	VANGL1	NA	NA	NA	NA	S86;S88;	1.01E-05	NA	NA
Q8TEV9	SMCR8	NA	NA	NA	NA	S487;S492;S498;T511;S513;	2.83E-05	NA	NA
Q8TF72	SHROOM3	NA	NA	NA	NA	S910;S913;S1279;S1441;	2.74E-05	NA	NA
Q8WWY3	PRPF31	NA	NA	NA	NA	T455;S445;S446;T448;	4.07E-05	NA	NA
Q8WYA6	CTNNBL1	NA	NA	NA	NA	S545;	2.31E-05	NA	NA
Q92817	EVPL	NA	NA	NA	NA	S973;	3.43E-05	NA	NA
Q92887	ABCC2	NA	NA	NA	NA	S283;	1.55E-05	NA	NA
Q92917	GPKOW	NA	NA	NA	NA	S42;	3.33E-05	NA	NA
Q93008	USP9X	NA	NA	NA	NA	S2563;	2.59E-05	NA	NA

Q969E4	TCEAL3	NA	NA	NA	NA	S30;	9.31E-05	NA	NA
Q96CX2	KCTD12	NA	NA	NA	NA	S187;S171;S176;	2.44E-05	NA	NA
Q96DF8	DGCR14	NA	NA	NA	NA	S292;	1.00E-05	NA	NA
Q96DX7	TRIM44	NA	NA	NA	NA	S336;S339;T319;S320;	2.33E-05	NA	NA
Q96EN8	MOCOS	NA	NA	NA	NA	S530;	2.58E-05	NA	NA
Q96EY5	MVB12A	NA	NA	NA	NA	S18;S19;S21;	9.53E-06	NA	NA
Q96F63	CCDC97	NA	NA	NA	NA	S337;	2.06E-05	NA	NA
Q96JK2	DCAF5	NA	NA	NA	NA	S651;S648;S794;	1.70E-05	NA	NA
Q96K76	USP47	NA	NA	NA	NA	S1013;T1015;S832;S933;S1017;S910;	1.15E-04	NA	NA
Q96LA8	PRMT6	NA	NA	NA	NA	T21;	5.03E-06	NA	NA
Q96LW4	PRIMPOL	NA	NA	NA	NA	S255;	1.35E-05	NA	NA
Q96N20	ZNF75A	NA	NA	NA	NA	S287;	9.32E-05	NA	NA
Q96PU4	UHRF2	NA	NA	NA	NA	S667;	1.83E-05	NA	NA
Q96RE7	NACC1	NA	NA	NA	NA	S145;	2.51E-05	NA	NA
Q96RS0	TGS1	NA	NA	NA	NA	S55;	1.73E-05	NA	NA
Q96S90	LYSMD1	NA	NA	NA	NA	S99;	2.05E-05	NA	NA
Q96SI1	KCTD15	NA	NA	NA	NA	S35;S38;	1.01E-05	NA	NA
Q96SU4	OSBPL9	NA	NA	NA	NA	S326;S329;	4.12E-05	NA	NA
Q99543	DNAJC2	NA	NA	NA	NA	S47;S49;	2.81E-05	NA	NA
Q9BQQ3	GORASP1	NA	NA	NA	NA	T216;T237;S220;S241;S248;	2.45E-05	NA	NA
Q9BUI4	POLR3C	NA	NA	NA	NA	S204;S205;	1.87E-05	NA	NA
Q9BXW9	FANCD2	NA	NA	NA	NA	S592;	3.95E-05	NA	NA
Q9BYI3	FAM126A	NA	NA	NA	NA	S453;	1.61E-05	NA	NA
Q9BYX2	TBC1D2	NA	NA	NA	NA	S920;S915;	6.20E-05	NA	NA
Q9BZQ8	FAM129A	NA	NA	NA	NA	S926;S622;S602;	5.13E-05	NA	NA
Q9H694	BICC1	NA	NA	NA	NA	S612;	2.58E-05	NA	NA
Q9H6E5	TUT1	NA	NA	NA	NA	T642;S644;	6.55E-06	NA	NA
Q9H6S1	AZI2	NA	NA	NA	NA	S318;	9.05E-06	NA	NA
Q9H7M9	C10orf54	NA	NA	NA	NA	S235;	1.65E-05	NA	NA
Q9H930	SP140L	NA	NA	NA	NA	S180;	8.10E-06	NA	NA

Q9HB19	PLEKHA2	NA	NA	NA	NA	S184;T195;S202;Y204;S401;S314;	7.70E-05	NA	NA
Q9HCD6	TANC2	NA	NA	NA	NA	S1827;S1579;T1315;S1722;	7.72E-05	NA	NA
Q9HCS5	EPB41L4A	NA	NA	NA	NA	S402;S445;S541;S543;	5.78E-06	NA	NA
Q9NP74	PALMD	NA	NA	NA	NA	S384;S385;	1.68E-05	NA	NA
Q9NR31	SAR1A	NA	NA	NA	NA	T139;	1.68E-05	NA	NA
Q9NVN3	RIC8B	NA	NA	NA	NA	S468;T473;	1.61E-05	NA	NA
Q9NYM9	BET1L	NA	NA	NA	NA	S9;	1.69E-05	NA	NA
Q9NZD8	SPG21	NA	NA	NA	NA	T252;Y258;S304;	8.16E-06	NA	NA
Q9P0V3	SH3BP4	NA	NA	NA	NA	S246;S296;	1.60E-05	NA	NA
Q9P2F8	SIPA1L2	NA	NA	NA	NA	S1488;	1.32E-05	NA	NA
Q9UBG0	MRC2	NA	NA	NA	NA	S1457;	2.18E-04	NA	NA
Q9UBK8	MTRR	NA	NA	NA	NA	S198;S202;	5.45E-05	NA	NA
Q9UHW9	SLC12A6	NA	NA	NA	NA	S1029;S1032;	1.97E-05	NA	NA
Q9UKE5	TNIK	NA	NA	NA	NA	S678;S680;S640;S769;S766;	4.02E-05	NA	NA
Q9UNX4	WDR3	NA	NA	NA	NA	S241;	1.57E-04	NA	NA
Q9UPY5	SLC7A11	NA	NA	NA	NA	S26;	1.30E-04	NA	NA
Q9Y2D8	SSX2IP	NA	NA	NA	NA	S312;	3.44E-06	NA	NA
Q9Y2J4	AMOTL2	NA	NA	NA	NA	T45;S537;	9.42E-06	NA	NA
Q9Y2U5	MAP3K2	NA	NA	NA	NA	S239;S163;	8.70E-05	NA	NA
Q9Y3P9	RABGAP1	NA	NA	NA	NA	T996;	5.32E-05	NA	NA
Q9Y4H2	IRS2	NA	NA	NA	NA	S915;S1203;S1186;T520;S1162;S1174;S560;S577;S932;S620;	9.28E-05	NA	NA
Q9Y4P8	WIPI2	NA	NA	NA	NA	T415;S412;	1.06E-04	NA	NA
Q9Y4X0	AMMECR1	NA	NA	NA	NA	S14;S16;	1.21E-05	NA	NA
Q9Y666	SLC12A7	NA	NA	NA	NA	T30;	2.37E-04	NA	NA
Q9Y6Y8	SEC23IP	NA	NA	NA	NA	S926;	1.68E-05	NA	NA
O00159	MYO1C	NA	NA	NA	NA	S408;	7.38E-05	NA	NA
O14683	TP53I11	NA	NA	NA	NA	S14;	8.01E-06	NA	NA

O15379	HDAC3	NA	NA	NA	NA	S424;	4.42E-05	NA	NA
O75592	MYCBP2	NA	NA	NA	NA	S3467;	2.82E-05	NA	NA
O94819	KBTBD11	NA	NA	NA	NA	S310;S314;S316;	4.51E-05	NA	NA
O95456	PSMG1	NA	NA	NA	NA	T18;	1.57E-05	NA	NA
P03956	MMP1	NA	NA	NA	NA	Y411;	8.03E-05	NA	NA
P0C7T5	ATXN1L	NA	NA	NA	NA	S284;	4.81E-06	NA	NA
P15391	CD19	NA	NA	NA	NA	S208;S145;S146;S148;	2.02E-05	NA	NA
P16070	CD44	NA	NA	NA	NA	S697;	1.20E-05	NA	NA
P29323	EPHB2	NA	NA	NA	NA	S776;	5.59E-05	NA	NA
P29558	RBMS1	NA	NA	NA	NA	S112;	1.73E-05	NA	NA
P31949	S100A11	NA	NA	NA	NA	S6;	6.65E-05	NA	NA
P46109	CRKL	NA	NA	NA	NA	Y207;S222;	6.60E-05	NA	NA
P49759	CLK1	NA	NA	NA	NA	S140;	1.99E-05	NA	NA
P51580	TPMT	NA	NA	NA	NA	S14;	2.19E-05	NA	NA
P55201	BRPF1	NA	NA	NA	NA	S460;S462;S860;	3.36E-05	NA	NA
P83369	LSM11	NA	NA	NA	NA	S21;S15;	7.68E-05	NA	NA
Q05397	PTK2	NA	NA	NA	NA	S910;	1.62E-05	NA	NA
Q12893	TMEM115	NA	NA	NA	NA	T329;	4.16E-05	NA	NA
Q13029	PRDM2	NA	NA	NA	NA	S164;S165;S168;S1572;	1.21E-06	NA	NA
Q13099	IFT88	NA	NA	NA	NA	Y318;T321;Y324;S161;S166;	3.89E-06	NA	NA
Q15652	JMJD1C	NA	NA	NA	NA	S639;S641;S652;	1.46E-05	NA	NA
Q53GS7	GLE1	NA	NA	NA	NA	S367;S88;	2.47E-05	NA	NA
Q5EBL4	RILPL1	NA	NA	NA	NA	S259;	2.38E-05	NA	NA
Q68CP9	ARID2	NA	NA	NA	NA	S689;S1300;	1.62E-05	NA	NA
Q70EL1	USP54	NA	NA	NA	NA	T1422;T65;	8.88E-06	NA	NA
Q7RTS9	DYM	NA	NA	NA	NA	Y458;S391;Y395;T405;	1.45E-05	NA	NA
Q8IVL0	NAV3	NA	NA	NA	NA	S1189;S1044;S275;	1.97E-05	NA	NA
Q8N9B5	JMY	NA	NA	NA	NA	S713;	1.72E-05	NA	NA
Q8NB49	ATP11C	NA	NA	NA	NA	S445;	7.00E-06	NA	NA
Q8WUX9	CHMP7	NA	NA	NA	NA	S417;	2.03E-05	NA	NA

Q96BY7	ATG2B	NA	NA	NA	NA	S1008;S1018;S495;T1022;	3.10E-05	NA	NA
Q96LT9	RNPC3	NA	NA	NA	NA	S108;	1.14E-05	NA	NA
Q96MG7	NDNL2	NA	NA	NA	NA	S64;	2.99E-05	NA	NA
Q96N66	MBOAT7	NA	NA	NA	NA	S285;	2.46E-05	NA	NA
Q96S44	TP53RK	NA	NA	NA	NA	T7;T8;	1.37E-05	NA	NA
Q9BQI3	EIF2AK1	NA	NA	NA	NA	S295;	4.98E-06	NA	NA
Q9BYJ9	YTHDF1	NA	NA	NA	NA	S348;S350;	5.78E-05	NA	NA
Q9H1K0	RBSN	NA	NA	NA	NA	S209;S219;	1.67E-05	NA	NA
Q9H6A9	PCNXL3	NA	NA	NA	NA	T177;S178;T291;	6.77E-06	NA	NA
Q9HAZ1	CLK4	NA	NA	NA	NA	S138;S136;	2.65E-05	NA	NA
Q9NQX5	NPDC1	NA	NA	NA	NA	S229;	5.84E-06	NA	NA
Q9NS37	CREBZF	NA	NA	NA	NA	S50;	1.13E-05	NA	NA
Q9NUJ3	TCP11L1	NA	NA	NA	NA	S55;	1.17E-05	NA	NA
Q9NXV6	CDKN2AIP	NA	NA	NA	NA	S204;S201;	3.46E-06	NA	NA
Q9P107	GMIP	NA	NA	NA	NA	S234;S437;S441;	3.62E-05	NA	NA
Q9P2D0	IBTK	NA	NA	NA	NA	S990;S1045;S1021;S1033;	9.76E-05	NA	NA
Q9P2E3	ZNFX1	NA	NA	NA	NA	S686;S1823;S1837;Y1841;	9.95E-06	NA	NA
Q9UKI2	CDC42EP3	NA	NA	NA	NA	S89;S100;	3.15E-05	NA	NA
Q9ULI0	ATAD2B	NA	NA	NA	NA	S16;	3.57E-05	NA	NA
Q9UNN5	FAF1	NA	NA	NA	NA	S582;S270;	6.07E-06	NA	NA
Q9UPU7	TBC1D2B	NA	NA	NA	NA	S957;	4.04E-05	NA	NA
Q9Y485	DMXL1	NA	NA	NA	NA	S918;S436;	2.19E-05	NA	NA
Q9Y6K9	IKBKG	NA	NA	NA	NA	S387;	4.36E-05	NA	NA
Q9Y6V0	PCLO	NA	NA	NA	NA	S661;S663;S4244;	2.21E-04	NA	NA

Table S2: The list for novel phosphsites

Accession ID	Sites	Symbol	GeneName
A2RRP1	S1827	NBAS	neuroblastoma amplified sequence
A2RRP1	T1804	NBAS	neuroblastoma amplified sequence
A6ND36	T658	FAM83G	family with sequence similarity 83, member G
A7E2V4	S1202	ZSWIM8	zinc finger, SWIM-type containing 8
B1AK53	S612	ESPN	espin
B1AK53	S696	ESPN	espin
B1AK53	S700	ESPN	espin
C9JLW8	S25	FAM195B	family with sequence similarity 195, member B
O00257	S293	CBX4	chromobox homolog 4
O00443	S1645	PIK3C2A	phosphatidylinositol-4-phosphate 3-kinase, catalytic subunit type 2 alpha
O00443	S1648	PIK3C2A	phosphatidylinositol-4-phosphate 3-kinase, catalytic subunit type 2 alpha
O00443	T1662	PIK3C2A	phosphatidylinositol-4-phosphate 3-kinase, catalytic subunit type 2 alpha
O00470	S194	MEIS1	Meis homeobox 1
O00515	S420	LAD1	ladinin 1
O00515	T304	LAD1	ladinin 1
O00515	T305	LAD1	ladinin 1
O14640	S581	DVL1	dishevelled segment polarity protein 1
O14640	S582	DVL1	dishevelled segment polarity protein 1
O14686	S3202	KMT2D	lysine (K)-specific methyltransferase 2D
O14686	T2639	KMT2D	lysine (K)-specific methyltransferase 2D
O15021	S1394	MAST4	microtubule associated serine/threonine kinase family member 4
O15021	S1398	MAST4	microtubule associated serine/threonine kinase family member 4
O15021	S1406	MAST4	microtubule associated serine/threonine kinase family member 4
O15021	S1410	MAST4	microtubule associated serine/threonine kinase family member 4
O15021	S1828	MAST4	microtubule associated serine/threonine kinase family member 4
O15021	S2526	MAST4	microtubule associated serine/threonine kinase family member 4
O15021	T2516	MAST4	microtubule associated serine/threonine kinase family member 4
O15021	T2519	MAST4	microtubule associated serine/threonine kinase family member 4
O15027	S1044	SEC16A	SEC16 homolog A, endoplasmic reticulum export factor
O15027	S1050	SEC16A	SEC16 homolog A, endoplasmic reticulum export factor

O15061	S1107	SYNM	synemin, intermediate filament protein
O15061	T1109	SYNM	synemin, intermediate filament protein
O15234	S126	CASC3	cancer susceptibility candidate 3
O15234	T127	CASC3	cancer susceptibility candidate 3
O43318	Y33	MAP3K7	mitogen-activated protein kinase kinase kinase 7
O43491	S806	EPB41L2	erythrocyte membrane protein band 4.1-like 2
O43524	S438	FOXO3	forkhead box O3
O43683	S370	BUB1	BUB1 mitotic checkpoint serine/threonine kinase
O43683	S381	BUB1	BUB1 mitotic checkpoint serine/threonine kinase
O43683	S396	BUB1	BUB1 mitotic checkpoint serine/threonine kinase
O43683	T392	BUB1	BUB1 mitotic checkpoint serine/threonine kinase
O60292	S1501	SIPA1L3	signal-induced proliferation-associated 1 like 3
O60318	T530	MCM3AP	minichromosome maintenance complex component 3 associated protein
O60469	Y482	DSCAM	Down syndrome cell adhesion molecule
O60583	S530	CCNT2	cyclin T2
O60583	S531	CCNT2	cyclin T2
O60583	S532	CCNT2	cyclin T2
O60583	S537	CCNT2	cyclin T2
O60583	S542	CCNT2	cyclin T2
O60884	S400	DNAJA2	DnaJ (Hsp40) homolog, subfamily A, member 2
O60884	S401	DNAJA2	DnaJ (Hsp40) homolog, subfamily A, member 2
O75116	S25	ROCK2	Rho-associated, coiled-coil containing protein kinase 2
O75151	S840	PHF2	PHD finger protein 2
O75179	S1319	ANKRD17	ankyrin repeat domain 17
O75319	S18	DUSP11	dual specificity phosphatase 11 (RNA/RNP complex 1-interacting)
O75376	Y1966	NCOR1	nuclear receptor corepressor 1
O75400	S903	PRPF40A	PRP40 pre-mRNA processing factor 40 homolog A
O75448	T916	MED24	mediator complex subunit 24
O75494	S141	SRSF10	serine/arginine-rich splicing factor 10
O75494	Y142	SRSF10	serine/arginine-rich splicing factor 10
O75528	S296	TADA3	transcriptional adaptor 3
O75530	S29	EED	embryonic ectoderm development
O75691	S2523	UTP20	UTP20, small subunit (SSU) processome component, homolog (yeast)

O75962	S2426	TRIO	trio Rho guanine nucleotide exchange factor
O94885	S704	SASH1	SAM and SH3 domain containing 1
O94885	S706	SASH1	SAM and SH3 domain containing 1
O95071	S2192	UBR5	ubiquitin protein ligase E3 component n-recognin 5
O95071	S286	UBR5	ubiquitin protein ligase E3 component n-recognin 5
O95155	S871	UBE4B	ubiquitination factor E4B
O95155	T866	UBE4B	ubiquitination factor E4B
O95155	Y862	UBE4B	ubiquitination factor E4B
O95218	S318	ZRANB2	zinc finger, RAN-binding domain containing 2
O95232	S304	LUC7L3	LUC7-like 3 pre-mRNA splicing factor
O95232	T303	LUC7L3	LUC7-like 3 pre-mRNA splicing factor
O95359	S2084	TACC2	transforming, acidic coiled-coil containing protein 2
O95359	T2082	TACC2	transforming, acidic coiled-coil containing protein 2
O95361	S34	TRIM16	tripartite motif containing 16
O95490	S1116	ADGRL2	adhesion G protein-coupled receptor L2
O95684	S321	FGFR1OP	FGFR1 oncogene partner
O95747	S159	OXSR1	oxidative stress responsive 1
O95747	S246	OXSR1	oxidative stress responsive 1
O95747	T239	OXSR1	oxidative stress responsive 1
O95747	T249	OXSR1	oxidative stress responsive 1
O96028	S579	WHSC1	Wolf-Hirschhorn syndrome candidate 1
P00519	S1035	ABL1	ABL proto-oncogene 1, non-receptor tyrosine kinase
P00519	S1044	ABL1	ABL proto-oncogene 1, non-receptor tyrosine kinase
P00519	T1036	ABL1	ABL proto-oncogene 1, non-receptor tyrosine kinase
P01583	S89	IL1A	interleukin 1, alpha
P02751	S1527	FN1	fibronectin 1
P02751	S1530	FN1	fibronectin 1
P03956	Y411	MMP1	matrix metallopeptidase 1
P05114	T81	HMGN1	high mobility group nucleosome binding domain 1
P08069	S1364	IGF1R	insulin-like growth factor 1 receptor
P08069	T354	IGF1R	insulin-like growth factor 1 receptor
P08240	S307	SRPR	signal recognition particle receptor (docking protein)
P08240	T308	SRPR	signal recognition particle receptor (docking protein)

P09327	S747	VIL1	villin 1
P0DJ93	T62	SMIM13	small integral membrane protein 13
P10636	S428	MAPT	microtubule-associated protein tau
P10696	T429	ALPPL2	alkaline phosphatase, placental-like 2
P11171	T490	EPB41	erythrocyte membrane protein band 4.1
P11171	T492	EPB41	erythrocyte membrane protein band 4.1
P11274	Y316	BCR	breakpoint cluster region
P11388	T930	TOP2A	topoisomerase (DNA) II alpha
P11388	T932	TOP2A	topoisomerase (DNA) II alpha
P11388	T934	TOP2A	topoisomerase (DNA) II alpha
P11388	Y935	TOP2A	topoisomerase (DNA) II alpha
P15151	S407	PVR	poliovirus receptor
P15336	T320	ATF2	activating transcription factor 2
P15391	S145	CD19	CD19 molecule
P15391	S146	CD19	CD19 molecule
P15391	S148	CD19	CD19 molecule
P15391	S208	CD19	CD19 molecule
P15408	S16	FOSL2	FOS-like antigen 2
P15408	S314	FOSL2	FOS-like antigen 2
P15408	S315	FOSL2	FOS-like antigen 2
P16104	S122	H2AFX	H2A histone family, member X
P16104	S140	H2AFX	H2A histone family, member X
P17676	S237	CEBPB	CCAAT/enhancer binding protein (C/EBP), beta
P18583	S1829	SON	SON DNA binding protein
P18583	S1831	SON	SON DNA binding protein
P18583	S1832	SON	SON DNA binding protein
P18583	S1874	SON	SON DNA binding protein
P18583	S1876	SON	SON DNA binding protein
P18583	S1885	SON	SON DNA binding protein
P18583	S1887	SON	SON DNA binding protein
P18615	S178	NELFE	negative elongation factor complex member E
P19838	S226	NFKB1	nuclear factor of kappa light polypeptide gene enhancer in B-cells 1
P19838	T897	NFKB1	nuclear factor of kappa light polypeptide gene enhancer in B-cells 1

P19838	T898	NFKB1	nuclear factor of kappa light polypeptide gene enhancer in B-cells 1
P20749	T363	BCL3	B-cell CLL/lymphoma 3
P20749	T368	BCL3	B-cell CLL/lymphoma 3
P20749	T381	BCL3	B-cell CLL/lymphoma 3
P22607	T450	FGFR3	fibroblast growth factor receptor 3
P23443	T399	RPS6KB1	ribosomal protein S6 kinase, 70kDa, polypeptide 1
P23497	S111	SP100	SP100 nuclear antigen
P24928	T511	POLR2A	polymerase (RNA) II (DNA directed) polypeptide A, 220kDa
P25054	S559	APC	adenomatous polyposis coli
P26358	S1467	DNMT1	DNA (cytosine-5-)-methyltransferase 1
P26358	S1468	DNMT1	DNA (cytosine-5-)-methyltransferase 1
P26358	S1469	DNMT1	DNA (cytosine-5-)-methyltransferase 1
P27816	T270	MAP4	microtubule-associated protein 4
P27816	T82	MAP4	microtubule-associated protein 4
P28290	S318	SSFA2	sperm specific antigen 2
P28290	S320	SSFA2	sperm specific antigen 2
P28715	S532	ERCC5	excision repair cross-complementation group 5
P30414	S703	NKTR	natural killer cell triggering receptor
P30414	Y702	NKTR	natural killer cell triggering receptor
P31629	S37	HIVEP2	human immunodeficiency virus type I enhancer binding protein 2
P32004	S793	L1CAM	L1 cell adhesion molecule
P32004	T538	L1CAM	L1 cell adhesion molecule
P32004	T803	L1CAM	L1 cell adhesion molecule
P33991	S772	MCM4	minichromosome maintenance complex component 4
P33991	T774	MCM4	minichromosome maintenance complex component 4
P33991	T778	MCM4	minichromosome maintenance complex component 4
P35611	Y407	ADD1	adducin 1 (alpha)
P35658	S457	NUP214	nucleoporin 214kDa
P35658	T1981	NUP214	nucleoporin 214kDa
P35658	T2007	NUP214	nucleoporin 214kDa
P40425	S146	PBX2	pre-B-cell leukemia homeobox 2
P42166	S168	TMPO	thymopoietin
P42166	S66	TMPO	thymopoietin

P42166	S67	TMPO	thymopoietin
P42166	T160	TMPO	thymopoietin
P42166	T74	TMPO	thymopoietin
P42345	T1252	MTOR	mechanistic target of rapamycin (serine/threonine kinase)
P42566	S684	EPS15	epidermal growth factor receptor pathway substrate 15
P42566	T683	EPS15	epidermal growth factor receptor pathway substrate 15
P42858	S621	HTT	huntingtin
P42858	S634	HTT	huntingtin
P43243	S631	MATR3	matrin 3
P45974	T367	USP5	ubiquitin specific peptidase 5 (isopeptidase T)
P45974	T370	USP5	ubiquitin specific peptidase 5 (isopeptidase T)
P46100	S1236	ATRX	alpha thalassemia/mental retardation syndrome X-linked
P46100	T1230	ATRX	alpha thalassemia/mental retardation syndrome X-linked
P46821	S1631	MAP1B	microtubule-associated protein 1B
P46934	S675	NEDD4	neural precursor cell expressed, developmentally down-regulated 4, E3 ubiquitin protein ligase
P46937	T337	YAP1	Yes-associated protein 1
P47712	T659	PLA2G4A	phospholipase A2, group IVA (cytosolic, calcium-dependent)
P48051	S23	KCNJ6	potassium channel, inwardly rectifying subfamily J, member 6
P48634	S1004	PRRC2A	proline-rich coiled-coil 2A
P48634	S1219	PRRC2A	proline-rich coiled-coil 2A
P48634	S1306	PRRC2A	proline-rich coiled-coil 2A
P48634	S1410	PRRC2A	proline-rich coiled-coil 2A
P48634	S1691	PRRC2A	proline-rich coiled-coil 2A
P48634	S342	PRRC2A	proline-rich coiled-coil 2A
P48634	S350	PRRC2A	proline-rich coiled-coil 2A
P48634	S363	PRRC2A	proline-rich coiled-coil 2A
P48634	S380	PRRC2A	proline-rich coiled-coil 2A
P48634	S383	PRRC2A	proline-rich coiled-coil 2A
P48634	S766	PRRC2A	proline-rich coiled-coil 2A
P48634	S932	PRRC2A	proline-rich coiled-coil 2A
P48634	T205	PRRC2A	proline-rich coiled-coil 2A
P48634	T387	PRRC2A	proline-rich coiled-coil 2A
P48634	T610	PRRC2A	proline-rich coiled-coil 2A

P48681	S465	NES	nestin
P49411	T423	TUFM	Tu translation elongation factor, mitochondrial
P49761	S24	CLK3	CDC-like kinase 3
P49792	S2287	RANBP2	RAN binding protein 2
P49792	S2835	RANBP2	RAN binding protein 2
P49792	S2858	RANBP2	RAN binding protein 2
P49815	T659	TSC2	tuberous sclerosis 2
P49815	T667	TSC2	tuberous sclerosis 2
P50402	T122	EMD	emerin
P50479	T115	PDLIM4	PDZ and LIM domain 4
P50479	T124	PDLIM4	PDZ and LIM domain 4
P50851	S1669	LRBA	LPS-responsive vesicle trafficking, beach and anchor containing
P50851	S1919	LRBA	LPS-responsive vesicle trafficking, beach and anchor containing
P51003	S628	PAPOLA	poly(A) polymerase alpha
P51003	S629	PAPOLA	poly(A) polymerase alpha
P51003	S635	PAPOLA	poly(A) polymerase alpha
P51003	T640	PAPOLA	poly(A) polymerase alpha
P51003	T652	PAPOLA	poly(A) polymerase alpha
P51114	S448	FXR1	fragile X mental retardation, autosomal homolog 1
P51114	S450	FXR1	fragile X mental retardation, autosomal homolog 1
P51532	S1617	SMARCA4	SWI/SNF related, matrix associated, actin dependent regulator of chromatin, subfamily a, member 4
P51532	S1620	SMARCA4	SWI/SNF related, matrix associated, actin dependent regulator of chromatin, subfamily a, member 4
P51532	T859	SMARCA4	SWI/SNF related, matrix associated, actin dependent regulator of chromatin, subfamily a, member 4
P51532	Y860	SMARCA4	SWI/SNF related, matrix associated, actin dependent regulator of chromatin, subfamily a, member 4
P51532	Y862	SMARCA4	SWI/SNF related, matrix associated, actin dependent regulator of chromatin, subfamily a, member 4
P51784	Y642	USP11	ubiquitin specific peptidase 11
P51825	S634	AFF1	AF4/FMR2 family, member 1
P52701	S63	MSH6	mutS homolog 6
P52701	Y478	MSH6	mutS homolog 6
P53814	T517	SMTN	smoothelin
P54259	S14	ATN1	atrophin 1
P54259	S689	ATN1	atrophin 1
P54259	T736	ATN1	atrophin 1

P54278	S522	PMS2	PMS1 homolog 2, mismatch repair system component
P54725	S140	RAD23A	RAD23 homolog A, nucleotide excision repair protein
P54725	T131	RAD23A	RAD23 homolog A, nucleotide excision repair protein
P55198	S224	MLLT6	myeloid/lymphoid or mixed-lineage leukemia; translocated to, 6
P55198	T225	MLLT6	myeloid/lymphoid or mixed-lineage leukemia; translocated to, 6
P57682	S80	KLF3	Kruppel-like factor 3 (basic)
P60174	S36	TPI1	triosephosphate isomerase 1
P60174	S41	TPI1	triosephosphate isomerase 1
P62995	S22	TRA2B	transformer 2 beta homolog (Drosophila)
P62995	S73	TRA2B	transformer 2 beta homolog (Drosophila)
P78332	S582	RBM6	RNA binding motif protein 6
P78332	T581	RBM6	RNA binding motif protein 6
P78368	S37	CSNK1G2	casein kinase 1, gamma 2
P78371	S41	CCT2	chaperonin containing TCP1, subunit 2 (beta)
P78371	T27	CCT2	chaperonin containing TCP1, subunit 2 (beta)
P78371	T42	CCT2	chaperonin containing TCP1, subunit 2 (beta)
P78371	T64	CCT2	chaperonin containing TCP1, subunit 2 (beta)
P78371	T69	CCT2	chaperonin containing TCP1, subunit 2 (beta)
P78524	S1011	ST5	suppression of tumorigenicity 5
P78524	S1013	ST5	suppression of tumorigenicity 5
P78524	S1015	ST5	suppression of tumorigenicity 5
P78563	S458	ADARB1	adenosine deaminase, RNA-specific, B1
P78563	S467	ADARB1	adenosine deaminase, RNA-specific, B1
P80723	T222	BASP1	brain abundant, membrane attached signal protein 1
P84098	T194	RPL19	ribosomal protein L19
P85037	S705	FOXK1	forkhead box K1
P85037	S711	FOXK1	forkhead box K1
P98082	S471	DAB2	Dab, mitogen-responsive phosphoprotein, homolog 2 (Drosophila)
Q00341	T567	HDLBP	high density lipoprotein binding protein
Q00587	S142	CDC42EP1	CDC42 effector protein (Rho GTPase binding) 1
Q00839	S764	HNRNPU	heterogeneous nuclear ribonucleoprotein U (scaffold attachment factor A)
Q00839	S766	HNRNPU	heterogeneous nuclear ribonucleoprotein U (scaffold attachment factor A)
Q01130	S119	SRSF2	serine/arginine-rich splicing factor 2

Q01130	S140	SRSF2	serine/arginine-rich splicing factor 2
Q01167	S168	FOXK2	forkhead box K2
Q01780	T675	EXOSC10	exosome component 10
Q01814	S1205	ATP2B2	ATPase, Ca++ transporting, plasma membrane 2
Q02487	T512	DSC2	desmocollin 2
Q02487	T515	DSC2	desmocollin 2
Q02487	T519	DSC2	desmocollin 2
Q03001	S7502	DST	dystonin
Q03001	T5847	DST	dystonin
Q03154	S208	ACY1	aminoacylase 1
Q03154	T201	ACY1	aminoacylase 1
Q03164	S523	KMT2A	lysine (K)-specific methyltransferase 2A
Q05193	S817	DNM1	dynamin 1
Q07065	S299	CKAP4	cytoskeleton-associated protein 4
Q07065	S302	CKAP4	cytoskeleton-associated protein 4
Q07065	T298	CKAP4	cytoskeleton-associated protein 4
Q07157	S171	TJP1	tight junction protein 1
Q08170	S184	SRSF4	serine/arginine-rich splicing factor 4
Q08170	S267	SRSF4	serine/arginine-rich splicing factor 4
Q08170	S269	SRSF4	serine/arginine-rich splicing factor 4
Q08170	S444	SRSF4	serine/arginine-rich splicing factor 4
Q08170	S464	SRSF4	serine/arginine-rich splicing factor 4
Q08170	S466	SRSF4	serine/arginine-rich splicing factor 4
Q08945	T642	SSRP1	structure specific recognition protein 1
Q09666	S1088	AHNAK	AHNAK nucleoprotein
Q0JRZ9	S579	FCHO2	FCH domain only 2
Q0JRZ9	T570	FCHO2	FCH domain only 2
Q0VF96	S196	CGNL1	cingulin-like 1
Q0ZGT2	S665	NEXN	nexilin (F actin binding protein)
Q0ZGT2	S673	NEXN	nexilin (F actin binding protein)
Q0ZGT2	T666	NEXN	nexilin (F actin binding protein)
Q0ZGT2	T670	NEXN	nexilin (F actin binding protein)
Q12766	S1500	HMGXB3	HMG box domain containing 3

Q12766	S226	HMGXB3	HMG box domain containing 3
Q12766	S227	HMGXB3	HMG box domain containing 3
Q12802	S403	AKAP13	A kinase (PRKA) anchor protein 13
Q12872	S872	SFSWAP	splicing factor, suppressor of white-apricot family
Q12873	S1219	CHD3	chromodomain helicase DNA binding protein 3
Q12873	S1221	CHD3	chromodomain helicase DNA binding protein 3
Q12894	S87	IFRD2	interferon-related developmental regulator 2
Q12894	S88	IFRD2	interferon-related developmental regulator 2
Q12955	S1569	ANK3	ankyrin 3, node of Ranvier (ankyrin G)
Q12996	S676	CSTF3	cleavage stimulation factor, 3' pre-RNA, subunit 3, 77kDa
Q13029	S1572	PRDM2	PR domain containing 2, with ZNF domain
Q13029	S164	PRDM2	PR domain containing 2, with ZNF domain
Q13029	S165	PRDM2	PR domain containing 2, with ZNF domain
Q13029	S168	PRDM2	PR domain containing 2, with ZNF domain
Q13099	S161	IFT88	intraflagellar transport 88
Q13099	S166	IFT88	intraflagellar transport 88
Q13099	T321	IFT88	intraflagellar transport 88
Q13099	Y318	IFT88	intraflagellar transport 88
Q13099	Y324	IFT88	intraflagellar transport 88
Q13243	S208	SRSF5	serine/arginine-rich splicing factor 5
Q13243	S211	SRSF5	serine/arginine-rich splicing factor 5
Q13243	Y212	SRSF5	serine/arginine-rich splicing factor 5
Q13247	S212	SRSF6	serine/arginine-rich splicing factor 6
Q13247	S214	SRSF6	serine/arginine-rich splicing factor 6
Q13247	S272	SRSF6	serine/arginine-rich splicing factor 6
Q13427	S585	PPIG	peptidylprolyl isomerase G (cyclophilin G)
Q13427	S587	PPIG	peptidylprolyl isomerase G (cyclophilin G)
Q13427	S616	PPIG	peptidylprolyl isomerase G (cyclophilin G)
Q13459	S2002	MYO9B	myosin IXB
Q13459	T2001	MYO9B	myosin IXB
Q13459	T460	MYO9B	myosin IXB
Q13470	T91	TNK1	tyrosine kinase, non-receptor, 1
Q13523	S232	PRPF4B	pre-mRNA processing factor 4B

Q13523	S376	PRPF4B	pre-mRNA processing factor 4B
Q13523	S379	PRPF4B	pre-mRNA processing factor 4B
Q13523	S451	PRPF4B	pre-mRNA processing factor 4B
Q13523	S453	PRPF4B	pre-mRNA processing factor 4B
Q13523	T615	PRPF4B	pre-mRNA processing factor 4B
Q13765	S166	NACA	nascent polypeptide-associated complex alpha subunit
Q14004	S206	CDK13	cyclin-dependent kinase 13
Q14106	S75	TOB2	transducer of ERBB2, 2
Q14135	T151	VGLL4	vestigial-like family member 4
Q14139	S50	UBE4A	ubiquitination factor E4A
Q14139	S53	UBE4A	ubiquitination factor E4A
Q14139	S57	UBE4A	ubiquitination factor E4A
Q14155	S760	ARHGEF7	Rho guanine nucleotide exchange factor (GEF) 7
Q14315	S2602	FLNC	filamin C, gamma
Q14315	T2606	FLNC	filamin C, gamma
Q14432	S1030	PDE3A	phosphodiesterase 3A, cGMP-inhibited
Q14432	S1033	PDE3A	phosphodiesterase 3A, cGMP-inhibited
Q14432	T1036	PDE3A	phosphodiesterase 3A, cGMP-inhibited
Q14444	S24	CAPRIN1	cell cycle associated protein 1
Q14498	S40	RBM39	RNA binding motif protein 39
Q14498	S42	RBM39	RNA binding motif protein 39
Q14498	S44	RBM39	RNA binding motif protein 39
Q14669	Y1015	TRIP12	thyroid hormone receptor interactor 12
Q14694	Y791	USP10	ubiquitin specific peptidase 10
Q14694	Y792	USP10	ubiquitin specific peptidase 10
Q14696	S220	MESDC2	mesoderm development candidate 2
Q14696	S221	MESDC2	mesoderm development candidate 2
Q14739	S73	LBR	lamin B receptor
Q14739	T68	LBR	lamin B receptor
Q14746	S104	COG2	component of oligomeric golgi complex 2
Q14966	Y514	ZNF638	zinc finger protein 638
Q14966	Y516	ZNF638	zinc finger protein 638
Q14C86	S772	GAPVD1	GTPase activating protein and VPS9 domains 1

Q14C86	T938	GAPVD1	GTPase activating protein and VPS9 domains 1
Q15014	S8	MORF4L2	mortality factor 4 like 2
Q15056	S66	EIF4H	eukaryotic translation initiation factor 4H
Q15149	T723	PLEC	plectin
Q15154	Y535	PCM1	pericentriolar material 1
Q15276	S419	RABEP1	rabaptin, RAB GTPase binding effector protein 1
Q15287	S137	RNPS1	RNA binding protein S1, serine-rich domain
Q15287	S139	RNPS1	RNA binding protein S1, serine-rich domain
Q15287	S274	RNPS1	RNA binding protein S1, serine-rich domain
Q15287	S276	RNPS1	RNA binding protein S1, serine-rich domain
Q15311	S647	RALBP1	ralA binding protein 1
Q15386	S379	UBE3C	ubiquitin protein ligase E3C
Q15386	S380	UBE3C	ubiquitin protein ligase E3C
Q15413	S3391	RYR3	ryanodine receptor 3
Q15413	S3395	RYR3	ryanodine receptor 3
Q15424	S325	SAFB	scaffold attachment factor B
Q15648	T391	MED1	mediator complex subunit 1
Q15751	T227	HERC1	HECT and RLD domain containing E3 ubiquitin protein ligase family member 1
Q15910	S84	EZH2	enhancer of zeste 2 polycomb repressive complex 2 subunit
Q16629	S130	SRSF7	serine/arginine-rich splicing factor 7
Q16629	S132	SRSF7	serine/arginine-rich splicing factor 7
Q16629	S134	SRSF7	serine/arginine-rich splicing factor 7
Q2KHR3	S1227	QSER1	glutamine and serine rich 1
Q2KHT3	S864	CLEC16A	C-type lectin domain family 16, member A
Q2NKX8	S946	ERCC6L	excision repair cross-complementation group 6-like
Q2TAC6	T549	KIF19	kinesin family member 19
Q32MZ4	S639	LRRFIP1	leucine rich repeat (in FLII) interacting protein 1
Q4ADV7	S995	RIC1	RIC1 homolog, RAB6A GEF complex partner 1
Q4KMP7	S316	TBC1D10B	TBC1 domain family, member 10B
Q4KMP7	S322	TBC1D10B	TBC1 domain family, member 10B
Q53F19	S225	NCBP3	nuclear cap binding subunit 3
Q53GS7	S367	GLE1	GLE1 RNA export mediator
Q53H80	S31	AKIRIN2	akirin 2

Q5C9Z4	T327	NOM1	nucleolar protein with MIF4G domain 1
Q5H9R7	T517	PPP6R3	protein phosphatase 6, regulatory subunit 3
Q5JRA6	S1553	MIA3	melanoma inhibitory activity family, member 3
Q5JRA6	S1561	MIA3	melanoma inhibitory activity family, member 3
Q5M775	S356	SPECC1	sperm antigen with calponin homology and coiled-coil domains 1
Q5M775	T357	SPECC1	sperm antigen with calponin homology and coiled-coil domains 1
Q5NUL3	S366	FFAR4	free fatty acid receptor 4
Q5NUL3	T363	FFAR4	free fatty acid receptor 4
Q5NUL3	T365	FFAR4	free fatty acid receptor 4
Q5SW79	T945	CEP170	centrosomal protein 170kDa
Q5SW79	T948	CEP170	centrosomal protein 170kDa
Q5SXM2	S1407	SNAPC4	small nuclear RNA activating complex, polypeptide 4, 190kDa
Q5SXM2	T625	SNAPC4	small nuclear RNA activating complex, polypeptide 4, 190kDa
Q5SXM8	S171	DNLZ	DNL-type zinc finger
Q5SYE7	S720	NHSL1	NHS-like 1
Q5SYE7	S723	NHSL1	NHS-like 1
Q5SYE7	S851	NHSL1	NHS-like 1
Q5SYE7	S853	NHSL1	NHS-like 1
Q5SYE7	S857	NHSL1	NHS-like 1
Q5SYE7	T862	NHSL1	NHS-like 1
Q5SYE7	Y856	NHSL1	NHS-like 1
Q5T200	S1278	ZC3H13	zinc finger CCCH-type containing 13
Q5T200	Y851	ZC3H13	zinc finger CCCH-type containing 13
Q5T3F8	S113	TMEM63B	transmembrane protein 63B
Q5T4S7	S364	UBR4	ubiquitin protein ligase E3 component n-recognin 4
Q5T4S7	S457	UBR4	ubiquitin protein ligase E3 component n-recognin 4
Q5T6F2	T476	UBAP2	ubiquitin associated protein 2
Q5T8P6	S184	RBM26	RNA binding motif protein 26
Q5T8P6	S186	RBM26	RNA binding motif protein 26
Q5T8P6	S188	RBM26	RNA binding motif protein 26
Q5T8P6	Y179	RBM26	RNA binding motif protein 26
Q5TCZ1	S318	SH3PXD2A	SH3 and PX domains 2A
Q5TG92	S24	C1orf195	chromosome 1 open reading frame 195

Q5TG92	S29	C1orf195	chromosome 1 open reading frame 195
Q5TG92	T25	C1orf195	chromosome 1 open reading frame 195
Q5TGP6	S135	MROH9	maestro heat-like repeat family member 9
Q5TGP6	S136	MROH9	maestro heat-like repeat family member 9
Q5TGP6	S357	MROH9	maestro heat-like repeat family member 9
Q5TGP6	Y137	MROH9	maestro heat-like repeat family member 9
Q5UIP0	S2231	RIF1	replication timing regulatory factor 1
Q5VT52	S599	RPRD2	regulation of nuclear pre-mRNA domain containing 2
Q5VT52	S925	RPRD2	regulation of nuclear pre-mRNA domain containing 2
Q5VTB9	T401	RNF220	ring finger protein 220
Q5VTR2	S652	RNF20	ring finger protein 20, E3 ubiquitin protein ligase
Q5VTR2	T31	RNF20	ring finger protein 20, E3 ubiquitin protein ligase
Q5VV41	S576	ARHGEF16	Rho guanine nucleotide exchange factor (GEF) 16
Q5VV41	T226	ARHGEF16	Rho guanine nucleotide exchange factor (GEF) 16
Q5VVP1	S556	SPATA31A6	SPATA31 subfamily A, member 6
Q5VWQ8	S33	DAB2IP	DAB2 interacting protein
Q5VWQ8	S35	DAB2IP	DAB2 interacting protein
Q5VWQ8	T37	DAB2IP	DAB2 interacting protein
Q5VYS8	S777	ZCCHC6	zinc finger, CCHC domain containing 6
Q5VYS8	T778	ZCCHC6	zinc finger, CCHC domain containing 6
Q5VYS8	T796	ZCCHC6	zinc finger, CCHC domain containing 6
Q5VZK9	S1148	LRRC16A	leucine rich repeat containing 16A
Q641Q2	S1091	FAM21A	family with sequence similarity 21, member A
Q641Q2	S56	FAM21A	family with sequence similarity 21, member A
Q659A1	S570	ICE2	interactor of little elongation complex ELL subunit 2
Q659A1	S571	ICE2	interactor of little elongation complex ELL subunit 2
Q659A1	T573	ICE2	interactor of little elongation complex ELL subunit 2
Q659C4	S869	LARP1B	La ribonucleoprotein domain family, member 1B
Q68DQ2	S1043	CRYBG3	beta-gamma crystallin domain containing 3
Q6DD88	S149	ATL3	atlastin GTPase 3
Q6IQ23	S463	PLEKHA7	pleckstrin homology domain containing, family A member 7
Q6IQ49	T314	SDE2	SDE2 telomere maintenance homolog (S. pombe)
Q6IQ49	T316	SDE2	SDE2 telomere maintenance homolog (S. pombe)

Q6KC79	S850	NIPBL	Nipped-B homolog (Drosophila)
Q6NT89	S78	TRNP1	TMF1-regulated nuclear protein 1
Q6NT89	S90	TRNP1	TMF1-regulated nuclear protein 1
Q6NYC8	S139	PPP1R18	protein phosphatase 1, regulatory subunit 18
Q6P158	S36	DHX57	DEAH (Asp-Glu-Ala-Asp/His) box polypeptide 57
Q6P1L5	S116	FAM117B	family with sequence similarity 117, member B
Q6P1L5	S417	FAM117B	family with sequence similarity 117, member B
Q6P1L5	T162	FAM117B	family with sequence similarity 117, member B
Q6P1M3	T284	LLGL2	lethal giant larvae homolog 2 (Drosophila)
Q6P1M3	Y275	LLGL2	lethal giant larvae homolog 2 (Drosophila)
Q6P2E9	S705	EDC4	enhancer of mRNA decapping 4
Q6P2E9	S890	EDC4	enhancer of mRNA decapping 4
Q6P4R8	S347	NFRKB	nuclear factor related to kappaB binding protein
Q6P6C2	Y367	ALKBH5	alkB homolog 5, RNA demethylase
Q6PJG2	S648	ELMSAN1	ELM2 and Myb/SANT-like domain containing 1
Q6PJT7	S135	ZC3H14	zinc finger CCCH-type containing 14
Q6T4R5	S855	NHS	Nance-Horan syndrome (congenital cataracts and dental anomalies)
Q6UN15	T68	FIP1L1	factor interacting with PAPOLA and CPSF1
Q6UX04	S299	CWC27	CWC27 spliceosome-associated protein homolog
Q6VMQ6	S862	ATF7IP	activating transcription factor 7 interacting protein
Q6VMQ6	T860	ATF7IP	activating transcription factor 7 interacting protein
Q6Y7W6	S19	GIGYF2	GRB10 interacting GYF protein 2
Q6Y7W6	S201	GIGYF2	GRB10 interacting GYF protein 2
Q6Y7W6	S23	GIGYF2	GRB10 interacting GYF protein 2
Q6Y7W6	S26	GIGYF2	GRB10 interacting GYF protein 2
Q6Y7W6	S30	GIGYF2	GRB10 interacting GYF protein 2
Q6Y7W6	S392	GIGYF2	GRB10 interacting GYF protein 2
Q6Y7W6	T25	GIGYF2	GRB10 interacting GYF protein 2
Q6ZNJ1	T1879	NBEAL2	neurobeachin-like 2
Q6ZNJ1	T651	NBEAL2	neurobeachin-like 2
Q6ZNJ1	T658	NBEAL2	neurobeachin-like 2
Q6ZRS2	S808	SRCAP	Snf2-related CREBBP activator protein
Q6ZRS2	T812	SRCAP	Snf2-related CREBBP activator protein

Q6ZRS2	Y821	SRCAP	Snf2-related CREBBP activator protein
Q6ZS17	T728	FAM65A	family with sequence similarity 65, member A
Q6ZUM4	S633	ARHGAP27	Rho GTPase activating protein 27
Q70CQ2	T3390	USP34	ubiquitin specific peptidase 34
Q70EL1	T1422	USP54	ubiquitin specific peptidase 54
Q70EL1	T65	USP54	ubiquitin specific peptidase 54
Q75WM6	S236	H1FNT	H1 histone family, member N, testis-specific
Q75WM6	S237	H1FNT	H1 histone family, member N, testis-specific
Q76FK4	S662	NOL8	nucleolar protein 8
Q7L014	S24	DDX46	DEAD (Asp-Glu-Ala-Asp) box polypeptide 46
Q7L0X2	S189	ERICH6	glutamate-rich 6
Q7L2J0	T245	MEPCE	methylphosphate capping enzyme
Q7LC44	S143	ARC	activity-regulated cytoskeleton-associated protein
Q7LC44	Y137	ARC	activity-regulated cytoskeleton-associated protein
Q7RTP6	S1173	MICAL3	microtubule associated monooxygenase, calponin and LIM domain containing 3
Q7RTS9	S391	DYM	dymeclin
Q7RTS9	T405	DYM	dymeclin
Q7RTS9	Y395	DYM	dymeclin
Q7Z2W4	S636	ZC3HAV1	zinc finger CCCH-type, antiviral 1
Q7Z2W4	Y637	ZC3HAV1	zinc finger CCCH-type, antiviral 1
Q7Z309	S149	FAM122B	family with sequence similarity 122B
Q7Z333	S687	SETX	senataxin
Q7Z333	S692	SETX	senataxin
Q7Z333	S693	SETX	senataxin
Q7Z3C6	Y762	ATG9A	autophagy related 9A
Q7Z3K3	T851	POGZ	pogo transposable element with ZNF domain
Q7Z417	S306	NUFIP2	nuclear fragile X mental retardation protein interacting protein 2
Q7Z417	S379	NUFIP2	nuclear fragile X mental retardation protein interacting protein 2
Q7Z4S6	S1309	KIF21A	kinesin family member 21A
Q7Z4V5	S418	HDGFRP2	hepatoma-derived growth factor-related protein 2
Q7Z6E9	S1273	RBBP6	retinoblastoma binding protein 6
Q7Z6E9	S1535	RBBP6	retinoblastoma binding protein 6
Q7Z6E9	S1715	RBBP6	retinoblastoma binding protein 6

Q7Z6E9	S716	RBBP6	retinoblastoma binding protein 6
Q7Z6E9	Y715	RBBP6	retinoblastoma binding protein 6
Q7Z6Z7	S3936	HUWE1	HECT, UBA and WWE domain containing 1, E3 ubiquitin protein ligase
Q86TC9	T820	MYPN	myopalladin
Q86TN4	S244	TRPT1	tRNA phosphotransferase 1
Q86TN4	S245	TRPT1	tRNA phosphotransferase 1
Q86U86	Y462	PBRM1	polybromo 1
Q86U86	Y470	PBRM1	polybromo 1
Q86V48	T947	LUZP1	leucine zipper protein 1
Q86VM9	T93	ZC3H18	zinc finger CCCH-type containing 18
Q86VQ1	S138	GLCCI1	glucocorticoid induced 1
Q86VQ1	S139	GLCCI1	glucocorticoid induced 1
Q86VQ1	S394	GLCCI1	glucocorticoid induced 1
Q86VQ1	S397	GLCCI1	glucocorticoid induced 1
Q86X95	S305	CIR1	corepressor interacting with RBPJ, 1
Q86YZ3	S540	HRNR	hornerin
Q8IV63	S75	VRK3	vaccinia related kinase 3
Q8IV63	S78	VRK3	vaccinia related kinase 3
Q8IWS0	S4	PHF6	PHD finger protein 6
Q8IWS0	T12	PHF6	PHD finger protein 6
Q8IWU2	S1310	LMTK2	lemur tyrosine kinase 2
Q8IWX8	S802	CHERP	calcium homeostasis endoplasmic reticulum protein
Q8IWZ3	T86	ANKHD1-EIF4EBP3	ANKHD1-EIF4EBP3 readthrough
Q8IY57	T158	YAF2	YY1 associated factor 2
Q8IY67	S17	RAVER1	ribonucleoprotein, PTB-binding 1
Q8IY67	T463	RAVER1	ribonucleoprotein, PTB-binding 1
Q8IY81	S468	FTSJ3	FtsJ homolog 3 (E. coli)
Q8IY81	S471	FTSJ3	FtsJ homolog 3 (E. coli)
Q8IYB3	S162	SRRM1	serine/arginine repetitive matrix 1
Q8IYB3	S379	SRRM1	serine/arginine repetitive matrix 1
Q8IYB3	T378	SRRM1	serine/arginine repetitive matrix 1
Q8IYW2	T2217	CFAP46	cilia and flagella associated protein 46
Q8N1F7	S769	NUP93	nucleoporin 93kDa

Q8N1G0	S239	ZNF687	zinc finger protein 687
Q8N292	S82	GAPT	GRB2-binding adaptor protein, transmembrane
Q8N292	T89	GAPT	GRB2-binding adaptor protein, transmembrane
Q8N2M8	S315	CLASRP	CLK4-associating serine/arginine rich protein
Q8N2M8	Y453	CLASRP	CLK4-associating serine/arginine rich protein
Q8N2S1	S1169	LTBP4	latent transforming growth factor beta binding protein 4
Q8N3D4	T891	EHBP1L1	EH domain binding protein 1-like 1
Q8N5A5	T271	ZGPAT	zinc finger, CCCH-type with G patch domain
Q8N5F7	S25	NKAP	NFKB activating protein
Q8N5F7	S27	NKAP	NFKB activating protein
Q8N5F7	S36	NKAP	NFKB activating protein
Q8N5F7	S48	NKAP	NFKB activating protein
Q8N5F7	S85	NKAP	NFKB activating protein
Q8N8S7	S541	ENAH	enabled homolog (Drosophila)
Q8N9T8	Y130	KRI1	KRI1 homolog
Q8NAF0	T482	ZNF579	zinc finger protein 579
Q8NBB4	S247	ZSCAN1	zinc finger and SCAN domain containing 1
Q8NC51	S74	SERBP1	SERPINE1 mRNA binding protein 1
Q8NC51	T226	SERBP1	SERPINE1 mRNA binding protein 1
Q8NCD3	S210	HJURP	Holliday junction recognition protein
Q8NCN4	S371	RNF169	ring finger protein 169
Q8NCN4	S374	RNF169	ring finger protein 169
Q8NDI1	S295	EHBP1	EH domain binding protein 1
Q8NEG2	S242	C7orf57	chromosome 7 open reading frame 57
Q8NEG2	S245	C7orf57	chromosome 7 open reading frame 57
Q8NEL9	S731	DDHD1	DDHD domain containing 1
Q8NEN9	T982	PDZD8	PDZ domain containing 8
Q8NEY8	S143	PPHLN1	periphilin 1
Q8NF91	T2001	SYNE1	spectrin repeat containing, nuclear envelope 1
Q8NF91	T3803	SYNE1	spectrin repeat containing, nuclear envelope 1
Q8NFG4	S161	FLCN	folliculin
Q8NI08	T360	NCOA7	nuclear receptor coactivator 7
Q8TAX0	S157	OSR1	odd-skipped related transciption factor 1

Q8TAX0	T154	OSR1	odd-skipped related transciption factor 1
Q8TBF4	T68	ZCRB1	zinc finger CCHC-type and RNA binding motif 1
Q8TC20	S190	CAGE1	cancer antigen 1
Q8TC20	S197	CAGE1	cancer antigen 1
Q8TC20	T204	CAGE1	cancer antigen 1
Q8TCU6	S1559	PREX1	phosphatidylinositol-3,4,5-trisphosphate-dependent Rac exchange factor 1
Q8TCU6	S512	PREX1	phosphatidylinositol-3,4,5-trisphosphate-dependent Rac exchange factor 1
Q8TCU6	T1585	PREX1	phosphatidylinositol-3,4,5-trisphosphate-dependent Rac exchange factor 1
Q8TCU6	T500	PREX1	phosphatidylinositol-3,4,5-trisphosphate-dependent Rac exchange factor 1
Q8TCU6	Y496	PREX1	phosphatidylinositol-3,4,5-trisphosphate-dependent Rac exchange factor 1
Q8TDC3	S322	BRSK1	BR serine/threonine kinase 1
Q8TDC3	S325	BRSK1	BR serine/threonine kinase 1
Q8TE67	S441	EPS8L3	EPS8-like 3
Q8TE67	S444	EPS8L3	EPS8-like 3
Q8TE67	S445	EPS8L3	EPS8-like 3
Q8TE67	S518	EPS8L3	EPS8-like 3
Q8TE67	S520	EPS8L3	EPS8-like 3
Q8TE67	T511	EPS8L3	EPS8-like 3
Q8TF01	Y576	PNISR	PNN-interacting serine/arginine-rich protein
Q8TF72	S1279	SHROOM3	shroom family member 3
Q8WUU4	S8	ZNF296	zinc finger protein 296
Q8WVC0	S637	LEO1	LEO1 homolog, Paf1/RNA polymerase II complex component
Q8WVK2	S6	SNRNP27	small nuclear ribonucleoprotein 27kDa (U4/U6.U5)
Q8WVK2	S8	SNRNP27	small nuclear ribonucleoprotein 27kDa (U4/U6.U5)
Q8WXA9	S251	SREK1	splicing regulatory glutamine/lysine-rich protein 1
Q8WXA9	S253	SREK1	splicing regulatory glutamine/lysine-rich protein 1
Q8WXA9	S255	SREK1	splicing regulatory glutamine/lysine-rich protein 1
Q8WXA9	S361	SREK1	splicing regulatory glutamine/lysine-rich protein 1
Q8WXA9	S386	SREK1	splicing regulatory glutamine/lysine-rich protein 1
Q8WXA9	S390	SREK1	splicing regulatory glutamine/lysine-rich protein 1
Q8WXA9	S392	SREK1	splicing regulatory glutamine/lysine-rich protein 1
Q8WXG6	S1160	MADD	MAP-kinase activating death domain
Q8WY36	T722	BBX	bobby sox homolog (Drosophila)

Q8WZ42	S14046	TTN	titin
Q8WZ42	S16647	TTN	titin
Q8WZ42	S16649	TTN	titin
Q8WZ42	T14042	TTN	titin
Q8WZ42	T6046	TTN	titin
Q8WZ42	Y6045	TTN	titin
Q92504	T294	SLC39A7	solute carrier family 39 (zinc transporter), member 7
Q92538	S128	GBF1	golgi brefeldin A resistant guanine nucleotide exchange factor 1
Q92576	S122	PHF3	PHD finger protein 3
Q92614	S2010	MYO18A	myosin XVIIIA
Q92615	Y501	LARP4B	La ribonucleoprotein domain family, member 4B
Q92733	T215	PRCC	papillary renal cell carcinoma (translocation-associated)
Q92766	T1121	RREB1	ras responsive element binding protein 1
Q92804	S228	TAF15	TAF15 RNA polymerase II, TATA box binding protein (TBP)-associated factor, 68kDa
Q92804	T235	TAF15	TAF15 RNA polymerase II, TATA box binding protein (TBP)-associated factor, 68kDa
Q92817	S973	EVPL	envoplakin
Q92841	S125	DDX17	DEAD (Asp-Glu-Ala-Asp) box helicase 17
Q92994	S357	BRF1	BRF1, RNA polymerase III transcription initiation factor 90 kDa subunit
Q96AP0	T431	ACD	adrenocortical dysplasia homolog (mouse)
Q96AY2	S17	EME1	essential meiotic structure-specific endonuclease 1
Q96AY2	S6	EME1	essential meiotic structure-specific endonuclease 1
Q96AY2	S9	EME1	essential meiotic structure-specific endonuclease 1
Q96D71	S428	REPS1	RALBP1 associated Eps domain containing 1
Q96D71	T520	REPS1	RALBP1 associated Eps domain containing 1
Q96E09	S286	FAM122A	family with sequence similarity 122A
Q96EV2	S739	RBM33	RNA binding motif protein 33
Q96EV2	T853	RBM33	RNA binding motif protein 33
Q96EY5	S18	MVB12A	multivesicular body subunit 12A
Q96EY5	S19	MVB12A	multivesicular body subunit 12A
Q96EY5	S21	MVB12A	multivesicular body subunit 12A
Q96GA3	Y204	LTV1	LTV1 ribosome biogenesis factor
Q96JY6	T312	PDLIM2	PDZ and LIM domain 2 (mystique)
Q96JY6	Y305	PDLIM2	PDZ and LIM domain 2 (mystique)

Q96N20	S287	ZNF75A	zinc finger protein 75a
Q96N64	S521	PWWP2A	PWWP domain containing 2A
Q96PE1	T1329	ADGRA2	adhesion G protein-coupled receptor A2
Q96PE1	Y1311	ADGRA2	adhesion G protein-coupled receptor A2
Q96RK0	T435	CIC	capicua transcriptional repressor
Q96SB4	S39	SRPK1	SRSF protein kinase 1
Q96T58	S250	SPEN	spen family transcriptional repressor
Q96T58	T1643	SPEN	spen family transcriptional repressor
Q96T58	T2374	SPEN	spen family transcriptional repressor
Q99081	S332	TCF12	transcription factor 12
Q99549	T440	MPHOSPH8	M-phase phosphoprotein 8
Q99569	S227	PKP4	plakophilin 4
Q99569	S293	PKP4	plakophilin 4
Q99590	S472	SCAF11	SR-related CTD-associated factor 11
Q99590	S475	SCAF11	SR-related CTD-associated factor 11
Q99590	S830	SCAF11	SR-related CTD-associated factor 11
Q99590	S832	SCAF11	SR-related CTD-associated factor 11
Q99590	S834	SCAF11	SR-related CTD-associated factor 11
Q99590	S839	SCAF11	SR-related CTD-associated factor 11
Q99590	S941	SCAF11	SR-related CTD-associated factor 11
Q99590	S943	SCAF11	SR-related CTD-associated factor 11
Q99618	S151	CDCA3	cell division cycle associated 3
Q99624	T466	SLC38A3	solute carrier family 38, member 3
Q99638	S270	RAD9A	RAD9 checkpoint clamp component A
Q99933	T123	BAG1	BCL2-associated athanogene
Q99956	S356	DUSP9	dual specificity phosphatase 9
Q99956	S364	DUSP9	dual specificity phosphatase 9
Q99956	S368	DUSP9	dual specificity phosphatase 9
Q9BQ61	S49	C19orf43	chromosome 19 open reading frame 43
Q9BQA9	S173	C17orf62	chromosome 17 open reading frame 62
Q9BTC0	S834	DIDO1	death inducer-obliterator 1
Q9BTC0	S835	DIDO1	death inducer-obliterator 1
Q9BTC0	T1733	DIDO1	death inducer-obliterator 1

Q9BU76	S178	C1orf35	chromosome 1 open reading frame 35
Q9BU76	T175	C1orf35	chromosome 1 open reading frame 35
Q9BUQ8	S39	DDX23	DEAD (Asp-Glu-Ala-Asp) box polypeptide 23
Q9BUT9	T89	FAM195A	family with sequence similarity 195, member A
Q9BVS4	T295	RIOK2	RIO kinase 2
Q9BVS4	T328	RIOK2	RIO kinase 2
Q9BW85	Y313	CCDC94	coiled-coil domain containing 94
Q9BXB4	T24	OSBPL11	oxysterol binding protein-like 11
Q9BYJ9	S348	YTHDF1	YTH N(6)-methyladenosine RNA binding protein 1
Q9C0C2	S1031	TNKS1BP1	tankyrase 1 binding protein 1, 182kDa
Q9C0C2	S1046	TNKS1BP1	tankyrase 1 binding protein 1, 182kDa
Q9C0C9	S475	UBE2O	ubiquitin-conjugating enzyme E2O
Q9C0C9	T834	UBE2O	ubiquitin-conjugating enzyme E2O
Q9C0H6	S414	KLHL4	kelch-like family member 4
Q9C0H6	S426	KLHL4	kelch-like family member 4
Q9C0H6	T153	KLHL4	kelch-like family member 4
Q9C0H6	T427	KLHL4	kelch-like family member 4
Q9GZP8	S62	C19orf33	chromosome 19 open reading frame 33
Q9H0L4	S560	CSTF2T	cleavage stimulation factor, 3' pre-RNA, subunit 2, 64kDa, tau variant
Q9H1B7	S336	IRF2BPL	interferon regulatory factor 2 binding protein-like
Q9H3S7	T587	PTPN23	protein tyrosine phosphatase, non-receptor type 23
Q9H4H8	T459	FAM83D	family with sequence similarity 83, member D
Q9H6A9	S178	PCNXL3	pecanex-like 3 (Drosophila)
Q9H6A9	T177	PCNXL3	pecanex-like 3 (Drosophila)
Q9H6E5	T642	TUT1	terminal uridylyl transferase 1, U6 snRNA-specific
Q9H792	T582	PEAK1	pseudopodium-enriched atypical kinase 1
Q9H799	S2779	C5orf42	chromosome 5 open reading frame 42
Q9H7N4	S1005	SCAF1	SR-related CTD-associated factor 1
Q9H7N4	T1012	SCAF1	SR-related CTD-associated factor 1
Q9H8M2	T448	BRD9	bromodomain containing 9
Q9H9J4	Y607	USP42	ubiquitin specific peptidase 42
Q9HA82	S350	CERS4	ceramide synthase 4
Q9HAW4	S1005	CLSPN	claspin

Q9HB19	S202	PLEKHA2	pleckstrin homology domain containing, family A (phosphoinositide binding specific) member 2
Q9HB19	Y204	PLEKHA2	pleckstrin homology domain containing, family A (phosphoinositide binding specific) member 2
Q9HCD6	T1315	TANC2	tetratricopeptide repeat, ankyrin repeat and coiled-coil containing 2
Q9HCG8	S102	CWC22	CWC22 spliceosome-associated protein
Q9HCG8	Y33	CWC22	CWC22 spliceosome-associated protein
Q9NP71	S361	MLXIPL	MLX interacting protein-like
Q9NQ29	S304	LUC7L	LUC7-like
Q9NQ29	S306	LUC7L	LUC7-like
Q9NQ55	T280	PPAN-P2RY11	PPAN-P2RY11 readthrough
Q9NQC3	S361	RTN4	reticulon 4
Q9NQC3	S362	RTN4	reticulon 4
Q9NQC3	S664	RTN4	reticulon 4
Q9NQC3	S666	RTN4	reticulon 4
Q9NR09	S490	BIRC6	baculoviral IAP repeat containing 6
Q9NR48	T561	ASH1L	ash1 (absent, small, or homeotic)-like (Drosophila)
Q9NTZ6	S413	RBM12	RNA binding motif protein 12
Q9NUQ3	S510	TXLNG	taxilin gamma
Q9NW97	T104	TMEM51	transmembrane protein 51
Q9NWM3	S8	CUEDC1	CUE domain containing 1
Q9NXD2	Y224	MTMR10	myotubularin related protein 10
Q9NXV6	S201	CDKN2AIP	CDKN2A interacting protein
Q9NXV6	S204	CDKN2AIP	CDKN2A interacting protein
Q9NYF8	S17	BCLAF1	BCL2-associated transcription factor 1
Q9NYF8	S19	BCLAF1	BCL2-associated transcription factor 1
Q9NYF8	S20	BCLAF1	BCL2-associated transcription factor 1
Q9NYF8	S23	BCLAF1	BCL2-associated transcription factor 1
Q9NYF8	S25	BCLAF1	BCL2-associated transcription factor 1
Q9NYF8	S27	BCLAF1	BCL2-associated transcription factor 1
Q9NYF8	S717	BCLAF1	BCL2-associated transcription factor 1
Q9NYV4	T20	CDK12	cyclin-dependent kinase 12
Q9NYZ3	S547	GTSE1	G-2 and S-phase expressed 1
Q9NZ56	S450	FMN2	formin 2
Q9NZ56	S452	FMN2	formin 2

Q9NZD8	T252	SPG21	spastic paraplegia 21 (autosomal recessive, Mast syndrome)
Q9NZD8	Y258	SPG21	spastic paraplegia 21 (autosomal recessive, Mast syndrome)
Q9NZN5	S1068	ARHGEF12	Rho guanine nucleotide exchange factor (GEF) 12
Q9NZN5	S1077	ARHGEF12	Rho guanine nucleotide exchange factor (GEF) 12
Q9NZT2	S468	OGFR	opioid growth factor receptor
Q9NZT2	S473	OGFR	opioid growth factor receptor
Q9P0P8	S106	C6orf203	chromosome 6 open reading frame 203
Q9P0P8	S116	C6orf203	chromosome 6 open reading frame 203
Q9P1Y5	S350	CAMSAP3	calmodulin regulated spectrin-associated protein family, member 3
Q9P1Y6	S1114	PHRF1	PHD and ring finger domains 1
Q9P1Y6	S1116	PHRF1	PHD and ring finger domains 1
Q9P1Y6	S1167	PHRF1	PHD and ring finger domains 1
Q9P1Y6	S589	PHRF1	PHD and ring finger domains 1
Q9P206	S620	KIAA1522	KIAA1522
Q9P219	S2007	CCDC88C	coiled-coil domain containing 88C
Q9P219	S2008	CCDC88C	coiled-coil domain containing 88C
Q9P275	S611	USP36	ubiquitin specific peptidase 36
Q9P2E3	S1823	ZNFX1	zinc finger, NFX1-type containing 1
Q9P2E3	S1837	ZNFX1	zinc finger, NFX1-type containing 1
Q9P2E3	S686	ZNFX1	zinc finger, NFX1-type containing 1
Q9P2E3	Y1841	ZNFX1	zinc finger, NFX1-type containing 1
Q9P2E9	S1403	RRBP1	ribosome binding protein 1
Q9P2R6	T658	RERE	arginine-glutamic acid dipeptide (RE) repeats
Q9UBG0	S1457	MRC2	mannose receptor, C type 2
Q9UBK8	S202	MTRR	5-methyltetrahydrofolate-homocysteine methyltransferase reductase
Q9UGN5	T262	PARP2	poly (ADP-ribose) polymerase 2
Q9UGN5	Y260	PARP2	poly (ADP-ribose) polymerase 2
Q9UGU0	Y1533	TCF20	transcription factor 20 (AR1)
Q9UGY1	T141	NOL12	nucleolar protein 12
Q9UH62	S70	ARMCX3	armadillo repeat containing, X-linked 3
Q9UHR5	S33	SAP30BP	SAP30 binding protein
Q9UJV9	S83	DDX41	DEAD (Asp-Glu-Ala-Asp) box polypeptide 41
Q9UKI8	S159	TLK1	tousled-like kinase 1

Q9UKJ3	S1087	GPATCH8	G patch domain containing 8
Q9UKJ3	S814	GPATCH8	G patch domain containing 8
Q9UKJ3	S820	GPATCH8	G patch domain containing 8
Q9UKJ3	S989	GPATCH8	G patch domain containing 8
Q9UKJ3	Y988	GPATCH8	G patch domain containing 8
Q9UKV3	S206	ACIN1	apoptotic chromatin condensation inducer 1
Q9UKV3	S605	ACIN1	apoptotic chromatin condensation inducer 1
Q9UKZ4	S1868	TENM1	teneurin transmembrane protein 1
Q9UKZ4	S1873	TENM1	teneurin transmembrane protein 1
Q9UKZ4	Y1865	TENM1	teneurin transmembrane protein 1
Q9ULD2	S541	MTUS1	microtubule associated tumor suppressor 1
Q9ULG1	S58	INO80	INO80 complex subunit
Q9ULH7	S834	MKL2	MKL/myocardin-like 2
Q9ULH7	T215	MKL2	MKL/myocardin-like 2
Q9ULH7	T217	MKL2	MKL/myocardin-like 2
Q9ULH7	T227	MKL2	MKL/myocardin-like 2
Q9ULJ3	S415	ZBTB21	zinc finger and BTB domain containing 21
Q9ULJ8	S184	PPP1R9A	protein phosphatase 1, regulatory subunit 9A
Q9ULT8	S1380	HECTD1	HECT domain containing E3 ubiquitin protein ligase 1
Q9UMN6	T1026	KMT2B	lysine (K)-specific methyltransferase 2B
Q9UN76	S21	SLC6A14	solute carrier family 6 (amino acid transporter), member 14
Q9UNF0	T424	PACSIN2	protein kinase C and casein kinase substrate in neurons 2
Q9UPN3	S2451	MACF1	microtubule-actin crosslinking factor 1
Q9UPQ0	S680	LIMCH1	LIM and calponin homology domains 1
Q9UPQ9	S561	TNRC6B	trinucleotide repeat containing 6B
Q9UPS6	T1679	SETD1B	SET domain containing 1B
Q9UPS6	Y849	SETD1B	SET domain containing 1B
Q9UPU5	S2081	USP24	ubiquitin specific peptidase 24
Q9UQ35	S1749	SRRM2	serine/arginine repetitive matrix 2
Q9UQ35	S202	SRRM2	serine/arginine repetitive matrix 2
Q9UQ35	S2731	SRRM2	serine/arginine repetitive matrix 2
Q9UQ35	S817	SRRM2	serine/arginine repetitive matrix 2
Q9UQ35	S834	SRRM2	serine/arginine repetitive matrix 2

Q9UQ35	Y1820	SRRM2	serine/arginine repetitive matrix 2
Q9UQR1	T789	ZNF148	zinc finger protein 148
Q9Y261	S306	FOXA2	forkhead box A2
Q9Y261	T297	FOXA2	forkhead box A2
Q9Y261	Y405	FOXA2	forkhead box A2
Q9Y261	Y411	FOXA2	forkhead box A2
Q9Y2D5	T131	AKAP2	A kinase (PRKA) anchor protein 2
Q9Y2G1	S294	MYRF	myelin regulatory factor
Q9Y2G1	S304	MYRF	myelin regulatory factor
Q9Y2H0	S844	DLGAP4	discs, large (Drosophila) homolog-associated protein 4
Q9Y2H0	S854	DLGAP4	discs, large (Drosophila) homolog-associated protein 4
Q9Y2H5	S453	PLEKHA6	pleckstrin homology domain containing, family A member 6
Q9Y2K7	Y22	KDM2A	lysine (K)-specific demethylase 2A
Q9Y2W1	S176	THRAP3	thyroid hormone receptor associated protein 3
Q9Y2W1	S34	THRAP3	thyroid hormone receptor associated protein 3
Q9Y2W1	S36	THRAP3	thyroid hormone receptor associated protein 3
Q9Y2W1	S38	THRAP3	thyroid hormone receptor associated protein 3
Q9Y2W1	S40	THRAP3	thyroid hormone receptor associated protein 3
Q9Y2W1	S533	THRAP3	thyroid hormone receptor associated protein 3
Q9Y2W1	Y54	THRAP3	thyroid hormone receptor associated protein 3
Q9Y383	S336	LUC7L2	LUC7-like 2 pre-mRNA splicing factor
Q9Y3T9	S22	NOC2L	NOC2-like nucleolar associated transcriptional repressor
Q9Y4B4	S744	RAD54L2	RAD54-like 2 (S. cerevisiae)
Q9Y4E1	S56	FAM21C	family with sequence similarity 21, member C
Q9Y4F1	S878	FARP1	FERM, RhoGEF (ARHGEF) and pleckstrin domain protein 1 (chondrocyte-derived)
Q9Y4F5	S785	CEP170B	centrosomal protein 170B
Q9Y4F5	S796	CEP170B	centrosomal protein 170B
Q9Y4F5	S809	CEP170B	centrosomal protein 170B
Q9Y4H2	S932	IRS2	insulin receptor substrate 2
Q9Y4K4	S362	MAP4K5	mitogen-activated protein kinase kinase kinase kinase 5
Q9Y4K4	T379	MAP4K5	mitogen-activated protein kinase kinase kinase kinase 5
Q9Y4P8	S412	WIPI2	WD repeat domain, phosphoinositide interacting 2
Q9Y4X0	S14	AMMECR1	Alport syndrome, mental retardation, midface hypoplasia and elliptocytosis chromosomal region gene 1

Q9Y520	S1489	PRRC2C	proline-rich coiled-coil 2C
Q9Y520	S1503	PRRC2C	proline-rich coiled-coil 2C
Q9Y5G4	S223	PCDHGA9	protocadherin gamma subfamily A, 9
Q9Y5G4	S224	PCDHGA9	protocadherin gamma subfamily A, 9
Q9Y618	S2205	NCOR2	nuclear receptor corepressor 2
Q9Y618	S2208	NCOR2	nuclear receptor corepressor 2
Q9Y618	S922	NCOR2	nuclear receptor corepressor 2
Q9Y618	T1569	NCOR2	nuclear receptor corepressor 2
Q9Y6D5	T1588	ARFGEF2	ADP-ribosylation factor guanine nucleotide-exchange factor 2 (brefeldin A-inhibited)
Q9Y6D5	T1597	ARFGEF2	ADP-ribosylation factor guanine nucleotide-exchange factor 2 (brefeldin A-inhibited)
Q9Y6J9	S605	TAF6L	TAF6-like RNA polymerase II, p300/CBP-associated factor (PCAF)-associated factor, 65kDa
Q9Y6J9	T604	TAF6L	TAF6-like RNA polymerase II, p300/CBP-associated factor (PCAF)-associated factor, 65kDa
Q9Y6M1	T243	IGF2BP2	insulin-like growth factor 2 mRNA binding protein 2
Q9Y6M1	T247	IGF2BP2	insulin-like growth factor 2 mRNA binding protein 2
Q9Y6M1	T251	IGF2BP2	insulin-like growth factor 2 mRNA binding protein 2
Q9Y6R1	S262	SLC4A4	solute carrier family 4 (sodium bicarbonate cotransporter), member 4
Q9Y6V0	S4244	PCLO	piccolo presynaptic cytomatrix protein
Q9Y6V0	S661	PCLO	piccolo presynaptic cytomatrix protein
Q9Y6V0	S663	PCLO	piccolo presynaptic cytomatrix protein
Q9Y6X6	S200	MYO16	myosin XVI
Q9Y6X6	S210	MYO16	myosin XVI
Q9Y6X6	S211	MYO16	myosin XVI

Table S3: Phosphoproteins shared by three celllines and involved in RNA related biological processes.

Accession ID	Gene Name	QGY Sites	QGY Quality	Hep3B Sites	Hep3B Quality	L02 Sites	L02 Quality	QGY/L02	Hep3B/L02
O75475	PSIP1	S273;S275;T267;T272;S177;S129;S106;S271;T122;S102;	1.78E-03	T272;S273;S275;S271;T169;S106;T115;S116;T167;	4.83E-04	S273;S275;S106;S129;T115;	1.92E-04	9.27E+00	2.52E+00
O75494	SRSF10	S129;S131;S133;S156;S158;S160;S119;S121;S123;S141;S251;T255;S256;	3.01E-03	S129;S131;S133;S123;S119;S121;S156;S158;Y142;	2.69E-03	S129;S131;S133;S156;S158;S160;S256;S119;S251;T255;S121;	2.16E-03	1.39E+00	1.24E+00
O95232	LUC7L3	S425;S431;T303;S304;	6.66E-05	S425;S431;	3.10E-04	S431;T419;S425;	1.39E-04	4.79E-01	2.23E+00
P07910	HNRNPC	S260;S253;S299;S233;S162;S239;	3.77E-03	S260;S299;S233;	7.34E-04	S260;S253;S299;S233;S162;S241;	3.20E-03	1.18E+00	2.29E-01
P08621	SNRNP70	S226;S268;S410;S293;S295;	2.22E-04	S410;S268;	5.75E-05	S410;S226;S268;	1.30E-04	1.71E+00	4.42E-01
P16383	GCFC2	S174;S180;S16;S17;S19;	5.77E-05	S16;S17;S19;S174;S180;S96;T97;S40;	1.82E-04	S16;S19;S25;S174;S180;S17;S96;T97;	2.11E-04	2.74E-01	8.65E-01
P18583	SON	S1780;S1782;S1556;S2009;S2011;S2013;S1697;S1783;S1769;S94;S1026;S2029;S2031;S283;S910;S2020;T2022;S1948;S1950;S1954;S1874;S1876;S1784;S1885;S1887;S1952;S1829;S1831;S1832;S1929;S1931;T1933;	1.86E-03	S2009;S2011;S2013;S1697;S283;S2020;T2022;S1950;S1952;S2001;S2003;S2129;S1940;S1942;T1555;S2029;S2031;S1948;S1829;S1831;	1.29E-03	S2009;S2011;S2013;S1697;S1948;S1950;S1952;S1784;S2029;S2031;S1782;S1783;S283;S2020;T2022;	1.30E-03	1.43E+00	9.92E-01
P22626	HNRNPA2B1	S259;S344;S236;	5.20E-04	S259;S212;	7.64E-04	S259;S212;S236;	6.35E-04	8.20E-01	1.20E+00
P24928	POLR2A	S1906;S1913;T1915;S1920;S1878;S1843;T1854;Y1853;S1882;S1847;	3.49E-04	S1913;T1919;Y1853;S1878;T1884;S1843;S1966;Y1909;T511;Y1916;	3.73E-04	Y1874;T1884;S1847;S1878;Y1909;S1910;S1920;S1913;S1843;Y1853;S1906;	9.25E-05	3.78E+00	4.03E+00
P26368	U2AF2	S79;	2.11E-04	S79;	3.22E-04	S79;	8.98E-05	2.35E+00	3.58E+00
P31943	HNRNPH1	S104;	1.00E-03	S104;	7.01E-04	S310;S104;	1.07E-03	9.37E-01	6.55E-01
P49756	RBM25	S677;S683;S583;	5.29E-04	S677;	9.15E-04	S677;S683;	6.90E-04	7.67E-01	1.33E+00
P52756	RBM5	S624;S621;S59;	7.03E-05	S621;S624;	5.47E-05	S621;S624;	2.61E-04	2.70E-01	2.10E-01
P61978	HNRNPK	S284;S379;	1.10E-03	S379;T118;S284;	7.26E-05	S379;S216;Y135;S284;	3.11E-04	3.55E+00	2.33E-01

P62995	TRA2B	S95;S97;S99;S264;S266;T33;S37;T201;T69;S71;S73;S280;S284;S102;S81;S83;S85;S39;S282;S29;Y86;S87;S22;	5.09E-03	S29;T33;S264;S266;S280;S284;S95;S97;S99;S39;S85;Y86;S87;S81;S83;S37;T69;S71;S26;	5.29E-03	S264;S266;S280;Y283;S284;S83;Y86;S87;T33;S37;S39;S95;S97;S99;S29;S85;T69;S71;S73;S81;	4.96E-03	1.03E+00	1.07E+00
P67809	YBX1	S174;S176;S314;S165;S167;S102;	1.67E-03	S165;S176;S314;S167;S174;	1.70E-03	S165;S176;S314;S174;	2.90E-03	5.75E-01	5.87E-01
Q00839	HNRNPU	S271;S59;	2.74E-04	S271;S764;S766;	2.62E-04	S271;S59;	5.27E-05	5.21E+00	4.98E+00
Q01130	SRSF2	S206;S208;S212;S187;S189;S191;S119;S121;S26;	5.51E-03	S206;S208;S212;S189;S191;S187;S119;S121;	5.69E-03	S187;S189;S191;S206;S208;S212;S26;S119;S121;S140;S142;	5.46E-03	1.01E+00	1.04E+00
Q07955	SRSF1	S234;S238;S199;S201;S205;S223;S227;S231;Y237;S242;Y202;S225;	5.83E-03	S234;S238;S199;S223;S227;S201;Y237;S205;	6.02E-03	S199;Y237;S238;S223;Y226;S227;S201;S205;S234;Y202;	5.01E-03	1.16E+00	1.20E+00
Q08170	SRSF4	S431;S444;S448;S458;S269;S267;S186;S188;S190;S446;S450;S290;S304;S322;	9.34E-05	S267;S269;S446;S448;S290;S464;S466;S444;	1.60E-05	S431;S444;S446;S448;S269;S456;S450;S458;S460;S267;S184;S186;S188;	4.86E-05	1.92E+00	3.29E-01
Q09666	AHNAK	S41;S210;S216;S5731;S511;S5763;S5841;S135;S5552;T5798;S5863;S5110;S5752;S5780;S5782;S5793;S93;S4908;S177;S4986;S5077;S115;T5839;S3054;S5784;S5864;S5867;S1042;S3426;S5762;S559;	7.72E-03	S216;S210;S212;S5731;S5752;S5782;T5839;S5077;S1088;S177;	2.13E-04	S115;S5752;S5763;S210;S216;S135;S5731;S5749;T4430;S5552;S41;S1042;T5839;S93;T4100;S5780;S4908;S5841;S3412;S5830;S5782;S5077;S177;S4360;T5794;S5110;S5790;S793;S1170;S3054;S4220;S4993;S5762;S5400;S5784;S5332;S220;T5798;S1298;S212;T4766;S3426;	1.43E-02	5.41E-01	1.49E-02
Q12872	SFSWAP	S909;S283;S604;S866;S868;S870;S815;	3.49E-05	S866;S868;S815;S283;S870;S604;S872;	6.29E-05	S604;S909;S283;S866;S868;S870;S815;	2.41E-04	1.45E-01	2.62E-01
Q12874	SF3A3	S365;S367;S369;	1.96E-05	S365;S367;S369;	3.90E-05	S365;S367;S369;T475;	1.25E-05	1.57E+00	3.13E+00
Q13242	SRSF9	S197;S199;S204;S211;S189;S193;S216;S195;	1.26E-03	S211;S216;S204;S195;	1.02E-03	S211;S216;S204;S197;S199;S189;S193;S195;	1.77E-03	7.11E-01	5.78E-01
Q13243	SRSF5	S246;S248;S250;S229;S231;S233;	5.28E-04	S208;S211;Y212;S248;S250;S253;S213;	2.52E-06	S250;S253;S229;S231;S233;	3.51E-04	1.50E+00	7.19E-03

Q13247	SRSF6	S314;S316;S297;S299;S303;S265;S272;S257;S212;S214;S259;S261;S263;	5.92E-03	S314;S316;S303;S295;S297;S301;S299;	4.26E-03	S314;S316;S265;S272;S303;	1.75E-03	3.38E+00	2.43E+00
Q14103	HNRNPD	S80;S83;S82;	3.31E-03	S83;S80;S82;T193;	3.93E-03	S80;S83;	1.20E-03	2.75E+00	3.26E+00
Q14671	PUM1	S229;S709;S247;	2.69E-05	S709;S209;S247;	1.58E-04	S709;	7.52E-05	3.58E-01	2.10E+00
Q15287	RNPS1	S155;S157;S251;S137;S139;S141;S27;S274;S276;	6.66E-04	S155;S157;T161;S137;S139;S141;S274;S276;S251;	1.29E-03	S155;S157;S251;S274;S276;S27;	3.97E-04	1.68E+00	3.24E+00
Q15459	SF3A1	S329;S359;	1.98E-04	S329;	3.53E-05	S329;S451;	9.94E-05	2.00E+00	3.55E-01
Q15637	SF1	S80;S82;	1.65E-03	S80;S82;	3.09E-03	S80;S82;	1.36E-03	1.21E+00	2.27E+00
Q16629	SRSF7	S231;S233;S192;S194;S171;S173;S175;S196;S163;S165;S200;S202;S204;S179;S181;S183;S130;S132;S134;S223;S225;S227;S167;	6.05E-03	S179;S181;S183;S192;S194;S173;S175;S196;S231;S233;S171;S223;S225;S227;S155;S157;S159;S165;S167;S163;	3.78E-03	S179;S181;S183;S192;S194;S196;S171;S173;S175;S163;S165;S167;	4.49E-03	1.35E+00	8.42E-01
Q16637	SMN1	S28;S31;	6.33E-04	S28;S31;T25;	1.05E-03	S28;S31;T25;	9.87E-04	6.42E-01	1.06E+00
Q53GS9	USP39	S82;	3.15E-05	S82;	1.36E-04	S82;S46;	1.49E-04	2.11E-01	9.12E-01
Q6PJT7	ZC3H14	S515;S409;S620;S132;S135;	4.03E-04	S515;S409;	3.56E-04	S515;S409;S132;S135;S620;	4.17E-04	9.68E-01	8.54E-01
Q8NC51	SERBP1	S234;T226;S197;S74;S25;T388;	1.48E-04	S234;S25;S394;	8.22E-05	S25;S234;S394;S74;T226;S392;	1.68E-04	8.82E-01	4.88E-01
Q8NDT2	RBM15B	S552;S109;S267;S562;S609;	1.45E-04	S552;S598;S600;S601;S562;	2.92E-05	S552;S265;S267;S113;	5.42E-05	2.68E+00	5.39E-01
Q96MU7	YTHDC1	S146;T148;S308;	1.01E-04	S308;S146;T148;	3.16E-04	S308;S146;T148;	2.45E-04	4.10E-01	1.29E+00
Q99590	SCAF11	S405;S413;S338;S776;S830;S832;S834;S608;S796;S802;S816;S818;S848;S850;S473;S878;S880;S882;S400;S401;S402;S937;S939;S941;S902;S798;S963;T410;S771;S943;	1.78E-03	S338;S830;S832;S834;S878;S880;S882;S816;S818;S848;S850;S796;S802;S839;	3.87E-04	S405;S413;S608;S472;S907;S796;S802;S816;S818;S830;S832;S834;S878;S880;S882;S338;S848;S850;S839;S475;S902;S937;S939;S943;	7.67E-04	2.32E+00	5.04E-01
Q9BUQ8	DDX23	S107;S109;S14;S39;S23;T25;S106;	8.87E-04	S106;S107;S14;S109;	6.38E-04	S16;S39;S106;S107;S14;S23;T25;S109;	4.43E-04	2.00E+00	1.44E+00
Q9H0G5	NSRP1	S33;S254;S255;S248;S289;S291;S293;	1.94E-04	S248;S254;S255;S33;S27;	3.43E-04	S33;S291;S293;S289;S248;S254;S255;	1.27E-04	1.53E+00	2.71E+00
Q9NYV4	CDK12	S681;S685;S274;S276;S383;S385;S423;S323;S325;T6	4.92E-04	S681;S685;S274;S276;S236;S251;S383;S385;S34	8.66E-04	S274;S276;S383;S385;S681;S685;S14;S18;T20;	4.08E-04	1.21E+00	2.12E+00

		92;S334;S1083;S357;S359;S338;S341;S343;S1082;S345;Y319;Y327;		3;S345;S334;S357;S359;S382;S332;S333;S1083;S320;S323;S325;		S1083;S355;S357;S359;S334;S301;S303;S1082;S423;			
Q9NZI8	IGF2BP1	S181;	7.82E-04	S181;	2.27E-03	S181;	3.48E-04	2.25E+00	6.53E+00
Q9UKV3	ACIN1	S216;T682;S240;S243;S490;S838;S410;T414;S1004;S386;S388;S478;S210;S1329;S1331;T1332;S710;S714;S384;S655;S657;T393;S729;T408;S208;S365;S605;	5.99E-03	S240;S243;S838;S216;S490;S410;S1004;S714;S1329;S1331;T1332;S655;S657;S729;S384;S386;S388;S825;T414;S208;S365;	3.11E-03	S240;S243;S208;S216;S490;T254;S410;T414;S838;S714;S655;S657;S1004;S386;S388;S898;S561;T393;S729;S1329;S1331;T1332;S384;S365;S710;T563;S206;T408;	4.86E-03	1.23E+00	6.40E-01
Q9Y2V2	CARHSP1	S30;S32;T45;S41;	3.35E-03	S30;S32;S41;S52;	1.04E-02	S30;S32;S41;T45;	2.05E-03	1.63E+00	5.09E+00
Q9Y2W1	THRAP3	S740;S743;S746;S939;S51;S53;S55;S928;S320;S698;S672;S379;S315;S243;S248;S253;S682;S406;S408;Y54;S533;S119;S134;S136;T941;S34;S36;S38;S917;S753;	5.01E-03	S928;S939;S248;S257;T941;S243;S315;S379;S672;S320;S51;S55;S682;S134;S136;S753;S34;S36;S40;S253;S406;S408;S53;	4.92E-03	S928;S939;T941;S51;S53;S55;S406;S408;S248;S253;S320;S243;S672;S379;S315;S682;S740;S743;S746;S399;S134;S136;S34;S36;S38;Y54;S575;S176;S753;S326;	5.08E-03	9.86E-01	9.68E-01
Q9Y383	LUC7L2	S323;S327;S336;S281;S283;S285;S358;S354;S383;S384;	2.09E-04	S336;S358;	7.87E-06	S336;S323;S327;S383;S384;S354;S358;	3.19E-05	6.56E+00	2.46E-01
P26599	PTBP1	T138;S141;	1.85E-04	S141;	1.15E-04	S141;	9.18E-05	2.02E+00	1.26E+00
Q86U44	METTL3	S43;	1.42E-05	S43;	3.04E-06	S43;S48;	3.33E-05	4.27E-01	9.12E-02

Table S4: Phosphoproteins shared by three celllines and involved in cell cycle related biological processes.

Accession ID	Gene Name	QGY Sites	QGY Quality	Hep3B Sites	Hep3B Quality	L02 Sites	L02 Quality	QGY/L02	Hep3B/L02
O00499	BIN1	S296;S303;S298;	1.62E-04	S296;S298;S331;	7.77E-05	S296;S298;S303;S331;S333;	5.04E-04	3.22E-01	1.54E-01
O14737	PDCD5	S119;	1.17E-03	S119;	2.57E-05	S119;	3.87E-05	3.02E+01	6.64E-01
O14974	PPP1R12A	S299;S445;T508;S509;S507;S871;S910;S908;	2.09E-04	S910;S871;S862;S507;	3.70E-05	T859;S871;S445;S507;T508;S908;S422;S509;S862;S910;	8.94E-04	2.33E-01	4.13E-02
O43159	RRP8	S62;S64;S104;S106;	6.48E-05	S62;S64;S104;S106;	1.99E-04	S62;S64;S104;S106;S176;S171;S174;	3.84E-04	1.69E-01	5.19E-01
O43290	SART1	S448;S474;S486;	2.24E-05	S591;S596;S598;S448;	9.85E-05	S591;S596;S598;S448;	1.71E-04	1.31E-01	5.76E-01
O43399	TPD52L2	S21;S166;	8.48E-04	S166;S96;	1.49E-03	S166;S96;	1.90E-03	4.47E-01	7.86E-01
O43815	STRN	S245;	3.63E-05	S245;	3.29E-05	S245;	1.34E-04	2.71E-01	2.46E-01
O60763	USO1	S942;	1.05E-05	S942;	2.56E-04	S942;	3.18E-04	3.32E-02	8.04E-01
O60832	DKC1	S494;S451;S453;S455;S485;S513;T497;	3.27E-04	S451;S455;S453;S513;S21;S494;T497;S485;	3.79E-03	S494;S451;S455;S453;S21;S513;S485;	3.09E-03	1.06E-01	1.23E+00
O60884	DNAJA2	S401;S394;S395;	3.70E-05	S394;S395;Y391;	1.23E-03	S400;S401;S394;S395;	6.58E-04	5.63E-02	1.87E+00
O60885	BRD4	S1126;T1080;S1083;S1117;	4.75E-05	S1126;S1117;	1.14E-05	S1126;S1117;	4.45E-05	1.07E+00	2.56E-01
O75116	ROCK2	S1137;S1134;S25;	3.89E-05	S1134;S1137;	1.97E-04	S1137;S1134;	2.13E-04	1.83E-01	9.25E-01
O75179	ANKRD17	S2044;S2047;S1696;S1940;S2045;S2059;Y2038;S1319;	9.86E-05	S19;S2401;S2041;S2042;S2047;S1696;	1.75E-04	S2045;S2047;T5;S2401;S1709;S19;S2067;S2059;S2042;	4.21E-04	2.34E-01	4.15E-01
O75351	VPS4B	S102;	3.75E-05	S102;	8.30E-05	S102;	2.62E-04	1.43E-01	3.17E-01
O75674	TOM1L1	S323;	2.07E-05	S323;	1.61E-04	S321;S323;	1.32E-04	1.56E-01	1.22E+00
O75691	UTP20	T1741;S2523;	3.68E-05	T1741;	6.77E-05	T1741;S2601;	7.98E-05	4.61E-01	8.48E-01
O94804	STK10	S438;T952;	2.23E-04	S438;	3.94E-05	S438;	2.23E-04	1.00E+00	1.77E-01
O94874	UFL1	S458;	5.00E-05	S458;	1.25E-04	S458;	1.89E-04	2.64E-01	6.59E-01
O95071	UBR5	S286;S287;S289;S2192;T2213;S1549;	1.67E-04	S287;S327;S1549;S289;	5.61E-04	S287;S1549;S286;	1.13E-04	1.48E+00	4.98E+00
O95359	TACC2	S2317;S2321;S2359;S2226;S2072;	2.92E-04	S2317;S2321;	2.43E-04	T2082;S2084;S2317;S2321;	4.60E-05	6.35E+00	5.29E+00

O95684	FGFR1OP	S156;S160;S321;S326;	1.02E-04	S156;S160;S152;	3.60E-04	S156;S160;	8.76E-05	1.17E+00	4.11E+00
O95997	PTTG1	S165;	3.91E-05	S165;	8.61E-06	S165;	1.15E-05	3.41E+00	7.49E-01
P02545	LMNA	S403;S404;S407;S615;T19;S22;S636;S414;S390;S392;S277;T416;S612;S12;S458;S613;S406;S652;T424;T394;	2.25E-02	S22;S628;S390;S392;S395;S406;S404;S407;S616;T19;S403;T409;S632;	4.14E-03	S616;S636;T19;S22;S406;S404;S407;S390;S392;T409;T394;S277;S414;S403;S612;S628;T416;	1.45E-02	1.56E+00	2.86E-01
P04049	RAF1	S43;T260;S621;T303;	9.65E-05	S621;S296;	4.21E-04	S642;T303;S43;S621;S289;S296;S301;	6.75E-04	1.43E-01	6.23E-01
P06748	NPM1	S125;S70;S254;S260;T234;T237;S243;S227;S222;T199;S139;T219;	6.79E-03	S125;T199;S227;S260;S243;S214;T219;	1.40E-03	S125;S70;T199;S260;T234;S139;S243;T219;S227;	3.88E-03	1.75E+00	3.62E-01
P07900	HSP90AA1	S263;S231;	3.99E-03	S263;S231;S252;	1.10E-02	S231;S263;	1.47E-02	2.71E-01	7.45E-01
P10644	PRKAR1A	S83;	1.20E-04	S83;T75;S77;	1.06E-03	S83;T75;S77;	5.07E-04	2.37E-01	2.09E+00
P11388	TOP2A	S1106;S1247;S1469;S1471;S1474;S1393;S1377;S1525;T1343;T1470;T930;T932;T934;Y935;S1374;T1112;S1332;S1337;S1351;S1213;S1392;	1.90E-03	S1247;S1374;S1377;S1393;S1106;S1471;S1474;T1470;S1525;T1397;S1332;S1337;T1343;S1469;S29;	2.71E-03	S1106;S1374;S1377;S1247;S1525;S1471;S1474;S1476;T1397;S29;	1.07E-03	1.78E+00	2.54E+00
P12270	TPR	S379;S2048;T2042;	1.30E-04	S2048;	1.76E-05	S2048;S1185;S2034;S2155;	6.42E-05	2.02E+00	2.73E-01
P16333	NCK1	S85;	3.54E-05	S85;	1.51E-05	S166;S85;	9.78E-05	3.62E-01	1.54E-01
P17096	HMGA1	S102;S99;S103;S36;T53;T39;S49;	1.30E-02	S99;S102;S103;S36;T53;T39;S44;	2.03E-02	S99;S102;S103;S36;T39;T53;	9.41E-03	1.38E+00	2.16E+00
P18858	LIG1	S911;S913;S76;T195;S66;S141;	3.31E-04	S911;S913;S66;S76;S141;T197;S47;S49;	6.13E-04	S66;S76;S141;S911;S913;T195;S199;S47;T183;S49;S51;	6.80E-04	4.86E-01	9.02E-01
P21333	FLNA	S1084;S1459;S2152;S1906;	3.54E-04	S2152;	2.08E-05	S1338;S2152;S1084;S1459;S2327;S2158;	1.77E-03	2.01E-01	1.18E-02
P25205	MCM3	S672;T674;S711;T713;T722;	1.07E-03	S672;T674;S711;T722;Y708;	3.68E-04	S672;T674;T722;S711;	4.27E-04	2.50E+00	8.62E-01
P25788	PSMA3	S250;	2.71E-03	S250;	8.07E-05	S250;	5.25E-04	5.15E+00	1.54E-01
P27816	MAP4	S507;S510;T521;S280;S789;S636;S624;S1073;S928;S358;T627;S787;T354;S115	3.90E-03	S636;S1151;S787;S822;T521;S928;T942;T270;S827;T82;S280;	2.76E-04	S1151;T521;S636;S99;T828;S787;T917;S928;T82;S94;T571;	7.83E-04	4.99E+00	3.53E-01

		1;T828;S822;T687;S941;							
P29590	PML	S36;S518;S527;S530;	3.05E-05	S518;S527;S530;	9.86E-05	S518;S527;S530;S36;S38;S40;S480;S403;S504;S505;	2.85E-04	1.07E-01	3.45E-01
P31350	RRM2	S20;	1.62E-04	S20;	1.50E-04	S20;	6.00E-04	2.70E-01	2.50E-01
P33991	MCM4	S26;S32;S31;	4.14E-05	S131;S142;S145;S26;S31;S32;S34;	5.67E-05	S131;S772;T774;T778;S26;S105;T110;	2.29E-05	1.81E+00	2.48E+00
P35221	CTNNA1	S641;T634;	3.86E-04	S641;S652;S655;T658;T654;T634;	2.26E-03	T634;S652;S641;T654;S655;	8.56E-04	4.51E-01	2.64E+00
P35251	RFC1	S69;S71;S73;S108;	3.40E-04	S69;S71;S156;T161;S312;S368;S73;S29;	1.50E-03	S69;S71;S368;	1.58E-04	2.15E+00	9.49E+00
P35580	MYH10	S1956;S1975;S1952;	5.41E-05	S1952;S1956;S1975;T1960;	2.22E-04	S1956;S1975;S1952;	1.20E-04	4.53E-01	1.85E+00
P35658	NUP214	T436;T987;S988;S433;T439;T670;S678;T1981;S1985;T2007;T437;S657;	3.72E-04	T434;T437;T436;T670;S678;S433;S457;	5.16E-04	S1023;S433;T437;S985;S986;T670;S678;S430;S648;S646;	9.30E-04	4.00E-01	5.55E-01
P46013	MKI67	S1861;S2828;S308;S2344;S357;S1376;S859;S1131;S2223;S2588;S2105;T2502;S2471;S1983;S579;S584;S2827;	1.54E-03	S308;S357;S2223;S2344;S1131;S859;T2231;S2588;S374;S579;S584;S1071;	2.77E-04	S308;S357;S1131;S3197;T1355;T1359;	9.42E-05	1.63E+01	2.94E+00
P46100	ATRX	S677;S1348;S1352;S92;S849;S850;S784;T977;S675;S1073;S1076;S876;Y89;S729;S731;S974;S978;	3.29E-04	S677;S675;S974;T977;S978;T674;S1073;S1076;S1061;S596;S1244;S1245;S1077;S849;S850;S875;S876;S1352;	1.25E-04	S677;S849;S850;S1352;S675;S598;T1230;S1236;S594;S1073;S1076;	1.02E-04	3.23E+00	1.23E+00
P46937	YAP1	S367;S289;S61;S138;S109;T337;	4.05E-04	S138;S61;S109;S131;T141;	2.30E-05	S138;S61;S127;S109;T143;S367;	3.49E-04	1.16E+00	6.57E-02
P48681	NES	S1409;S1418;S680;S471;S1615;S1617;S1618;S465;	3.99E-04	S1409;S1418;S471;S1496;S1498;S1492;S680;S1617;S1618;S768;S459;S352;S1615;S1489;S1502;T315;S320;S323;	1.24E-03	S1409;S1418;S471;S680;	4.35E-05	9.17E+00	2.86E+01
P49006	MARCKSL1	S104;S22;S120;	8.69E-05	S120;T178;S135;S22;T148;S48;S104;S93;S119;S71;	3.95E-03	S22;T178;T148;S104;S93;S120;T122;	1.07E-03	8.14E-02	3.70E+00
P49321	NASP	S451;S244;S726;S751;T477;S480;S421;	1.10E-04	T390;S726;	2.09E-05	T390;S726;S451;S244;	4.54E-04	2.42E-01	4.60E-02
P49736	MCM2	S108;S139;S41;S27;Y137;S	4.51E-03	S139;S108;S40;S41;T25;S26	3.29E-03	S139;T25;S26;S27;S	7.34E-03	6.14E-01	4.48E-01

		26;T25;T39;S40;		;S27;T39;Y137;		41;S40;			
P49790	NUP153	S334;S338;S192;S687;T691;	1.43E-04	S338;S334;	1.98E-05	S614;S619;S334;S338;S343;S209;S192;S217;	5.78E-05	2.47E+00	3.43E-01
P49792	RANBP2	S2287;S2290;T2293;S2510;S781;S1573;S2900;T1644;S2462;S796;T799;S2276;T1396;S2835;S2741;T2743;S1160;T779;S2280;T2458;S2858;S2831;S2278;	4.19E-04	S2741;T2743;S1509;S1160;T1396;S781;S2454;T779;S2280;S2900;S1456;T783;S1400;	4.29E-04	S2741;T2293;S2668;S2250;S1160;S2510;T19;S21;S955;S2900;S796;T799;S1400;T1396;T2743;T2458;S2462;S2454;T2450;S2278;S2280;S2241;S2251;S18;S2457;S2246;S1573;	9.13E-04	4.59E-01	4.71E-01
P49959	MRE11A	S688;S689;	2.41E-04	S649;	2.42E-05	S688;S689;	1.80E-05	1.34E+01	1.34E+00
P51610	HCFC1	S1507;S598;S1205;S666;	2.79E-04	S1507;S666;	8.01E-05	S1507;S666;S1205;S669;T413;	3.75E-04	7.44E-01	2.14E-01
P52701	MSH6	S252;S261;S254;S227;S65;	1.27E-04	T139;S227;S41;S252;S256;S261;S65;S63;S43;Y478;	3.66E-05	S252;S254;S261;S14;S65;S830;S256;S227;S137;	2.02E-04	6.29E-01	1.81E-01
P52756	RBM5	S624;S621;S59;	7.03E-05	S621;S624;	5.47E-05	S621;S624;	2.61E-04	2.70E-01	2.10E-01
P52948	NUP98	S623;S888;S1023;S1028;	1.10E-03	S623;S888;S1023;S612;S1028;S608;	6.90E-04	S623;S888;S1028;S1023;S612;	1.48E-03	7.48E-01	4.67E-01
P55327	TPD52	S223;S176;S171;	5.17E-04	S223;T173;S176;	5.60E-04	S223;T173;S171;S176;	1.59E-04	3.25E+00	3.52E+00
P56945	BCAR1	S139;S355;S428;T432;S434;	6.40E-05	S355;	2.47E-05	S139;S355;	3.14E-05	2.04E+00	7.86E-01
P62753	RPS6	S240;S242;S247;S235;S236;S244;	6.98E-04	S235;S236;S240;T241;S244;	6.26E-03	S240;T241;S235;S236;S246;S242;S244;S247;	4.91E-03	1.42E-01	1.28E+00
P78317	RNF4	S94;S95;	5.50E-05	S94;S95;	6.27E-05	S94;S95;	1.12E-04	4.90E-01	5.58E-01
Q00613	HSF1	T367;S368;S303;S363;T369;	1.84E-04	S303;S307;S314;S326;T367;T369;	4.74E-05	S363;T369;S303;S307;	1.39E-04	1.32E+00	3.40E-01
Q01082	SPTBN1	S2138;S2165;S2169;S2102;S2161;	2.17E-03	S2138;S2341;S2165;S2169;S2102;	4.59E-03	S2138;S2164;S2169;S2165;S2102;	1.47E-03	1.47E+00	3.12E+00
Q01831	XPC	S883;S884;	8.77E-05	S883;S884;	4.06E-04	S883;S884;S397;S398;S399;	4.22E-04	2.08E-01	9.62E-01
Q02880	TOP2B	S1400;S1413;S1424;S1522	4.21E-04	S1400;S1413;S1424;S1522;	1.49E-03	S1400;S1424;S1522;	1.75E-03	2.41E-01	8.53E-01

		;S1524;S1375;S1550;S1552;S1581;T1403;S1336;S1344;S1466;		S1524;S1375;S1466;S1581;S1526;T1575;S1550;S1552;T1403;S1461;		S1524;S1375;S1413;S1581;T1403;S1526;S1466;T1575;S1550;S1552;Y1609;S1336;S1340;S1344;S1457;S1461;			
Q03164	KMT2A	S3036;T3510;T3038;S3527;	5.49E-05	S3036;S2691;S2196;S2201;	1.55E-05	S1837;T1845;S3036;S2391;S2392;S523;	2.09E-05	2.63E+00	7.42E-01
Q08J23	NSUN2	S743;S751;S456;	8.83E-04	S743;S751;S456;	2.26E-03	S743;S751;S456;S473;	7.64E-03	1.16E-01	2.95E-01
Q12797	ASPH	S29;	2.65E-05	S14;S20;S29;	9.26E-05	S29;	3.53E-05	7.51E-01	2.62E+00
Q13177	PAK2	S141;Y139;	4.53E-05	S141;S58;	4.59E-05	S141;T169;T143;	4.80E-04	9.43E-02	9.56E-02
Q13200	PSMD2	S16;	7.22E-04	S16;S361;	1.21E-03	S16;	2.09E-03	3.45E-01	5.79E-01
Q13243	SRSF5	S246;S248;S250;S229;S231;S233;	5.28E-04	S208;S211;Y212;S248;S250;S253;S213;	2.52E-06	S250;S253;S229;S231;S233;	3.51E-04	1.50E+00	7.19E-03
Q13247	SRSF6	S314;S316;S297;S299;S303;S265;S272;S257;S212;S214;S259;S261;S263;	5.92E-03	S314;S316;S303;S295;S297;S301;S299;	4.26E-03	S314;S316;S265;S272;S303;	1.75E-03	3.38E+00	2.43E+00
Q13442	PDAP1	S60;S63;S57;S19;	3.86E-03	S60;S63;S57;	2.04E-03	S60;S63;S57;	1.64E-03	2.35E+00	1.24E+00
Q13541	EIF4EBP1	T37;S101;S65;T46;T77;S83;T70;T68;	1.47E-03	T37;T41;T46;	6.64E-04	T46;T41;S65;T68;T37;	1.55E-04	9.50E+00	4.29E+00
Q13547	HDAC1	S421;S423;S393;	2.07E-04	S421;S423;	1.35E-03	S421;S423;	8.34E-04	2.48E-01	1.62E+00
Q13769	THOC5	S312;S314;	2.40E-05	S312;S314;S307;	4.07E-04	S312;S314;S307;	7.09E-05	3.38E-01	5.74E+00
Q13895	BYSL	S98;	1.85E-04	S98;	6.48E-05	S98;	1.48E-04	1.25E+00	4.38E-01
Q14004	CDK13	S337;S340;S352;S437;S439;S383;S315;S317;S328;T442;S325;S395;S397;	1.00E-04	S383;S437;S439;S400;T1147;S315;S317;S325;S397;Y399;	2.12E-04	S315;S317;S325;T1147;S383;S395;S397;S437;S439;S411;S413;T496;S400;T500;S340;S342;S348;S441;S204;S205;S206;	2.29E-04	4.38E-01	9.26E-01
Q14676	MDC1	S168;T449;S453;T1425;S402;T404;S780;S988;S1711;S793;S1820;S1775;S299;T301;T455;T966;S998;S495;S498;S307;	1.54E-03	S372;T449;S453;S780;S299;T301;S1775;T455;S168;S1786;	5.94E-04	S780;S299;T301;S1775;T449;S1786;	2.11E-04	7.32E+00	2.82E+00
Q14839	CHD4	T1549;S1535;S103;S515;S531;S105;S108;	5.01E-04	S515;S531;S1535;S103;S105;S108;T529;S1537;	1.67E-04	T1549;S103;S105;S108;S515;S531;T529;	7.42E-05	6.75E+00	2.25E+00

						S1535;			
Q14978	NOLC1	S563;S698;S643;S508;S397;S538;T607;T610;S622;S623;	2.77E-03	S563;S698;S538;S397;T607;T610;S508;S643;	6.71E-04	S563;S538;S397;T607;T610;S698;S643;S508;	4.09E-04	6.78E+00	1.64E+00
Q14980	NUMA1	S169;S1757;T2000;S200;S203;S271;S1862;	2.01E-03	S169;S1853;S1757;	2.01E-04	S169;S1757;S1862;S271;T2000;	8.48E-04	2.37E+00	2.37E-01
Q15019	42980	S218;	1.48E-03	S218;	1.90E-03	S218;	4.02E-03	3.69E-01	4.72E-01
Q15121	PEA15	S116;	1.51E-04	S116;	1.27E-04	S116;	6.82E-05	2.21E+00	1.86E+00
Q15154	PCM1	S65;S1257;S1260;S1263;S68;S69;S1765;S1768;S1776;S159;T530;S533;S537;S430;S93;Y535;	2.80E-04	S1765;S1768;S1776;S65;S68;S69;S116;S119;T530;S537;Y535;	9.11E-04	S1768;S65;S1765;S1776;S1730;S430;S159;S68;S69;T530;S533;S537;S428;S116;S119;	4.14E-04	6.76E-01	2.20E+00
Q15637	SF1	S80;S82;	1.65E-03	S80;S82;	3.09E-03	S80;S82;	1.36E-03	1.21E+00	2.27E+00
Q15648	MED1	S1479;S1481;S1156;S1223;S1401;S1207;	1.59E-04	S1479;S1482;S1403;S770;S1481;T391;S774;	3.00E-05	S1481;S1156;S1437;S1479;	1.47E-04	1.09E+00	2.05E-01
Q16513	PKN2	S583;	3.87E-05	S583;	4.23E-05	S583;S535;S360;S21;T958;	3.94E-04	9.83E-02	1.07E-01
Q29RF7	PDS5A	S1305;	1.72E-04	S1195;S1305;	4.05E-04	S1305;	8.40E-05	2.05E+00	4.81E+00
Q53EL6	PDCD4	S94;S78;T93;	2.29E-04	S94;	2.18E-04	S76;	9.05E-06	2.54E+01	2.41E+01
Q53GS9	USP39	S82;	3.15E-05	S82;	1.36E-04	S82;S46;	1.49E-04	2.11E-01	9.12E-01
Q56P03	EAPP	S109;S111;	2.63E-05	S109;S111;T121;	2.89E-05	S109;T121;S111;	6.60E-05	3.99E-01	4.38E-01
Q5UIP0	RIF1	S2144;S1688;S1579;S2161;S2243;S2393;S2172;S2176;S1162;S2196;	2.59E-04	S2144;S1688;S1579;S2196;S1542;S1613;S2231;S2393;	6.07E-04	S2144;S1579;S1688;S1613;S782;S2393;	1.11E-04	2.33E+00	5.46E+00
Q68CZ2	TNS3	S776;S660;S850;	2.60E-05	S690;S660;S1154;S1115;S1123;Y780;S332;T692;S1149;	3.06E-04	S690;Y780;S660;S332;	1.28E-04	2.03E-01	2.39E+00
Q6KC79	NIPBL	S256;S280;S284;S306;S850;S2658;	9.82E-05	S2658;S274;S280;S284;S306;S2493;S2498;	1.70E-04	S1089;S1090;S1096;S2658;S280;S306;S2672;S284;S318;	3.01E-04	3.26E-01	5.66E-01
Q7Z460	CLASP1	S646;S647;S572;S727;T730;S731;	6.55E-05	S646;S649;S1091;S572;S731;S797;S600;S555;S1196;S647;S727;T730;	3.76E-04	S646;S1091;S723;S727;T798;S600;S649;S1196;S647;S797;S572;	3.38E-04	1.94E-01	1.11E+00
Q86WB0	ZC3HC1	S335;S62;S344;S338;S350;S407;	8.33E-05	S58;S62;S350;S338;S344;S407;S370;S335;S358;S24;T2	3.70E-04	S62;S335;S338;S344;S354;S407;S370;S3	6.90E-04	1.21E-01	5.37E-01

				8;		58;S321;S409;S410;			
Q8IVT2	MISP	S575;S543;S284;T287;S394;S395;	4.76E-05	S284;T287;S575;S394;	1.80E-04	S284;T287;S575;S394;S395;S397;S471;S214;S400;S586;S541;S675;S543;T377;S213;	9.22E-04	5.16E-02	1.96E-01
Q8N122	RPTOR	S863;T865;	3.59E-05	S877;S859;S863;S722;	1.86E-04	S859;S863;S877;S722;T865;	2.27E-04	1.59E-01	8.19E-01
Q8N5F7	NKAP	S157;T161;S149;S9;S36;	9.96E-05	S149;S46;S48;S25;S27;S85;S157;T161;	1.08E-04	S157;T161;S149;S9;	5.36E-05	1.86E+00	2.01E+00
Q8TAP9	MPLKIP	S115;S47;T51;	4.49E-05	S115;	3.05E-05	S47;T51;S115;	7.10E-05	6.33E-01	4.29E-01
Q8WVB6	CHTF18	S871;S64;	3.42E-05	S871;S64;	4.06E-05	S64;S871;	8.95E-05	3.83E-01	4.54E-01
Q8WWQ0	PHIP	S1783;S911;S1281;S1283;S879;S880;S881;	6.07E-05	S1281;S1283;S1783;S911;S879;S880;S881;	2.78E-04	S1281;S1283;S879;S880;S881;S1783;S911;S1315;S674;	2.45E-04	2.48E-01	1.14E+00
Q8WYP5	AHCTF1	S1160;S1222;S1283;S1541;S1944;S1218;S1214;S1216;	2.90E-04	S1160;S1218;S1944;S1541;S1884;S1222;S2222;S2226;	8.14E-05	S1160;S1232;S1222;S1944;S1541;S1218;	1.01E-04	2.87E+00	8.04E-01
Q8WZ42	TTN	S16647;S16661;	4.31E-04	S16649;S16661;	2.10E-03	S16661;Y6045;T6046;T14042;S14046;S16649;S21720;	5.73E-04	7.52E-01	3.67E+00
Q92530	PSMF1	S153;	2.44E-05	S153;	3.67E-04	S153;	2.92E-05	8.36E-01	1.25E+01
Q92538	GBF1	T1317;S128;S1298;S1318;	1.26E-04	Y1316;S1298;S1318;	1.01E-03	S1298;S1773;S1784;Y1316;S1318;S1300;	5.07E-04	2.48E-01	2.00E+00
Q92597	NDRG1	S330;S333;S336;	8.46E-05	S330;	3.97E-05	S330;T328;S333;T335;S336;	1.21E-04	7.00E-01	3.28E-01
Q92733	PRCC	S157;S159;S267;S212;T239;S241;S209;	1.11E-03	S267;S157;S159;T261;	2.65E-03	S157;S159;S267;S212;S209;T215;S241;	2.27E-03	4.91E-01	1.17E+00
Q92766	RREB1	S1653;S1219;S1225;S1320;	1.97E-05	S1219;S1225;S1167;S1174;S1175;S42;S36;S161;T31;T1121;	1.36E-04	S1167;S1174;S1175;	1.88E-05	1.05E+00	7.20E+00
Q92769	HDAC2	S422;S424;S394;	7.94E-04	S422;S424;	1.52E-03	S422;S424;S394;	1.62E-03	4.90E-01	9.35E-01
Q92974	ARHGEF2	S956;S941;S960;S932;S782;S645;	2.72E-04	S886;S932;S953;S960;S952;S645;S782;	9.33E-05	S953;S956;S932;S960;S645;S696;S782;	4.81E-04	5.65E-01	1.94E-01
Q96FS4	SIPA1	S55;	3.62E-05	S67;S55;	5.37E-05	S834;S839;S55;S67;	7.90E-05	4.59E-01	6.80E-01
Q96IZ0	PAWR	S233;S108;	2.27E-05	S231;	8.25E-05	S231;S108;	4.98E-05	4.56E-01	1.66E+00

Q96K21	ZFYVE19	S354;S144;S463;	9.74E-04	S354;	2.91E-04	S354;S144;S463;	3.87E-04	2.52E+00	7.52E-01
Q96NB3	ZNF830	S351;	2.28E-05	S351;	3.31E-05	S351;	6.05E-05	3.76E-01	5.47E-01
Q96PK6	RBM14-RBM4	T572;S582;S215;T206;	7.26E-04	S618;T206;S582;	2.61E-04	T206;S582;S215;Y648;S618;	2.87E-04	2.53E+00	9.10E-01
Q99618	CDCA3	S29;S31;S87;	9.86E-06	S31;T37;S29;	2.87E-05	S44;S29;S31;T37;S151;T161;S87;	3.11E-05	3.16E-01	9.21E-01
Q9BPX3	NCAPG	S1015;S973;S975;S674;	3.41E-05	S674;S1015;	7.13E-05	S674;S1015;	6.14E-04	5.55E-02	1.16E-01
Q9BQG0	MYBBP1A	S1267;T1161;S1163;T1239;S11;S1248;	1.37E-04	S1267;T1161;S1163;S1166;T1227;	1.46E-03	S1267;T1161;S1163;	9.67E-04	1.42E-01	1.51E+00
Q9BTA9	WAC	S525;S511;S53;	7.31E-05	S525;T293;S64;	3.52E-05	S511;S523;S535;S525;S64;	1.77E-05	4.14E+00	1.99E+00
Q9BXP5	SRRT	S67;S74;S540;T544;	2.64E-04	S540;S74;S67;T544;	2.26E-04	T544;S550;S74;S540;S493;S67;	5.61E-04	4.71E-01	4.02E-01
Q9H1H9	KIF13A	S1698;	7.81E-05	S1698;T1696;	6.05E-05	S1698;	3.84E-05	2.04E+00	1.58E+00
Q9H8Y8	GORASP2	S451;	3.89E-05	S451;T415;	3.81E-04	S451;S432;	2.68E-04	1.45E-01	1.42E+00
Q9HC35	EML4	S146;S176;S144;	5.09E-05	T899;S144;S146;S903;T96;T897;T160;	4.97E-04	S176;S144;S146;T899;	5.29E-05	9.62E-01	9.39E+00
Q9NQS7	INCENP	S828;S831;T832;S148;S899;S218;T219;S481;S197;S263;S306;T199;	2.74E-04	T213;S214;S306;S314;T199;T298;S312;S311;	1.33E-04	S197;T213;S481;S218;T219;T298;S314;	5.64E-05	4.86E+00	2.36E+00
Q9NQW6	ANLN	S99;S102;S54;S182;S323;S642;S95;S97;	3.09E-04	S54;S182;S225;S485;Y671;S672;	4.59E-04	S54;S225;S661;T320;S323;T364;S485;S642;	2.09E-04	1.48E+00	2.19E+00
Q9NWV8	BABAM1	S49;S29;	2.09E-05	S49;	2.12E-05	S66;S29;S49;	1.69E-04	1.24E-01	1.26E-01
Q9NYV4	CDK12	S681;S685;S274;S276;S383;S385;S423;S323;S325;T692;S334;S1083;S357;S359;S338;S341;S343;S1082;S345;Y319;Y327;	4.92E-04	S681;S685;S274;S276;S236;S251;S383;S385;S343;S345;S334;S357;S359;S382;S332;S333;S1083;S320;S323;S325;	8.66E-04	S274;S276;S383;S385;S681;S685;S14;S18;T20;S1083;S355;S357;S359;S334;S301;S303;S1082;S423;	4.08E-04	1.21E+00	2.12E+00
Q9NYZ3	GTSE1	S592;S575;S547;	6.70E-05	S592;S575;S583;S536;	2.27E-05	S477;S592;S575;S580;S138;S141;	1.91E-05	3.50E+00	1.18E+00
Q9UER7	DAXX	S495;S737;S739;	4.26E-05	S737;S739;S671;S668;S702;S688;S178;	2.14E-04	S671;S178;S737;S739;S668;S675;S702;S688;	1.03E-04	4.15E-01	2.08E+00
Q9UHD8	SEPT9	S30;S82;	1.01E-04	S30;	9.01E-04	S30;T42;T38;S80;S327;S85;	5.43E-04	1.85E-01	1.66E+00
Q9UK58	CCNL1	S352;	3.47E-05	S352;S329;T330;	8.70E-05	S335;S338;S342;S35	4.23E-04	8.20E-02	2.06E-01

						2;			
Q9ULW0	TPX2	S738;S121;S125;S186;	4.29E-04	S738;S292;S186;S121;S125;S293;	8.66E-04	S738;	1.75E-04	2.45E+00	4.94E+00
Q9UN37	VPS4A	S95;S97;S99;	1.93E-05	S95;S97;S99;	5.68E-05	S95;S97;S99;	1.99E-05	9.67E-01	2.85E+00
Q9UPN3	MACF1	S4521;	3.10E-05	S4521;S7330;	1.60E-05	S4521;S2451;S7292;T5594;	8.82E-05	3.52E-01	1.82E-01
Q9UPP1	PHF8	S857;	3.57E-05	S854;S857;S1021;	3.55E-05	S857;S854;S1021;S880;	9.34E-06	3.82E+00	3.80E+00
Q9UQ88	CDK11A	S271;	2.92E-05	S265;S271;S217;S222;S577;T583;T739;	1.67E-04	S271;S265;T739;	7.72E-05	3.78E-01	2.16E+00
Q9UQN3	CHMP2B	S199;	4.91E-05	S199;	1.10E-04	S199;	1.28E-04	3.84E-01	8.56E-01
Q9Y266	NUDC	T108;S139;T145;	2.76E-05	S139;T145;T108;	6.17E-05	S139;T108;S326;T145;	1.15E-04	2.39E-01	5.34E-01
Q9Y2X7	GIT1	S388;S592;S596;S385;T392;	3.54E-05	S385;S388;S592;T601;Y383;T392;	1.34E-04	Y383;S388;S592;S596;Y598;S362;S385;	3.08E-04	1.15E-01	4.34E-01
Q9Y5K6	CD2AP	S510;S514;	9.64E-06	S542;S510;T539;	3.74E-05	S514;S510;	3.50E-05	2.75E-01	1.07E+00
Q9Y6G9	DYNC1LI1	S510;T513;S516;T389;S207;S419;	2.62E-04	S207;T513;S516;S510;T512;T515;	8.33E-04	S516;S421;T513;S207;S398;S414;	1.69E-03	1.55E-01	4.93E-01
O00401	WASL	S484;S485;	5.03E-05	S484;S485;	1.53E-04	S484;S485;	1.16E-04	4.32E-01	1.31E+00
O14686	KMT2D	S3202;T3197;S3199;T2639;	2.19E-05	S1294;S1293;S1298;	4.87E-05	T3197;S3199;S4738;S1671;S2274;S2296;	6.16E-05	3.55E-01	7.91E-01
P17812	CTPS1	S574;S575;	1.71E-04	S575;S574;S573;S578;S568;	1.67E-04	S574;S575;S571;S573;S578;S562;	1.20E-03	1.42E-01	1.40E-01
P42684	ABL2	S817;S820;	1.69E-05	S817;S820;S620;S631;	5.64E-05	S936;S820;S631;S620;S819;	5.33E-05	3.17E-01	1.06E+00
P78344	EIF4G2	T508;	5.82E-05	T508;S902;	9.61E-05	T508;S17;	4.56E-04	1.28E-01	2.11E-01
Q9Y2U8	LEMD3	S140;S141;S144;S261;	3.82E-05	S140;S141;S144;S259;S261;	1.54E-04	S259;S261;S141;S140;S144;	6.73E-05	5.67E-01	2.29E+00

Table S5: Phosphoproteins shared by three cell lines and involved in top 20 signaling pathways.

Accession ID	Gene Name	Des	QGY Quality	Hep3B Quality	L02 Quality	QGY/L02	Hep3B/L02
O00418	EEF2K	eukaryotic elongation factor 2 kinase	2.77E-04	2.03E-04	8.51E-05	3.26E+00	2.38E+00
Q13085	ACACA	acetyl-CoA carboxylase alpha	1.13E-05	9.50E-05	1.17E-04	9.62E-02	8.09E-01
Q13541	EIF4EBP1	eukaryotic translation initiation factor 4E binding protein 1	1.47E-03	6.64E-04	1.55E-04	9.50E+00	4.29E+00
Q8N122	RPTOR	regulatory associated protein of MTOR, complex 1	3.59E-05	1.86E-04	2.27E-04	1.58E-01	8.19E-01
Q96B36	AKT1S1	AKT1 substrate 1 (proline-rich)	1.10E-03	1.66E-03	7.63E-04	1.44E+00	2.18E+00
O14974	PPP1R12A	protein phosphatase 1, regulatory subunit 12A	2.09E-04	3.70E-05	8.94E-04	2.33E-01	4.14E-02
O75116	ROCK2	Rho-associated, coiled-coil containing protein kinase 2	3.89E-05	1.97E-04	2.13E-04	1.83E-01	9.25E-01
P04049	RAF1	Raf-1 proto-oncogene, serine/threonine kinase	9.65E-05	4.21E-04	6.75E-04	1.43E-01	6.23E-01
P78347	GTF2I	general transcription factor IIi	6.44E-04	1.18E-04	8.43E-05	7.64E+00	1.40E+00
Q14814	MEF2D	myocyte enhancer factor 2D	1.21E-04	9.29E-06	3.08E-04	3.92E-01	3.02E-02
P16333	NCK1	NCK adaptor protein 1	3.54E-05	1.51E-05	9.78E-05	3.62E-01	1.54E-01
Q13177	PAK2	p21 protein (Cdc42/Rac)-activated kinase 2	4.53E-05	4.59E-05	4.80E-04	9.44E-02	9.56E-02
P42684	ABL2	ABL proto-oncogene 2, non-receptor tyrosine kinase	1.69E-05	5.64E-05	5.33E-05	3.17E-01	1.06E+00
P07900	HSP90AA1	heat shock protein 90kDa alpha (cytosolic), class A member 1	3.99E-03	1.10E-02	1.47E-02	2.71E-01	7.45E-01
P08238	HSP90AB1	heat shock protein 90kDa alpha (cytosolic), class B member 1	2.29E-02	5.46E-02	4.18E-02	5.49E-01	1.31E+00
P62753	RPS6	ribosomal protein S6	6.98E-04	6.26E-03	4.91E-03	1.42E-01	1.28E+00
P46937	YAP1	Yes-associated protein 1	4.05E-04	2.30E-05	3.49E-04	1.16E+00	6.58E-02
P10644	PRKAR1A	protein kinase, cAMP-dependent, regulatory, type I, alpha	1.20E-04	1.06E-03	5.07E-04	2.37E-01	2.09E+00
P13861	PRKAR2A	protein kinase, cAMP-dependent, regulatory, type II, alpha	1.87E-03	1.49E-03	3.17E-03	5.89E-01	4.70E-01
Q15642	TRIP10	thyroid hormone receptor interactor 10	1.37E-04	8.81E-04	3.25E-04	4.21E-01	2.71E+00
P04792	HSPB1	heat shock 27kDa protein 1	1.12E-02	1.19E-02	7.65E-03	1.46E+00	1.56E+00
P16949	STMN1	stathmin 1	5.98E-04	6.20E-04	9.79E-04	6.10E-01	6.33E-01
P21333	FLNA	filamin A, alpha	3.54E-04	2.08E-05	1.77E-03	2.01E-01	1.18E-02
Q9UER7	DAXX	death-domain associated protein	4.26E-05	2.14E-04	1.03E-04	4.15E-01	2.08E+00
P23588	EIF4B	eukaryotic translation initiation factor 4B	7.40E-03	3.40E-03	6.92E-03	1.07E+00	4.90E-01
Q8IUD2	ERC1	ELKS/RAB6-interacting/CAST family member 1	1.89E-04	1.36E-05	7.84E-05	2.41E+00	1.73E-01
Q13547	HDAC1	histone deacetylase 1	2.07E-04	1.35E-03	8.34E-04	2.48E-01	1.62E+00
Q86X95	CIR1	corepressor interacting with RBPJ, 1	2.18E-05	1.04E-04	6.72E-05	3.24E-01	1.55E+00
Q92769	HDAC2	histone deacetylase 2	7.94E-04	1.52E-03	1.62E-03	4.90E-01	9.35E-01

P31350	RRM2	ribonucleotide reductase M2	1.62E-04	1.50E-04	6.00E-04	2.70E-01	2.50E-01
Q9NYZ3	GTSE1	G-2 and S-phase expressed 1	6.70E-05	2.27E-05	1.91E-05	3.51E+00	1.19E+00
Q16513	PKN2	protein kinase N2	3.87E-05	4.23E-05	3.94E-04	9.82E-02	1.07E-01
Q7Z434	MAVS	mitochondrial antiviral signaling protein	2.73E-05	6.15E-04	6.15E-05	4.44E-01	1.00E+01
Q96G74	OTUD5	OTU deubiquitinase 5	1.99E-04	6.71E-05	4.75E-04	4.18E-01	1.41E-01
O75376	NCOR1	nuclear receptor corepressor 1	2.87E-04	3.86E-04	3.52E-04	8.16E-01	1.10E+00

Table S6: The list of phosphoproteins encoded by cancer driver genes

Accession ID	Gene Name	QGY Sites	Hep3B Sites	L02 Sites	QGY Quality	Hep3B Quality	L02 Quality	QGY/L02	Hep3B/L02
O00571	DDX3X	NA	S612;S92;S594;	NA	NA	1.75E-04	NA	NA	NA
O14497	ARID1A	S696;S366;S702;S363;S381;	S696;S702;S1182;	S1182;S696;	1.38E-04	4.41E-05	7.66E-05	1.80E+00	5.75E-01
O15119	TBX3	S371;S375;S707;S432;S435;	NA	NA	2.84E-04	NA	NA	NA	NA
O43493	TGOLN2	S71;	S71;T296;S70;S68;	S71;T296;S224;	8.09E-05	1.33E-04	2.07E-04	3.91E-01	6.44E-01
O60566	BUB1B	NA	NA	S543;S670;T1042;	NA	NA	5.19E-05	NA	NA
O60716	CTNND1	NA	S859;Y865;S349;S268;S346;S352;S269;T650;S651;	NA	NA	1.55E-04	NA	NA	NA
O60784	TOM1	S462;	S355;S462;S461;	S462;S464;	4.94E-05	7.07E-04	6.09E-05	8.11E-01	1.16E+01
O75175	CNOT3	S299;	NA	S299;	4.46E-05	NA	6.18E-05	7.22E-01	NA
O75376	NCOR1	S2184;S1472;S1206;S2136;S2151;S157;S2120;Y1966;S2436;S2438;S1977;S1981;S158;S172;	S2184;S2151;S1472;S224;S2436;S2438;S157;S172;S1322;S1977;S1981;S158;	S2436;S2438;S2184;S2151;S158;S2120;S224;S1977;S1472;S2136;S999;S1196;	2.87E-04	3.86E-04	3.52E-04	8.16E-01	1.10E+00
O75533	SF3B1	NA	NA	T211;T278;	NA	NA	2.33E-05	NA	NA
O75592	MYCBP2	NA	NA	S3467;	NA	NA	2.82E-05	NA	NA
O75962	TRIO	S2429;S2426;S2455;S2459;	NA	S2455;S2459;S2426;	3.69E-05	NA	4.55E-05	8.11E-01	NA
O95425	SVIL	S1000;	NA	Y966;S245;S968;	3.02E-04	NA	6.54E-05	4.62E+00	NA
O96028	WHSC1	S579;S580;S447;	NA	NA	5.22E-06	NA	NA	NA	NA
P00519	ABL1	NA	NA	S569;S1035;T1036;S1044;S718;	NA	NA	2.11E-05	NA	NA
P00533	EGFR	NA	S1039;S1042;T1041;S1166;Y1069;T693;S1037;	S1039;S1042;S1166;T693;Y1069;S1045;S1037;T1041;	NA	1.57E-04	2.17E-04	NA	7.24E-01
P02751	FN1	NA	NA	S1527;S1530;T1786;	NA	NA	1.54E-04	NA	NA
P04626	ERBB2	NA	NA	S1054;	NA	NA	9.81E-06	NA	NA
P06400	RB1	NA	NA	S249;S788;S794;S37;	NA	NA	4.28E-05	NA	NA
P06748	NPM1	S125;S70;S254;S260;T234;T237;S243;S227;S	S125;T199;S227;S260;S243;S214;T219;	S125;S70;T199;S260;T234;S139;S243;T219;S227;	6.79E-03	1.40E-03	3.88E-03	1.75E+00	3.62E-01

		222;T199;S139;T219;							
P07948	LYN	NA	Y508;S13;	S13;	NA	5.25E-05	8.48E-06	NA	6.19E+00
P08238	HSP90AB1	S255;S261;S226;	S255;S261;S226;	S255;S261;S226;	2.29E-02	5.46E-02	4.18E-02	5.49E-01	1.31E+00
P10644	PRKAR1A	S83;	S83;T75;S77;	S83;T75;S77;	1.20E-04	1.06E-03	5.07E-04	2.37E-01	2.09E+00
P12270	TPR	S379;S2048;T2042;	S2048;	S2048;S1185;S2034;S2155;	1.30E-04	1.76E-05	6.42E-05	2.02E+00	2.73E-01
P15056	BRAF	NA	S365;S151;	S365;S151;S447;S729;S363;	NA	6.64E-05	2.95E-05	NA	2.25E+00
P15408	FOSL2	S230;S200;	NA	S230;S16;S19;S307;S320;S314;S315;	1.62E-04	NA	4.62E-05	3.51E+00	NA
P16403	HIST1H1C	NA	NA	S36;S173;T146;T31;	NA	NA	2.84E-05	NA	NA
P20810	CAST	S243;	S243;T240;	S243;S364;S563;	6.77E-04	1.44E-03	8.14E-04	8.32E-01	1.77E+00
P21333	FLNA	S1084;S1459;S2152;S1906;	S2152;	S1338;S2152;S1084;S1459;S2327;S2158;	3.54E-04	2.08E-05	1.77E-03	2.01E-01	1.18E-02
P21359	NF1	NA	NA	S2515;S2817;	NA	NA	5.42E-05	NA	NA
P22607	FGFR3	NA	S444;T450;S445;	NA	NA	5.86E-06	NA	NA	NA
P25054	APC	S2671;S1044;S1360;S559;S2830;S1861;S1863;S1864;S1436;	S1861;S1863;S1864;S1360;S2088;S2093;S2830;S780;S2772;S2283;	S2088;S2093;S780;S1861;S1864;S2671;S1863;S2674;S1360;S2830;	6.49E-05	4.61E-05	4.41E-05	1.47E+00	1.05E+00
P27708	CAD	S1859;	S1859;	S1859;S1038;	1.83E-05	3.27E-04	8.26E-04	2.21E-02	3.96E-01
P27816	MAP4	S507;S510;T521;S280;S789;S636;S624;S1073;S928;S358;T627;S787;T354;S1151;T828;S822;T687;S941;	S636;S1151;S787;S822;T521;S928;T942;T270;S827;T82;S280;	S1151;T521;S636;S99;T828;S787;T917;S928;T82;S94;T571;	3.90E-03	2.76E-04	7.83E-04	4.99E+00	3.53E-01
P30622	CLIP1	S204;S147;S43;S48;	NA	S195;S44;S48;S204;S197;	2.58E-04	NA	5.49E-05	4.70E+00	NA
P31948	STIP1	S481;	S481;	S481;	1.19E-04	1.34E-04	2.45E-04	4.86E-01	5.45E-01
P33981	TTK	NA	NA	S281;S436;	NA	NA	4.82E-05	NA	NA
P35579	MYH9	S1943;	NA	S1943;T1151;	1.43E-02	NA	6.00E-03	2.38E+00	NA
P35658	NUP214	T436;T987;S988;S433;T439;T670;S678;T1981;S1985;T2007;T437;S657;	T434;T437;T436;T670;S678;S433;S457;	S1023;S433;T437;S985;S986;T670;S678;S430;S648;S646;	3.72E-04	5.16E-04	9.30E-04	4.00E-01	5.55E-01
P38159	RBMX	S208;S88;	S208;S88;	NA	4.06E-04	3.76E-04	NA	NA	NA

P38398	BRCA1	S395;S398;	NA	S395;S398;	1.63E-05	NA	1.92E-05	8.46E-01	NA
P41229	KDM5C	NA	NA	S1359;S301;	NA	NA	7.52E-05	NA	NA
P42345	MTOR	T1252;S1166;	NA	T1162;S1166;	2.54E-03	NA	3.90E-03	6.52E-01	NA
P46013	MKI67	S1861;S2828;S308;S2344;S357;S1376;S859;S1131;S2223;S2588;S2105;T2502;S2471;S1983;S579;S584;S2827;	S308;S357;S2223;S2344;S1131;S859;T2231;S2588;S374;S579;S584;S1071;	S308;S357;S1131;S3197;T1355;T1359;	1.54E-03	2.77E-04	9.42E-05	1.63E+01	2.94E+00
P46060	RANGAP1	S428;S442;	S428;S442;	S428;S442;S427;	1.28E-04	2.14E-04	2.50E-04	5.13E-01	8.55E-01
P46100	ATRX	S677;S1348;S1352;S92;S849;S850;S784;T977;S675;S1073;S1076;S876;Y89;S729;S731;S974;S978;	S677;S675;S974;T977;S978;T674;S1073;S1076;S1061;S596;S1244;S1245;S1077;S849;S850;S875;S876;S1352;	S677;S849;S850;S1352;S675;S598;T1230;S1236;S594;S1073;S1076;	3.29E-04	1.25E-04	1.02E-04	3.23E+00	1.23E+00
P46777	RPL5	NA	NA	S286;	NA	NA	2.54E-06	NA	NA
P46821	MAP1B	S1389;S1915;S1396;S1400;S1501;S1298;S1312;S1819;S2209;S2211;S1779;S1154;S614;S1782;S1443;S1797;S1965;S1438;T1633;T704;T1302;S1801;S1254;S1256;S1262;S1324;T1067;S1322;S1339;S1917;T1788;S1792;S831;S832;	NA	S1400;S1501;T1067;S1917;S937;S1396;S1298;S1312;S1779;S1782;T1949;S1016;S1915;S891;S1819;S1339;S1254;S1265;S1793;S1797;S1965;S1785;T1788;S2271;S1801;S614;S1852;S831;S832;S1322;T1328;S1276;T1282;S1881;S1252;S1154;S1256;S1260;S1792;S1412;T1334;S561;T1633;S2209;S2211;S1415;S1818;S1258;S1324;S1326;S1280;S1631;T704;	1.01E-03	NA	5.33E-03	1.89E-01	NA
P47974	ZFP36L2	S426;	NA	NA	2.90E-05	NA	NA	NA	NA
P48634	PRRC2A	S380;S383;S761;S342;S350;S1106;T1112;S1306;S1691;	S1306;S342;S350;S363;S764;T205;S766;S380;S1092;S1085;S1089;S1004;S1147;S761;S1106;T387;S1410;S932;S383;	S1306;S342;S350;S363;T610;S1147;S1219;S380;S761;	2.03E-04	6.61E-04	2.65E-04	7.66E-01	2.50E+00

P49006	MARCKSL1	S104;S22;S120;	S120;T178;S135;S22;T148;S48;S104;S93;S119;S71;	S22;T178;T148;S104;S93;S120;T122;	8.69E-05	3.95E-03	1.07E-03	8.14E-02	3.70E+00
P49768	PSEN1	NA	NA	S366;S367;	NA	NA	4.64E-05	NA	NA
P49792	RANBP2	S2287;S2290;T2293;S2510;S781;S1573;S2900;T1644;S2462;S796;T799;S2276;T1396;S2835;S2741;T2743;S1160;T779;S2280;T2458;S2858;S2831;S2278;	S2741;T2743;S1509;S1160;T1396;S781;S2454;T779;S2280;S2900;S1456;T783;S1400;	S2741;T2293;S2668;S2250;S1160;S2510;T19;S21;S955;S2900;S796;T799;S1400;T1396;T2743;T2458;S2462;S2454;T2450;S2278;S2280;S2241;S2251;S18;S2457;S2246;S1573;	4.19E-04	4.29E-04	9.13E-04	4.59E-01	4.71E-01
P49815	TSC2	NA	NA	S1371;S1385;S1388;S1411;T659;S664;T667;S1364;	NA	NA	7.25E-05	NA	NA
P51532	SMARCA4	S1570;S1575;S1586;S1421;S1617;S1620;T859;Y860;Y862;S1452;	S1452;S1570;S1575;S1586;S1382;T1423;S695;	T1423;S1570;S1575;S1586;S1452;T1425;S1417;S1421;S695;	1.18E-04	7.30E-05	7.59E-05	1.56E+00	9.62E-01
P52948	NUP98	S623;S888;S1023;S1028;	S623;S888;S1023;S612;S1028;S608;	S623;S888;S1028;S1023;S612;	1.10E-03	6.90E-04	1.48E-03	7.48E-01	4.67E-01
P55196	MLLT4	S1182;S1779;	S1182;Y1132;S1173;T1207;S1799;S1275;S1779;S1140;S1143;S1721;S1172;	NA	9.89E-05	7.98E-04	NA	NA	NA
P55317	FOXA1	NA	S331;S307;	S331;	NA	1.98E-04	3.01E-05	NA	6.58E+00
P58107	EPPK1	S2716;S2508;	NA	S2716;S2508;S1529;	6.88E-04	NA	2.51E-03	2.75E-01	NA
P78559	MAP1A	S2104;S1654;	NA	NA	2.95E-05	NA	NA	NA	NA
P98175	RBM10	S733;S736;S738;S723;	S736;S738;S89;S733;S723;	S736;S738;S733;S797;S723;S845;Y732;S89;S207;	7.40E-04	3.74E-04	1.02E-03	7.29E-01	3.68E-01
Q03001	DST	NA	S7510;S7513;S7502;	S7510;S7513;T5847;	NA	4.62E-06	5.15E-05	NA	8.97E-02
Q05655	PRKCD	NA	NA	S304;	NA	NA	2.59E-05	NA	NA
Q07352	ZFP36L1	NA	NA	S54;S92;	NA	NA	2.07E-05	NA	NA
Q07889	SOS1	S1082;S1210;	NA	NA	1.69E-05	NA	NA	NA	NA
Q09666	AHNAK	S41;S210;S216;S5731;S511;S5763;S5841;S135;S5552;T5798;S5863;S5110;S5752;S5780;S5782;S5793;S93;S4908;S177;S4986;S5077;S115;T5839;S3054;S5784;S5864;S5867;S10	S216;S210;S212;S5731;S5752;S5782;T5839;S5077;S1088;S177;	S115;S5752;S5763;S210;S216;S135;S5731;S5749;T4430;S5552;S41;S1042;T5839;S93;T4100;S5780;S4908;S5841;S3412;S5830;S5782;S5077;S177;S4360;T5794;S5110;S5790;S5793;S1170;S3054;S4220;	7.72E-03	2.13E-04	1.43E-02	5.41E-01	1.49E-02

		42;S3426;S5762;S559;		S4993;S5762;S5400;S5784;S5332;S220;T5798;S1298;S212;T4766;S3426;					
Q0JRZ9	FCHO2	S403;S394;S533;T570;S579;	S488;S496;S403;S533;	S488;S403;S496;S478;	7.31E-05	1.06E-04	1.97E-04	3.72E-01	5.39E-01
Q12873	CHD3	S1601;S1549;T715;	NA	T715;S1219;S1221;S713;S1545;	9.20E-05	NA	1.20E-04	7.64E-01	NA
Q12888	TP53BP1	S500;S1028;S294;S1462;S1114;S727;S482;S552;S525;S1317;S380;S1094;S1101;S1426;S1430;T1056;S831;S398;S862;S265;S1673;S1678;S1481;S316;S1759;S809;T1055;S1362;S523;S222;S1086;	S1028;S500;S1426;S1430;S380;S1362;S1758;S1759;	S294;S1028;S500;S1758;S1759;S222;S1618;S1430;S831;S834;S1426;S782;S784;S786;S380;S1481;T1056;S1362;S1068;T1654;S1460;S1701;S1705;S552;S830;T1756;S771;S809;S1462;S1646;S1480;S1673;S1678;S1094;S1101;	2.99E-03	1.86E-04	2.84E-03	1.05E+00	6.55E-02
Q13029	PRDM2	NA	NA	S164;S165;S168;S1572;	NA	NA	1.21E-06	NA	NA
Q13233	MAP3K1	S250;S21;	S21;	NA	7.18E-06	1.79E-05	NA	NA	NA
Q13283	G3BP1	S232;S149;	S149;S232;S231;	S232;S149;S231;	1.06E-02	9.06E-03	9.88E-03	1.08E+00	9.17E-01
Q13610	PWP1	S50;	S485;	S50;S485;	1.11E-04	1.01E-05	1.81E-05	6.11E+00	5.57E-01
Q13813	SPTAN1	NA	S1217;	S1217;	NA	4.69E-05	1.33E-05	NA	3.52E+00
Q14139	UBE4A	NA	S57;S50;S53;	NA	NA	4.12E-06	NA	NA	NA
Q14247	CTTN	T401;S405;S418;T399;T411;S417;Y421;	S418;S426;S11;T399;T411;Y421;T401;S405;S417;S113;T24;S33;S150;	S405;T411;T399;S417;T401;S418;	3.07E-03	2.89E-02	6.28E-03	4.89E-01	4.60E+00
Q14676	MDC1	S168;T449;S453;T1425;S402;T404;S780;S988;S1711;S793;S1820;S1775;S299;T301;T455;T966;S998;S495;S498;S307;	S372;T449;S453;S780;S299;T301;S1775;T455;S168;S1786;	S780;S299;T301;S1775;T449;S1786;	1.54E-03	5.94E-04	2.11E-04	7.32E+00	2.82E+00
Q14839	CHD4	T1549;S1535;S103;S515;S531;S105;S108;	S515;S531;S1535;S103;S105;S108;T529;S1537;	T1549;S103;S105;S108;S515;S531;T529;S1535;	5.01E-04	1.67E-04	7.42E-05	6.75E+00	2.25E+00
Q14865	ARID5B	S1032;	NA	NA	2.03E-05	NA	NA	NA	NA
Q14980	NUMA1	S169;S1757;T2000;S200;S203;S271;S1862;	S169;S1853;S1757;	S169;S1757;S1862;S271;T2000;	2.01E-03	2.01E-04	8.48E-04	2.37E+00	2.37E-01
Q15149	PLEC	S4406;S4382;S4385;S	S4626;S4386;S4391;S1435	S4626;S4406;S4618;T462	2.21E-03	2.03E-04	2.62E-03	8.45E-01	7.76E-02

		4386;S4396;S4618;S1732;S4626;S1435;T723;S4389;S4384;	;S4389;S4390;T4623;S4622;S4385;	8;S4386;S4390;S4382;S4385;S1435;S1732;S4389;S4622;S4630;S149;S125;S720;T4623;S4384;Y4611;					
Q15154	PCM1	S65;S1257;S1260;S1263;S68;S69;S1765;S1768;S1776;S159;T530;S533;S537;S430;S93;Y535;	S1765;S1768;S1776;S65;S68;S69;S116;S119;T530;S537;Y535;	S1768;S65;S1765;S1776;S1730;S430;S159;S68;S69;T530;S533;S537;S428;S116;S119;	2.80E-04	9.11E-04	4.14E-04	6.76E-01	2.20E+00
Q15233	NONO	T450;T428;	NA	T450;	1.91E-04	NA	1.11E-05	1.73E+01	NA
Q15365	PCBP1	S190;	NA	NA	2.53E-04	NA	NA	NA	NA
Q15652	JMJD1C	NA	NA	S639;S641;S652;	NA	NA	1.46E-05	NA	NA
Q15751	HERC1	NA	NA	T1429;S1514;S1521;T227;S237;S4857;	NA	NA	1.78E-05	NA	NA
Q15910	EZH2	NA	T487;T367;S375;	T487;T367;S84;	NA	1.90E-05	5.11E-05	NA	3.72E-01
Q15911	ZFHX3	S1590;S3409;S3418;	NA	NA	1.84E-05	NA	NA	NA	NA
Q29RF7	PDS5A	S1305;	S1195;S1305;	S1305;	1.72E-04	4.05E-04	8.40E-05	2.05E+00	4.81E+00
Q53SF7	COBLL1	S362;S1146;S1023;	S294;S518;S515;S1023;T298;	NA	4.23E-05	1.82E-04	NA	NA	NA
Q5SW79	CEP170	S1079;S1529;S838;S971;S928;S930;S933;S630;	S928;S933;T937;S930;S446;S838;	S928;S930;S933;S379;T1078;S446;S838;S1529;T632;T945;T948;S1112;S630;S1160;T1533;T937;S939;S1521;S1522;	8.04E-05	5.92E-05	2.86E-04	2.82E-01	2.07E-01
Q5TCZ1	SH3PXD2A	S547;S318;Y319;	S547;	S547;	1.74E-05	2.74E-05	1.16E-05	1.50E+00	2.36E+00
Q5TGY3	AHDC1	NA	NA	S1187;S1399;T1401;	NA	NA	1.18E-05	NA	NA
Q5UIP0	RIF1	S2144;S1688;S1579;S2161;S2243;S2393;S2172;S2176;S1162;S2196;	S2144;S1688;S1579;S2196;S1542;S1613;S2231;S2393;	S2144;S1579;S1688;S1613;S782;S2393;	2.59E-04	6.07E-04	1.11E-04	2.33E+00	5.46E+00
Q5VWQ8	DAB2IP	S35;T37;S33;	NA	NA	5.58E-04	NA	NA	NA	NA
Q5VZL5	ZMYM4	T118;S122;	S122;T118;S1542;S1547;	S122;S1241;S1542;S1547;T118;S1539;	5.61E-05	4.05E-05	1.85E-04	3.03E-01	2.19E-01
Q63HN8	RNF213	NA	NA	S1258;S2273;	NA	NA	1.72E-04	NA	NA

Q68CP9	ARID2	NA	NA	S689;S1300;	NA	NA	1.62E-05	NA	NA
Q6GYQ0	RALGAPA1	NA	NA	S797;S864;S860;	NA	NA	6.80E-05	NA	NA
Q6KC79	NIPBL	S256;S280;S284;S306;S850;S2658;	S2658;S274;S280;S284;S306;S2493;S2498;	S1089;S1090;S1096;S2658;S280;S306;S2672;S284;S318;	9.82E-05	1.70E-04	3.01E-04	3.26E-01	5.66E-01
Q6UUV7	CRTC3	NA	NA	S329;S443;	NA	NA	1.26E-05	NA	NA
Q6VMQ6	ATF7IP	S474;S559;S113;T860;S862;S852;	S113;	S113;T118;S673;	2.70E-04	3.86E-05	9.56E-05	2.83E+00	4.03E-01
Q6Y7W6	GIGYF2	S26;S30;S201;S160;T382;	S26;S201;S30;S160;S19;S23;T382;	T382;T25;S26;S160;S392;S30;S201;S236;	2.57E-04	3.20E-04	4.67E-04	5.51E-01	6.85E-01
Q6ZRS2	SRCAP	S2790;S3177;T2425;S2430;S3148;S274;S1859;	S3148;S2790;	S3148;S808;T812;Y821;	6.26E-05	2.03E-05	1.09E-05	5.73E+00	1.86E+00
Q7Z333	SETX	NA	S1017;S1019;S687;S692;S693;	NA	NA	2.06E-05	NA	NA	NA
Q7Z6Z7	HUWE1	S2362;S2365;T2366;S1907;	S1907;S2362;S2365;S3373;S2887;T2889;S3919;T3924;T3927;S3662;	S1907;S2595;S3373;S2362;S2365;S3919;T3924;T3927;S2593;S3936;S2918;S3662;S2887;S2888;	4.95E-05	2.91E-04	1.21E-03	4.11E-02	2.42E-01
Q86U86	PBRM1	S1453;S648;Y462;Y470;	S1453;S10;	S1453;S648;	1.57E-05	1.69E-05	2.44E-05	6.44E-01	6.96E-01
Q86UU0	BCL9L	S116;S118;S21;S25;S813;S750;	S116;S118;S21;S25;	S88;S21;S25;S118;	1.72E-04	3.33E-05	1.08E-04	1.59E+00	3.08E-01
Q8IVF2	AHNAK2	S294;S280;T5709;	NA	S294;S593;S280;T5709;S765;S3408;S1112;S509;	9.13E-05	NA	3.72E-04	2.46E-01	NA
Q8IVL0	NAV3	NA	NA	S1189;S1044;S275;	NA	NA	1.97E-05	NA	NA
Q8IWS0	PHF6	S199;S204;S155;S138;S203;S206;	S155;S203;S204;S199;	S154;S138;S4;T12;S155;S199;S203;	2.03E-04	3.00E-05	1.02E-04	1.99E+00	2.94E-01
Q8IYB7	DIS3L2	NA	S875;	S875;	NA	1.22E-04	5.09E-05	NA	2.39E+00
Q8N1F7	NUP93	NA	NA	S767;S769;	NA	NA	3.04E-04	NA	NA
Q8N201	INTS1	NA	NA	S1326;S1318;	NA	NA	1.12E-04	NA	NA
Q8NF91	SYNE1	T3803;S1993;T2001;	NA	NA	2.80E-05	NA	NA	NA	NA
Q8TBA6	GOLGA5	NA	S116;	NA	NA	6.77E-06	NA	NA	NA
Q8TDZ2	MICAL1	NA	S872;S875;S876;	NA	NA	2.20E-05	NA	NA	NA
Q8TEK3	DOT1L	NA	NA	S1001;S1009;S826;S834;	NA	NA	5.51E-06	NA	NA

Q8WYP5	AHCTF1	S1160;S1222;S1283;S1541;S1944;S1218;S1214;S1216;	S1160;S1218;S1944;S1541;S1884;S1222;S2222;S2226;	S1160;S1232;S1222;S1944;S1541;S1218;	2.90E-04	8.14E-05	1.01E-04	2.87E+00	8.04E-01
Q93008	USP9X	NA	NA	S2563;	NA	NA	2.59E-05	NA	NA
Q96IZ7	RSRC1	S6;S116;S61;	NA	S6;	1.66E-05	NA	1.89E-05	8.82E-01	NA
Q96L73	NSD1	NA	NA	S2471;	NA	NA	9.47E-06	NA	NA
Q96PU5	NEDD4L	NA	S446;S448;S342;	S479;S483;S487;S446;	NA	1.91E-04	1.16E-05	NA	1.65E+01
Q96RK0	CIC	S1382;S1397;S1409;S431;T435;	S1373;S1382;S1397;S1402;	S1373;S1382;S700;S173;	1.97E-05	2.85E-05	3.61E-05	5.45E-01	7.89E-01
Q96RT1	ERBB2IP	S602;S603;S857;S1015;	T917;S857;	S1015;S857;S913;	6.32E-05	4.64E-05	3.59E-05	1.76E+00	1.29E+00
Q96T58	SPEN	S736;S740;S1222;S2493;S1006;S725;S727;S1918;S1390;S749;S2120;S2124;S1268;S1278;S1261;S1380;S1382;S2366;T2393;S2101;S1857;	S2120;S2124;S1380;S1382;S1390;T1643;S1261;	S1268;S1278;S1380;S1382;S1390;S1225;S2120;S2124;S736;S740;S725;S727;S1392;T2374;S1222;S250;	2.18E-04	4.80E-05	1.83E-04	1.19E+00	2.63E-01
Q96TA1	FAM129B	S665;S646;S641;S692;S696;S681;S638;	S641;S646;S665;S681;S692;S696;	S665;S641;S646;S681;S638;S692;S696;	9.03E-04	5.78E-04	2.64E-03	3.42E-01	2.19E-01
Q99081	TCF12	S558;S559;S67;	NA	S67;S559;S332;	9.77E-05	NA	4.93E-05	1.98E+00	NA
Q9BTC0	DIDO1	T151;S152;S154;S1456;T1256;S1260;S805;S809;S898;T1255;S1040;T1719;	T151;S152;S154;S805;S809;S834;S835;T1256;S1260;S1040;S1456;	S1456;T151;S152;S154;S1260;S1040;T1255;S805;S809;T1733;T1256;	7.06E-04	2.55E-04	7.38E-04	9.56E-01	3.46E-01
Q9BVJ6	UTP14A	S29;S31;S437;S405;S445;Y26;	S29;S31;S445;S405;S407;	S29;S31;S405;S407;S437;	3.35E-04	3.00E-04	2.30E-04	1.45E+00	1.30E+00
Q9BXF6	RAB11FIP5	S393;S307;	NA	NA	2.27E-05	NA	NA	NA	NA
Q9BXJ9	NAA15	NA	S856;S855;	S856;S855;T850;	NA	1.50E-04	1.70E-04	NA	8.77E-01
Q9BYW2	SETD2	S458;S459;S461;S744;S754;S2080;S2082;S321;S323;S1413;S1415;S1417;S624;	S321;S323;S1413;S1415;S1417;	S458;S459;S461;S624;S1413;S1415;S1417;S321;S323;	9.11E-05	2.43E-05	6.67E-05	1.37E+00	3.64E-01
Q9C0C2	TNKS1BP1	S762;S1620;S1621;S494;S1666;S836;S672;S691;S601;S1138;S221;	S1620;S1621;S601;S1473;S1476;S429;S836;S1029;S1385;T501;S672;S691;S16	S984;S836;S601;S691;S695;S712;S1029;S494;S498;S1666;S672;S429;S936;	2.54E-03	8.78E-04	3.30E-03	7.69E-01	2.66E-01

		S1103;S1297;S498;S1029;S712;S1715;S228;S872;	66;S178;S1024;S504;S987;S872;	S1138;S1024;S1046;S1620;S1621;S987;T501;S893;S1473;S1476;S1715;S221;S228;S1652;S920;S178;S1031;S872;S435;S1385;S1103;					
Q9H1A4	ANAPC1	NA	S686;S547;S555;S563;	S688;S345;	NA	8.63E-06	2.29E-05	NA	3.77E-01
Q9H4L7	SMARCAD1	S211;S213;S34;S39;T54;S57;Y217;S95;S96;S37;S50;S214;S124;S127;	S34;S39;S211;S214;S95;S96;S98;Y217;T54;S57;	S96;S98;S103;S34;S39;S37;S50;S212;S213;S214;S95;T54;S57;S211;Y217;	2.34E-04	2.21E-04	3.70E-04	6.31E-01	5.98E-01
Q9H6Z4	RANBP3	S96;S101;S108;S333;S100;	S353;S108;S333;S96;S101;S100;	S96;S101;S108;S100;S333;S353;T214;	4.07E-04	9.69E-04	6.24E-04	6.52E-01	1.55E+00
Q9HCD6	TANC2	NA	NA	S1827;S1579;T1315;S1722;	NA	NA	7.72E-05	NA	NA
Q9HCE5	METTL14	NA	S399;	NA	NA	4.06E-06	NA	NA	NA
Q9HCK8	CHD8	NA	NA	S2519;S2008;T1993;S1995;	NA	NA	5.67E-05	NA	NA
Q9NQX3	GPHN	NA	S188;S194;S200;	NA	NA	6.83E-06	NA	NA	NA
Q9NRY4	ARHGAP35	NA	S975;S985;S980;S1179;	S975;S1179;S985;	NA	3.48E-04	6.80E-05	NA	5.12E+00
Q9NTJ3	SMC4	S22;S27;S28;	S22;S27;S28;	S22;S27;S28;S41;	5.54E-04	5.11E-04	7.31E-04	7.58E-01	6.99E-01
Q9NU22	MDN1	T4898;S5015;	S4538;S5015;T4898;	T4898;S5015;S4538;	1.94E-05	1.04E-04	2.86E-05	6.78E-01	3.65E+00
Q9NYF8	BCLAF1	S397;S264;S268;S222;S385;S177;S297;S496;S760;S183;S102;S104;S290;Y284;S512;S285;S658;S119;S122;S450;S531;S648;S17;S19;S20;T726;S763;T257;S759;S262;S23;S25;S27;T661;T402;S757;S161;S717;S718;	S397;T402;S385;S177;S512;S281;S290;S496;S222;S183;S757;S759;T257;S268;S658;S272;S102;S104;S161;S531;S297;S264;Y284;S259;S760;S274;S660;S717;S718;	S397;T402;S222;S177;S385;S290;S512;S183;Y511;T257;S264;S102;S104;S658;S285;S660;S119;S122;S496;S531;T726;Y383;S268;S181;Y284;S648;S760;S389;	9.28E-03	1.00E-02	1.18E-02	7.89E-01	8.52E-01
Q9NYV4	CDK12	S681;S685;S274;S276;S383;S385;S423;S323;S325;T692;S334;S1083;S357;S359;S338;S34	S681;S685;S274;S276;S236;S251;S383;S385;S343;S345;S334;S357;S359;S382;S332;S333;S1083;S320;S32	S274;S276;S383;S385;S681;S685;S14;S18;T20;S1083;S355;S357;S359;S334;S301;S303;S1082;S423;	4.92E-04	8.66E-04	4.08E-04	1.21E+00	2.12E+00

		1;S343;S1082;S345;Y319;Y327;	3;S325;						
Q9P2D1	CHD7	NA	S2956;S2983;S2559;	NA	NA	3.07E-05	NA	NA	NA
Q9UDY2	TJP2	S1159;S170;S174;S244;S986;S966;S266;S441;S398;S400;S292;S294;S296;S961;	S398;S400;S130;T925;S1067;S1068;S170;S174;S244;S978;S986;T933;S292;S294;S296;S394;S1159;S961;S266;Y423;Y428;S163;S966;T1027;S979;S702;	S130;S1159;S1068;S986;S398;S400;S244;T925;T933;S170;S174;S266;S292;S294;S296;S966;S163;	1.91E-04	6.63E-03	9.32E-04	2.05E-01	7.11E+00
Q9UIF9	BAZ2A	T1399;S1770;S1397;	S1783;	NA	7.26E-05	1.12E-05	NA	NA	NA
Q9UIG0	BAZ1B	S158;S1468;	S1468;S705;S708;S1342;S158;	S708;T710;S1468;S947;S705;S158;S161;S160;	5.51E-05	6.58E-04	4.98E-04	1.11E-01	1.32E+00
Q9ULU4	ZMYND8	S490;S668;S655;S486;S488;	S490;S486;S668;S655;	S486;S488;S490;S655;S652;S547;S668;	2.73E-04	1.88E-04	2.18E-04	1.26E+00	8.63E-01
Q9UPN3	MACF1	S4521;	S4521;S7330;	S4521;S2451;S7292;T5594;	3.10E-05	1.60E-05	8.82E-05	3.52E-01	1.82E-01
Q9UPY3	DICER1	NA	S413;S415;	NA	NA	2.75E-05	NA	NA	NA
Q9UQ35	SRRM2	S377;S398;S1387;T2316;S1648;S295;S297;S857;S950;S952;S954;T983;S994;S1497;S1499;S1502;T384;T856;S1818;S1822;S1824;T866;S1404;S2272;S1542;S1552;S1579;S1581;S1582;T1003;S1014;S1384;S435;S436;S437;S1598;S1600;S1601;S1691;S1693;S1694;T1413;T848;S972;S973;S974;S875;S876;S1420;S1421;S1415;S1401;S741;S743;S746;T2738;S2740;S346;S351;S353;S562;S564;S566;S2702;S2706;S1864;S1866;S1869;S440;T1231;S1441;S1443;S1444;S21	S377;S398;S1383;S1387;T1003;S1014;S295;S297;S351;S353;S358;S1064;S1069;S1497;S1499;S1502;T384;S854;S857;S1099;S1101;S950;S952;S954;S2100;T2104;S322;T326;S300;S957;S1318;S1320;S1329;S846;S323;S2690;S2692;S1539;S1552;S974;S1478;S1482;S1483;S876;S778;S780;T866;S435;S436;S562;S564;S566;S2702;T983;T2289;S1188;S2067;T2069;S2071;S2706;S1600;S1601;S2449;S437;S1579;S1581;S1582;S1441;S1913;S1916;S204;S1864;S1866;S1869;S1598;S972;S973;S2398;S2030;S2032;T2034;S2018;S2020;T2022;S506;S508;S510;S1	S377;S398;S1387;S1320;S1326;S1329;T384;T1003;S1014;S322;S323;S950;S952;S954;S351;S353;S1099;S1101;S1102;S1579;S1581;S1582;S1497;S1499;S1502;S875;S876;S1818;S1824;S973;S974;S2449;S2272;S295;S297;S1691;S1693;S1694;S1864;S1866;S1869;S435;S436;S437;S1152;S1064;S1069;T1071;S2690;S2692;S2694;S1482;S1483;S846;T848;S1103;S1542;S1550;S2453;S1539;S1552;S2702;S2706;S1403;S2100;S2102;T2104;S857;S506;S508;S510;S778;S780;S2067;T2069;S2071;S1762;S1764;S901;S908;S910;S1916;S14	1.03E-01	6.52E-02	6.71E-02	1.54E+00	9.71E-01

		00;S1482;S1483;S1762;S1764;S778;S780;S783;T1453;S1923;S1925;T1927;S1975;T2409;S2102;T2104;S2407;S2412;S2042;S2044;S2046;T1071;S1072;S1073;S2382;S854;S506;S508;S510;S2121;S2123;S1103;S322;S323;S901;T903;S908;S1458;S1460;S988;S990;S1517;S1519;S1320;S2581;S1616;S1620;S1621;S2415;S1179;S2018;S2020;T2022;S2132;S1462;S1463;S2067;T2069;S2071;S1318;S1329;S2690;S2692;S2694;S1972;S1857;S1854;S895;S1132;S2030;S2032;T2034;S1124;S2449;S892;S894;S2398;S838;S1888;S1890;S1101;T1106;S1982;S1984;S1987;S1478;T1531;S472;S478;S1893;S474;S864;S2684;S1112;T1434;T476;S534;S536;S1911;S1913;S1916;S992;S454;S455;S456;S713;S715;T717;T1698;T810;T1205;T1472;S2727;S2731;S1423;S1424;T1856;S883;S887;S808;S1900;S1902;S1905;S543;S761;S763;S764;S1970;S1876;S1878;T	923;S1925;T1927;S1103;S1110;S1672;S1674;S1675;S2581;S1384;S2042;S2044;S2046;S1693;S1694;S1935;S1937;T1939;S1975;S1124;S1444;S1762;S1764;S1083;T1531;S2694;S472;S474;S478;S2688;S575;T577;S1691;S346;S2102;S625;T627;S629;S1984;T1986;S2412;S2675;S2677;S2407;S819;S817;S818;S783;S395;S713;S715;T717;S2090;T2092;S1888;S1890;S484;S486;T1856;S1857;T848;S1747;S1749;S1750;S1682;T1684;S1911;T903;S908;S1902;S1905;S702;S704;S706;S1893;T1904;S901;S573;S543;S248;S250;T252;S594;S596;T598;S455;S456;S1382;S1326;S387;S875;S1542;S1102;S2453;T856;S1219;Y996;T2316;S892;S1690;S1443;S1987;S534;S536;T1698;S200;S202;S1896;S2415;T2409;S910;T251;S759;S761;S763;S764;S1982;S839;S1112;S895;T489;S808;T810;S1822;S1824;T1071;S440;S988;S2417;T476;S1179;S820;S1970;S1972;S1517;S1854;	04;T983;S988;S1441;S1443;S1444;S2030;S2032;T2034;S1923;S1925;T1927;S1987;S440;S1318;S2044;S2046;T2316;T2329;S1188;S1478;S1179;S2407;S2412;S892;T2289;S2398;S2727;S566;T569;T2409;S2581;S472;S474;T476;S2020;T2022;T903;S1112;S2042;S713;S715;T717;S1598;S1600;S248;S250;S2018;S534;S536;S1888;S1890;S783;T866;S1682;S1672;S2688;S1124;S1911;S1913;T1856;S1857;S1601;S864;S782;S1517;S1519;S1521;S1522;S1893;S562;S564;S1970;S1972;S455;S456;T1531;S808;T810;S1984;S1982;S1012;S817;S231;T233;T885;S895;S1822;T1434;S573;T1990;S1415;S1541;T326;S957;S1501;S854;S2382;S1073;S2456;S1975;T1974;S1083;S2729;S2132;S887;S890;T251;T252;S1110;S702;S704;S706;S972;S454;S834;S759;S761;S1382;Y1820;T359;S395;S1690;T856;S346;S1085;S1383;Y996;S902;S2415;S311;S2675;S2677;S478;S1937;T1939;S2426;T1986;S1674;S1675;S1935;S575;T577;S1577;S1010;T1844;S1848;T1684;S484;S486;					

		1880;S702;S704;S706;T1974;S1682;T1684;S1672;S1674;S1675;S625;S629;S573;S575;T577;S834;S839;S231;T233;S1727;S1729;S1731;T569;S357;S1382;S1061;S1069;S387;S1099;S1326;S1102;T428;S1064;S1008;S818;S248;S250;S1088;S1732;S819;S820;S486;S1110;S957;S2426;S2675;S2677;T1986;T489;S594;S596;T598;S890;T627;S759;S846;S300;S1383;S358;S395;T326;Y1820;S2115;S2456;S1152;S1398;S910;S1233;T2289;S484;S1188;S1083;S1852;S817;S2090;T2092;S1935;S1937;T1939;T1208;S200;S204;S1842;T1844;T1847;S2688;T252;S1791;S1793;S1796;S1794;S1081;T1904;S902;S745;S220;S222;S525;S527;							
Q9UQE7	SMC3	S1067;	S1067;	S1067;	3.37E-05	3.68E-05	2.63E-05	1.28E+00	1.40E+00
Q9Y261	FOXA2	NA	S303;S306;Y405;Y411;T297;	NA	NA	2.91E-05	NA	NA	NA
Q9Y2D8	SSX2IP	NA	NA	S312;	NA	NA	3.44E-06	NA	NA
Q9Y383	LUC7L2	S323;S327;S336;S281;S283;S285;S358;S354;S383;S384;	S336;S358;	S336;S323;S327;S383;S384;S354;S358;	2.09E-04	7.87E-06	3.19E-05	6.56E+00	2.46E-01
Q9Y4H2	IRS2	NA	NA	S915;S1203;S1186;T520;S1162;S1174;S560;S577;S932;S620;	NA	NA	9.28E-05	NA	NA

Q9Y618	NCOR2	S746;S750;S753;T553;S554;S922;S2269;S2234;S2065;S2205;S2208;S2214;S1018;S1025;S956;S149;S152;S1786;S1181;T756;S2057;S2068;S2069;T550;S1783;S1982;S215;S2016;T1391;S1259;	NA	S2065;S2069;S1025;S922;S2269;S2016;S1786;T553;S554;S149;S152;S215;S956;T1391;S1181;S1487;S1432;S2055;S1018;S2214;S2205;S2208;S746;S750;S753;S1783;S939;S943;T1569;	7.98E-04	NA	7.38E-04	1.08E+00	NA
Q9Y6K1	DNMT3A	NA	NA	S105;	NA	NA	2.84E-05	NA	NA
Q9Y6R4	MAP3K4	NA	S499;S72;S84;S1252;	NA	NA	2.07E-05	NA	NA	NA

Note: The differential phosphoproteins of cancer driver genes marked by read.

Table S7: The list for up/down-phosphorylated sites in cancer driver genes

Accession ID	Gene Name	Des	Sites	Mean in L02	Mean in Hep3B	Mean in QGY	QGY/L02	Hep3B/L02
P25054	APC	adenomatous polyposis coli	S1864	8.31E-06	2.91E-05	2.42E-05	2.92E+00	3.50E+00
P25054	APC	adenomatous polyposis coli	S1861	8.31E-06	2.91E-05	2.42E-05	2.92E+00	3.50E+00
P25054	APC	adenomatous polyposis coli	S1863	5.23E-06	2.91E-05	2.42E-05	4.63E+00	5.57E+00
P46100	ATRX	alpha thalassemia/mental retardation syndrome X-linked	S677	1.29E-05	8.86E-05	1.31E-04	1.02E+01	6.88E+00
Q14839	CHD4	chromodomain helicase DNA binding protein 4	S103	2.79E-05	8.45E-05	5.83E-05	2.09E+00	3.02E+00
Q14839	CHD4	chromodomain helicase DNA binding protein 4	S105	2.79E-05	7.87E-05	6.38E-05	2.28E+00	2.82E+00
Q14839	CHD4	chromodomain helicase DNA binding protein 4	S108	2.79E-05	7.87E-05	6.38E-05	2.28E+00	2.82E+00
Q6Y7W6	GIGYF2	GRB10 interacting GYF protein 2	S26	5.08E-05	2.23E-04	2.32E-04	4.57E+00	4.38E+00
Q9Y383	LUC7L2	LUC7-like 2 (S. cerevisiae)	S336	1.36E-06	1.36E-05	4.54E-06	3.33E+00	1.00E+01
Q14676	MDC1	mediator of DNA-damage checkpoint 1	T449	1.65E-04	3.44E-04	4.22E-04	2.56E+00	2.08E+00
Q14676	MDC1	mediator of DNA-damage checkpoint 1	T301	1.25E-05	1.10E-04	4.13E-05	3.30E+00	8.76E+00
Q14676	MDC1	mediator of DNA-damage checkpoint 1	S299	1.25E-05	9.00E-05	4.13E-05	3.30E+00	7.20E+00
P46013	MKI67	marker of proliferation Ki-67	S357	3.68E-05	1.12E-04	2.79E-04	7.59E+00	3.03E+00
P46013	MKI67	marker of proliferation Ki-67	S1131	1.12E-05	6.56E-05	3.07E-04	2.75E+01	5.87E+00
Q15154	PCM1	pericentriolar material 1	S69	8.01E-05	1.60E-04	2.02E-04	2.52E+00	2.00E+00
Q29RF7	PDS5A	PDS5 cohesin associated factor A	S1305	8.40E-05	3.61E-04	1.72E-04	2.05E+00	4.30E+00
P48634	PRRC2A	proline-rich coiled-coil 2A	S761	2.40E-05	1.61E-04	7.85E-05	3.27E+00	6.71E+00
Q5UIP0	RIF1	replication timing regulatory factor 1	S1579	4.20E-05	2.82E-04	8.46E-05	2.01E+00	6.72E+00
Q9UDY2	TJP2	tight junction protein 2	S398	1.79E-05	2.78E-04	5.14E-05	2.87E+00	1.55E+01
Q9UDY2	TJP2	tight junction protein 2	S400	1.79E-05	2.97E-04	5.14E-05	2.87E+00	1.66E+01
Q9UDY2	TJP2	tight junction protein 2	S966	6.99E-06	3.05E-05	2.06E-05	2.95E+00	4.36E+00
P27708	CAD	carbamoyl-phosphate synthetase 2, aspartate transcarbamylase, and dihydroorotase	S1859	8.12E-04	3.27E-04	1.83E-05	2.25E-02	4.03E-01
Q09666	AHNAK	AHNAK nucleoprotein	S210	4.43E-03	1.34E-04	1.08E-03	2.43E-01	3.02E-02
Q09666	AHNAK	AHNAK nucleoprotein	S177	9.68E-04	2.43E-05	3.39E-04	3.50E-01	2.51E-02
Q5SW79	CEP170	centrosomal protein 170kDa	S930	1.03E-04	1.44E-05	2.75E-05	2.68E-01	1.41E-01
Q9C0C2	TNKS1BP1	tankyrase 1 binding protein 1, 182kDa	S691	1.20E-04	2.12E-06	5.01E-05	4.17E-01	1.76E-02
Q12888	TP53BP1	tumor protein p53 binding protein 1	S1759	3.05E-04	5.36E-06	1.79E-05	5.86E-02	1.76E-02
Q12888	TP53BP1	tumor protein p53 binding protein 1	S1430	3.20E-04	1.29E-05	6.16E-05	1.92E-01	4.04E-02
Q12888	TP53BP1	tumor protein p53 binding protein 1	S1426	2.24E-04	1.29E-05	6.16E-05	2.75E-01	5.78E-02

Q96TA1	FAM129B	family with sequence similarity 129, member B	S665	8.16E-04	1.34E-04	7.55E-05	9.26E-02	1.65E-01
Q96TA1	FAM129B	family with sequence similarity 129, member B	S692	1.41E-03	3.93E-04	5.79E-04	4.12E-01	2.80E-01
Q96TA1	FAM129B	family with sequence similarity 129, member B	S696	9.87E-04	3.93E-04	4.86E-04	4.92E-01	3.99E-01
P21333	FLNA	filamin A, alpha	S2152	3.14E-04	2.08E-05	1.57E-05	4.99E-02	6.62E-02
Q7Z6Z7	HUWE1	HECT, UBA and WWE domain containing 1, E3 ubiquitin protein ligase	S2365	1.59E-04	1.84E-05	7.89E-06	4.97E-02	1.16E-01
Q7Z6Z7	HUWE1	HECT, UBA and WWE domain containing 1, E3 ubiquitin protein ligase	S1907	9.11E-04	2.64E-04	6.07E-05	6.66E-02	2.90E-01
Q7Z6Z7	HUWE1	HECT, UBA and WWE domain containing 1, E3 ubiquitin protein ligase	S2362	2.61E-04	2.74E-05	2.47E-05	9.44E-02	1.05E-01
Q9UPN3	MACF1	microtubule-actin crosslinking factor 1	S4521	6.67E-05	1.96E-05	3.10E-05	4.65E-01	2.95E-01
Q9NU22	MDN1	MDN1, midasin homolog (yeast)	S5015	1.75E-04	8.71E-05	3.17E-05	1.81E-01	4.96E-01
P06748	NPM1	nucleophosmin (nucleolar phosphoprotein B23, numatrin)	T219	6.45E-04	2.21E-06	3.54E-05	5.50E-02	3.43E-03
P06748	NPM1	nucleophosmin (nucleolar phosphoprotein B23, numatrin)	S243	9.90E-05	4.67E-05	3.18E-05	3.21E-01	4.72E-01
P35658	NUP214	nucleoporin 214kDa	S678	2.50E-04	6.28E-05	1.12E-04	4.49E-01	2.52E-01
Q8IWS0	PHF6	PHD finger protein 6	S203	1.01E-04	2.68E-05	2.06E-05	2.03E-01	2.64E-01
Q15149	PLEC	plectin	S4626	1.05E-03	1.47E-04	1.70E-04	1.63E-01	1.40E-01
Q15149	PLEC	plectin	S4389	3.25E-04	5.93E-05	1.14E-04	3.50E-01	1.83E-01
Q9H6Z4	RANBP3	RAN binding protein 3	S100	1.60E-04	7.02E-05	2.40E-05	1.50E-01	4.37E-01
Q9NYV4	CDK12	cyclin-dependent kinase 12	S383	1.44E-04	2.59E-05	5.99E-05	4.17E-01	1.80E-01
Q9NYV4	CDK12	cyclin-dependent kinase 12	S385	1.44E-04	2.59E-05	5.99E-05	4.17E-01	1.80E-01
Q9NYV4	CDK12	cyclin-dependent kinase 12	S274	5.16E-05	4.06E-04	1.70E-04	3.30E+00	7.87E+00
Q9NYV4	CDK12	cyclin-dependent kinase 12	S276	5.16E-05	4.06E-04	1.70E-04	3.30E+00	7.87E+00
Q9NYV4	CDK12	cyclin-dependent kinase 12	S334	5.67E-06	6.00E-05	2.54E-05	4.48E+00	1.06E+01
P49792	RANBP2	RAN binding protein 2	T1396	2.06E-04	5.79E-05	7.22E-06	3.51E-02	2.82E-01
P49792	RANBP2	RAN binding protein 2	S1160	4.15E-05	1.61E-05	8.13E-06	1.96E-01	3.88E-01
P49792	RANBP2	RAN binding protein 2	S2741	3.76E-04	1.47E-04	1.39E-04	3.68E-01	3.91E-01
P49792	RANBP2	RAN binding protein 2	S2900	8.50E-06	7.43E-05	3.24E-05	3.81E+00	8.74E+00
Q9NYF8	BCLAF1	BCL2-associated transcription factor 1	S385	1.07E-03	3.61E-04	2.76E-04	2.58E-01	3.37E-01
Q9NYF8	BCLAF1	BCL2-associated transcription factor 1	T257	1.87E-04	9.13E-05	9.20E-05	4.93E-01	4.89E-01
Q9NYF8	BCLAF1	BCL2-associated transcription factor 1	S102	2.49E-05	1.09E-04	1.85E-04	7.40E+00	4.38E+00
Q9NYF8	BCLAF1	BCL2-associated transcription factor 1	S104	2.49E-05	1.09E-04	1.85E-04	7.40E+00	4.38E+00
Q9NYF8	BCLAF1	BCL2-associated transcription factor 1	S760	9.93E-06	9.32E-05	9.91E-05	9.97E+00	9.38E+00

Q9UQ35	SRRM2	serine/arginine repetitive matrix 2	S478	8.90E-04	5.34E-05	1.26E-04	1.41E-01	6.01E-02
Q9UQ35	SRRM2	serine/arginine repetitive matrix 2	S957	7.38E-05	3.66E-05	1.22E-05	1.65E-01	4.96E-01
Q9UQ35	SRRM2	serine/arginine repetitive matrix 2	T2409	1.11E-03	1.35E-04	2.18E-04	1.96E-01	1.21E-01
Q9UQ35	SRRM2	serine/arginine repetitive matrix 2	T326	1.91E-03	4.11E-04	7.15E-04	3.75E-01	2.15E-01
Q9UQ35	SRRM2	serine/arginine repetitive matrix 2	S846	3.02E-04	8.18E-05	1.25E-04	4.13E-01	2.71E-01
Q9UQ35	SRRM2	serine/arginine repetitive matrix 2	T2289	6.77E-05	1.38E-05	3.05E-05	4.50E-01	2.04E-01
Q9UQ35	SRRM2	serine/arginine repetitive matrix 2	T848	2.93E-04	8.24E-07	1.42E-04	4.84E-01	2.81E-03
Q9UQ35	SRRM2	serine/arginine repetitive matrix 2	S1691	1.70E-04	4.15E-04	4.66E-04	2.75E+00	2.44E+00
Q9UQ35	SRRM2	serine/arginine repetitive matrix 2	S1581	7.19E-06	1.64E-05	2.54E-05	3.54E+00	2.28E+00
Q9UQ35	SRRM2	serine/arginine repetitive matrix 2	S1582	7.19E-06	1.64E-05	3.61E-05	5.02E+00	2.28E+00
Q9UQ35	SRRM2	serine/arginine repetitive matrix 2	T1856	1.28E-04	4.59E-04	6.64E-04	5.17E+00	3.57E+00
Q9UQ35	SRRM2	serine/arginine repetitive matrix 2	S1857	1.36E-04	5.32E-04	8.19E-04	6.04E+00	3.93E+00
Q9UQ35	SRRM2	serine/arginine repetitive matrix 2	S2675	2.57E-05	1.39E-04	1.74E-04	6.78E+00	5.43E+00
Q9UQ35	SRRM2	serine/arginine repetitive matrix 2	S2677	2.57E-05	1.39E-04	1.74E-04	6.78E+00	5.43E+00
Q9UQ35	SRRM2	serine/arginine repetitive matrix 2	S566	6.01E-05	2.85E-04	4.18E-04	6.95E+00	4.74E+00
Q9UQ35	SRRM2	serine/arginine repetitive matrix 2	T252	1.10E-05	3.64E-05	8.17E-05	7.43E+00	3.31E+00
Q9UQ35	SRRM2	serine/arginine repetitive matrix 2	S575	1.91E-05	5.21E-04	1.64E-04	8.57E+00	2.73E+01
Q9UQ35	SRRM2	serine/arginine repetitive matrix 2	T577	1.91E-05	5.21E-04	1.64E-04	8.57E+00	2.73E+01
Q9UQ35	SRRM2	serine/arginine repetitive matrix 2	S573	1.74E-05	5.04E-04	1.64E-04	9.43E+00	2.90E+01
Q9UQ35	SRRM2	serine/arginine repetitive matrix 2	S1110	5.23E-05	2.48E-04	5.11E-04	9.78E+00	4.75E+00
Q9UQ35	SRRM2	serine/arginine repetitive matrix 2	S440	6.46E-06	1.50E-05	1.07E-04	1.66E+01	2.33E+00
Q9UQ35	SRRM2	serine/arginine repetitive matrix 2	S817	7.00E-07	5.45E-05	3.08E-05	4.39E+01	7.78E+01
Q9UQ35	SRRM2	serine/arginine repetitive matrix 2	S395	4.42E-06	3.70E-05	2.28E-04	5.17E+01	8.38E+00
Q9UQ35	SRRM2	serine/arginine repetitive matrix 2	S562	7.17E-06	2.85E-04	4.18E-04	5.83E+01	3.97E+01
Q9UQ35	SRRM2	serine/arginine repetitive matrix 2	S564	7.17E-06	2.85E-04	4.18E-04	5.83E+01	3.97E+01

Note: up-phosphorylated proteins in QGY and Hep3B marked by red; down-phosphorylated proteins in QGY and Hep3B marked by green

Table S8: The primers lists of five genes

Gene Name	mRNA ID	Shared exons in all isoform	Primers for forward	Primers for reverse	Exons in special isoform	Primers for forward	Primers for reverse
TP53	CCDS11118.1	Exon4	TGGTAATCTACTGGGACGGA	CTCGCTTAGTGCTCCCTGGG	Exon 3	TCCCCCTTGCCGTCCCAAGC	CGTGCAAGTCACAGACTTGG
KRAS	CCDS8702.1	Exon2	GATTCCTACAGGAAGCAAGT	CTATAATGGTGAATATCTTC	Exon4	AGAGTGGAGGATGCTTTTTA	TTACATTATAATGCATTTTT
NPM1	CCDS4376.1	Exon9	GGACAAGAATCCTTCAAGAA	TTTTTCTATACTTGCTTGCA	Exon8	TCTATACGAGATACTCCAGC	TTTTGATCTTGGTGTTGATG
ATRX	CCDS14434.1	Exon9	GTGGTGTGCGGAAGGTGGAA	TTTGGAGAAAGTCTGGATGT	Exon6	GTACAGTGATTGTACAGCCA	CTCCGCGTTTTTTGAGATTT
MAPT	CCDS45715.1	Exon1	ATGGCTGAGCCCCGCCAGGA	CTTTCAGGCCAGCGTCCGTG	Exon10	GTGAACCTCCAAAATCAGGG	CTTCCCGCCTCCCGGCTGGT
Note: The left of primers is 5’; right is 3’ of primers.							
